# Leveraging Canadian Health Care Worker Volunteers to Address COVID-19 Vaccine Misinformation on Facebook: Qualitative Program Evaluation Study

**DOI:** 10.2196/65361

**Published:** 2025-07-24

**Authors:** Caitlin Ford, Hinna Hasan, Madison Fullerton, Janette Wong, Margaret Pateman, Hao Ming Chen, Theresa Tang, Jia Hu, Kirsten Cornelson

**Affiliations:** 1 19 to Zero Inc, 4702 21 Street SW, Calgary, AB, T2T 5T4, Canada, 1 4162001812; 2 Cumming School of Medicine, University of Calgary, Calgary, AB, Canada; 3 Michael G. DeGroote School of Medicine, McMaster University, Hamilton, ON, Canada; 4 Department of Community Health Sciences, University of Calgary, Calgary, AB, Canada; 5 Faculty of Nursing, Cumming School of Medicine, University of Calgary, Calgary, AB, Canada; 6 Business Economics and Public Policy, Ivey School of Business, Western University, London, ON, Canada

**Keywords:** COVID-19, misinformation, vaccine hesitancy, vaccines, intervention, social media, health care worker, health care workers, SARS-COV-2, Coronavirus, pandemic, COVID-19 vaccine, vaccine misinformation, Canadian, Facebook, semistructured interview, self-efficacy, digital health, mHealth, health informatics

## Abstract

**Background:**

Social media serves as a tool for increased digital interconnectedness and has resulted in playing an instrumental role in sharing health-related information with a wide audience. In conjunction with the vast availability of information, there has been a rapid spread of misinformation, leading to public mistrust, safety concerns, and discrimination. The COVID-19 pandemic has amplified the threat of misinformation resulting in detrimental health outcomes due to individuals becoming fatigued with COVID-19 health guidance. Although vaccinations are the key to combating COVID-19, the overwhelming amount of misinformation has resulted in diminished vaccine acceptance.

**Objective:**

This study aims to (1) train and deploy a group of health care workers and student volunteers to address antivaccine sentiment on Facebook (Meta Platforms, Inc) and (2) evaluate the intervention through semistructured interviews to determine lessons learned and suggestions for future initiatives to address internet-based misinformation online.

**Methods:**

The project used volunteers to address vaccine-hesitant comments on Facebook (Meta Platforms, Inc), with the overall goal of empowering health care professionals to counteract the spread of vaccine misinformation. Eligible participants included health care workers and students in health care–related disciplines recruited through social media and email advertising campaigns. Informational training sessions followed, to better equip volunteers with the ability to use their working knowledge of health communication and behavior change to correct web-based misinformation. The volunteers were provided a file containing Facebook posts that discussed COVID-19 vaccines to act as a starting point for leaving or responding to comments that spread vaccine misinformation. Participants were provided with working knowledge of health communication, behavior change, and correct misinformation through the informational training sessions. Qualitative evaluation in the form of interviews was used to examine participant experiences.

**Results:**

Three main themes emerged regarding the project’s format and training model, the factors motivating volunteers to participate, and overall experiences tackling misinformation on a social media platform. The first theme showcased that the training format was effective due to its use of interactive components and overall flexibility, resulting in it being well received by volunteers. The second identified theme highlighted that a main driving factor for participation included a balance of professional development and societal good. The third theme revealed that the volunteers’ experiences in interacting with the public revealed a rich tapestry of emotions and perspectives, where vaccine hesitancy is interconnected with emotional responses and personal beliefs.

**Conclusions:**

The Informed Choice Project provided an opportunity to increase self-efficacy and confidence for more than a dozen health care professionals and students while engaging in vaccine-related conversations on social media. To enhance both participant satisfaction and compliance, future interventions should consider using a self-paced format, flexible hours, and highlight the vitality of health care professionals as key advocates for trusted sources of information for the public.

## Introduction

The digital age has revolutionized how information is disseminated. Social media platforms have connected individuals across the globe with unparalleled speed and reach. This digital interconnectedness has allowed social media to be a useful catalyst for sharing health information in the last 2 decades [1]. While social media platforms have enhanced information accessibility, they have also accelerated the dissemination of misinformation, leading to increased public mistrust, safety concerns, and instances of discrimination [2]. Misinformation is not new, but the rise of social media has amplified its impact, particularly during the COVID-19 pandemic. This infodemic was fueled by social media platforms, where accounts used their audiences to spread controversial advice and opinions [3].

With an overwhelming amount of information, individuals have become desensitized to the health risks of COVID-19, influencing an individual’s choice in adhering to health guidelines such as handwashing, social distancing, mask mandates, and vaccination [4]. Despite vaccines being widely recognized as a key public health intervention, individuals’ vaccination decisions have been influenced by factors such as concerns about vaccine safety and efficacy, complacency or a low perceived risk of illness, and the inconvenience of accessing vaccination services [5]. Efforts to address concerns and highlight the safety and efficacy of COVID-19 vaccines have been met with significant challenges, as the spread of misinformation on social media has hindered vaccine uptake [6].

As a result, multiple strategies have been implemented to identify vaccine misinformation. In 2020, Fondazione Bruno Kessler studied 112 million COVID-19 social media posts and found that more than 40% of posts were cited from unreliable sources while 42% of posts were controlled through bots, an automated computer program that interacts within a network that can spread spam or a virus [7,8]. In 2020, Naeem and Brideman analyzed 620,000 tweets related to COVID-19 and discovered that the use of social media was correlated with misinterpretations of the pandemic [7]. Health care providers have been identified as a key group to address web-based vaccine misinformation [9]; however, there has been limited effort to use their potential in effectively combating these false vaccine narratives. Therefore, in efforts to address the infodemic and help stop the spread of COVID-19 misinformation on social media, the objectives of this project are to (1) train and deploy a group of health care workers and student volunteers to address antivaccine sentiment on Facebook (Meta Platforms, Inc), and (2) to evaluate the intervention through semistructured interviews to determine lessons learned and suggestions for future initiatives to address misinformation on social media platforms.

## Methods

### Project Overview

The Informed Choice Project was initiated to combat misinformation by having volunteers address vaccine-hesitant comments on the social media platform, Facebook. The goal of the Informed Choice Project was to directly address vaccine hesitancy sentiment (eg, comments on Facebook posts) by empowering health care professionals to engage with vaccine-hesitant individuals and counteract the spread of vaccine misinformation through tailored messaging.

### Volunteer Recruitment

Participants were recruited using social media and email advertising and the University of Calgary, School of Nursing student contact list, ensuring adherence to ethical guidelines and informed consent protocols. Eligible participants were health care workers and students in health care–related disciplines (eg, doctors, nurses, pharmacists, and postsecondary students interested in health care). Those who were interested in participating self-identified by contacting a member of the research team. As an honorarium for participating, volunteers were given CAD $300 (US $222) for 30 hours of volunteer work.

### Information and Training Sessions

Participants who expressed interest in the project attended an online Informed Choice Project information session followed by one 30-minute training session through the platform Zoom software (version 5.14.12; Zoom Video Communications, Inc). Prior to attending the training session, volunteers were asked to review a training manual (Multimedia Appendix 1) outlining behavioral principles for addressing vaccine hesitancy, strategies for identifying productive interaction opportunities, and tips for how the volunteers could safely engage on social media. Both the information and training sessions included comprehensive briefings on the project’s objectives, data collection methods, ethical considerations, and an example of how to engage in vaccine conversations with social media users. These sessions were conducted by the project team and were designed to equip participants with the necessary knowledge and skills for their involvement using behavior change theory principles. There were several opportunities throughout the session for volunteers to actively participate and practice. At the end of the session, participants were expected to have a working knowledge of health communication, behavior change, and correcting misinformation.

### Deployment of Volunteers

From January to August 2023, the Informed Choice Project volunteers engaged in conversations with individuals posting antivaccine comments and on social media. The research team provided volunteers with a file containing a list of Facebook posts that discussed COVID-19 vaccines (Multimedia Appendix 2). The list was compiled by searching the CrowdTangle database (Meta) for public posts from American or Canadian accounts containing keywords related to the vaccine. CrowdTangle is a tool provided by Meta that allows access to information about what is happening on public accounts on their social media platforms. This tool allows researchers to track the performance of different accounts and use search terms to view popular social media content (Multimedia Appendix 3). CrowdTangle only provides information that is already publicly available through Facebook (eg, the name of a person running a public account, number of likes, keyword searching, engagement histories, and so on).

Volunteers were asked to engage in at least 3‐5 conversations per week, in which they accessed a relevant Facebook post and scrolled through comments before deciding whether to either (1) leave a new comment on the thread or (2) respond to an existing comment. Once they decided, volunteers opened a web-based online survey tool (Qualtrics) and entered information about the post or comment, including their name, the text of the comment, the username of the original poster, and a link to the post. The survey then randomly instructed the volunteer to leave or not leave a response through the web-based survey’s built-in randomization function (Qualtrics). If they were told to leave a response, the survey asked them to type their response into a text box. To assist them in crafting their responses, volunteers were provided with an FAQ (frequently asked question) document that provided suggested responses and links to information on key vaccine issues.

### Qualitative Evaluation: Participant Experience Interviews

To develop an understanding of the volunteers’ experience with the Informed Choice Project, the research team conducted a series of semistructured interviews. Volunteers were contacted via email and were asked to attend a 30-minute online interview with members of the research team via Zoom software (version 5.14.12). The interviews were conducted using a standardized interview guide developed specifically for this project (Multimedia Appendix 4). The guide was designed to capture relevant information pertaining to the volunteer’s experience within the project. Questions were structured to facilitate clear and consistent data collection across all participants. A facilitator and moderator met volunteers on Zoom software, individually, and audiorecorded and transcribed the conversation.

### Participant Experience Interview Analysis

Following the completion of interviews, the facilitator and moderator independently reviewed the transcripts and inductively developed a set of codes to represent the dataset. A final codebook was agreed upon by both researchers (Multimedia Appendix 5), and one researcher coded the transcripts independently. Once coded, 2 researchers agreed on the coding, and a reflexive thematic analysis was conducted by each researcher independently to identify common themes across the dataset [10]. The final list of themes was agreed upon by the research team.

### Ethical Considerations

Ethical approval was obtained from the University of Calgary Joint Ethics Board (REB22-0315_REN1). Prior to beginning the interviews, participants were read a consent script that outlined their ability to withdraw from the study at any time. All participants provided verbal consent to proceed with the interviews. Interviews were recorded and transcribed verbatim; however, no identifying information was included in the transcripts. In alignment with institutional ethics requirements, the interview data were destroyed once transcription was complete. As an honorarium for participating in the interview, volunteers were offered a CAD $50 (US $37) gift card.

## Results

### Informed Choice Project Volunteer Enrollment and Retention

In total, 8108 posts were included in the dataset and 716 were responded to by volunteers. After 60 people attended the Informed Choice Project training sessions, 14 volunteers actively participated in the project and engaged in social media discussions about COVID-19 vaccine misinformation. Of these 14 initial volunteers, 8 were retained until the end of the project (August 2023) and participated in the evaluative interviews.

### Themes From Volunteer Experience Interviews

From the interviews, 3 key themes emerged surrounding the Informed Choice Project’s format and training model, the drivers for volunteers to participate, and their experiences interacting with the public on social media to address misinformation (Table 1).

**Table 1.** Postprogram semistructured interviews with health care worker volunteers: themes and subthemes generated from reflexive thematic analysis.

Themes and subthemes	Key quotes	
The program’s model and training were effective and well-received by participants.		
The volunteers learned well from interactive components.	“I think the training sessions were really good. I enjoyed them. They went through everything, and then I'm pretty sure after that you sent everything to us anyway, so it was really handy to go back and recheck what I was unsure of. And I think after the initial training sessions you had more training sessions, which was really good as well, so I really liked them. They were really helpful.” – Participant 1	
Volunteers felt that strategies for interacting with the public were effective.	“It was very different from anything that I've ever seen before or participated in. I feel like having the online format was very unique. And I'm sure there's many similar studies that are kinda maybe chatting with people in person, one on one, but I feel like people are a lot more intense with their feelings about this topic online. So it was cool to try it in that format. [...] It was very well organized, and I felt like writing answers was pretty easy given the information that we had. I also referred... I referred a lot to the Frequently Asked Questions document and I also referred a lot to the Q&A website on the WHO website.” – Participant 3	
There were limited barriers to completing the training. The self-paced model provided flexibility for participants, despite the risks of procrastination.	1. “My schedule can be quite variable. So, that was really appreciated [...] for my purposes and my personal life, this format worked actually quite well.” – Participant 8 2. “I actually liked it a lot just because of the freedom of it and there's not many constraints.[...] The main thing that I haven't liked is being in charge of my own schedule just because I'm a really big procrastinator. If there were set times, or set days where everyone else was doing it as well, I feel like I would kinda schedule the time out for it and have more hours [completed] by now” – Participant 1	
The motivation to participate came from a balance of professional development and societal good.		
Many saw this as an opportunity to contribute meaningfully to a public health crisis.	1. “I think that it's an opportunity where I can engage with the people, where I can communicate with them, and where I can educate them regarding vaccinations so that I can help them to remove all the misinformation that they have. So that's a good opportunity where I could promote health and promote vaccination” – Participant 2 2. “The incentive [to participate] wasn't the money for most people I believe. I believe the incentive was that it's either really personal to them, or as professionals they wanted to reach as many people as they could, and it's a really good idea.” – Participant 6	
Some were motivated by the chance to develop skills that would be beneficial in their healthcare careers.	1. “I just felt that [the Informed Choice Project] was really relevant to both what I was learning in school and what I was going to be doing in practice and I thought that it would just be good to, you know, brush up on strategies for discussing vaccine hesitancy and things like that with patients.” – Participant 4 2. “I was really interested in finding out ways of addressing misinformation and importantly, disseminate better information.” – Participant 8	
The volunteers' experiences in interacting with the public revealed a rich tapestry of emotions and perspectives.		
Volunteers found that most public anger was fuelled by fear of the unknown.	“The media barrage of problems was scaring people. It was either, ‘don't get it because there's side effects and it does this or that, or it was there's no way to control it, it's out of control, you're gonna die’. It was just, there was no medium in there, you know. There was no, ‘okay, this is what we do, this is our plan, this is how we're gonna try to keep people healthy’. So, my whole interaction when I did have a chance to interact with people on social media was not just as a patient, but also as a healthcare provider. And if you tell someone you're a pharmacist, they will listen to you if you give them the time and you're patient with them.” – Participant 6	
Misinformation was a major driver for public discourse and anti-vaccine sentiment.	“A lot of the posts that I came across would be like a link to a news article, and then I didn't often read the articles that they shared, but they were clearly not real news websites. They were like blogs or just, you know, fake news [...]. And people would engage with that post as if it were like a real news website and it was just very confusing to me.” – Participant 3	
Volunteers observed the public’s lack of trust in the establishment, yet a strong trust in their inner circles.	“I noticed that despite, even in my colloquial circles, people talk about trust and mistrust and how a lot of the conspiracy theorists are mistrustful. But I found it quite interesting, conversely actually, they're very trustful. But it's these very situational, certain people that they trust [...]. They seem to have a smaller network of closer knit people, unfortunately seem to be fact, just because they all think alike, and then this is their trust network. But this trust network supersedes almost anything that institutions, organizations or anything would actually tell them, and they will refute anything with almost anecdotal evidence.” – Participant 8	
Personal experiences drove emotional reactions online and helped volunteers understand and empathize.	“I used to think that a lot of the misinformation and opinions are based on what they're reading online, but it often can come down to experience and interpretation of experiences, whether it's a loved one or a friend, and things like that. So what I gathered was to kind of be cognizant of their background and what knowledge they're coming from, and then kind of challenge certain assumptions that are made, and kind of go from there.” – Participant 5	

### Theme 1: The Informed Choice Project’s Model and Training Format Was Effective and Well Received by Participants

Overall, the volunteers had positive opinions on how the Informed Choice Project was structured. Participants felt that the training was helpful and provided all the necessary information to begin correcting misinformation. The interactive components of the training were praised for their effectiveness and participants felt that the training leads were accessible to troubleshoot and provide clarification once the project was underway. Some participants identified that the self-paced model presented challenges such as procrastination, distractions from personal life, and occasional technological issues. However, a majority of participants appreciated the flexibility that this model offered and identified that it allowed them to continue volunteering despite busy and varied schedules.

The strengths were definitely the relevancy of what we were discussing and learning. I felt that was super applicable to the field that I’m in and the co-op rotation that I was doing. Again, the self-guided or self-directed learning, where you could go at your own pace, it wasn’t something where you had to sit down for two or three hours and do it in one shot, which I thought was really nice, especially because when I was doing it I was in the middle of working and didn’t have a whole bunch of free time on my hands. [Participant 4]

### Theme 2: The Motivation to Participate Came From a Balance of Professional Development and Societal Good

The volunteers’ reasons for participating were deeply rooted in both their personal and professional spheres. Several participants felt that this opportunity would provide the chance to develop skills that would benefit their careers in health care, and some stated that they would be applying their experiences from the Informed Choice Project to communicate with their patients about vaccines. Others saw this as an opportunity to contribute meaningfully to a public health crisis and wanted to do their part in preventing the spread of COVID-19. This dual motivation was a driving force behind their active participation and commitment to the project. However, most participants expressed that the impact on society and their patients was the key factor, which motivated them.

I thought it was an amazing opportunity, and also very timely because, outside of school, I was in the pharmacy administering COVID-19 injections, so at that time there were always conversations when patients were not so willing, or they had apprehension. So I thought this training definitely prepared me very well for that. [Participant 5]

### Theme 3: The Volunteers’ Experiences in Interacting With the Public Revealed a Rich Tapestry of Emotions and Perspectives

The volunteers’ experiences in interacting with the public revealed a rich tapestry of emotions and perspectives. The vaccine hesitancy they encountered was not just a matter of misinformation but also deeply tied to emotional responses and personal beliefs. This experience was eye-opening for many volunteers, as it broadened their understanding of the public’s fears and hesitations.

[Reading online comments] made me feel like, ‘Oh my god, these people are so angry. I don’t understand.’ Just being a pretty scientifically minded person. But then as time went on, I would say, I actually started to gain a lot of empathy for those people and just started to notice that they’re just really scared and confused. [Participant 3]

## Discussion

### Principal Findings

The Informed Choice Project, set against the backdrop of the COVID-19 pandemic, represents a pivotal effort in addressing the surge of misinformation in the digital age. Conversations on social media surrounding COVID-19 vaccines were underscored by “COVID fatigue,” or the general weariness toward pandemic-related news and guidelines likely influenced both the public’s reception of health information [11,12]. Furthermore, the highly politicized nature of pandemic discourse, especially surrounding vaccines, added another layer of complexity to public discourse [13]. To combat these issues, global calls have been made for health care providers to address vaccine misinformation on social media [9]. A meta-analysis conducted prior to the COVID-19 pandemic identified that on social media, misinformation debunking interventions are most effective when they come from credible experts [14], and a recent study found that when COVID-19 misinformation comments on social media were refuted by a reputable source, such as the Centers for Disease Control and Infection—vaccine misperceptions shifted [15].

Some efforts to address COVID-19 misinformation on social media have been documented, including providing “myth busting” informative posts [16]. However, most research has investigated the impact on social media COVID-19 misinformation organically, observationally, and without intervention [7,8,17-19]. As such, the Informed Choice Project aimed to fill the gap using a unique and evidence-informed approach to correcting COVID-19 misinformation on social media, by using health care providers to engage in personal contact with those perpetuating these messages.

### Program Strengths

Overall, the Informed Choice Project was considered a positive experience by volunteers who completed the evaluative interviews. Volunteers who felt that this work made a difference in the web-based vaccine conversation landscape were retained through this process. Most notably, participants felt that this opportunity allowed them to increase their confidence and self-efficacy in having vaccine conversations, both on social media and in health care settings. These volunteers appreciated the self-paced format for participating, and despite minor technological hiccups and procrastination, completed the required hours without major barriers. As health care professionals, the Informed Choice Project model allowed them to participate in an extracurricular volunteer activity, without imposing clinical obligations.

This project has not only contributed to the immediate goal of addressing vaccine hesitancy but has also provided valuable insights into the dynamics of public engagement and communication in the era of digital interconnectedness. Our findings contribute to the growing understanding of the drivers for vaccine hesitancy among health care providers, which has notably increased empathy and upskilled these individuals for having conversations with vaccine-hesitant individuals in their clinical practice.

### Program Limitations and Lessons Learned

One of the main limitations to completing the Informed Choice Project was the introduction of Canada’s federal Bill C-18, which prompted Meta to block news media content to Canadian social media platform users, including on Facebook [20]. This legislation was adopted in the Summer of 2023, at which point all news content was removed from Canadian Facebook users’ feeds [21]. This legislation severely limits the content that Informed Choice Project volunteers can access and respond to.

Another challenge experienced through this project was retaining volunteers. The Informed Choice Project information sessions had a good attendance rate (n=60); however, onboarding and retaining volunteers was challenging as the project had 14 volunteers complete their required hours and 8 volunteers participate in evaluative interviews. Given the high number of vaccine-related posts on Facebook, higher participation rates would have garnered better correction of misinformation. Thus, recruitment strategies would need to be reconsidered for future efforts.

Finally, there exist limitations in the impact that the Informed Choice Project has on Facebook audiences. The most important group to target vaccine communications efforts is the “moveable middle”—defined as a group of unvaccinated individuals who are unsure about getting the vaccine, but have a mid-range probability of receiving it [22]. True “antivaccine” audiences have entrenched their views in echo chambers and confirmation bias by the content they consume on social media [23,24]. These audiences may respond better to community-based vaccine promotion, namely from members of their trusted networks [25]. While volunteers were instructed to focus on comments from vaccine-hesitant, rather than antivaccine, individuals, many of their responses in practice were to the latter group. As such, targeting individuals who have engaged in blatant COVID-19 vaccine fear-mongering and misinformation spread on Facebook may not be the most efficient use of health care provider time and resources.

### Recommendations for Future Initiatives

Based on the lessons learned throughout the project, the Informed Choice Project model would lend itself well to training and empowering health care providers and health volunteers to address health-related topics in web-based discussions. Based on the qualitative evaluation of this project, the following suggestions can be made for future efforts:

1. Enhanced support systems: implementing more robust support systems for volunteers, such as mentorship programs or peer discussion forums [26], could alleviate the emotional toll of dealing with vaccine misinformation and hesitancy. Strategies like regularly scheduled training sessions might have provided more structure, aiding volunteers in managing their time more effectively [27], and alleviated some of this burden. Furthermore, a more interactive platform for volunteer collaboration could have fostered a sense of community and support, which is crucial in such emotionally taxing work [26].
2. Adaptation to political and social contexts: future projects should consider the prevailing political and social climates, adapting their strategies accordingly. This might involve tailoring communication to be sensitive to political sentiments and public weariness toward pandemic information [28] and ensuring the timing of the project is appropriate to the current context, as our project was funded 2 years after the pandemic. Furthermore, as with Bill C-18 [20], political landscapes can disrupt communication channels and impact the way people receive information. Thus, programs must be nimble and use contingency plans to ensure that misinformation continues to be addressed using alternative methods.
3. Engagement with broader audiences: expanding the reach of such projects beyond the immediate circle of health care professionals and students could bring in diverse perspectives and approaches to tackling misinformation [29].

### Conclusions

The Informed Choice Project was a collaborative, innovative, and adaptive effort to mitigate the harms of vaccine information on social media during the COVID-19 pandemic. The program upskilled more than a dozen health care professionals and students to engage in vaccine conversations on social media, and the resulting increase in self-efficacy and confidence for having these conversations was a major success. This project not only addressed the immediate challenges of the COVID-19 pandemic but also set the foundation for stronger, more informed future public health communication strategies. Future interventions should consider adopting a similar, self-paced format and flexible hours to ensure participant satisfaction and compliance. However, considerations must be made to increase recruitment and retention of volunteers, and while choosing appropriate social media platforms for engagement. The lessons learned through this evaluation highlight the potential for even greater impact for health care providers to continue to be key advocates for vaccination and trusted sources of information for the public. As the world navigates through an evolving digital and social landscape, the Informed Choice Project can serve as a roadmap for future initiatives to promote vaccination and combat misinformation.

## Supplementary material

10.2196/65361 Multimedia Appendix 1 Volunteer Training Manual.

10.2196/65361 Multimedia Appendix 2 Post ID and engagement.

10.2196/65361 Multimedia Appendix 3 Search terms.

10.2196/65361 Multimedia Appendix 4 Volunteer Interview Guide.

10.2196/65361 Multimedia Appendix 5 Codebook.
