# Supplementary material for: Leveraging Canadian Health Care Worker Volunteers to Address COVID-19 Vaccine Misinformation on Facebook: Qualitative Program Evaluation Study
Source: J Med Internet Res. 2025 Jul 24;27:e65361. doi: 10.2196/65361 (PMC12288766; doi:10.2196/65361)
Supplement: Multimedia Appendix 1 [file jmir-v27-e65361-s001.pdf]

19 TO ZERO
UNITED AGAINST COVID-19

World Health Organization

THE INFORMED CHOICE PROJECT

Strategies For Building Vaccine Confidence And Addressing Misinformation Online

Table of Contents

1. Volunteer Orientation
2. Building Vaccine Confidence: Behavioral Principles
3. Social Media Personas
4. Social Media Security

19 to Zero: Building Vaccine Confidence Online

- Our aim is to address misinformation on social media, in order to increase vaccine confidence and vaccine uptake
- You are part of a network of healthcare workers and volunteers who will actively engage with vaccine-hesitant comments & posts on social media

Your Role

- We will ask you to sign up for shifts totalling 3-5 hours per week. We also ask that you keep conversations going throughout the week, as you are able to find time.
- In this module, we will take you through the actions you will follow as you work your shifts.
- The following modules will provide guidance on how to engage, and who to engage with.

Step 1: Open your key documents

1. Social media guide
2. To-do list
3. FAQ

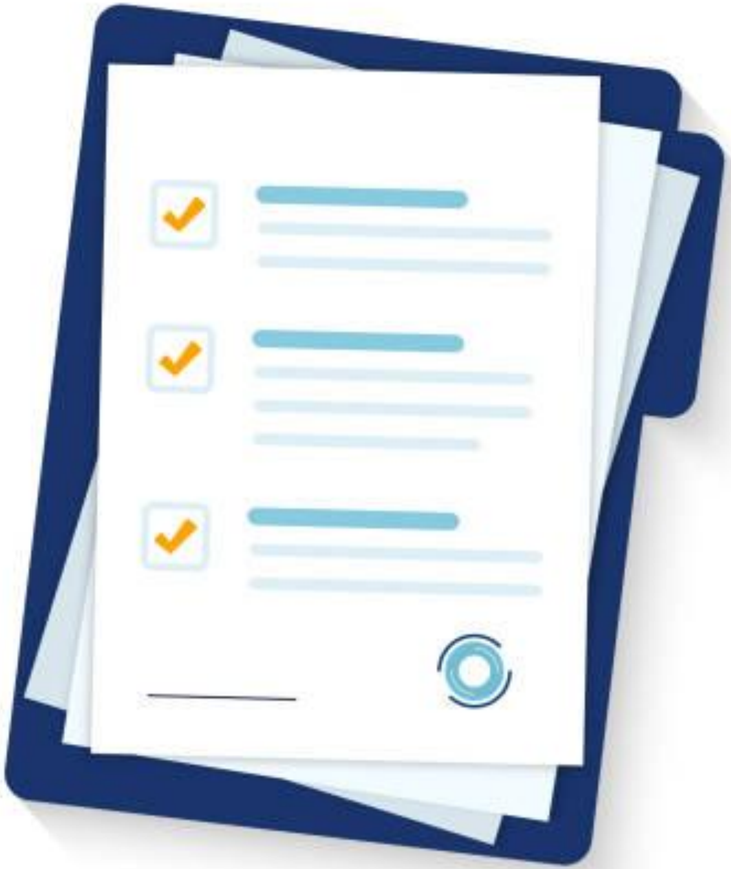

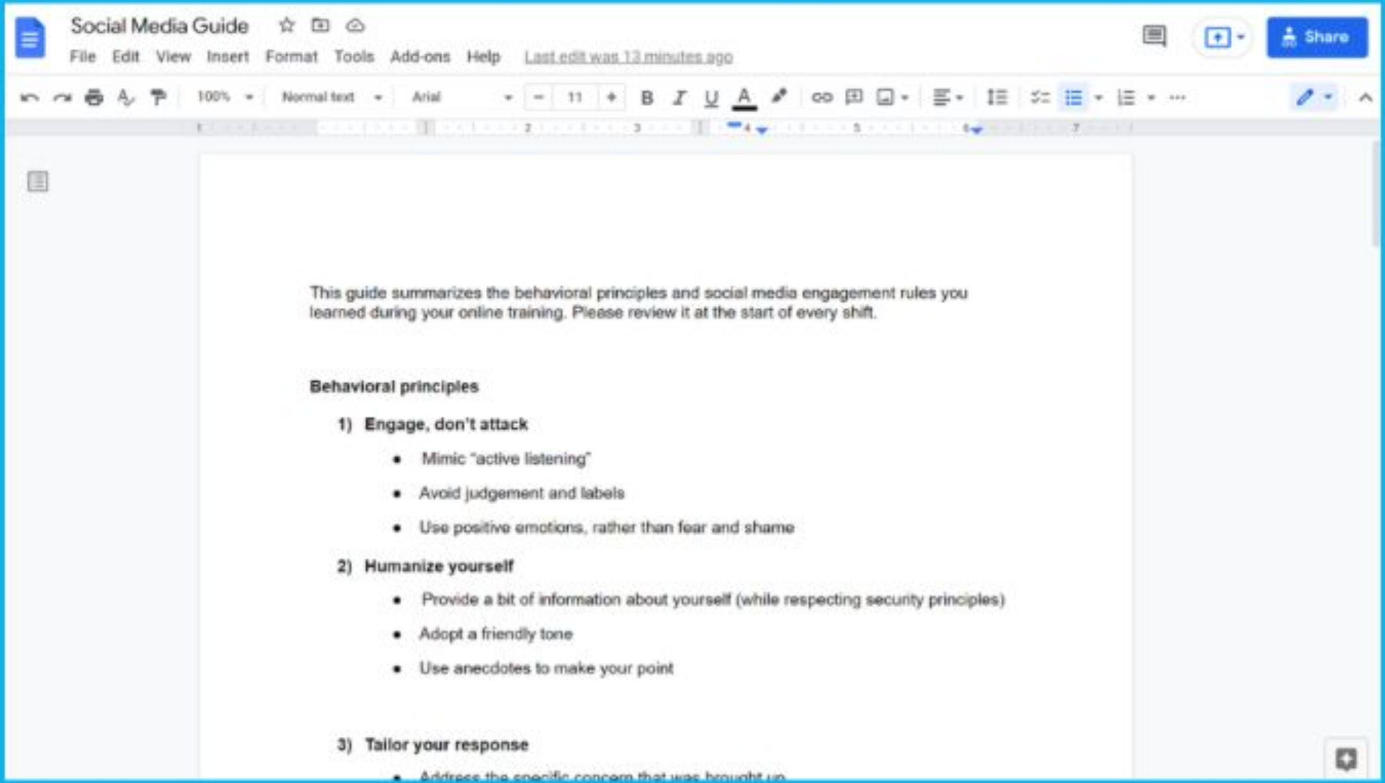

Social Media Guide

File Edit View Insert Format Tools Add-ons Help Last edit was 13 minutes ago

This guide summarizes the behavioral principles and social media engagement rules you learned during your online training. Please review it at the start of every shift.

Behavioral principles

1) **Engage, don't attack**
   - Mimic "active listening"
   - Avoid judgement and labels
   - Use positive emotions, rather than fear and shame
2) **Humanize yourself**
   - Provide a bit of information about yourself (while respecting security principles)
   - Adopt a friendly tone
   - Use anecdotes to make your point
3) **Tailor your response**
   - Address the specific concern that was brought up

The Social Media Guide summarizes the information that you will learn in the next two modules about how to engage nline. Please review it before starting a conversation to make sure your comments follow these principles. You can find a link to the SMG in the Instructions Sheet.

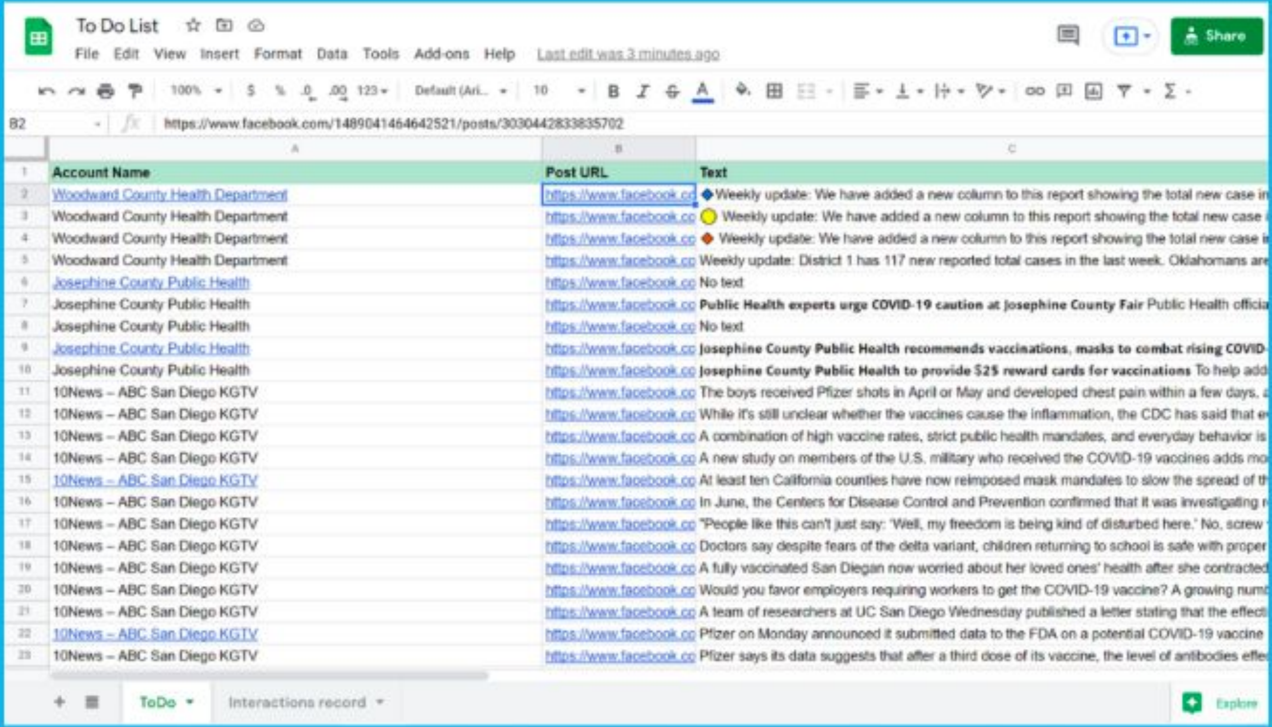

To Do List

File Edit View Insert Format Data Tools Add-ons Help Last edit was 3 minutes ago

B2 https://www.facebook.com/1489041464642521/posts/3030442833835702

	Account Name	Post URL	Text
2	Woodward County Health Department	https://www.facebook.co	◆Weekly update: We have added a new column to this report showing the total new case in
3	Woodward County Health Department	https://www.facebook.co	Weekly update: We have added a new column to this report showing the total new case
4	Woodward County Health Department	https://www.facebook.co	◆ Weekly update: We have added a new column to this report showing the total new case i
5	Woodward County Health Department	https://www.facebook.co	Weekly update: District 1 has 117 new reported total cases in the last week. Oklahomans are
6	Josephine County Public Health	https://www.facebook.co	No text
7	Josephine County Public Health	https://www.facebook.co	**Public Health experts urge COVID-19 caution at Josephine County Fair** Public Health officia
8	Josephine County Public Health	https://www.facebook.co	No text
9	Josephine County Public Health	https://www.facebook.co	**Josephine County Public Health recommends vaccinations, masks to combat rising COVID-**
10	Josephine County Public Health	https://www.facebook.co	**Josephine County Public Health to provide $25 reward cards for vaccinations** To help add
11	10News – ABC San Diego KGTV	https://www.facebook.co	The boys received Pfizer shots in April or May and developed chest pain within a few days, a
12	10News – ABC San Diego KGTV	https://www.facebook.co	While it's still unclear whether the vaccines cause the inflammation, the CDC has said that e
13	10News – ABC San Diego KGTV	https://www.facebook.co	A combination of high vaccine rates, strict public health mandates, and everyday behavior is
14	10News – ABC San Diego KGTV	https://www.facebook.co	A new study on members of the U.S. military who received the COVID-19 vaccines adds mo
15	10News – ABC San Diego KGTV	https://www.facebook.co	At least ten California counties have now reimposed mask mandates to slow the spread of th
16	10News – ABC San Diego KGTV	https://www.facebook.co	In June, the Centers for Disease Control and Prevention confirmed that it was investigating r
17	10News – ABC San Diego KGTV	https://www.facebook.co	"People like this can't just say: 'Well, my freedom is being kind of disturbed here.' No, screw
18	10News – ABC San Diego KGTV	https://www.facebook.co	Doctors say despite fears of the delta variant, children returning to school is safe with proper
19	10News – ABC San Diego KGTV	https://www.facebook.co	A fully vaccinated San Diegan now worried about her loved ones' health after she contracted
20	10News – ABC San Diego KGTV	https://www.facebook.co	Would you favor employers requiring workers to get the COVID-19 vaccine? A growing numb
21	10News – ABC San Diego KGTV	https://www.facebook.co	A team of researchers at UC San Diego Wednesday published a letter stating that the effecti
22	10News – ABC San Diego KGTV	https://www.facebook.co	Pfizer on Monday announced it submitted data to the FDA on a potential COVID-19 vaccine
23	10News – ABC San Diego KGTV	https://www.facebook.co	Pfizer says its data suggests that after a third dose of its vaccine, the level of antibodies effe

ToDo Interactions record Explore

The To-Do list is a Google Sheet containing a list of Facebook posts that may contain misinformation, either in the post or in the comments. You will use this to find places to leave comments. You will find a link to the To-Do List in the Instructions Sheet.

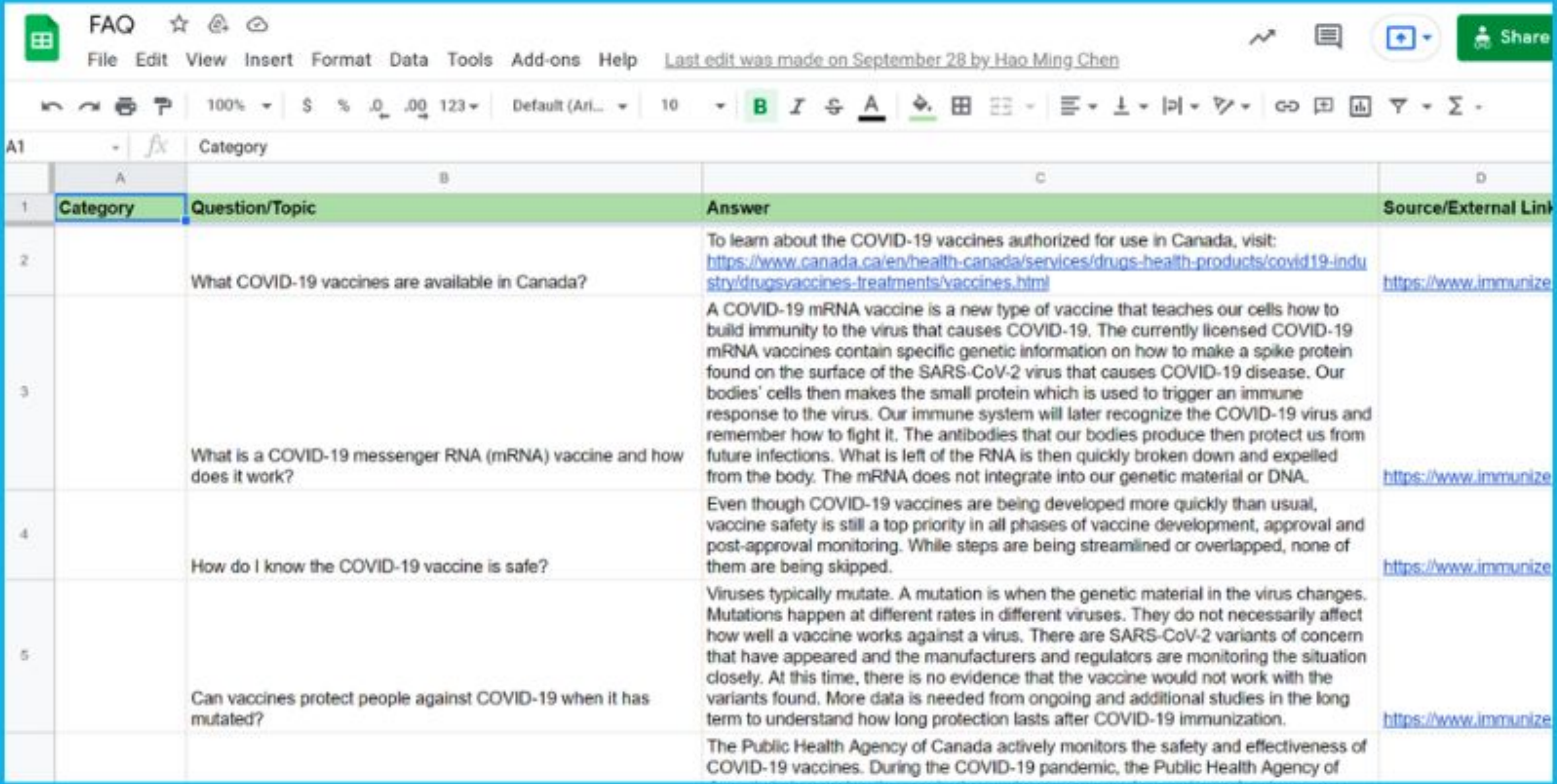

Category	Question/Topic	Answer	Source/External Link
	What COVID-19 vaccines are available in Canada?	To learn about the COVID-19 vaccines authorized for use in Canada, visit: https://www.canada.ca/en/health-canada/services/drugs-health-products/covid19-industry/drugsvaccines-treatments/vaccines.html	https://www.immunize
	What is a COVID-19 messenger RNA (mRNA) vaccine and how does it work?	A COVID-19 mRNA vaccine is a new type of vaccine that teaches our cells how to build immunity to the virus that causes COVID-19. The currently licensed COVID-19 mRNA vaccines contain specific genetic information on how to make a spike protein found on the surface of the SARS-CoV-2 virus that causes COVID-19 disease. Our bodies' cells then makes the small protein which is used to trigger an immune response to the virus. Our immune system will later recognize the COVID-19 virus and remember how to fight it. The antibodies that our bodies produce then protect us from future infections. What is left of the RNA is then quickly broken down and expelled from the body. The mRNA does not integrate into our genetic material or DNA.	https://www.immunize
	How do I know the COVID-19 vaccine is safe?	Even though COVID-19 vaccines are being developed more quickly than usual, vaccine safety is still a top priority in all phases of vaccine development, approval and post-approval monitoring. While steps are being streamlined or overlapped, none of them are being skipped.	https://www.immunize
	Can vaccines protect people against COVID-19 when it has mutated?	Viruses typically mutate. A mutation is when the genetic material in the virus changes. Mutations happen at different rates in different viruses. They do not necessarily affect how well a vaccine works against a virus. There are SARS-CoV-2 variants of concern that have appeared and the manufacturers and regulators are monitoring the situation closely. At this time, there is no evidence that the vaccine would not work with the variants found. More data is needed from ongoing and additional studies in the long term to understand how long protection lasts after COVID-19 immunization.	https://www.immunize
		The Public Health Agency of Canada actively monitors the safety and effectiveness of COVID-19 vaccines. During the COVID-19 pandemic, the Public Health Agency of [illegible]	

The FAQ provides a list of commonly asked questions about the COVID-19 vaccines, with answers and links to sources.
You can use this for reference. You will find a link to the FAQ in the Instructions Sheet.

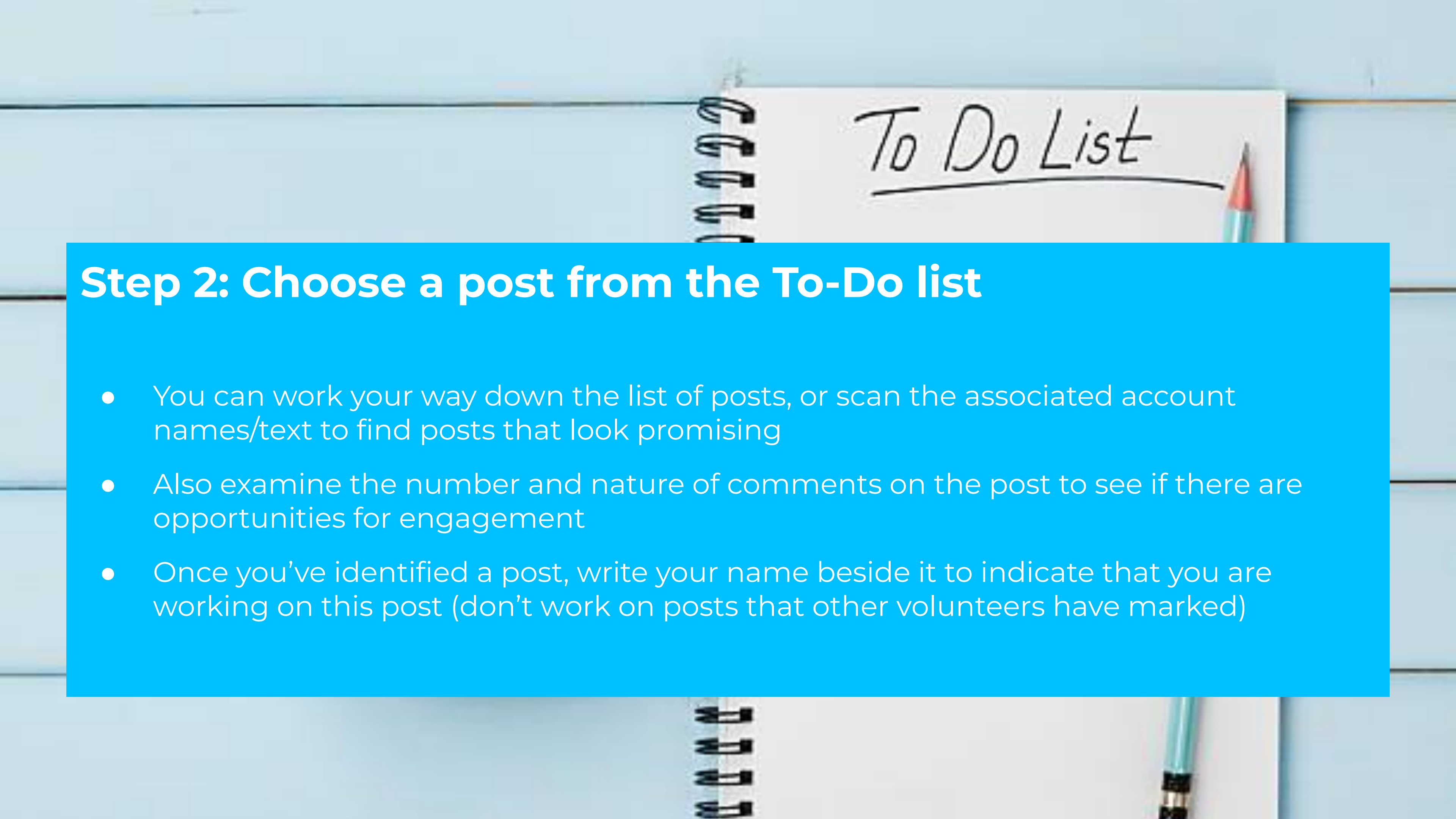

Step 2: Choose a post from the To-Do list

- You can work your way down the list of posts, or scan the associated account names/text to find posts that look promising
- Also examine the number and nature of comments on the post to see if there are opportunities for engagement
- Once you’ve identified a post, write your name beside it to indicate that you are working on this post (don’t work on posts that other volunteers have marked)

Step 3: Find a place to engage, and use the randomizer

- Read through the comments more carefully until you find one that you would like to engage with
- Enter information about the comment into the randomization tool

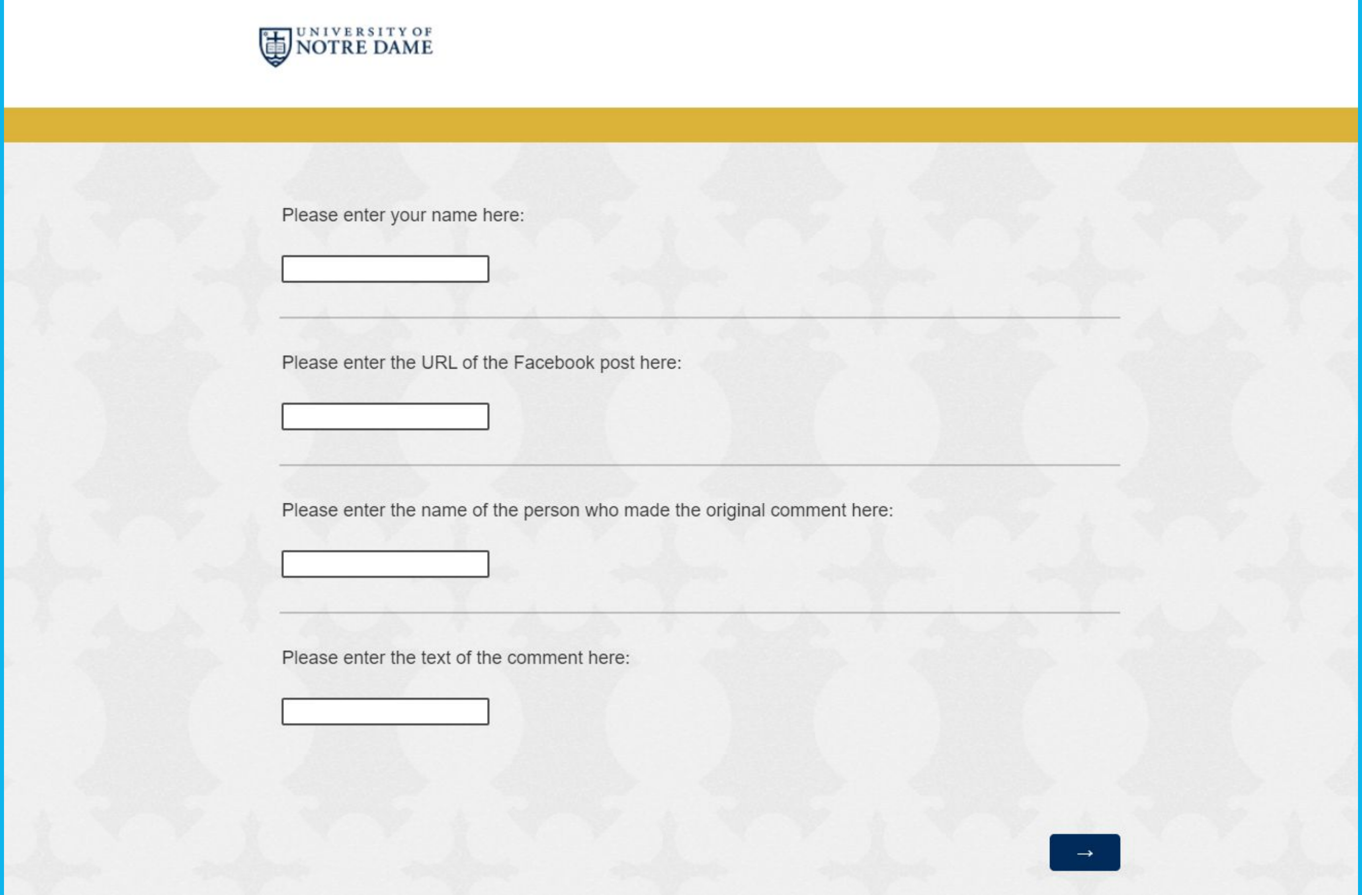
UNIVERSITY OF NOTRE DAME

Please enter your name here:

Please enter the URL of the Facebook post here:

Please enter the name of the person who made the original comment here:

Please enter the text of the comment here:

→

The first part of the randomization tool asks you to enter information about the comment you plan to engage with.

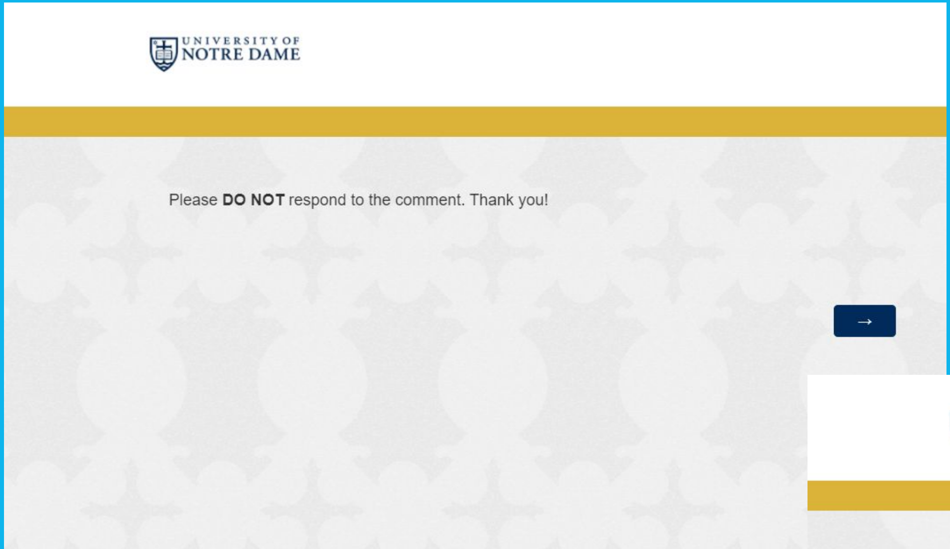

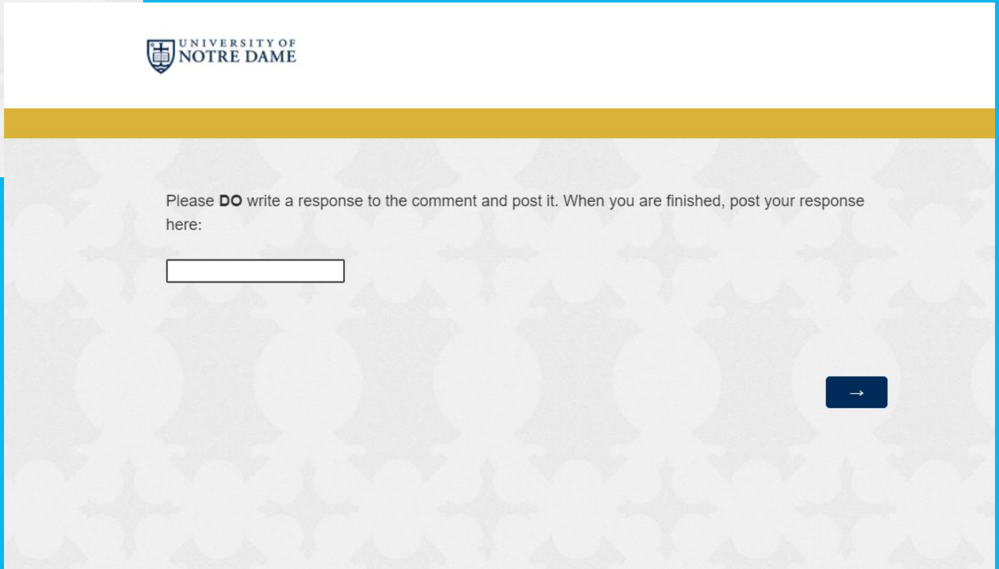

The randomizer will either tell you not to respond to the comment, or to respond and post the text of your response into the form.

Step 4: Write your response (if required)

- Use the behavioral principles we outline in the next section to write your response.
- Refer to the FAQ if needed, or find appropriate studies and citations from other sources (CDC, WHO, academic journals, etc)
- Once you’ve written and posted your response, paste the text into the randomization form
- If the randomizer told you not to respond, skip to Step 5 instead

Step 5: Keep working on your post until you’ve identified all comments requiring a response

- Read through all comments on your post, and repeat steps 3-4 as needed
- You don’t need to respond to every comment - only engage where it seems productive
- Once you’ve either skipped or responded to all comments, delete post URL from the To-Do list

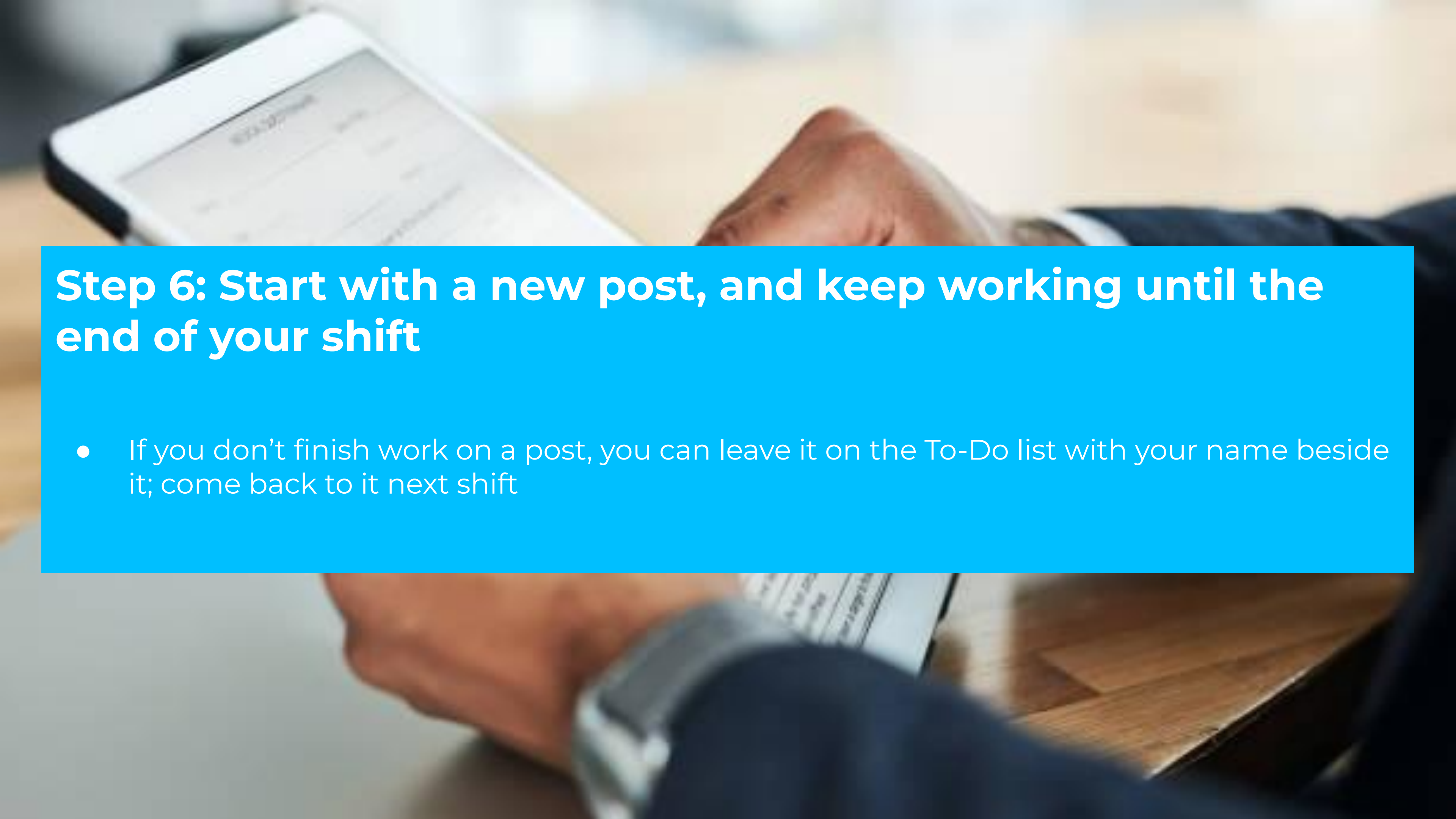

Step 6: Start with a new post, and keep working until the end of your shift

- If you don’t finish work on a post, you can leave it on the To-Do list with your name beside it; come back to it next shift

Building Vaccine Confidence: Behavioral Principles

This section outlines practical, evidence-based principles that can guide your engagement with vaccine-hesitant individuals online

We will show examples of how to respond (and how not to respond) to vaccine-hesitant posts, using real comments from social media

At the end, you will be asked to apply these principles by choosing an appropriate response to several social media posts.

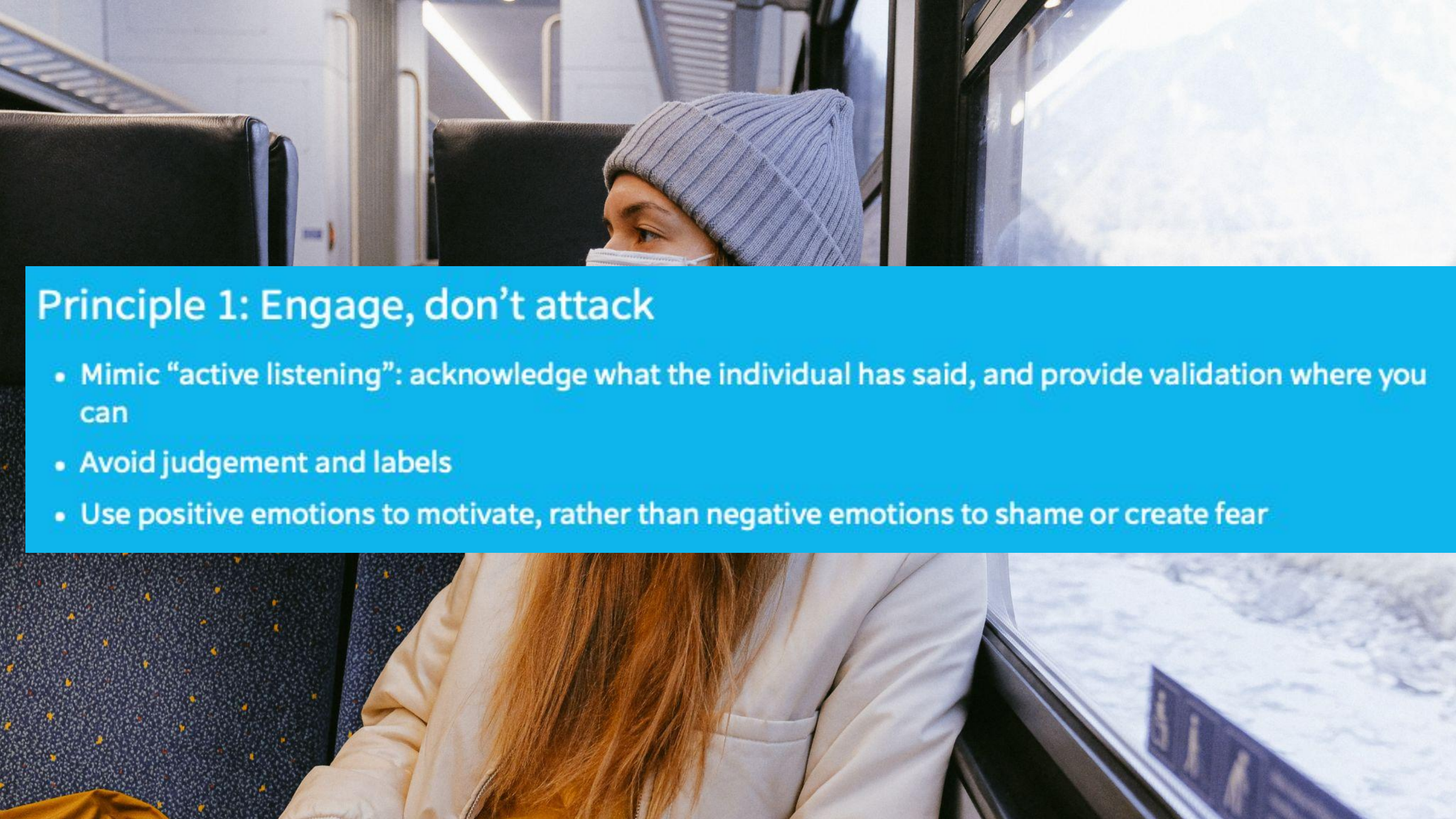

Principle 1: Engage, don't attack

- Mimic "active listening": acknowledge what the individual has said, and provide validation where you can
- Avoid judgement and labels
- Use positive emotions to motivate, rather than negative emotions to shame or create fear

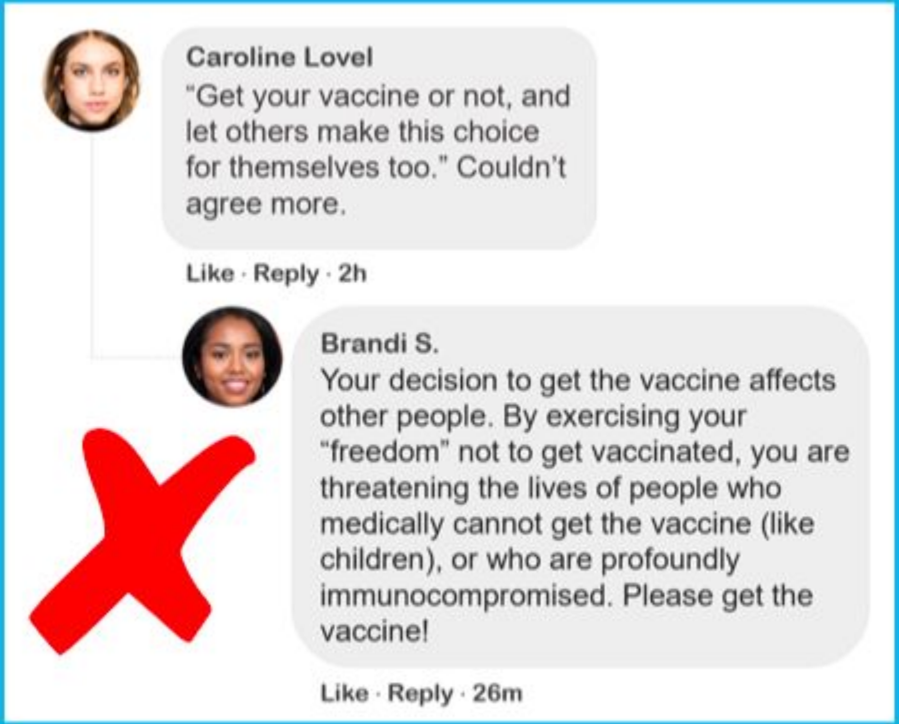

Example 1: Attacking

- Mocking tone (quotation marks around “freedom”)
- Negative framing: your decision threatens others

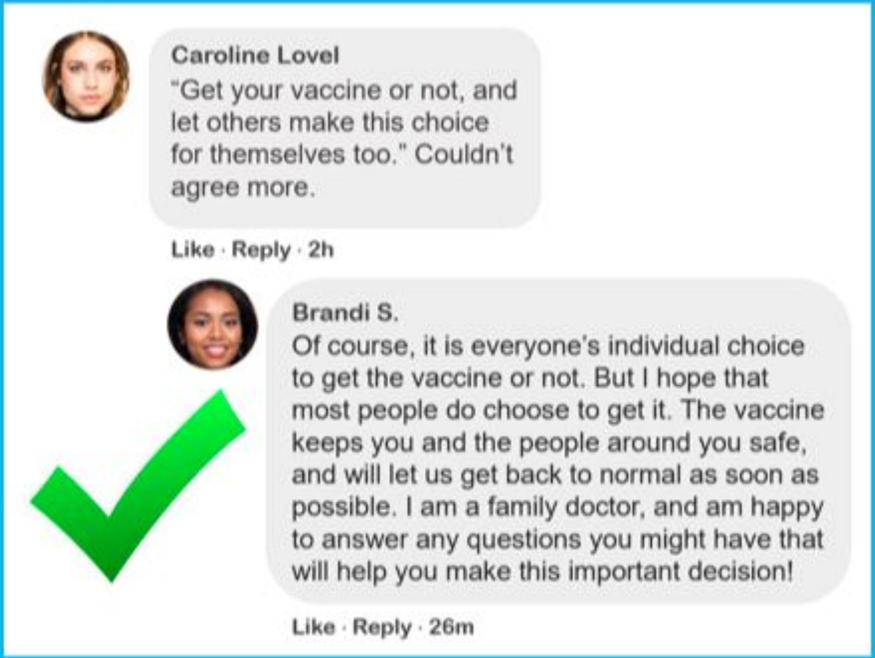

Example 2: Engaging

- Acknowledges the poster's perspective
- Uses positive framing ("the vaccine keeps you safe")
- Invites further discussion

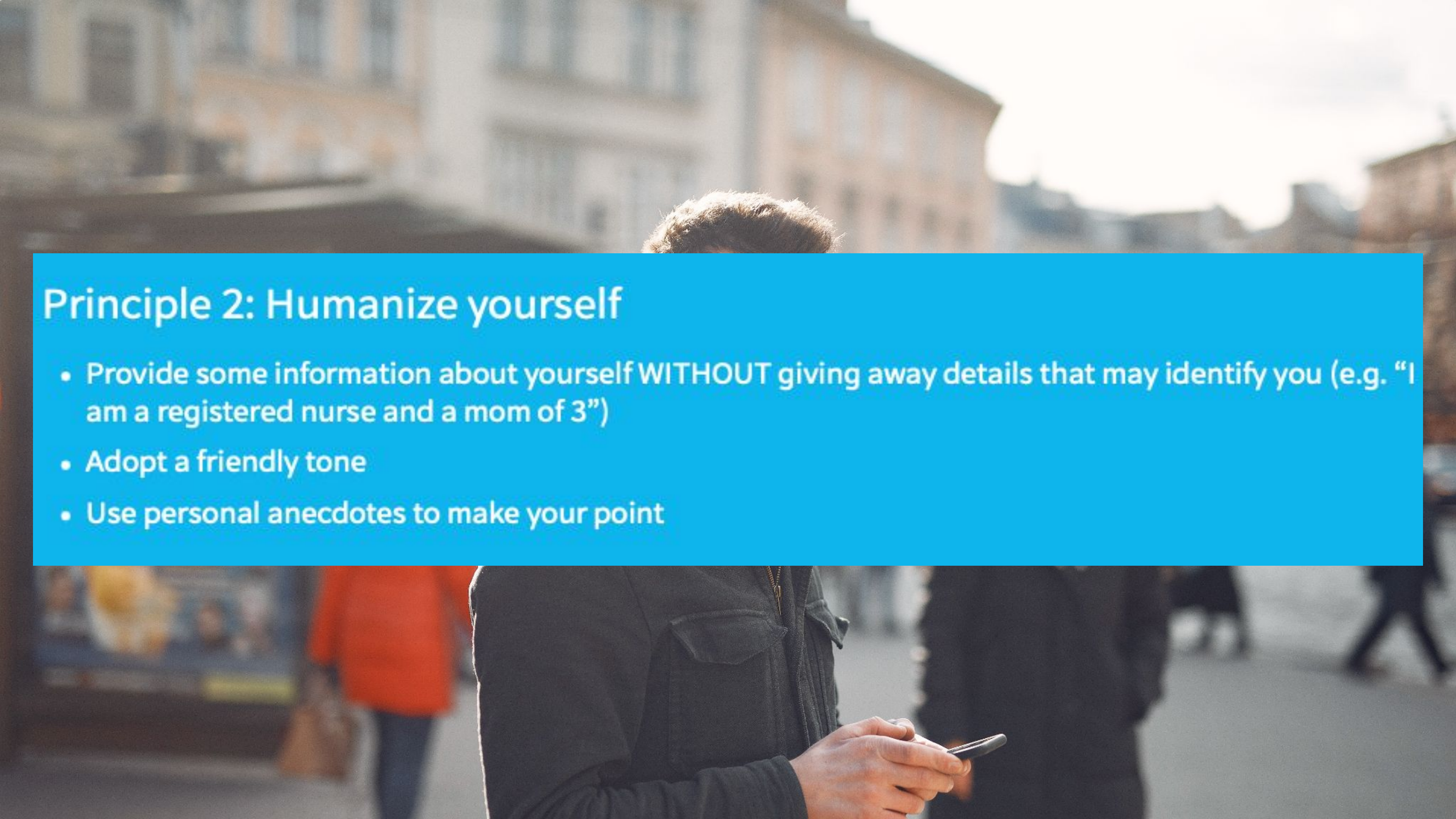

Principle 2: Humanize yourself

- Provide some information about yourself WITHOUT giving away details that may identify you (e.g. “I am a registered nurse and a mom of 3”)
- Adopt a friendly tone
- Use personal anecdotes to make your point

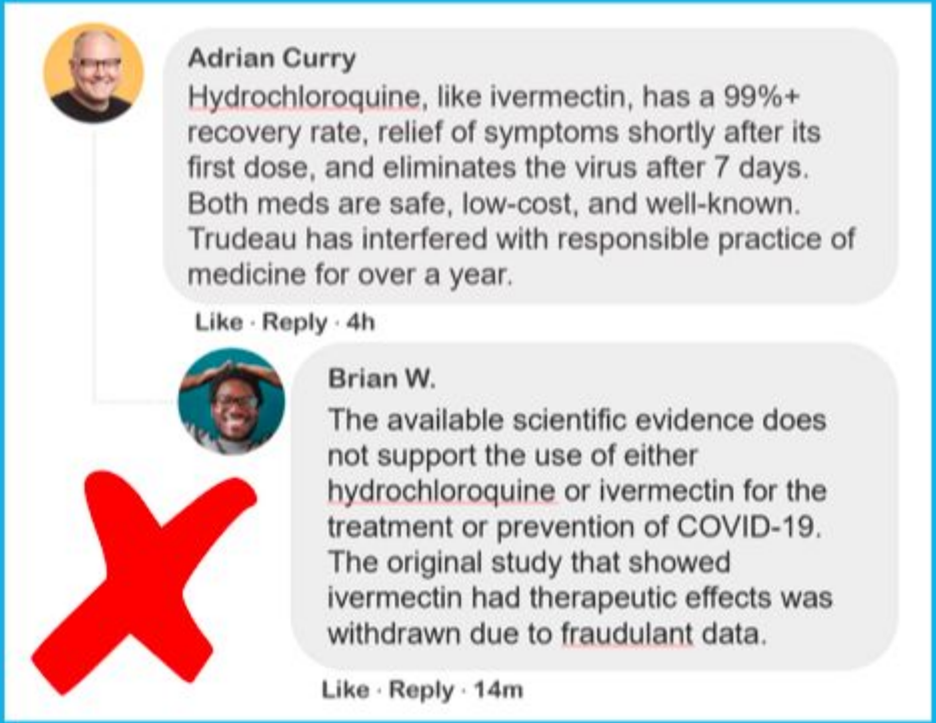

Example 2: Impersonal

- Gives no information about who is responding to the comment
- Dry/scientific tone

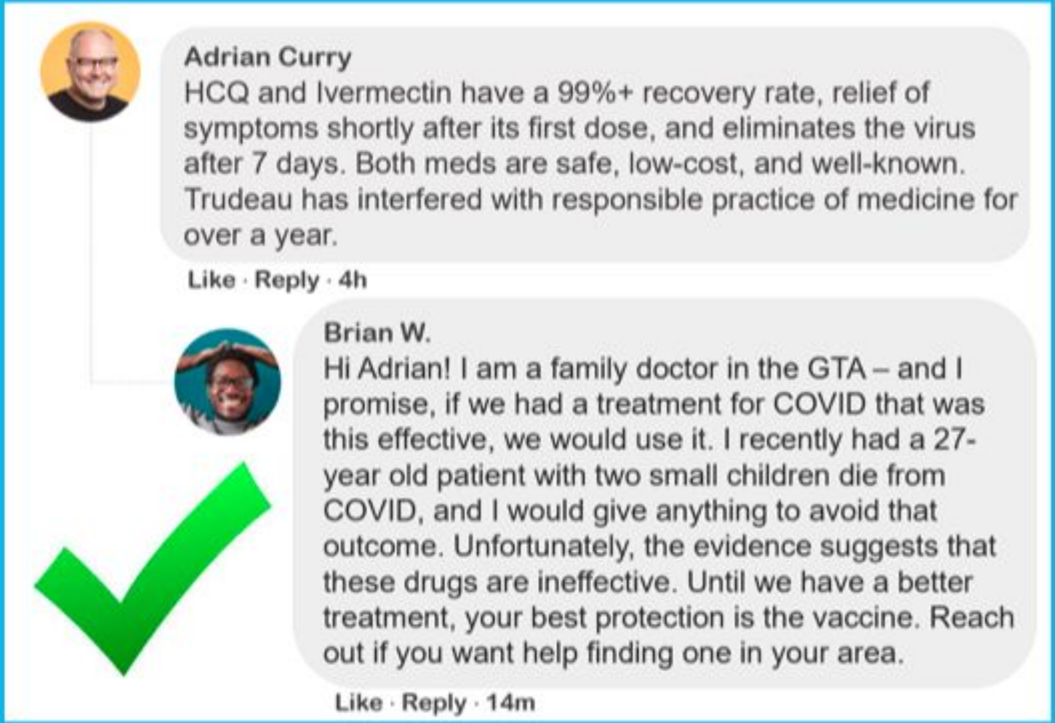

Example 2: Humanized

- Greets the other user in a friendly way, and introduces himself
- Uses a personal anecdote to make his point

Principle 3: Tailor your response

- Address the specific concern that was brought up. Don't expand the scope of the conversation to include other possible concerns about the vaccine.
- When possible, use facts and figures that are specific to the individual's age, gender, city, or other life circumstances.
- If you suspect that cultural background may play a role in the individual's vaccine hesitancy, get a team member who shares that background to respond.

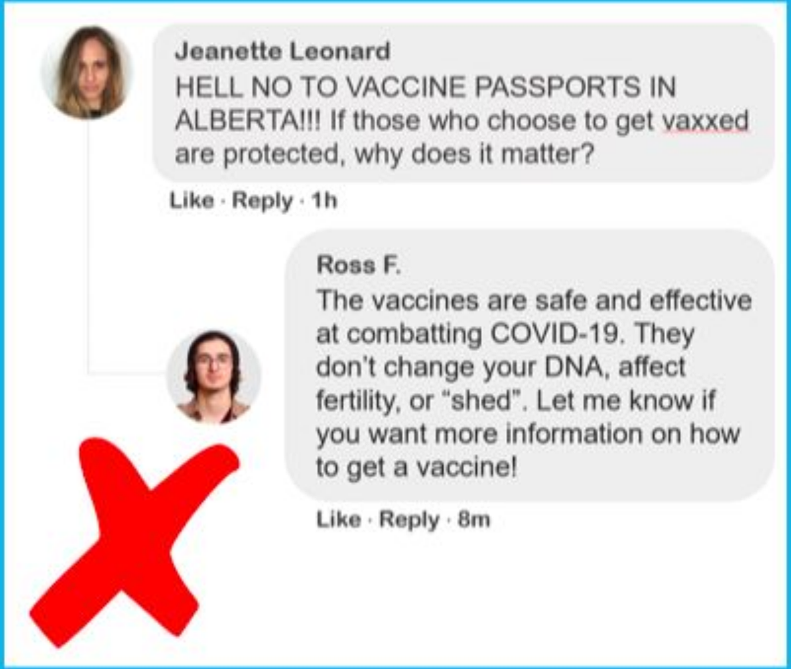

Example 3: Generic

- Doesn't address the original commenter's specific argument
- Mentions concerns about the vaccine not present in the original comment, which can reinforce these concerns through familiarity/repetition effects

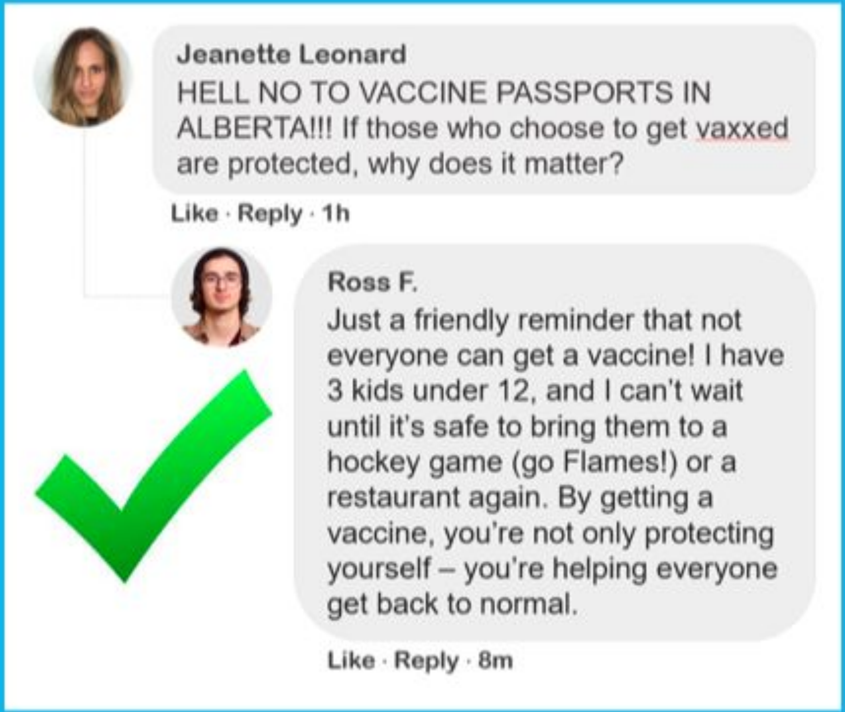

Example 3: Tailored

- Answers original commenter's question/concern
- Uses information in the comment or profile (Alberta/Flames connection) to connect with individual

Principle 4: Be clear and concrete

- Use plain language - avoid technical terms or jargon
- When expressing odds, use concrete framing (e.g. “If 100,000 people got the vaccine, just 2 would have any adverse reactions”, not “There is a 0.002% chance of adverse reactions”.)
- Analogies are a great way to make abstract concepts clearer.

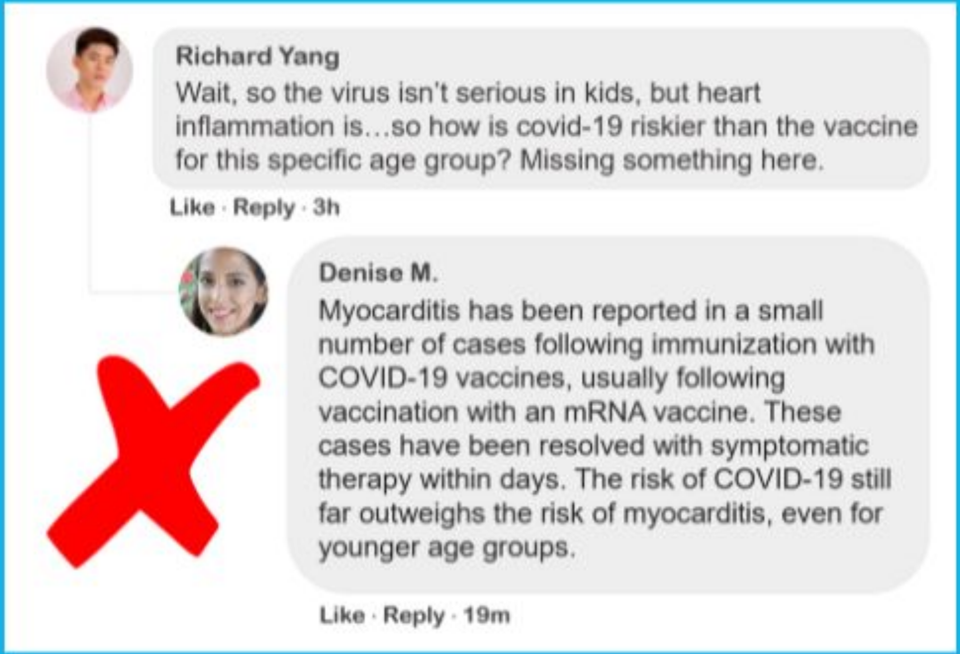

Example 4: Technical

- Dry language
- Uses jargon where simpler words are available (e.g. symptomatic therapy, myocarditis)
- Vague: states that the risk of COVID outweighs the risk of myocarditis, but doesn't show this with facts

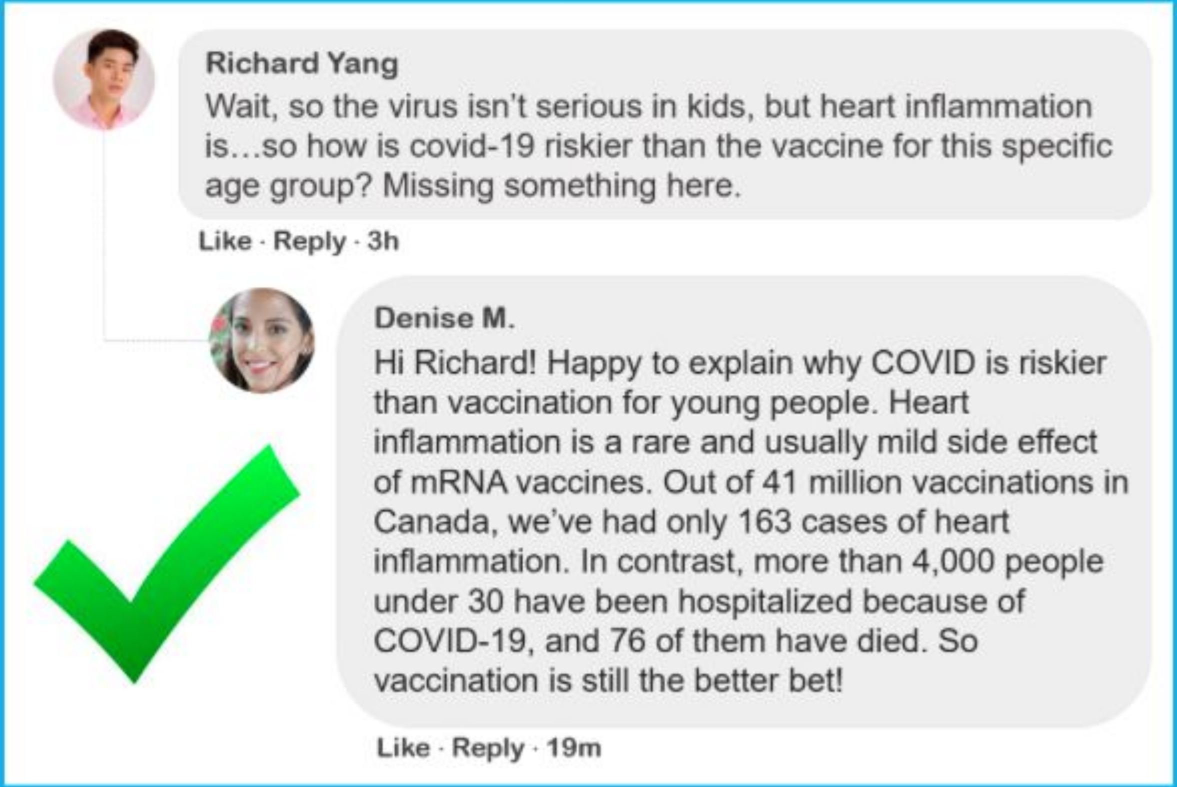

- Uses plain language
- Illustrates the relative risks using easy-to-understand numbers

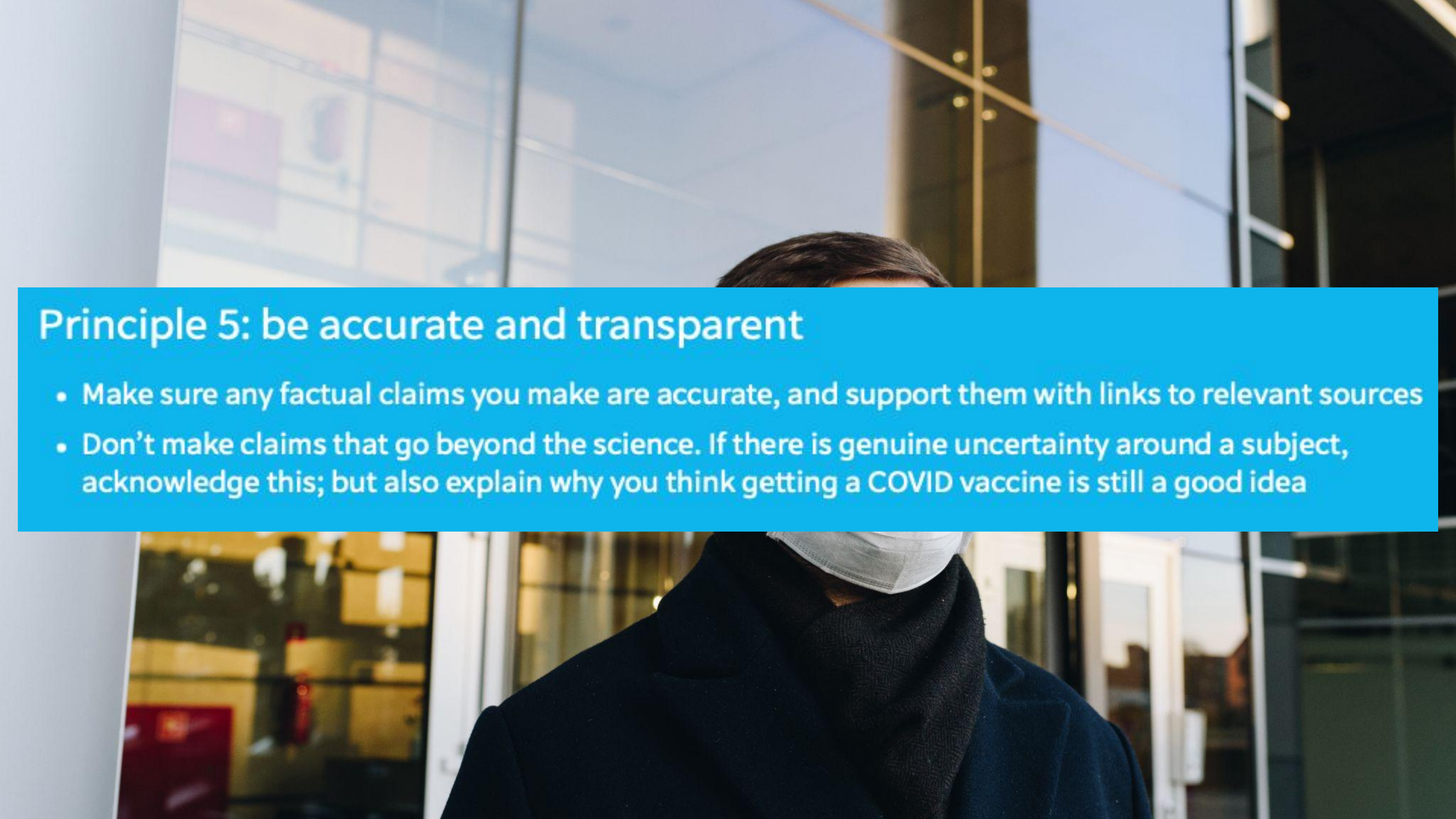

Principle 5: be accurate and transparent

- Make sure any factual claims you make are accurate, and support them with links to relevant sources
- Don’t make claims that go beyond the science. If there is genuine uncertainty around a subject, acknowledge this; but also explain why you think getting a COVID vaccine is still a good idea

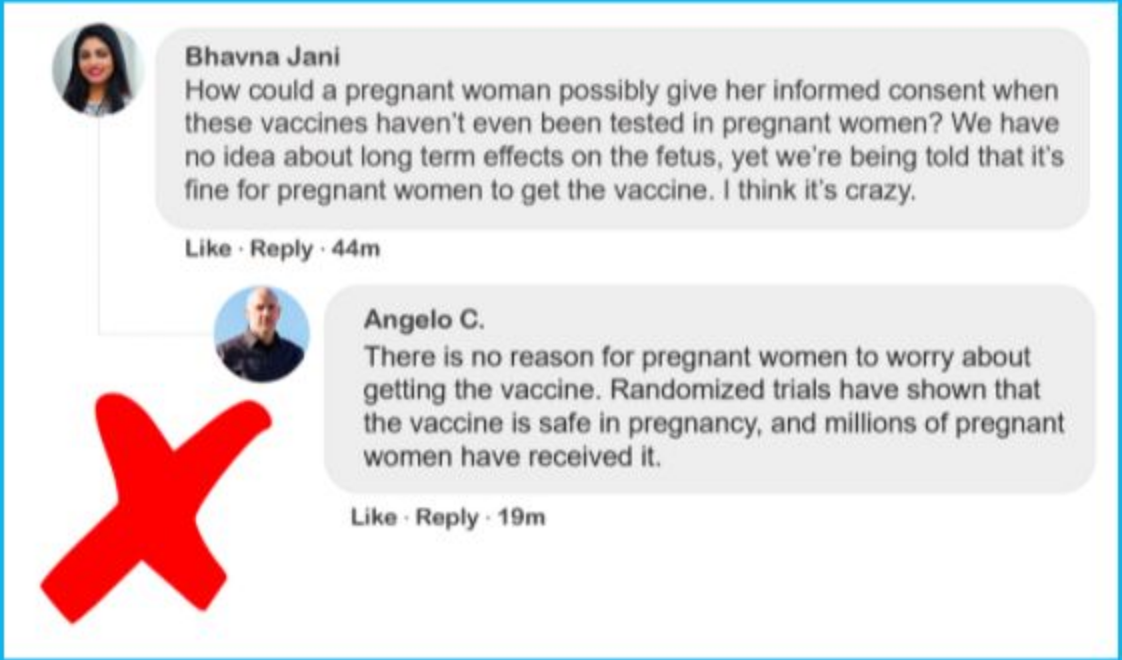

Example 5: Inaccurate

- Dismissive
- Goes beyond the science: randomized trials on pregnant women have not yet been completed
- Does not address the concern about long-term effects

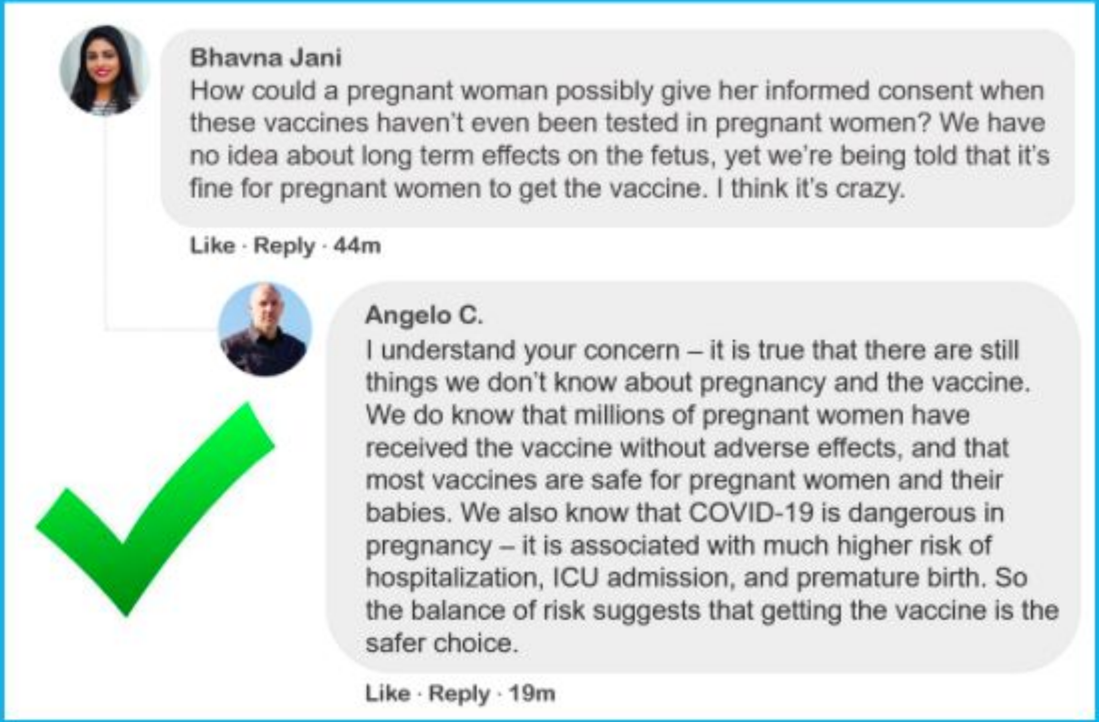

Example 5: Accurate

- **Acknowledges gaps in our understanding of the vaccine's effects**
- **Gives a good reason to get the vaccine, despite some uncertainty**

Some other principles to keep in mind:

Be kind and gracious

It can be very difficult to understand tone in an online conversation. This is why emojis were invented! Kindness goes a long way towards easing tension on social media. A general rule of thumb is to always be overly kind. For example, start your conversations by saying “Hi [firstname]! This is a great question, and one I’m happy to dive into”, or “Hey [name]! I’d like to help clarify this common question, and if anything doesn’t make sense to you, I’m happy to keep chatting.”

Explicitly mention your credentials

In the fight against COVID-19, many people have “become experts” in anything health sciences related. This is why it’s important that you mention your credentials. For example, you might say “I’ve been a family doctor in Alberta for 20 years”, or “I did my Ph.D in microbiology at the University of Toronto” before answering questions.

Give a detailed answer

Your answers should always provide a lot of details. Don't redirect to a link off the bat. You also want to use basic language that anyone can understand. A rule of thumb is writing as if you were explaining something to an 8-year-old. An example of a detailed answer is:

"Hi Sara, my name is Jessica and I am a pediatric infectious disease doctor from Alberta. I think your concern about fertility and the vaccine is an important one to talk about. People who are pregnant have a higher risk of severe illness, hospitalization and ICU admission if they get COVID-19. The vaccines do not cause infertility, in fact during the vaccine clinical trials some women became pregnant and had healthy pregnancies. We have been giving the vaccine for almost 10 months now and many women have gone on to get pregnant in Canada after having received it. If you want to learn more about this topic, check out this really interesting article: (link)"

Offer an open dialogue

There should be an option to keep the conversation going. If you stop responding, it might look like you don't have an answer and we've lost that battle. Always offer to keep answering their questions until they feel like they have enough information to make a decision (this might take some time).

What do you do if someone else enters a conversation?

First, use common sense. We heavily rely on our volunteers to know when to or not to. If the person you originally started talking to is still responding, just focus on them. If they stopped responding, then it means that they want to hear your answer to the new commenter. Whatever you do, never engage with a troll!

What is the preferred method of answering a question?

Always use your words before sending a link. The Facebook algorithm will prioritize conversations over outside links.

What if I don’t know the answer to a question?

We have a long list of FAQs and educational materials available here: https://docs.google.com/spreadsheets/d/1rKf9kAHp4KRcrVxAQXj4gG-zYK2kqKYL4UokcUfXk_Y/edit?usp=sharing

If you still can’t find the answer to a question, contact your volunteer manager. Never provide facts or information that you can’t verify with an external source.

Let’s Practice!

For each of the following social media comments, please identify the response that best employs the principles you’ve learned in this module.

A COMMENT ON AN ARTICLE ABOUT A FAMILY GETTING COVID AFTER BEING VACCINATED

How does the family know that their symptoms were milder bc of the vaccine? I don't know any unvaccinated person that has gotten delta. Js it seems like only the vaccinated peeps are getting the variant.

WHAT'S YOUR ANSWER?

Hi Kimberly! I'm a registered nurse working in Calgary, and I wanted to pass along some numbers about Delta infections among the vaccinated and unvaccinated. A large study in the UK looked at over 44,000 cases of COVID-19, including around 8,000 Delta variant cases, and found that only a tiny fraction occurred among the double vaccinated (1.8%, or around 800 cases). Vaccination also reduced the risk of hospitalization among people who did get COVID. If you want to learn more about the study, here's an article by CTV, which also has a link to the original study: [link]. Happy to chat more if you have any questions!

Hi Kimberly! A recent cohort study in the UK found that the hazard ratio of hospitalizations for delta vs alpha variant was similar for vaccinated and unvaccinated patients, but that vaccinated patients made up just 1.8% of total infections. So vaccination does seem to lower your risk of severe symptoms.

Kimberly - you should read the science before making dangerous statements like this. Of course the COVID vaccine doesn't completely eliminate the risk of getting the disease, but the vast majority of people who get COVID (alpha or delta) are unvaccinated.

The right answer is friendly, provides some information about the person who is responding (which both humanizes the responder, and establishes his or her credentials), and illustrates the risk of the Delta variant using concrete numbers. It also provides lots of details, and a link to the original study. It doesn't have a condescending or hostile tone, and it explains only the relevant statistics from the original study, using clear language.

COMMENT ON AN ARTICLE ABOUT VACCINE MANDATES FOR NURSING HOME STAFF

Not following the science. Vaccinated spread Delta as much as unvaccinated so its a pointless mandate. Way more harm to home residents than good.

WHAT'S YOUR ANSWER?

Hi Brian! I'm an ER doctor in Vancouver. You're probably thinking of this study that showed vaccinated people had a similar viral load as unvaccinated people when they had breakthrough Delta infections [link]. But that is conditional on getting the virus, which is much less likely among the double vaccinated.

Hi Brian! I'm an ER doctor, and I can tell you that almost all of our COVID patients in the hospital are unvaccinated. It's not just here in Vancouver, either - a recent study from the UK looked at 44,000 COVID cases and showed that just 1.8% were among double vaccinated people. Another study showed that vaccinated people are much less likely to transmit the Delta variant even when they get infected. The vaccine really does work to protect vulnerable people (and the rest of us, too!) Happy to chat more about this if you want. Links to the studies I mentioned are here: [links]

Not true - you are much less likely to get COVID if you are vaccinated [link]. If you don't get it, you can't spread it, plain and simple.

The right answer uses a personal anecdote to drive home the author’s point, but also backs up the anecdote with facts. It is friendly, and invites further discussion. It doesn’t repeat commonly misinterpreted facts or misinformation (which can reinforce misconceptions), and it doesn’t condescend to the original commenter.

A COMMENT ON A POST ABOUT 3 CASES OF MYOCARDITIS IN TEENS

What if one of those 3 was your child? Wouldn't 3 be too many? I know plenty of teens that have tested positive for covid and all of them has had mild to no symptoms with no long term damage

WHAT'S YOUR ANSWER?

Hi David! I am a family doctor in Montreal, and hope I can clarify the risks of vaccinations vs. COVID for you. The vaccines don't cause myocarditis in children. The rate of heart inflammation is similar among unvaccinated and vaccinated people. COVID is often very serious even among younger age groups, so getting the vaccine is still safer than remaining unvaccinated.

Hi David. I am a family doctor in Montreal, and I've been informing myself about the relative risks of heart inflammation vs vaccination for my young patients. I hope I can clarify a few things for you. First, the vaccines are much less likely to cause heart inflammation than COVID is. A large study in Israel showed that there were about 3 extra cases of heart inflammation per 100,000 vaccine doses, but 11 extra cases per 100,000 COVID infections. Second, while heart inflammation sounds scary, it is usually mild and resolves with rest and medical treatment. While lots of COVID cases are also mild, there have been lots of serious cases among young people - we've had over 4,000 hospitalizations and 76 deaths of people under 30 in Canada. So even with some risk of heart inflammation, vaccination is still the safest choice.

See this study: https://www.nejm.org/doi/full/10.1056/NEJMoa2110475

The right answer humanizes the writer by providing information about his or her professional background, and explains the relative risk of vaccines v COVID in concrete terms. It is friendly, but the tone is matched to the original commenter's post. It doesn't go beyond the science by claiming that there is no additional risk of myocarditis in children, and it provides detail rather than just a link.

Social Media Personas

The most important first step when getting ready to have a conversation with someone who is vaccine hesitant is to first assess which behaviour they are exhibiting. When we know the behaviour, we can assess how we should respond.

This module will introduce you to four types of people you may encounter online, and explain how to engage with each type.

At the end, you will practice identifying the 4 personas, using real social media comments.

Persona #1: The Troll

- The Troll is out to get into a fight at all costs.
- This person will only get vaccinated when they feel it is “right” for them, or maybe they never will.

In order to recognize a Troll, ask yourself:

- Are they using offensive language?
- Are they violent?
- Are they bullying someone else in the comments?
- Are they pushing misinformation aggressively?

How to respond to a Troll:

- Don’t!
- Engaging with a Troll is a waste of time.
- You don’t want the Troll’s violence to turn on you.

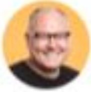

Adrian Curry

NOTHING. It's all a lie.

Revelation 6:2

And I saw, and behold a white horse: and he that sat on him had a "bow"; (G5115 toxon) and a crown (corona) was given unto him: and he went forth conquering and to conquer.

Word history

The Greek word "toxon" means bow or arrow. From this came the Greek toxikon, meaning "a poison in which arrows are dipped". Toxikon was borrowed into Latin as toxicum, which gave rise to the Latin verb intoxicare, "to poison".

Corona is Spanish for crown.

Revelation 18:23

And the light of a candle shall shine no more at all in thee; and the voice of the bridegroom and of the bride shall be heard no more at all in thee; for thy "merchants" were the great men of the earth; for by thy "sorceries" were all nations deceived.

Like · Reply · 4h

Example: The Troll

Persona #2: The Questioner

- Loves to ask a lot of questions of others in a suggestive manner.
- With so much misinformation out there, it can be hard for a questioner to know what is true and not true.
- This person uses logic to help them get closer to the truth.

In order to recognize a Questioner, ask yourself:

- Are they asking questions?
- Is a debate going on?
- Are they pointing out articles that are factually incorrect but they are reasoning logically, without being offensive?

How to respond to a Questioner:

- The Questioner can be convinced, so prepare yourself for a lot of back and forth, and come prepared with data to support your argument.
- Always be kind.
- If you notice they start getting offensive and aggressive, disengage. Sometimes Trolls can hide behind a Questioner persona.

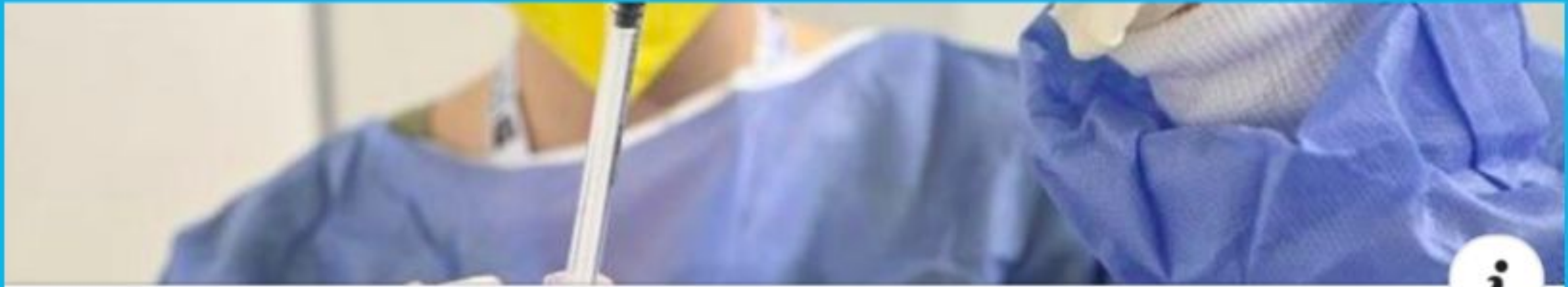

GLOBALNEWS.CA

Myocarditis: What we know about the heart reaction reported after COVID-19 vaccines

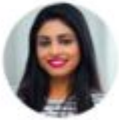

Bhavna Jani
Now the question is, what is the health status of these people? Are they overweight, do they have health problems from the past? Maybe that’s why the numbers are low.

Like · Reply · 44m

Example: The Questioner

Persona #3: The Undecided

- The Undecided feels insecure about the vaccine and all of the misinformation surrounding it.
- They aren’t sure why they should get vaccinated, but don’t have a specific reason not to.
- Trust is at the center of this persona; building a relationship with this person will be important in convincing them to get vaccinated.

In order to recognize an Undecided, ask yourself:

- Are they asking simple, basic questions?
- Are they spreading misinformation, but in a gentle tone (no apparent agenda)?
- Are they relating anecdotes, such as things they heard from a “friend of a friend”?

How to respond to an Undecided:

- Offer a dialogue where you introduce yourself as an expert. See if they want to chat with you, and offer them resources.
- Always be available and build a trusting relationship.
- Don't hand them off to another volunteer; keep the conversation between the two of you.

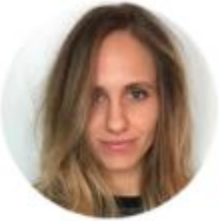

Jeanette Leonard

Any side effects that cause heart problems or blood clots in one person is dangerous. The government makes it sound like just because it is a small number that have had these side effects it is not a big deal. I am sure it is a very big deal to them and their families.

Like · Reply · 1h

Example: The Undecided

Persona #4: The Supporter

- Supporters are vaccinated, and encourage others to get vaccinated
- Engaging with supporters is important, because it encourages them to continue to comment and prevents “buyers’ remorse”

In order to recognize a Supporter, ask yourself:

- Are they leaving comments that explain accurate information in more depth?
- Are they posting vaccine-positive comments on heavily misinformed posts or conversations?

How to respond to a Supporter:

- Agree with them, and thank them for saying the right thing.
- You can also link to data that supports their argument.

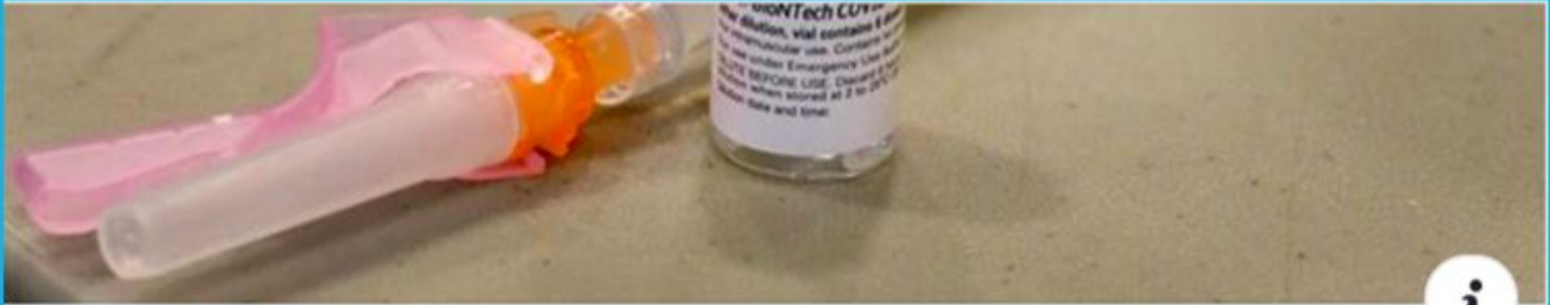

SACHEM.CA

One fully vaccinated person has died with COVID-19 in Hamilton

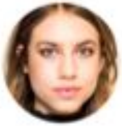

Caroline Lovel
This person had underlying health issues and had not yet completed the required wait period for optimum antibody resistance when COVID was contracted.

Like · Reply · 2h

Example: The Supporter

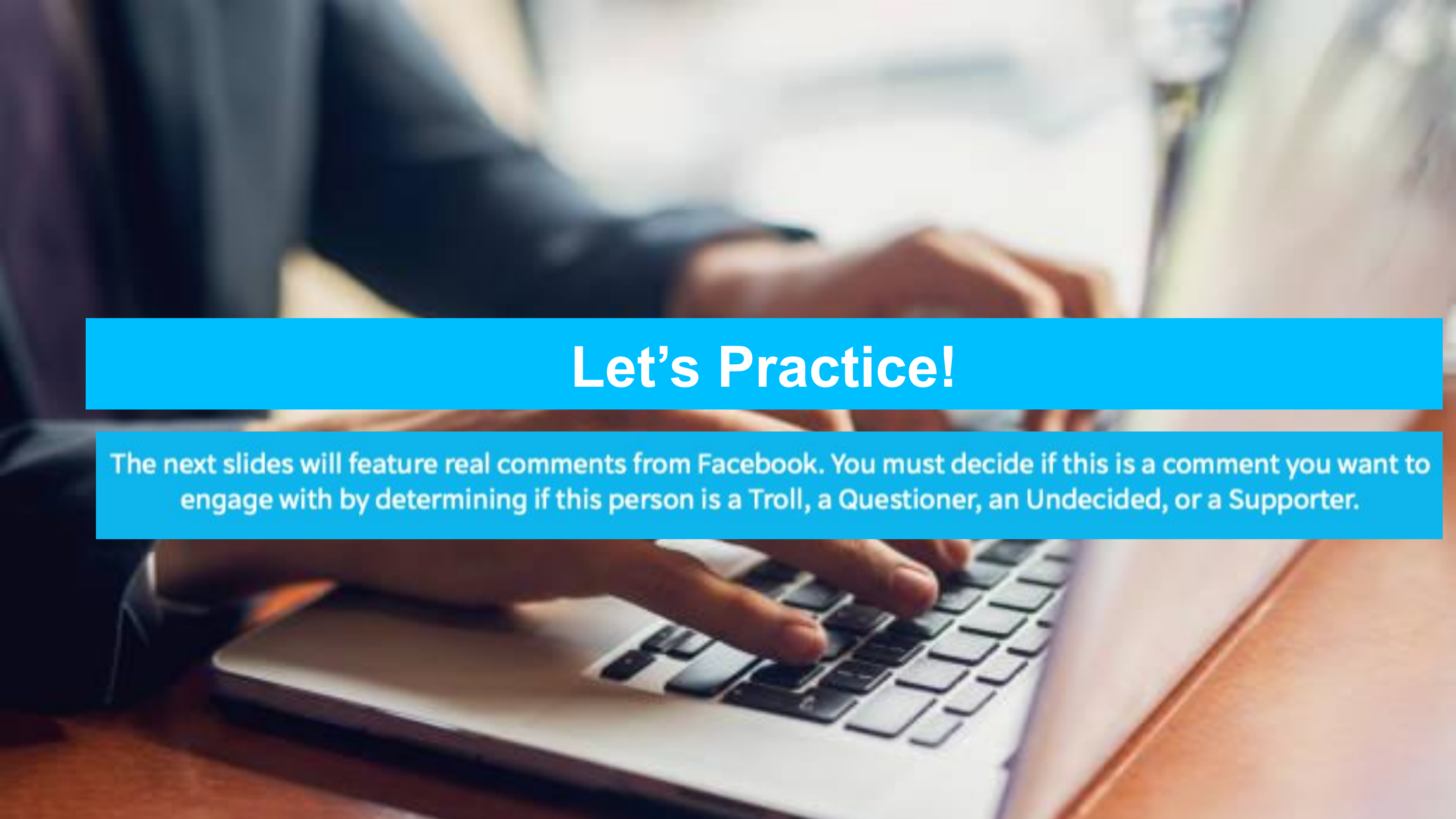

Let’s Practice!

The next slides will feature real comments from Facebook. You must decide if this is a comment you want to engage with by determining if this person is a Troll, a Questioner, an Undecided, or a Supporter.

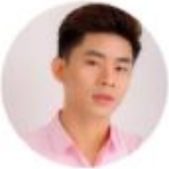

Richard Yang

What about people who cannot get the vaccine for medical reasons? I had the first dose of Pfizer on April 8 and I am still in a flare up of my chronic illness along with other side effects. I can't get answers or help from OPH. My doctor has strongly recommended that I don't get the second dose. I know of 2 other friends with chronic illness who are in a similar situation.

Like · Reply · 3h

Quiz Question #1: Read this comment. On the next slide, indicate which persona you think this person fits best.

Which persona does this comment fit best?

- Troll
- Questioner
- Undecided
- Supporter

You can tell this person is a Questioner, because he asks a valid, answerable question (should people with chronic illnesses get the vaccine?), and has demonstrated a desire to find answers (he asked his doctor and public health).

The best way to engage with this person is to explain that COVID is a bigger risk for many people with chronic illnesses, while encouraging him to follow his doctor's advice for his particular circumstances.

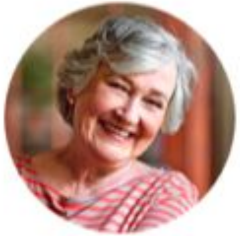

Gill Marshall

This poisonous injections need to be stopped!!

Like · Reply · 1h

Quiz Question #2: Read this comment. On the next slide, indicate which persona you think this person fits best.

Which persona does this comment fit best?

- Troll
- Questioner
- Undecided
- Supporter

You can tell this person is a Troll, because she spreads misinformation (that the vaccine is poison) in a hostile manner, without indicating a specific question or concern.

Never engage with a Troll.

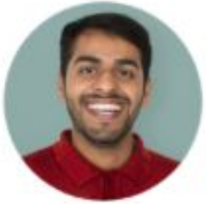

Zaman Sherazi

3 cases of myocarditis, or you name the number! Is that really a game you want to play with your children's life, considering they are such a low risk population to begin with?

Like · Reply · 1h

Quiz Question #3: Read this comment. On the next slide, indicate which persona you think this person fits best.

Which persona does this comment fit best?

- Troll
- Questioner
- Undecided
- Supporter

This person is probably a Questioner, because they are asking a specific question (does the risk of vaccination outweigh the risk of COVID for children?). However, because their question is mildly hostile, it is important to proceed with caution - they could be a Troll hiding behind the persona of a Questioner.

Depending on your comfort level, you could engage with this person by explaining the relative risks of myocarditis vs vaccination. However, if their response is in any way hostile, disengage immediately.

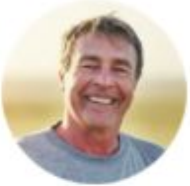

Clayton Seward

"A recent CDC study "suggested" unvaccinated people who've had COVID are more than twice as likely to get re-infected as unvaccinated people."

In other words, they don't know.

Like · Reply · 3h

Quiz Question #4: Read this comment. On the next slide, indicate which persona you think this person fits best.

Which persona does this comment fit best?

- Troll
- Questioner
- Undecided
- Supporter

This person is Undecided: they are spreading misleading information (that the CDC doesn't know about reinfection rates of vaccinated vs unvaccinated people), but in a way that doesn't have an apparent agenda. They are not a Questioner, because they are not asking for information. It would be appropriate to respond to this person by clarifying what the CDC does know about reinfection rates. If this person becomes hostile, however, disengage immediately.

Social Media Security

Keeping yourself safe online

Why do we need security measures?

- Online interactions can bring out the worst in people
- There is a lot of anger surrounding COVID measures and COVID vaccines
- While most people you engage with online are reasonable, there is a chance you might encounter someone dangerous or violent

Using a Social Media Account

- **For your safety, you will be provided with your own Informed Choice Project social media account that you will be in charge of. Each volunteer will have their own account to manage.**

Press Play!

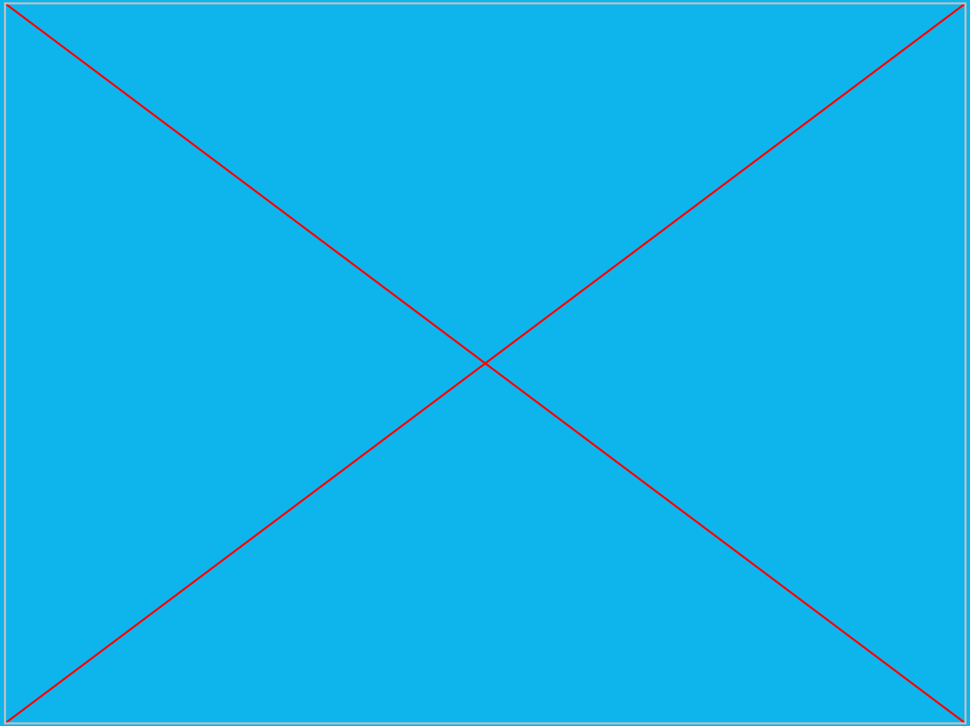

Remember: Avoid Trolls!

- Your best protection is not engaging with people who may become angry or violent
- If an interaction turns even slightly hostile, disengage and flag the user as hostile in the interactions record

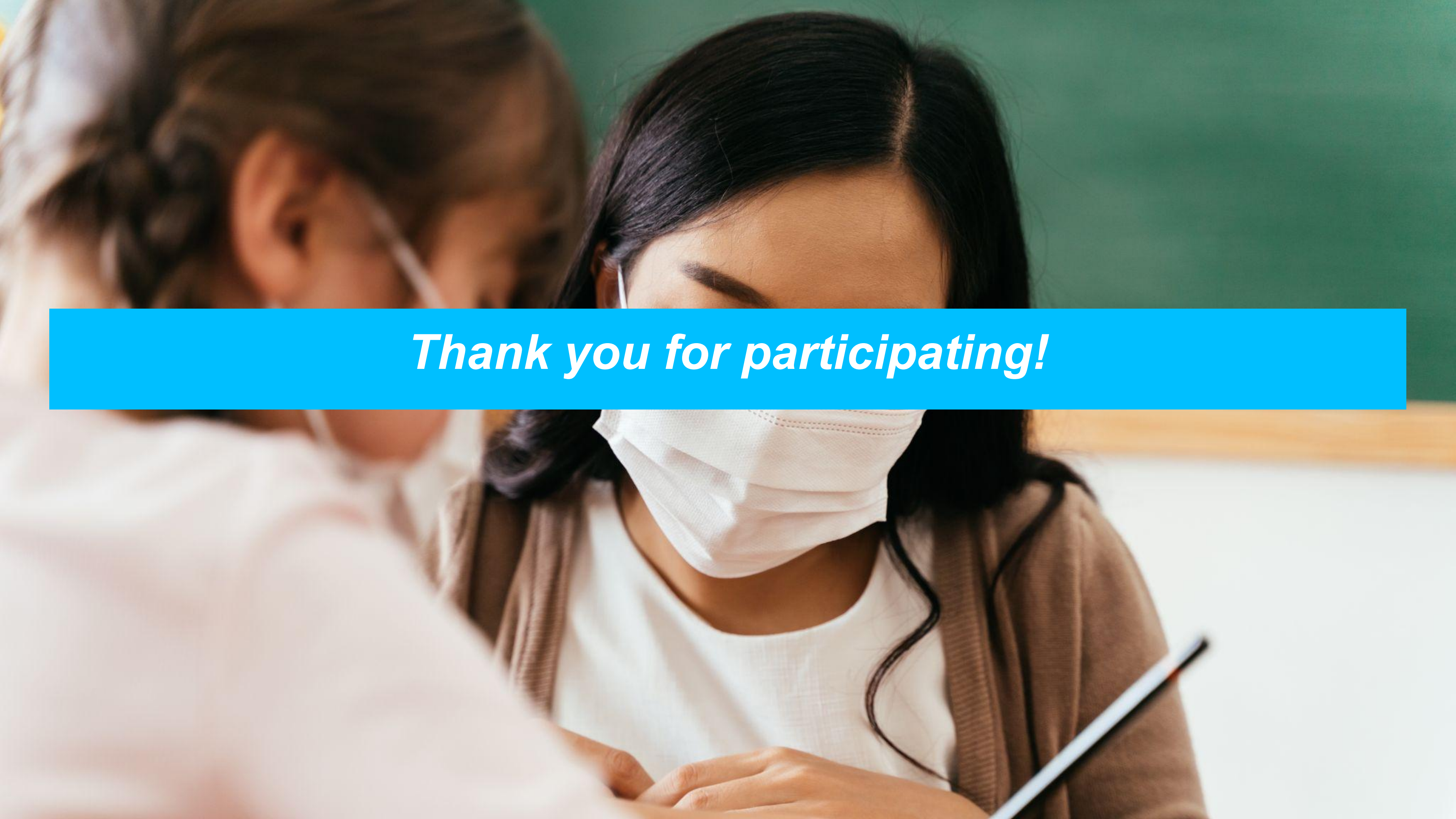
Thank you for participating!

UNIVERSITY OF CALGARY

This study has been approved by the University of Calgary Conjoint Health Research Ethics Board (REB22-0315). Version Date: **December 16, 2022**
