# Supplementary material for: Leveraging Canadian Health Care Worker Volunteers to Address COVID-19 Vaccine Misinformation on Facebook: Qualitative Program Evaluation Study
Source: J Med Internet Res. 2025 Jul 24;27:e65361. doi: 10.2196/65361 (PMC12288766; doi:10.2196/65361)
Supplement: Multimedia Appendix 2 [file jmir-v27-e65361-s002.pdf]

StartDate	EndDate	Status	Progress	Duration (in seco	Finished	RecordedDate	ResponseId	RecipientLastNa	RecipientFirstNa	RecipientEmail	ExternalReferenc	LocationLatitude	LocationLongitud	DistributionChan	UserLanguage
Start Date	End Date	Response Type	Progress	Duration (in seco	Finished	Recorded Date	Response ID	Recipient Last Na	Recipient First Na	Recipient Email	External Data Re	Location Latitude	Location Longitu	Distribution Char	User Language
{"ImportId":"startI	{"ImportId":"endD	{"ImportId":"statu	{"ImportId":"progr	{"ImportId":"durat	{"ImportId":"finish	{"ImportId":"recor	{"ImportId":"_rec	{"ImportId":"recip	{"ImportId":"recip	{"ImportId":"recip	{"ImportId":"exter	{"ImportId":"locat	{"ImportId":"locat	{"ImportId":"distri	{"ImportId":"userLanguage"}
2022-11-03 12:1	2022-11-03 12:1	IP Address	100	10	TRUE	2022-11-03 12:1	R_2BkZfiBH1qDKpZm							anonymous	EN
2022-11-03 12:1	2022-11-03 12:1	IP Address	83	7	FALSE	2022-11-10 11:1	R_2sQNblmZeixHLK4							anonymous	EN
2022-12-16 11:1	2022-12-16 11:1	IP Address	100	128	TRUE	2022-12-16 11:1	R_1LLjZhw5sVjhQ6R							anonymous	EN
2022-12-16 11:1	2022-12-16 11:2	IP Address	83	58	FALSE	2022-12-23 11:2	R_0829WJL4bAxjSsF							anonymous	EN
2023-01-09 10:1	2023-01-09 10:2	IP Address	83	268	FALSE	2023-01-16 10:2	R_1EhssLtTDJusybj							anonymous	EN
2023-01-19 19:1	2023-01-19 19:1	IP Address	100	37	TRUE	2023-01-19 19:1	R_Zk4BhUsR22XQ5Yl							anonymous	EN
2023-01-20 14:1	2023-01-20 14:1	IP Address	100	55	TRUE	2023-01-20 14:1	R_3PHbC1A0UXC5GDk							anonymous	EN
2023-01-20 14:1	2023-01-20 14:1	IP Address	100	15	TRUE	2023-01-20 14:1	R_2QlLROp5KldNHjg							anonymous	EN
2023-01-23 10:1	2023-01-23 10:1	IP Address	100	36	TRUE	2023-01-23 10:1	R_24p9CqcleweO6K6							anonymous	EN
2023-01-23 10:1	2023-01-23 10:1	IP Address	100	3	TRUE	2023-01-23 10:1	R_uq6U929gw5zmGsN							anonymous	EN
2023-01-23 10:1	2023-01-23 10:4	IP Address	100	1827	TRUE	2023-01-23 10:4	R_AtkVp3pg9JNc6l3							anonymous	EN
2023-01-23 10:2	2023-01-23 10:4	IP Address	100	1303	TRUE	2023-01-23 10:4	R_2cvhRNXAorjjzPy							anonymous	EN
2023-01-23 10:4	2023-01-23 10:5	IP Address	100	238	TRUE	2023-01-23 10:5	R_1IvlcZviwezL1Pr							anonymous	EN
2023-01-23 10:5	2023-01-23 11:1	IP Address	100	1242	TRUE	2023-01-23 11:1	R_SB15GtuwADMx8hH							anonymous	EN
2023-01-23 11:1	2023-01-23 11:2	IP Address	100	517	TRUE	2023-01-23 11:2	R_2ClivJAHkr8RgcM							anonymous	EN
2023-01-26 17:0	2023-01-26 17:0	IP Address	100	396	TRUE	2023-01-26 17:0	R_3Pu8q9GhtlSKfnf							anonymous	EN
2023-01-26 17:1	2023-01-26 17:1	IP Address	100	263	TRUE	2023-01-26 17:1	R_1d1aUwfvVQvfIrl							anonymous	EN
2023-01-26 17:1	2023-01-26 17:2	IP Address	100	426	TRUE	2023-01-26 17:2	R_2YFzcUCqdoQJxcT							anonymous	EN
2023-01-26 17:2	2023-01-26 17:2	IP Address	100	164	TRUE	2023-01-26 17:2	R_shi029NN0acqTyV							anonymous	EN
2023-01-26 17:3	2023-01-26 17:4	IP Address	100	56	TRUE	2023-01-26 17:4	R_xbVZCFeD4rUMJUt							anonymous	EN
2023-01-26 17:4	2023-01-26 17:4	IP Address	100	206	TRUE	2023-01-26 17:4	R_UASvrzWEPEMgkJb							anonymous	EN
2023-01-26 17:4	2023-01-26 17:5	IP Address	100	412	TRUE	2023-01-26 17:5	R_1oGTKKNrNUxLkLt							anonymous	EN
2023-01-26 17:5	2023-01-26 17:5	IP Address	100	45	TRUE	2023-01-26 17:5	R_2SCfje2KzUvQS0d							anonymous	EN
2023-01-26 17:5	2023-01-26 18:0	IP Address	100	904	TRUE	2023-01-26 18:0	R_1n3ERV3KItsEE4X							anonymous	EN
2023-01-26 18:0	2023-01-26 18:1	IP Address	100	120	TRUE	2023-01-26 18:1	R_OJszutxM3Q7yGxX							anonymous	EN
2023-01-23 10:4	2023-01-27 14:1	IP Address	100	358147	TRUE	2023-01-27 14:1	R_3nVhEfzrrR1Cnbk							anonymous	EN
2023-01-28 12:2	2023-01-28 12:4	IP Address	100	1367	TRUE	2023-01-28 12:4	R_1pm1DOGM0WwMqTk							anonymous	EN
2023-01-28 12:4	2023-01-28 12:4	IP Address	100	143	TRUE	2023-01-28 12:4	R_CeQpHV1xRXYv5pT							anonymous	EN
2023-01-28 12:4	2023-01-28 12:5	IP Address	100	232	TRUE	2023-01-28 12:5	R_26lSgXNzN9QgKdz							anonymous	EN
2023-01-28 12:5	2023-01-28 13:0	IP Address	100	162	TRUE	2023-01-28 13:0	R_2tgLJkmRlnSBLhb							anonymous	EN
2023-01-28 13:0	2023-01-28 13:0	IP Address	100	80	TRUE	2023-01-28 13:0	R_1KrHEaRdpx8zX8W							anonymous	EN
2023-01-28 13:0	2023-01-28 13:0	IP Address	100	314	TRUE	2023-01-28 13:0	R_277ndZTJF7h3o0N							anonymous	EN
2023-01-28 13:0	2023-01-28 13:1	IP Address	100	374	TRUE	2023-01-28 13:1	R_u9A3aM8vnaBfKsF							anonymous	EN
2023-01-28 13:1	2023-01-28 13:1	IP Address	100	38	TRUE	2023-01-28 13:1	R_1GyxxLFcAaBw8bz							anonymous	EN
2023-01-28 13:1	2023-01-28 13:1	IP Address	100	38	TRUE	2023-01-28 13:1	R_SAC2cZlfygDQdep							anonymous	EN
2023-01-28 13:1	2023-01-28 13:1	IP Address	100	33	TRUE	2023-01-28 13:1	R_1jg1H7aGF6kq2Hf							anonymous	EN
2023-01-28 13:1	2023-01-28 13:2	IP Address	100	338	TRUE	2023-01-28 13:2	R_BFbUt712txLfjeV							anonymous	EN
2023-01-28 13:2	2023-01-28 13:2	IP Address	100	32	TRUE	2023-01-28 13:2	R_1DpRJaZ4NnEN0eo							anonymous	EN
2023-01-28 13:2	2023-01-28 13:3	IP Address	100	371	TRUE	2023-01-28 13:3	R_1QAi5nSGIjY7WNw							anonymous	EN
2023-01-28 13:3	2023-01-28 13:3	IP Address	100	223	TRUE	2023-01-28 13:3	R_1HnlCpiPYTmWP4q							anonymous	EN
2023-01-28 13:3	2023-01-28 13:3	IP Address	100	36	TRUE	2023-01-28 13:3	R_2Yxnk41teiSNW0i							anonymous	EN
2023-01-23 10:1	2023-01-30 10:1	IP Address	100	604542	TRUE	2023-01-30 10:1	R_3HH7I5hf2JTBFFD							anonymous	EN
2023-01-30 2:09	2023-01-30 15:5	IP Address	100	49384	TRUE	2023-01-30 15:5	R_3Def4MQi9u6pH6Z							anonymous	EN
2023-01-30 15:5	2023-01-30 15:5	IP Address	100	92	TRUE	2023-01-30 15:5	R_1JF9owMvG4xLIk7							anonymous	EN
2023-01-30 15:5	2023-01-30 15:5	IP Address	100	32	TRUE	2023-01-30 15:5	R_29g1ijjSakRaHEh							anonymous	EN
2023-01-30 15:5	2023-01-30 15:5	IP Address	100	55	TRUE	2023-01-30 15:5	R_21cDfhhyt5akwHJ							anonymous	EN
2023-01-30 15:5	2023-01-30 16:0	IP Address	100	123	TRUE	2023-01-30 16:0	R_1NxiLD45XiYaAqg							anonymous	EN
2023-01-30 16:0	2023-01-30 16:0	IP Address	100	271	TRUE	2023-01-30 16:0	R_8qNjAacqHsA38B3							anonymous	EN
2023-01-30 16:0	2023-01-30 16:0	IP Address	100	105	TRUE	2023-01-30 16:0	R_1I5EjU0lbds5I2p							anonymous	EN
2023-01-30 16:0	2023-01-30 16:0	IP Address	100	145	TRUE	2023-01-30 16:0	R_0PzyMK9uKWlNDwt							anonymous	EN
2023-01-30 16:0	2023-01-30 16:1	IP Address	100	123	TRUE	2023-01-30 16:1	R_32YjLmDV8MFxUxj							anonymous	EN
2023-01-30 16:1	2023-01-30 16:1	IP Address	100	37	TRUE	2023-01-30 16:1	R_UonhmzVsK7e84pz							anonymous	EN
2023-01-30 16:1	2023-01-30 16:2	IP Address	100	551	TRUE	2023-01-30 16:2	R_1GPa4rqODgFvTCl							anonymous	EN
2023-01-30 16:2	2023-01-30 16:2	IP Address	100	28	TRUE	2023-01-30 16:2	R_2638PxOgsJIAMJ9							anonymous	EN
2023-01-30 16:2	2023-01-30 16:3	IP Address	100	728	TRUE	2023-01-30 16:3	R_2veSnO9UK4U6gqt							anonymous	EN
2023-01-30 16:3	2023-01-30 16:4	IP Address	100	552	TRUE	2023-01-30 16:4	R_uxCzzmnFh6bFP1f							anonymous	EN

2023-01-30 16:4	2023-01-30 16:4	IP Address	100	24	TRUE	2023-01-30 16:4	R_A1cLyDAdLRcVe6Z							anonymous	EN	
2023-01-30 16:4	2023-01-30 16:4	IP Address	100	44	TRUE	2023-01-30 16:4	R_XNVBpVQFKP5k4O5							anonymous	EN	
2023-01-31 6:25	2023-01-31 7:14	IP Address	100	2975	TRUE	2023-01-31 7:14	R_V3gxL88yOPXjCI9							anonymous	EN	
2023-01-31 7:15	2023-01-31 7:23	IP Address	100	487	TRUE	2023-01-31 7:23	R_1rO2FVR098c4fbr							anonymous	EN	
2023-01-31 7:23	2023-01-31 7:24	IP Address	100	46	TRUE	2023-01-31 7:24	R_z71jwhil9ygTnm9							anonymous	EN	
2023-01-31 7:24	2023-01-31 7:33	IP Address	100	534	TRUE	2023-01-31 7:33	R_3PozgiIplCSJEl1							anonymous	EN	
2023-01-31 7:33	2023-01-31 7:40	IP Address	100	395	TRUE	2023-01-31 7:40	R_74A5HFo1beEuJjz							anonymous	EN	
2023-01-31 7:42	2023-01-31 7:42	IP Address	100	32	TRUE	2023-01-31 7:42	R_xrOJPZB2voGzdoR							anonymous	EN	
2023-01-31 7:42	2023-01-31 7:44	IP Address	100	115	TRUE	2023-01-31 7:44	R_3PnnCjGx6EwN2fy							anonymous	EN	
2023-01-31 7:44	2023-01-31 7:45	IP Address	100	75	TRUE	2023-01-31 7:45	R_3KPKu2m6CQE7LNM							anonymous	EN	
2023-01-31 7:45	2023-01-31 7:53	IP Address	100	477	TRUE	2023-01-31 7:53	R_3e9ra4NQefPW4ZY							anonymous	EN	
2023-01-31 7:53	2023-01-31 7:54	IP Address	100	48	TRUE	2023-01-31 7:54	R_30qNNwkGRPw9A9f							anonymous	EN	
2023-01-31 7:54	2023-01-31 7:56	IP Address	100	97	TRUE	2023-01-31 7:56	R_2ZD40zZcLxnFdol							anonymous	EN	
2023-01-31 7:56	2023-01-31 7:59	IP Address	100	166	TRUE	2023-01-31 7:59	R_3n5W1f7Wk3Ck65X							anonymous	EN	
2023-01-31 7:59	2023-01-31 8:05	IP Address	100	388	TRUE	2023-01-31 8:05	R_3CBeg9UAJG3WU9V							anonymous	EN	
2023-01-31 8:05	2023-01-31 8:10	IP Address	100	305	TRUE	2023-01-31 8:10	R_3MmmpWXUvHu30Q0							anonymous	EN	
2023-01-31 8:10	2023-01-31 8:11	IP Address	100	49	TRUE	2023-01-31 8:11	R_2eOn05WliMvJIcj							anonymous	EN	
2023-01-31 8:11	2023-01-31 8:31	IP Address	100	1196	TRUE	2023-01-31 8:31	R_xloQ9WEo1TNRfLr							anonymous	EN	
2023-01-31 8:32	2023-01-31 8:37	IP Address	100	317	TRUE	2023-01-31 8:37	R_1k1eTphtMBPNqEw							anonymous	EN	
2023-01-31 8:37	2023-01-31 8:39	IP Address	100	104	TRUE	2023-01-31 8:39	R_CfXfWgB6b8e4gnL							anonymous	EN	
2023-01-31 8:39	2023-01-31 8:39	IP Address	100	40	TRUE	2023-01-31 8:39	R_2WuJMyVUdCqCqoA							anonymous	EN	
2023-01-31 8:39	2023-01-31 8:40	IP Address	100	38	TRUE	2023-01-31 8:40	R_2yarIB0yEMZuZs2							anonymous	EN	
2023-01-31 8:40	2023-01-31 8:51	IP Address	100	648	TRUE	2023-01-31 8:51	R_3Hw9Kf8KFBYik8H							anonymous	EN	
2023-01-31 8:51	2023-01-31 8:52	IP Address	100	58	TRUE	2023-01-31 8:52	R_2tG943WkfXMuzuR							anonymous	EN	
2023-01-31 8:52	2023-01-31 8:53	IP Address	100	45	TRUE	2023-01-31 8:53	R_3oSfygJlYQLUrlV							anonymous	EN	
2023-01-31 8:53	2023-01-31 8:54	IP Address	100	34	TRUE	2023-01-31 8:54	R_UgQckYU8qNp4VAl							anonymous	EN	
2023-01-31 8:54	2023-01-31 8:58	IP Address	100	252	TRUE	2023-01-31 8:58	R_3kzywswshNs8SwA							anonymous	EN	
2023-01-31 17:1	2023-01-31 17:1	IP Address	100	387	TRUE	2023-01-31 17:1	R_2ZJR9OtWxX4FfN7							anonymous	EN	
2023-01-31 17:1	2023-01-31 17:1	IP Address	100	91	TRUE	2023-01-31 17:1	R_3rrJ2bIwN5XNoKR							anonymous	EN	
2023-01-31 17:1	2023-01-31 17:3	IP Address	100	780	TRUE	2023-01-31 17:3	R_pcre6Jp45fMdS6Z							anonymous	EN	
2023-01-31 17:3	2023-01-31 17:3	IP Address	100	180	TRUE	2023-01-31 17:3	R_3k0rVJSsb7HU4zI							anonymous	EN	
2023-01-31 17:3	2023-01-31 17:3	IP Address	100	177	TRUE	2023-01-31 17:3	R_2CP3GjOJknzzNJY							anonymous	EN	
2023-01-31 17:3	2023-01-31 17:4	IP Address	100	212	TRUE	2023-01-31 17:4	R_2OHbFVoA27iDzA4							anonymous	EN	
2023-01-31 17:4	2023-01-31 17:4	IP Address	100	41	TRUE	2023-01-31 17:4	R_2EAjHwsAZnD9urS							anonymous	EN	
2023-02-01 2:19	2023-02-01 7:07	IP Address	100	17306	TRUE	2023-02-01 7:07	R_2UVGkuovq2GdOIu							anonymous	EN	
2023-02-01 7:07	2023-02-01 7:08	IP Address	100	49	TRUE	2023-02-01 7:08	R_2PBXRDfURWd2nqu							anonymous	EN	
2023-02-01 7:08	2023-02-01 7:10	IP Address	100	94	TRUE	2023-02-01 7:10	R_dbxDYhtkdudGbKN							anonymous	EN	
2023-02-01 7:10	2023-02-01 7:13	IP Address	100	206	TRUE	2023-02-01 7:13	R_30e3x8qpeclNDmc							anonymous	EN	
2023-02-01 7:14	2023-02-01 7:20	IP Address	100	357	TRUE	2023-02-01 7:20	R_vNtxKygARpz2Se5							anonymous	EN	
2023-02-01 7:20	2023-02-01 7:21	IP Address	100	114	TRUE	2023-02-01 7:21	R_2vZeLYotDbpkwya							anonymous	EN	
2023-02-01 7:21	2023-02-01 7:22	IP Address	100	56	TRUE	2023-02-01 7:22	R_2uBWlT1IYV1JLmf							anonymous	EN	
2023-02-01 7:22	2023-02-01 7:37	IP Address	100	883	TRUE	2023-02-01 7:37	R_O2bDmlusqzqhJHH							anonymous	EN	
2023-02-01 7:39	2023-02-01 7:39	IP Address	100	26	TRUE	2023-02-01 7:39	R_12JWHrcP7aNpy8g							anonymous	EN	
2023-02-01 7:41	2023-02-01 7:43	IP Address	100	139	TRUE	2023-02-01 7:43	R_2VwGcKWUbxojibs							anonymous	EN	
2023-02-01 7:43	2023-02-01 7:50	IP Address	100	411	TRUE	2023-02-01 7:50	R_1jrALqTWbL2KIsa							anonymous	EN	
2023-02-01 7:50	2023-02-01 7:51	IP Address	100	59	TRUE	2023-02-01 7:51	R_25FTfF5CZ0YGFfs							anonymous	EN	
2023-02-01 7:51	2023-02-01 7:59	IP Address	100	510	TRUE	2023-02-01 7:59	R_8dBbsj3RRpuvsLD							anonymous	EN	
2023-02-01 8:00	2023-02-01 8:02	IP Address	100	145	TRUE	2023-02-01 8:02	R_XtUG54H4KZBxTep							anonymous	EN	
2023-02-01 8:02	2023-02-01 8:07	IP Address	100	295	TRUE	2023-02-01 8:07	R_1QtKcIPtgKGf29d							anonymous	EN	
2023-02-01 8:07	2023-02-01 8:08	IP Address	100	53	TRUE	2023-02-01 8:08	R_3HCGpH6EmtLr0fC							anonymous	EN	
2023-02-01 8:08	2023-02-01 8:09	IP Address	100	63	TRUE	2023-02-01 8:09	R_2zLOh3GFcNs8auF							anonymous	EN	
2023-02-01 8:09	2023-02-01 8:14	IP Address	100	283	TRUE	2023-02-01 8:14	R_0rhhlE1mFDiboB3							anonymous	EN	
2023-02-01 8:14	2023-02-01 8:15	IP Address	100	64	TRUE	2023-02-01 8:15	R_1hyS6Q2RsGSmTOh							anonymous	EN	
2023-02-01 17:3	2023-02-01 17:3	IP Address	100	242	TRUE	2023-02-01 17:3	R_2Sk9MVwsMd6quOH							anonymous	EN	
2023-02-01 17:3	2023-02-01 17:3	IP Address	100	38	TRUE	2023-02-01 17:3	R_2fv1jC8GdouQDTw							anonymous	EN	
2023-02-01 17:4	2023-02-01 17:4	IP Address	100	129	TRUE	2023-02-01 17:4	R_O8dBOyR3ebmGhLH							anonymous	EN	
2023-02-01 17:4	2023-02-01 17:4	IP Address	100	429	TRUE	2023-02-01 17:4	R_9KSKhjc61mBJqff							anonymous	EN	
2023-02-01 17:4	2023-02-01 18:0	IP Address	100	656	TRUE	2023-02-01 18:0	R_2CCVcnsIU2FIFZO							anonymous	EN	
2023-02-02 16:5	2023-02-02 17:0	IP Address	100	346	TRUE	2023-02-02 17:0	R_3PMJAEAb3HPS1Hz							anonymous	EN	

2023-02-02 17:0	2023-02-02 17:1	IP Address	100	386	TRUE	2023-02-02 17:1	R_2fCeL6MFgqMfrcv							anonymous	EN	
2023-02-02 17:1	2023-02-02 17:1	IP Address	100	196	TRUE	2023-02-02 17:1	R_1nZvClPeB1nHRP7							anonymous	EN	
2023-02-02 17:1	2023-02-02 17:1	IP Address	100	88	TRUE	2023-02-02 17:1	R_3sdeyPJyM3zMjfm							anonymous	EN	
2023-02-02 17:1	2023-02-02 17:2	IP Address	100	565	TRUE	2023-02-02 17:2	R_3p6kB0EXngKsY2s							anonymous	EN	
2023-02-02 17:2	2023-02-02 17:2	IP Address	100	121	TRUE	2023-02-02 17:2	R_21FJ1w8HGQo5jsC							anonymous	EN	
2023-02-02 19:2	2023-02-02 19:2	IP Address	100	106	TRUE	2023-02-02 19:2	R_3nOxZoQAPfPZBCX							anonymous	EN	
2023-02-03 8:17	2023-02-03 8:48	IP Address	100	1884	TRUE	2023-02-03 8:48	R_2zI1UGib9HyN5d1							anonymous	EN	
2023-02-03 8:48	2023-02-03 8:53	IP Address	100	264	TRUE	2023-02-03 8:53	R_3DbdTdSuwt28OFR							anonymous	EN	
2023-02-03 8:53	2023-02-03 8:54	IP Address	100	75	TRUE	2023-02-03 8:54	R_1I0nZNEXzBXpt0e							anonymous	EN	
2023-02-03 8:54	2023-02-03 8:57	IP Address	100	160	TRUE	2023-02-03 8:57	R_3sobGDUSzJIwfha							anonymous	EN	
2023-02-03 8:58	2023-02-03 8:59	IP Address	100	47	TRUE	2023-02-03 8:59	R_114nmcatDNKzpIC							anonymous	EN	
2023-02-03 8:59	2023-02-03 9:40	IP Address	100	2477	TRUE	2023-02-03 9:40	R_5su12vlnXSbXvI5							anonymous	EN	
2023-02-03 9:40	2023-02-03 9:44	IP Address	100	184	TRUE	2023-02-03 9:44	R_3MRSl1bgpGZDSm4							anonymous	EN	
2023-02-03 9:44	2023-02-03 9:54	IP Address	100	600	TRUE	2023-02-03 9:54	R_1DwAY7ekRDFhlHu							anonymous	EN	
2023-02-03 9:54	2023-02-03 9:56	IP Address	100	143	TRUE	2023-02-03 9:56	R_2bIXSJJ2HiclouF							anonymous	EN	
2023-02-03 9:56	2023-02-03 9:59	IP Address	100	169	TRUE	2023-02-03 9:59	R_1Om0u97MjPL91Gk							anonymous	EN	
2023-02-03 9:59	2023-02-03 10:0	IP Address	100	235	TRUE	2023-02-03 10:0	R_sjyaKIsc0RCAzyp							anonymous	EN	
2023-02-03 10:0	2023-02-03 10:0	IP Address	100	155	TRUE	2023-02-03 10:0	R_1UnSJD7eJka0Oyt							anonymous	EN	
2023-02-03 10:0	2023-02-03 10:0	IP Address	100	132	TRUE	2023-02-03 10:0	R_2pQTTr30RBuHYlg							anonymous	EN	
2023-02-03 10:0	2023-02-03 10:1	IP Address	100	217	TRUE	2023-02-03 10:1	R_e359KjGwPbpqNj3							anonymous	EN	
2023-02-03 10:1	2023-02-03 10:1	IP Address	100	228	TRUE	2023-02-03 10:1	R_1IvcOwu3bdstaxW							anonymous	EN	
2023-02-03 10:1	2023-02-03 10:2	IP Address	100	240	TRUE	2023-02-03 10:2	R_3e7YKRdyMApbgi8							anonymous	EN	
2023-02-03 10:2	2023-02-03 10:2	IP Address	100	47	TRUE	2023-02-03 10:2	R_2azZtF3cZWIPuFW							anonymous	EN	
2023-02-03 10:2	2023-02-03 10:2	IP Address	100	77	TRUE	2023-02-03 10:2	R_2S7VkJepnuqvrAW							anonymous	EN	
2023-02-03 10:2	2023-02-03 10:2	IP Address	100	182	TRUE	2023-02-03 10:2	R_12A4Q5b7UIIRM34							anonymous	EN	
2023-02-03 10:2	2023-02-03 10:3	IP Address	100	167	TRUE	2023-02-03 10:3	R_27qbv80BEjn1WYq							anonymous	EN	
2023-02-03 10:3	2023-02-03 10:3	IP Address	100	380	TRUE	2023-02-03 10:3	R_SBlDe18j5cpdV97							anonymous	EN	
2023-02-03 10:3	2023-02-03 10:3	IP Address	100	97	TRUE	2023-02-03 10:3	R_RbpTKs9jb5crnYl							anonymous	EN	
2023-02-03 10:3	2023-02-03 10:4	IP Address	100	240	TRUE	2023-02-03 10:4	R_1P0nUeNTNUBb6iM							anonymous	EN	
2023-02-03 10:4	2023-02-03 10:5	IP Address	100	472	TRUE	2023-02-03 10:5	R_3dK3tysasOVDaXJ							anonymous	EN	
2023-02-03 10:5	2023-02-03 10:5	IP Address	100	90	TRUE	2023-02-03 10:5	R_1EXJyNpClYSy8yM							anonymous	EN	
2023-02-03 10:5	2023-02-03 10:5	IP Address	100	271	TRUE	2023-02-03 10:5	R_snScvKqcDYFTnBn							anonymous	EN	
2023-01-30 10:1	2023-02-03 14:1	IP Address	100	360126	TRUE	2023-02-03 14:1	R_2DYNEDFXsK0GO0R							anonymous	EN	
2023-01-27 14:1	2023-01-27 14:1	IP Address	83	305	FALSE	2023-02-03 14:1	R_2Xj6iN82xYJpjHJ							anonymous	EN	
2023-02-04 10:2	2023-02-04 10:3	IP Address	100	779	TRUE	2023-02-04 10:3	R_1NCC0W7IpOsIUfE							anonymous	EN	
2023-02-04 10:3	2023-02-04 10:4	IP Address	100	401	TRUE	2023-02-04 10:4	R_31ubdQhCQ0BNaeF							anonymous	EN	
2023-02-04 10:4	2023-02-04 10:4	IP Address	100	104	TRUE	2023-02-04 10:4	R_8phxFdXLS6CKkkF							anonymous	EN	
2023-02-04 10:4	2023-02-04 10:5	IP Address	100	605	TRUE	2023-02-04 10:5	R_1gwDu87sEJrYIH3							anonymous	EN	
2023-02-04 10:5	2023-02-04 11:0	IP Address	100	427	TRUE	2023-02-04 11:0	R_1jcwXgsbLSFfdSO							anonymous	EN	
2023-02-04 11:0	2023-02-04 11:0	IP Address	100	189	TRUE	2023-02-04 11:0	R_33y9Nqeskz7sFSq							anonymous	EN	
2023-02-04 11:0	2023-02-04 11:1	IP Address	100	128	TRUE	2023-02-04 11:1	R_vcM15agdG9EbPrz							anonymous	EN	
2023-02-04 11:1	2023-02-04 11:1	IP Address	100	32	TRUE	2023-02-04 11:1	R_1ovTLgTmyv3tTgs							anonymous	EN	
2023-02-04 11:1	2023-02-04 11:1	IP Address	100	329	TRUE	2023-02-04 11:1	R_3R8AAUpppGIg4bk							anonymous	EN	
2023-02-04 11:1	2023-02-04 11:1	IP Address	100	46	TRUE	2023-02-04 11:1	R_3shJZxWut6ZvGah							anonymous	EN	
2023-02-04 11:1	2023-02-04 11:2	IP Address	100	132	TRUE	2023-02-04 11:2	R_OCnlUkCRPm6OLa9							anonymous	EN	
2023-02-04 11:2	2023-02-04 11:2	IP Address	100	329	TRUE	2023-02-04 11:2	R_1gjUHpDo99hbwur							anonymous	EN	
2023-02-04 11:2	2023-02-04 11:3	IP Address	100	478	TRUE	2023-02-04 11:3	R_2pKRlL4lF0zeoKv							anonymous	EN	
2023-02-04 11:3	2023-02-04 11:3	IP Address	100	151	TRUE	2023-02-04 11:3	R_2wFTsyLMHBhjSR6							anonymous	EN	
2023-02-04 11:3	2023-02-04 12:0	IP Address	100	1222	TRUE	2023-02-04 12:0	R_23fnHUQ02sJOLAs							anonymous	EN	
2023-02-04 12:0	2023-02-04 12:0	IP Address	100	239	TRUE	2023-02-04 12:0	R_2ZOygv1OXGpSBeB							anonymous	EN	
2023-02-04 12:0	2023-02-04 12:0	IP Address	100	254	TRUE	2023-02-04 12:0	R_Q4Zdm0838rEGVX3							anonymous	EN	
2023-02-04 12:0	2023-02-04 12:1	IP Address	100	447	TRUE	2023-02-04 12:1	R_2sYJ1tgD9swtjoG							anonymous	EN	
2023-02-04 12:1	2023-02-04 12:2	IP Address	100	309	TRUE	2023-02-04 12:2	R_2sattE1fAvHXB0L							anonymous	EN	
2023-02-04 12:2	2023-02-04 12:2	IP Address	100	117	TRUE	2023-02-04 12:2	R_1kYwulpwwzCEZYN							anonymous	EN	
2023-02-04 12:2	2023-02-04 12:2	IP Address	100	59	TRUE	2023-02-04 12:2	R_vZShEwzPuIhomyt							anonymous	EN	
2023-02-04 12:2	2023-02-04 12:2	IP Address	100	38	TRUE	2023-02-04 12:2	R_1NghTgSQ7JfgekU							anonymous	EN	
2023-02-04 12:2	2023-02-04 12:2	IP Address	100	160	TRUE	2023-02-04 12:2	R_2f3rs6m2hrpK7ji							anonymous	EN	
2023-02-04 12:2	2023-02-04 12:3	IP Address	100	299	TRUE	2023-02-04 12:3	R_1cZzidGcu6Fk8HH							anonymous	EN	
2023-02-04 12:3	2023-02-04 12:3	IP Address	100	174	TRUE	2023-02-04 12:3	R_3szFVsSMMfPOnmu							anonymous	EN	

2023-02-05 17:0	2023-02-05 17:0	IP Address	100	134	TRUE	2023-02-05 17:0	R_29buFiGhXrt5Yt8						anonymous	EN	
2023-02-05 17:0	2023-02-05 17:1	IP Address	100	783	TRUE	2023-02-05 17:1	R_AbrEO0koUS3oDWF						anonymous	EN	
2023-02-05 17:1	2023-02-05 17:2	IP Address	100	130	TRUE	2023-02-05 17:2	R_881lOWEk3nBqwHT						anonymous	EN	
2023-02-05 17:2	2023-02-05 17:2	IP Address	100	84	TRUE	2023-02-05 17:2	R_xgsF85PtIlOm6Bz						anonymous	EN	
2023-02-05 17:2	2023-02-05 17:2	IP Address	100	80	TRUE	2023-02-05 17:2	R_RwRBjNcqBjl2pNv						anonymous	EN	
2023-02-05 17:2	2023-02-05 17:3	IP Address	100	842	TRUE	2023-02-05 17:3	R_1hNiINKELcWTQzs						anonymous	EN	
2023-02-06 12:3	2023-02-06 12:4	IP Address	100	570	TRUE	2023-02-06 12:4	R_1FsL9MbKzIWaKAq						anonymous	EN	
2023-02-06 12:4	2023-02-06 12:4	IP Address	100	140	TRUE	2023-02-06 12:4	R_1o7vnuwtzrvNks5						anonymous	EN	
2023-02-06 12:4	2023-02-06 12:5	IP Address	100	473	TRUE	2023-02-06 12:5	R_yyV7iukoYsAQpJn						anonymous	EN	
2023-02-06 12:5	2023-02-06 13:0	IP Address	100	138	TRUE	2023-02-06 13:0	R_2cnZQOz3ELFozkl						anonymous	EN	
2023-02-06 13:0	2023-02-06 13:0	IP Address	100	63	TRUE	2023-02-06 13:0	R_3NKwnfwPeJdb7Ve						anonymous	EN	
2023-02-06 13:0	2023-02-06 13:0	IP Address	100	470	TRUE	2023-02-06 13:0	R_1pG77CM4vJigQiu						anonymous	EN	
2023-02-06 13:0	2023-02-06 13:1	IP Address	100	500	TRUE	2023-02-06 13:1	R_OxM5Zn7ovzPuOJP						anonymous	EN	
2023-02-06 13:1	2023-02-06 13:1	IP Address	100	79	TRUE	2023-02-06 13:1	R_2YsHXkLE96Mms4R						anonymous	EN	
2023-02-06 13:1	2023-02-06 13:2	IP Address	100	340	TRUE	2023-02-06 13:2	R_1g5WlmPTo8TrZbq						anonymous	EN	
2023-02-06 13:2	2023-02-06 13:2	IP Address	100	84	TRUE	2023-02-06 13:2	R_1FwxfX2CpxRHs71						anonymous	EN	
2023-02-06 13:2	2023-02-06 13:3	IP Address	100	144	TRUE	2023-02-06 13:3	R_21hQsxHglAmwerf						anonymous	EN	
2023-02-06 13:3	2023-02-06 13:3	IP Address	100	96	TRUE	2023-02-06 13:3	R_1gefLPD63PBwZ2I						anonymous	EN	
2023-02-06 13:3	2023-02-06 13:3	IP Address	100	165	TRUE	2023-02-06 13:3	R_2zSZIkcuP88Frax						anonymous	EN	
2023-02-06 13:3	2023-02-06 13:3	IP Address	100	41	TRUE	2023-02-06 13:3	R_PtGmevd3eQAPT21						anonymous	EN	
2023-02-06 13:3	2023-02-06 13:3	IP Address	100	31	TRUE	2023-02-06 13:3	R_2YKiapmNV8UaWw6						anonymous	EN	
2023-02-06 13:3	2023-02-06 14:5	IP Address	100	4603	TRUE	2023-02-06 14:5	R_1ILWtL8y5YFP61B						anonymous	EN	
2023-02-06 14:5	2023-02-06 15:0	IP Address	100	456	TRUE	2023-02-06 15:0	R_2zcPhOWcgdYOALJ						anonymous	EN	
2023-02-06 15:0	2023-02-06 15:0	IP Address	100	196	TRUE	2023-02-06 15:0	R_RUjVaHGuRKK8DUR						anonymous	EN	
2023-02-06 15:0	2023-02-06 15:0	IP Address	100	57	TRUE	2023-02-06 15:0	R_3iDdHpFqd9BnmXr						anonymous	EN	
2023-02-06 15:0	2023-02-06 15:0	IP Address	100	45	TRUE	2023-02-06 15:0	R_3I5Ewg1VRMheir1						anonymous	EN	
2023-02-06 15:0	2023-02-06 15:1	IP Address	100	360	TRUE	2023-02-06 15:1	R_3qq9Y7YNNQ6ftlL						anonymous	EN	
2023-02-06 15:1	2023-02-06 15:3	IP Address	100	919	TRUE	2023-02-06 15:3	R_6XVCnjDClba067T						anonymous	EN	
2023-02-06 15:3	2023-02-06 15:3	IP Address	100	308	TRUE	2023-02-06 15:3	R_RxbzZGSqCE8XFE5						anonymous	EN	
2023-02-06 15:3	2023-02-06 15:3	IP Address	100	97	TRUE	2023-02-06 15:3	R_22speG9GWWdT43W						anonymous	EN	
2023-02-06 15:3	2023-02-06 15:4	IP Address	100	66	TRUE	2023-02-06 15:4	R_3QNIk2KEZrnfcXL						anonymous	EN	
2023-02-06 15:4	2023-02-06 15:4	IP Address	100	231	TRUE	2023-02-06 15:4	R_2z6LdvncT1zBFHX						anonymous	EN	
2023-02-06 15:4	2023-02-06 15:4	IP Address	100	244	TRUE	2023-02-06 15:4	R_2zu55sggalqn37W						anonymous	EN	
2023-02-06 15:4	2023-02-06 15:5	IP Address	100	366	TRUE	2023-02-06 15:5	R_2WxnUHqQQtvZHlX						anonymous	EN	
2023-02-06 15:5	2023-02-06 15:5	IP Address	100	83	TRUE	2023-02-06 15:5	R_2OZssWao2XXVSog						anonymous	EN	
2023-02-06 15:5	2023-02-06 15:5	IP Address	100	31	TRUE	2023-02-06 15:5	R_3Qw1tihSSdCJRjX						anonymous	EN	
2023-02-06 15:5	2023-02-06 16:0	IP Address	100	259	TRUE	2023-02-06 16:0	R_3Djb2GFey68V7pK						anonymous	EN	
2023-02-06 17:0	2023-02-06 17:1	IP Address	100	532	TRUE	2023-02-06 17:1	R_28TgVCq9ISNHILS						anonymous	EN	
2023-02-06 17:1	2023-02-06 17:2	IP Address	100	222	TRUE	2023-02-06 17:2	R_2w7bZXLJ5NKdmQ2						anonymous	EN	
2023-02-06 17:2	2023-02-06 17:2	IP Address	100	191	TRUE	2023-02-06 17:2	R_ywLR2Rrm1JU049z						anonymous	EN	
2023-02-06 17:2	2023-02-06 17:2	IP Address	100	97	TRUE	2023-02-06 17:2	R_NVBmriGYJ5afzBT						anonymous	EN	
2023-02-06 17:2	2023-02-06 17:3	IP Address	100	436	TRUE	2023-02-06 17:3	R_1gIovryNwPvf4Ed						anonymous	EN	
2023-02-06 16:0	2023-02-07 10:0	IP Address	100	65067	TRUE	2023-02-07 10:0	R_0ALre5oP65yqQ49						anonymous	EN	
2023-02-07 10:0	2023-02-07 10:0	IP Address	100	57	TRUE	2023-02-07 10:0	R_3PhtLhlbHE1mqQn						anonymous	EN	
2023-02-07 10:0	2023-02-07 10:0	IP Address	100	64	TRUE	2023-02-07 10:0	R_3KIKhnDTed9vuTT						anonymous	EN	
2023-02-07 10:0	2023-02-07 10:1	IP Address	100	301	TRUE	2023-02-07 10:1	R_1oItoB4H9rORrqD						anonymous	EN	
2023-02-07 10:1	2023-02-07 10:1	IP Address	100	307	TRUE	2023-02-07 10:1	R_1MMyHARfhH76o6G						anonymous	EN	
2023-02-07 10:1	2023-02-07 10:2	IP Address	100	232	TRUE	2023-02-07 10:2	R_2vijsrwaEYqOjUO						anonymous	EN	
2023-02-07 10:2	2023-02-07 10:2	IP Address	100	282	TRUE	2023-02-07 10:2	R_3EnAlNHgjmSjIW2						anonymous	EN	
2023-02-07 10:2	2023-02-07 10:2	IP Address	100	97	TRUE	2023-02-07 10:2	R_2ePesXTCB1IkDis						anonymous	EN	
2023-02-07 10:2	2023-02-07 10:3	IP Address	100	78	TRUE	2023-02-07 10:3	R_3oTOyIjDBapxY2G						anonymous	EN	
2023-02-07 10:3	2023-02-07 10:3	IP Address	100	100	TRUE	2023-02-07 10:3	R_PliktmULSxA40al						anonymous	EN	
2023-02-07 10:3	2023-02-07 10:3	IP Address	100	122	TRUE	2023-02-07 10:3	R_33xwbHuO6oI0JyI						anonymous	EN	
2023-02-07 10:3	2023-02-07 10:3	IP Address	100	104	TRUE	2023-02-07 10:3	R_3JjWstsscd5CEky						anonymous	EN	
2023-02-07 10:3	2023-02-07 10:4	IP Address	100	327	TRUE	2023-02-07 10:4	R_1ghXtDPVfBy40Jo						anonymous	EN	
2023-02-07 10:4	2023-02-07 10:4	IP Address	100	103	TRUE	2023-02-07 10:4	R_bvg91O4GqaEYTfz						anonymous	EN	
2023-02-07 10:4	2023-02-07 10:5	IP Address	100	404	TRUE	2023-02-07 10:5	R_sz13Nml1Hb1gWWt						anonymous	EN	
2023-02-07 10:5	2023-02-07 10:5	IP Address	100	470	TRUE	2023-02-07 10:5	R_W8adwDiGZg2hpy9						anonymous	EN	
2023-02-07 10:5	2023-02-07 11:0	IP Address	100	381	TRUE	2023-02-07 11:0	R_2pXNFSyFOVPqjeG						anonymous	EN	

2023-02-07 11:0	2023-02-07 11:0	IP Address	100	253	TRUE	2023-02-07 11:0	R_2Pjbsg4YlJKHDkj							anonymous	EN	
2023-02-07 11:0	2023-02-07 11:1	IP Address	100	533	TRUE	2023-02-07 11:1	R_1jkdePyznj4Pfrn							anonymous	EN	
2023-02-07 11:1	2023-02-07 11:2	IP Address	100	101	TRUE	2023-02-07 11:2	R_277irf1ykXS1UVq							anonymous	EN	
2023-02-07 11:2	2023-02-07 11:3	IP Address	100	320	TRUE	2023-02-07 11:3	R_6Wlvxv2YKSxPvjP							anonymous	EN	
2023-02-07 11:3	2023-02-07 11:3	IP Address	100	100	TRUE	2023-02-07 11:3	R_2A0ZyEmrmB0yMAN							anonymous	EN	
2023-02-07 11:3	2023-02-07 11:4	IP Address	100	547	TRUE	2023-02-07 11:4	R_eRJ8fNQYDMyMmw9							anonymous	EN	
2023-02-07 11:4	2023-02-07 11:4	IP Address	100	78	TRUE	2023-02-07 11:4	R_OwZOIhRcDs4Rs53							anonymous	EN	
2023-02-07 11:4	2023-02-07 11:4	IP Address	100	47	TRUE	2023-02-07 11:4	R_3nGkZ7xjGwIjjw7							anonymous	EN	
2023-02-07 11:4	2023-02-07 11:4	IP Address	100	35	TRUE	2023-02-07 11:4	R_1QF6rfUvicz7Vow							anonymous	EN	
2023-02-07 11:4	2023-02-07 11:4	IP Address	100	34	TRUE	2023-02-07 11:4	R_3PgIMhaUQ8DyZLq							anonymous	EN	
2023-02-07 11:4	2023-02-07 11:5	IP Address	100	283	TRUE	2023-02-07 11:5	R_2dQjXsjcCS0th1b							anonymous	EN	
2023-02-07 11:5	2023-02-07 11:5	IP Address	100	521	TRUE	2023-02-07 11:5	R_1lczhZ9KWXDrX7f							anonymous	EN	
2023-02-07 12:0	2023-02-07 12:0	IP Address	100	120	TRUE	2023-02-07 12:0	R_O3CBehkUH3SoBgd							anonymous	EN	
2023-01-31 16:1	2023-01-31 16:2	IP Address	83	754	FALSE	2023-02-07 16:2	R_29vpPcxod0CabiO							anonymous	EN	
2023-02-07 17:0	2023-02-07 17:0	IP Address	100	219	TRUE	2023-02-07 17:0	R_0GnMWB4uR07fqIV							anonymous	EN	
2023-02-08 12:3	2023-02-08 14:1	IP Address	100	6067	TRUE	2023-02-08 14:1	R_3MgqGCIUX0WsFpy							anonymous	EN	
2023-02-08 14:1	2023-02-08 14:2	IP Address	100	80	TRUE	2023-02-08 14:2	R_1IdbUtDd6bNgLsk							anonymous	EN	
2023-02-08 14:2	2023-02-08 14:3	IP Address	100	645	TRUE	2023-02-08 14:3	R_ZrDeItqsERoFDyh							anonymous	EN	
2023-02-08 14:3	2023-02-08 14:3	IP Address	100	54	TRUE	2023-02-08 14:3	R_2WOtecRG8fTOMWB							anonymous	EN	
2023-02-08 14:3	2023-02-08 14:3	IP Address	100	47	TRUE	2023-02-08 14:3	R_PGkOlIJ1WcjQ59L							anonymous	EN	
2023-02-08 14:3	2023-02-08 14:3	IP Address	100	287	TRUE	2023-02-08 14:3	R_300omlP0PySpYC8							anonymous	EN	
2023-02-08 14:3	2023-02-08 14:4	IP Address	100	240	TRUE	2023-02-08 14:4	R_3rTn7suiGOOu0NU							anonymous	EN	
2023-02-08 14:4	2023-02-08 14:4	IP Address	100	161	TRUE	2023-02-08 14:4	R_2Tpld6Rhd0kSEJl							anonymous	EN	
2023-02-08 14:4	2023-02-08 14:4	IP Address	100	50	TRUE	2023-02-08 14:4	R_1ertgw9Sc6R55ZX							anonymous	EN	
2023-02-08 16:5	2023-02-08 17:0	IP Address	100	261	TRUE	2023-02-08 17:0	R_2BhyDHCPH4lomT8							anonymous	EN	
2023-02-08 17:0	2023-02-08 17:0	IP Address	100	283	TRUE	2023-02-08 17:0	R_OfnrucFtQwE5zO1							anonymous	EN	
2023-02-08 17:0	2023-02-08 17:1	IP Address	100	419	TRUE	2023-02-08 17:1	R_2SIdqxk5zK71XIi							anonymous	EN	
2023-02-08 17:1	2023-02-08 17:1	IP Address	100	151	TRUE	2023-02-08 17:1	R_aeEaUHhFOzMtsXf							anonymous	EN	
2023-02-08 17:1	2023-02-08 17:2	IP Address	100	275	TRUE	2023-02-08 17:2	R_1OH0hdKFwgaFRwG							anonymous	EN	
2023-02-09 5:22	2023-02-09 6:06	IP Address	100	2631	TRUE	2023-02-09 6:06	R_29rXMkJDWakBoBC							anonymous	EN	
2023-02-09 6:08	2023-02-09 8:03	IP Address	100	6877	TRUE	2023-02-09 8:03	R_3RgZDNG3ixTCBa3							anonymous	EN	
2023-02-09 8:03	2023-02-09 8:10	IP Address	100	408	TRUE	2023-02-09 8:10	R_2Xd0LRQnTtcTwVZ							anonymous	EN	
2023-02-09 15:0	2023-02-09 15:1	IP Address	100	627	TRUE	2023-02-09 15:1	R_2ZKCbwep6hSXJtG							anonymous	EN	
2023-02-09 15:1	2023-02-09 15:2	IP Address	100	262	TRUE	2023-02-09 15:2	R_2rlAxurulcltSDw							anonymous	EN	
2023-02-09 15:2	2023-02-09 15:2	IP Address	100	270	TRUE	2023-02-09 15:2	R_22AqXuRe8hvqA46							anonymous	EN	
2023-02-09 15:2	2023-02-09 15:2	IP Address	100	75	TRUE	2023-02-09 15:2	R_22smlyWAKaMdPDt							anonymous	EN	
2023-02-09 15:2	2023-02-09 15:2	IP Address	100	70	TRUE	2023-02-09 15:2	R_31bWArWo8iF2erx							anonymous	EN	
2023-02-09 15:2	2023-02-09 15:3	IP Address	100	216	TRUE	2023-02-09 15:3	R_s4Z99BFo760MtPP							anonymous	EN	
2023-02-09 15:3	2023-02-09 15:3	IP Address	100	93	TRUE	2023-02-09 15:3	R_3sstWPp32oaqRCh							anonymous	EN	
2023-02-09 15:3	2023-02-09 15:3	IP Address	100	314	TRUE	2023-02-09 15:3	R_3g28faNMklm2x9B							anonymous	EN	
2023-02-09 15:3	2023-02-09 15:4	IP Address	100	103	TRUE	2023-02-09 15:4	R_2bT87iy7YH2fgyx							anonymous	EN	
2023-02-09 15:4	2023-02-09 15:4	IP Address	100	23	TRUE	2023-02-09 15:4	R_9LbdAa8rGHrLsSB							anonymous	EN	
2023-02-09 15:4	2023-02-09 15:4	IP Address	100	36	TRUE	2023-02-09 15:4	R_3QLU42DbjjD2YLK							anonymous	EN	
2023-02-09 15:4	2023-02-09 15:4	IP Address	100	109	TRUE	2023-02-09 15:4	R_3fP2EmF5b8KydQt							anonymous	EN	
2023-02-09 15:4	2023-02-09 15:5	IP Address	100	524	TRUE	2023-02-09 15:5	R_TpT3WgRSsu8kRDr							anonymous	EN	
2023-02-09 15:5	2023-02-09 15:5	IP Address	100	190	TRUE	2023-02-09 15:5	R_CmmHfOBjf0BMqxb							anonymous	EN	
2023-02-09 15:5	2023-02-09 16:0	IP Address	100	176	TRUE	2023-02-09 16:0	R_yrKpHHwsYcgct3P							anonymous	EN	
2023-02-09 16:0	2023-02-09 16:0	IP Address	100	108	TRUE	2023-02-09 16:0	R_3IXQqleNeL7bbJT							anonymous	EN	
2023-02-09 16:0	2023-02-09 16:0	IP Address	100	356	TRUE	2023-02-09 16:0	R_4G6OKTjZZ66hCgN							anonymous	EN	
2023-02-09 16:1	2023-02-09 16:1	IP Address	100	114	TRUE	2023-02-09 16:1	R_232kK6KFBzjAC6i							anonymous	EN	
2023-02-09 16:1	2023-02-09 16:1	IP Address	100	94	TRUE	2023-02-09 16:1	R_1DVjxw7x3gS4A56							anonymous	EN	
2023-02-09 16:1	2023-02-09 16:1	IP Address	100	41	TRUE	2023-02-09 16:1	R_25YHvVK8MnzMKHw							anonymous	EN	
2023-02-09 16:1	2023-02-09 16:1	IP Address	100	208	TRUE	2023-02-09 16:1	R_1QgSN0Fvq3B4Vhn							anonymous	EN	
2023-02-03 14:1	2023-02-03 14:1	IP Address	83	9	FALSE	2023-02-10 14:1	R_3nxbcguScensNSw							anonymous	EN	
2023-02-03 14:1	2023-02-03 14:2	IP Address	83	430	FALSE	2023-02-10 14:2	R_3n8TUxL5JuvCjfq							anonymous	EN	
2023-02-11 8:49	2023-02-11 10:1	IP Address	100	5305	TRUE	2023-02-11 10:1	R_vGhFSVi8RLzoDNT							anonymous	EN	
2023-02-11 10:1	2023-02-11 10:2	IP Address	100	315	TRUE	2023-02-11 10:2	R_10A8267C8Ea33up							anonymous	EN	
2023-02-11 10:2	2023-02-11 10:2	IP Address	100	34	TRUE	2023-02-11 10:2	R_3ixsAl8JtSkzKH2							anonymous	EN	
2023-02-11 10:2	2023-02-11 10:2	IP Address	100	167	TRUE	2023-02-11 10:2	R_3Mh7e2KBe7Pawd9							anonymous	EN	

2023-02-11 10:27	2023-02-11 10:31	IP Address	100	295	TRUE	2023-02-11 10:31	R_2UXygB3X7QNMANz							anonymous	EN	
2023-02-11 10:33	2023-02-11 10:38	IP Address	100	306	TRUE	2023-02-11 10:38	R_pc6RnR7Na7YJdfP							anonymous	EN	
2023-02-11 10:38	2023-02-11 10:42	IP Address	100	233	TRUE	2023-02-11 10:42	R_1MYRE95QKf7DIYY							anonymous	EN	
2023-02-11 10:42	2023-02-11 10:47	IP Address	100	286	TRUE	2023-02-11 10:47	R_21ogWubJwfBMNLW							anonymous	EN	
2023-02-11 10:47	2023-02-11 10:49	IP Address	100	141	TRUE	2023-02-11 10:49	R_OBVilgbAXLZdZ9n							anonymous	EN	
2023-02-11 10:49	2023-02-11 10:54	IP Address	100	277	TRUE	2023-02-11 10:54	R_1q2p4XUyGHbgYs2							anonymous	EN	
2023-02-11 10:54	2023-02-11 11:01	IP Address	100	440	TRUE	2023-02-11 11:01	R_1CHClT7BTDaHhdp							anonymous	EN	
2023-02-11 11:01	2023-02-11 11:02	IP Address	100	38	TRUE	2023-02-11 11:02	R_2uPwaDQeZ0AVFhc							anonymous	EN	
2023-02-11 11:02	2023-02-11 11:04	IP Address	100	91	TRUE	2023-02-11 11:04	R_31sdE0n2rtct79i							anonymous	EN	
2023-02-11 11:04	2023-02-11 11:05	IP Address	100	89	TRUE	2023-02-11 11:05	R_1LXt1WJrzSnFK44							anonymous	EN	
2023-02-11 11:05	2023-02-11 11:08	IP Address	100	197	TRUE	2023-02-11 11:08	R_YXfPpA5DJ7mmKlz							anonymous	EN	
2023-02-11 11:08	2023-02-11 11:09	IP Address	100	44	TRUE	2023-02-11 11:09	R_3PAW3jLNxBR1slb							anonymous	EN	
2023-02-11 11:09	2023-02-11 11:14	IP Address	100	274	TRUE	2023-02-11 11:14	R_39GR0a055WBtJZv							anonymous	EN	
2023-02-11 11:14	2023-02-11 11:15	IP Address	100	43	TRUE	2023-02-11 11:15	R_1EcAcMGN2mF6NmF							anonymous	EN	
2023-02-11 11:15	2023-02-11 11:23	IP Address	100	500	TRUE	2023-02-11 11:23	R_2tnULzMshHlXQLJ							anonymous	EN	
2023-02-11 11:23	2023-02-11 11:27	IP Address	100	249	TRUE	2023-02-11 11:27	R_1OJFjqGDeyalSFF							anonymous	EN	
2023-02-11 11:27	2023-02-11 11:28	IP Address	100	27	TRUE	2023-02-11 11:28	R_O1m1WMBY0uEJyj7							anonymous	EN	
2023-02-11 11:28	2023-02-11 11:29	IP Address	100	108	TRUE	2023-02-11 11:29	R_3hgOIiFUlVxMONU							anonymous	EN	
2023-02-11 11:30	2023-02-11 11:31	IP Address	100	52	TRUE	2023-02-11 11:31	R_2Ensd3Q6PAxKdl1							anonymous	EN	
2023-02-11 11:31	2023-02-11 11:33	IP Address	100	103	TRUE	2023-02-11 11:33	R_wUedAvHthzWKNxL							anonymous	EN	
2023-02-11 11:33	2023-02-11 11:34	IP Address	100	88	TRUE	2023-02-11 11:34	R_2cvEnKpZbq7Vf6F							anonymous	EN	
2023-02-11 11:34	2023-02-11 11:35	IP Address	100	52	TRUE	2023-02-11 11:35	R_3m2hqfNpuZ24064							anonymous	EN	
2023-02-11 11:35	2023-02-11 11:37	IP Address	100	122	TRUE	2023-02-11 11:37	R_C4zhu7mAkRnVT6F							anonymous	EN	
2023-02-11 11:37	2023-02-11 11:40	IP Address	100	185	TRUE	2023-02-11 11:40	R_2rYZwgYwRISZdn4							anonymous	EN	
2023-02-11 11:40	2023-02-11 11:43	IP Address	100	123	TRUE	2023-02-11 11:43	R_2q8a4tFKAmEWAwB							anonymous	EN	
2023-02-11 11:43	2023-02-11 11:46	IP Address	100	228	TRUE	2023-02-11 11:46	R_2zwELiCLwMdHB1U							anonymous	EN	
2023-02-11 11:46	2023-02-11 11:50	IP Address	100	227	TRUE	2023-02-11 11:50	R_3MDQNU2CdVLeluR							anonymous	EN	
2023-02-11 11:50	2023-02-11 11:53	IP Address	100	196	TRUE	2023-02-11 11:53	R_2v7VrR1yzIrKjJC							anonymous	EN	
2023-02-11 11:54	2023-02-11 11:54	IP Address	100	39	TRUE	2023-02-11 11:54	R_1IfTLsDyhQKLMLz							anonymous	EN	
2023-02-11 11:54	2023-02-11 11:58	IP Address	100	217	TRUE	2023-02-11 11:58	R_30vPAso3aV4dhVv							anonymous	EN	
2023-02-11 11:58	2023-02-11 11:59	IP Address	100	43	TRUE	2023-02-11 11:59	R_2tbc2z9y6ZLTUH3							anonymous	EN	
2023-02-11 11:59	2023-02-11 12:04	IP Address	100	325	TRUE	2023-02-11 12:04	R_3p9FYP9v04ufx45							anonymous	EN	
2023-02-11 12:04	2023-02-11 12:05	IP Address	100	33	TRUE	2023-02-11 12:05	R_30q69CE7dm0mCqw							anonymous	EN	
2023-02-11 12:05	2023-02-11 12:05	IP Address	100	34	TRUE	2023-02-11 12:05	R_PNysnbjXA0obz9v							anonymous	EN	
2023-02-11 12:05	2023-02-11 12:07	IP Address	100	83	TRUE	2023-02-11 12:07	R_db6mQBhnlDgzgmB							anonymous	EN	
2023-02-11 12:07	2023-02-11 12:07	IP Address	100	30	TRUE	2023-02-11 12:07	R_2BzhAAmWGSm7gOg							anonymous	EN	
2023-02-11 12:07	2023-02-11 12:08	IP Address	100	42	TRUE	2023-02-11 12:08	R_r3i1sCN6ruXqvtv							anonymous	EN	
2023-02-11 12:08	2023-02-11 12:09	IP Address	100	84	TRUE	2023-02-11 12:09	R_1hGMGwLUECqtKKS							anonymous	EN	
2023-02-11 12:09	2023-02-11 12:15	IP Address	100	338	TRUE	2023-02-11 12:15	R_WkTdvgsnEKaUmiJ							anonymous	EN	
2023-02-11 12:15	2023-02-11 12:23	IP Address	100	446	TRUE	2023-02-11 12:23	R_2pXJuToHEAxSFxV							anonymous	EN	
2023-02-11 12:23	2023-02-11 12:26	IP Address	100	180	TRUE	2023-02-11 12:26	R_rj16abO0kk9yUDL							anonymous	EN	
2023-02-11 12:26	2023-02-11 12:28	IP Address	100	120	TRUE	2023-02-11 12:28	R_SCMeH3aKoZRqv4J							anonymous	EN	
2023-02-11 12:28	2023-02-11 12:29	IP Address	100	101	TRUE	2023-02-11 12:29	R_3rNaDQXvMnamJRW							anonymous	EN	
2023-02-11 12:30	2023-02-11 12:36	IP Address	100	362	TRUE	2023-02-11 12:36	R_1Fl9nCiPMT5qOzl							anonymous	EN	
2023-02-12 3:31:	2023-02-12 3:37:	IP Address	100	406	TRUE	2023-02-12 3:37:	R_0VUTgHrrdox2NsR							anonymous	EN	
2023-02-12 3:37:	2023-02-12 3:41:	IP Address	100	239	TRUE	2023-02-12 3:41:	R_eKEj2jEKPtixZuh							anonymous	EN	
2023-02-12 3:43:	2023-02-12 3:45:	IP Address	100	134	TRUE	2023-02-12 3:45:	R_28ZI8nVjZcgmnIQ							anonymous	EN	
2023-02-12 3:45:	2023-02-12 3:46:	IP Address	100	36	TRUE	2023-02-12 3:46:	R_785LjAKTKzGjrih							anonymous	EN	
2023-02-12 3:46:	2023-02-12 3:47:	IP Address	100	61	TRUE	2023-02-12 3:47:	R_20YhtmlXqSaxXwi							anonymous	EN	
2023-02-12 3:47:	2023-02-12 3:52:	IP Address	100	322	TRUE	2023-02-12 3:52:	R_BzV64ZHswtX60Uh							anonymous	EN	
2023-02-12 3:52:	2023-02-12 3:53:	IP Address	100	35	TRUE	2023-02-12 3:53:	R_31tio27wtl2S4ka							anonymous	EN	
2023-02-12 3:56:	2023-02-12 4:00:	IP Address	100	217	TRUE	2023-02-12 4:00:	R_2zGSBBMt2me79np							anonymous	EN	
2023-02-12 4:00:	2023-02-12 4:02:	IP Address	100	116	TRUE	2023-02-12 4:02:	R_33dIrPIpv7zKjap							anonymous	EN	
2023-02-12 4:02:	2023-02-12 4:02:	IP Address	100	35	TRUE	2023-02-12 4:02:	R_BPbqm1el3BLFIRf							anonymous	EN	
2023-02-12 4:02:	2023-02-12 4:04:	IP Address	100	127	TRUE	2023-02-12 4:04:	R_3qrktnqKCdgvbqC							anonymous	EN	
2023-02-12 4:04:	2023-02-12 4:07:	IP Address	100	158	TRUE	2023-02-12 4:07:	R_ALFhqOVJxge7bsB							anonymous	EN	
2023-02-12 4:07:	2023-02-12 4:12:	IP Address	100	313	TRUE	2023-02-12 4:12:	R_8puSYmgENnkNTiN							anonymous	EN	
2023-02-12 4:12:	2023-02-12 4:17:	IP Address	100	291	TRUE	2023-02-12 4:17:	R_1NrWmIvxINgkzKd							anonymous	EN	
2023-02-12 4:17:	2023-02-12 4:18:	IP Address	100	32	TRUE	2023-02-12 4:18:	R_2f6E20mjdAdEhwl							anonymous	EN	

2023-02-12 4:18	2023-02-12 4:19	IP Address	100	79	TRUE	2023-02-12 4:19	R_22S9tMdUel01RVk						anonymous	EN	
2023-02-12 4:19	2023-02-12 4:20	IP Address	100	47	TRUE	2023-02-12 4:20	R_1gUu58p86aTWalt						anonymous	EN	
2023-02-12 4:20	2023-02-12 4:22	IP Address	100	135	TRUE	2023-02-12 4:22	R_3qQhQ2cnKk8h6a6						anonymous	EN	
2023-02-12 4:22	2023-02-12 4:26	IP Address	100	185	TRUE	2023-02-12 4:26	R_2EsC9jGbTLhlDxQ						anonymous	EN	
2023-02-12 4:26	2023-02-12 4:32	IP Address	100	400	TRUE	2023-02-12 4:32	R_3P5mBfQphwVLTr6						anonymous	EN	
2023-02-12 4:32	2023-02-12 4:36	IP Address	100	210	TRUE	2023-02-12 4:36	R_3koBq2VjFbOwNXg						anonymous	EN	
2023-02-12 4:36	2023-02-12 4:39	IP Address	100	178	TRUE	2023-02-12 4:39	R_2wn3R2XuAgDKmw2						anonymous	EN	
2023-02-12 4:39	2023-02-12 4:47	IP Address	100	466	TRUE	2023-02-12 4:47	R_3PFTVsagPTwrFjX						anonymous	EN	
2023-02-12 4:47	2023-02-12 4:49	IP Address	100	92	TRUE	2023-02-12 4:49	R_3fkk8crnIM4NLlW						anonymous	EN	
2023-02-12 4:49	2023-02-12 4:50	IP Address	100	89	TRUE	2023-02-12 4:50	R_sLjHFaYr253Riyl						anonymous	EN	
2023-02-12 4:50	2023-02-12 4:53	IP Address	100	186	TRUE	2023-02-12 4:53	R_9EkPc67DvyRVcbv						anonymous	EN	
2023-02-12 11:1	2023-02-12 11:1	IP Address	100	25	TRUE	2023-02-12 11:1	R_x0cg5KB3gZz7XnH						anonymous	EN	
2023-02-12 11:1	2023-02-12 11:1	IP Address	100	36	TRUE	2023-02-12 11:1	R_3ktxgBbNf9ubnWj						anonymous	EN	
2023-02-12 11:1	2023-02-12 11:1	IP Address	100	22	TRUE	2023-02-12 11:1	R_3JdTtJjjLdeM2mx						anonymous	EN	
2023-02-12 11:2	2023-02-12 11:3	IP Address	100	473	TRUE	2023-02-12 11:3	R_01AaqxUjKix67MB						anonymous	EN	
2023-02-13 15:4	2023-02-13 15:4	IP Address	100	45	TRUE	2023-02-13 15:4	R_2XmlPsCD19PEvqt						anonymous	EN	
2023-02-13 15:4	2023-02-13 15:4	IP Address	100	144	TRUE	2023-02-13 15:4	R_3nGgAwz1tHU1arJ						anonymous	EN	
2023-02-13 15:4	2023-02-13 15:5	IP Address	100	564	TRUE	2023-02-13 15:5	R_3kA5NV7sE5mdmpz						anonymous	EN	
2023-02-13 15:5	2023-02-13 16:1	IP Address	100	1389	TRUE	2023-02-13 16:1	R_2X0DGzQN2b4WQZX						anonymous	EN	
2023-02-13 16:1	2023-02-13 16:3	IP Address	100	1116	TRUE	2023-02-13 16:3	R_27iXlfvDc2Hn1T8						anonymous	EN	
2023-02-12 4:53	2023-02-14 3:18	IP Address	100	167058	TRUE	2023-02-14 3:18	R_1IWbjvWT8A1kcUA						anonymous	EN	
2023-02-14 3:18	2023-02-14 3:27	IP Address	100	567	TRUE	2023-02-14 3:27	R_24bIFgXZpAXlJAJ						anonymous	EN	
2023-02-14 3:27	2023-02-14 3:35	IP Address	100	482	TRUE	2023-02-14 3:35	R_3CUgDoGFk20yNmk						anonymous	EN	
2023-02-14 3:38	2023-02-14 3:45	IP Address	100	454	TRUE	2023-02-14 3:45	R_3lE29nJW6JX6gVV						anonymous	EN	
2023-02-14 3:45	2023-02-14 3:48	IP Address	100	145	TRUE	2023-02-14 3:48	R_Ucklm3tn7SVxn4B						anonymous	EN	
2023-02-14 3:48	2023-02-14 3:49	IP Address	100	64	TRUE	2023-02-14 3:49	R_2PC2ZpJfZJ8j9lH						anonymous	EN	
2023-02-14 3:49	2023-02-14 3:53	IP Address	100	272	TRUE	2023-02-14 3:53	R_2dsShy7xi9121GQ						anonymous	EN	
2023-02-16 21:5	2023-02-16 22:0	IP Address	100	264	TRUE	2023-02-16 22:0	R_3JhhO2zl4FSZdVj						anonymous	EN	
2023-02-16 22:1	2023-02-16 22:1	IP Address	100	164	TRUE	2023-02-16 22:1	R_3HpI2ARIkU3hTG7						anonymous	EN	
2023-02-16 22:1	2023-02-16 22:1	IP Address	100	108	TRUE	2023-02-16 22:1	R_1gNmgcA0BFlJHrm						anonymous	EN	
2023-02-16 22:1	2023-02-16 22:1	IP Address	100	55	TRUE	2023-02-16 22:1	R_WwUy6DDJzRMls0p						anonymous	EN	
2023-02-16 22:2	2023-02-16 22:2	IP Address	100	45	TRUE	2023-02-16 22:2	R_3Lb9gQF7UlAvmM3						anonymous	EN	
2023-02-16 22:2	2023-02-16 22:2	IP Address	100	363	TRUE	2023-02-16 22:2	R_e5RUNO4pAEj4lHP						anonymous	EN	
2023-02-17 5:42	2023-02-17 6:53	IP Address	100	4272	TRUE	2023-02-17 6:53	R_ZmCLcls7m8KYSM9						anonymous	EN	
2023-02-17 6:53	2023-02-17 6:54	IP Address	100	47	TRUE	2023-02-17 6:54	R_3E9ACuI3JyvEL79						anonymous	EN	
2023-02-17 6:54	2023-02-17 7:02	IP Address	100	494	TRUE	2023-02-17 7:02	R_3hfEU94loYafC4o						anonymous	EN	
2023-02-17 7:02	2023-02-17 7:21	IP Address	100	1113	TRUE	2023-02-17 7:21	R_2X4ekqKHQDVc0sU						anonymous	EN	
2023-02-17 7:21	2023-02-17 7:30	IP Address	100	496	TRUE	2023-02-17 7:30	R_0iV7836NxSVKOHL						anonymous	EN	
2023-02-17 7:30	2023-02-17 7:35	IP Address	100	301	TRUE	2023-02-17 7:35	R_BsrddDxQTtKk9VL						anonymous	EN	
2023-02-17 9:25	2023-02-17 15:1	IP Address	100	21247	TRUE	2023-02-17 15:1	R_10CTd5sZTokgDiH						anonymous	EN	
2023-02-17 15:1	2023-02-17 15:2	IP Address	100	49	TRUE	2023-02-17 15:2	R_2YK9tl5dmXWmthO						anonymous	EN	
2023-02-17 15:2	2023-02-17 15:2	IP Address	100	41	TRUE	2023-02-17 15:2	R_1QhAPFQJw579rh4						anonymous	EN	
2023-02-17 15:2	2023-02-17 15:2	IP Address	100	63	TRUE	2023-02-17 15:2	R_2pS7Pz1nMEsXJ46						anonymous	EN	
2023-02-17 15:2	2023-02-17 15:3	IP Address	100	316	TRUE	2023-02-17 15:3	R_27PTlaC2iVk3wmO						anonymous	EN	
2023-02-17 15:3	2023-02-17 15:4	IP Address	100	733	TRUE	2023-02-17 15:4	R_3n7YOWNzVL39bF7						anonymous	EN	
2023-02-17 15:4	2023-02-17 15:4	IP Address	100	185	TRUE	2023-02-17 15:4	R_1QAgBaQEye06ES8						anonymous	EN	
2023-02-17 15:4	2023-02-17 15:5	IP Address	100	101	TRUE	2023-02-17 15:5	R_1kY35skaZ5JnnXv						anonymous	EN	
2023-02-17 15:5	2023-02-17 15:5	IP Address	100	134	TRUE	2023-02-17 15:5	R_31bH1SYl7M9fqq6						anonymous	EN	
2023-02-17 15:5	2023-02-17 15:5	IP Address	100	145	TRUE	2023-02-17 15:5	R_1oImRf5YuOo1JlR						anonymous	EN	
2023-02-17 15:5	2023-02-17 15:5	IP Address	100	113	TRUE	2023-02-17 15:5	R_29hwYEHAEWxnfG6						anonymous	EN	
2023-02-17 15:5	2023-02-17 16:0	IP Address	100	246	TRUE	2023-02-17 16:0	R_1kUKCsxXESx5AVB						anonymous	EN	
2023-02-17 16:0	2023-02-17 16:0	IP Address	100	33	TRUE	2023-02-17 16:0	R_2YQ7GxsbiPehXXu						anonymous	EN	
2023-02-17 12:3	2023-02-17 20:4	IP Address	100	29437	TRUE	2023-02-17 20:4	R_3qmMm3gJ1AZ7uNu						anonymous	EN	
2023-02-18 8:00	2023-02-18 8:05	IP Address	100	301	TRUE	2023-02-18 8:05	R_3NCYb0sb1FjvuTK						anonymous	EN	
2023-02-18 8:05	2023-02-18 8:09	IP Address	100	202	TRUE	2023-02-18 8:09	R_3ozjGc4DTobdKEy						anonymous	EN	
2023-02-18 8:09	2023-02-18 8:12	IP Address	100	183	TRUE	2023-02-18 8:12	R_2v1R81DKupL15En						anonymous	EN	
2023-02-18 8:12	2023-02-18 8:15	IP Address	100	196	TRUE	2023-02-18 8:15	R_AGWiXLi9tTCYWdj						anonymous	EN	
2023-02-18 8:15	2023-02-18 8:16	IP Address	100	31	TRUE	2023-02-18 8:16	R_30w8LrxPJBzau0T						anonymous	EN	
2023-02-18 8:16	2023-02-18 8:20	IP Address	100	277	TRUE	2023-02-18 8:20	R_31EGmFCjUrVC6Vw						anonymous	EN	

2023-02-18 8:20	2023-02-18 8:23	IP Address	100	142	TRUE	2023-02-18 8:23	R_10DgjPcbD4x0aFQ						anonymous	EN	
2023-02-18 8:23	2023-02-18 8:26	IP Address	100	181	TRUE	2023-02-18 8:26	R_AHbngnbYQRXhNtL						anonymous	EN	
2023-02-18 8:26	2023-02-18 9:37	IP Address	100	4238	TRUE	2023-02-18 9:37	R_pxCd8npyBe9RHCV						anonymous	EN	
2023-02-18 9:37	2023-02-18 9:42	IP Address	100	342	TRUE	2023-02-18 9:42	R_3nIREpMLfRy9y41						anonymous	EN	
2023-02-18 9:43	2023-02-18 9:46	IP Address	100	170	TRUE	2023-02-18 9:46	R_2D1waplg2DfH0rD						anonymous	EN	
2023-02-18 9:46	2023-02-18 9:54	IP Address	100	485	TRUE	2023-02-18 9:54	R_25t0AvTnQphPwF6						anonymous	EN	
2023-02-18 9:55	2023-02-18 10:0	IP Address	100	296	TRUE	2023-02-18 10:0	R_PNXqXf9SbBECyqZ						anonymous	EN	
2023-02-18 10:0	2023-02-18 10:0	IP Address	100	400	TRUE	2023-02-18 10:0	R_XsQLQSzQnaFqWqd						anonymous	EN	
2023-02-18 10:0	2023-02-18 10:1	IP Address	100	236	TRUE	2023-02-18 10:1	R_3rIcGoF3VL7OKqB						anonymous	EN	
2023-02-18 10:1	2023-02-18 10:1	IP Address	100	234	TRUE	2023-02-18 10:1	R_3Emcm4QiIvC1Pa7						anonymous	EN	
2023-02-18 10:1	2023-02-18 10:1	IP Address	100	247	TRUE	2023-02-18 10:1	R_2VgpBeET16onvwL						anonymous	EN	
2023-02-18 10:2	2023-02-18 10:2	IP Address	100	534	TRUE	2023-02-18 10:2	R_1MPQpYisdtZIjJH						anonymous	EN	
2023-02-18 10:2	2023-02-18 10:3	IP Address	100	338	TRUE	2023-02-18 10:3	R_3j1mKJ1fPuDfaD0						anonymous	EN	
2023-02-18 10:3	2023-02-18 10:3	IP Address	100	281	TRUE	2023-02-18 10:3	R_1kG2TBaVx7UphHD						anonymous	EN	
2023-02-18 10:3	2023-02-18 10:4	IP Address	100	259	TRUE	2023-02-18 10:4	R_2YgCslBcGX3JxwV						anonymous	EN	
2023-02-18 10:4	2023-02-18 10:4	IP Address	100	311	TRUE	2023-02-18 10:4	R_1pKe4qqGaIWCL6E						anonymous	EN	
2023-02-18 10:4	2023-02-18 10:5	IP Address	100	90	TRUE	2023-02-18 10:5	R_1oG7ip83lkZhR1d						anonymous	EN	
2023-02-18 10:5	2023-02-18 10:5	IP Address	100	343	TRUE	2023-02-18 10:5	R_3dQ7VytPZglH3WO						anonymous	EN	
2023-02-18 10:5	2023-02-18 10:5	IP Address	100	136	TRUE	2023-02-18 10:5	R_3QGgxRoapRToi8L						anonymous	EN	
2023-02-18 10:5	2023-02-18 11:0	IP Address	100	80	TRUE	2023-02-18 11:0	R_1DZuphWycev7i83						anonymous	EN	
2023-02-18 11:0	2023-02-18 11:0	IP Address	100	365	TRUE	2023-02-18 11:0	R_2UXIiOizgJeqpqf						anonymous	EN	
2023-02-18 11:0	2023-02-18 11:0	IP Address	100	27	TRUE	2023-02-18 11:0	R_1Ivihm7OAxCJDqg						anonymous	EN	
2023-02-18 11:0	2023-02-18 11:1	IP Address	100	407	TRUE	2023-02-18 11:1	R_26ewC6NghZsKlbs						anonymous	EN	
2023-02-18 11:1	2023-02-18 11:1	IP Address	100	185	TRUE	2023-02-18 11:1	R_1ClrOIs3l4M8KTx						anonymous	EN	
2023-02-18 11:1	2023-02-18 11:2	IP Address	100	202	TRUE	2023-02-18 11:2	R_1PSJ52xA5Y21P03						anonymous	EN	
2023-02-18 11:2	2023-02-18 11:2	IP Address	100	122	TRUE	2023-02-18 11:2	R_2TTNHGpFNOnrH1F						anonymous	EN	
2023-02-18 11:2	2023-02-18 11:3	IP Address	100	418	TRUE	2023-02-18 11:3	R_2fBdJQKfG2CUa66						anonymous	EN	
2023-02-18 11:3	2023-02-18 11:3	IP Address	100	413	TRUE	2023-02-18 11:3	R_Z7PgJnvjATsvlfz						anonymous	EN	
2023-02-18 11:3	2023-02-18 11:4	IP Address	100	209	TRUE	2023-02-18 11:4	R_71UBQttIapyINVn						anonymous	EN	
2023-02-18 11:4	2023-02-18 11:4	IP Address	100	141	TRUE	2023-02-18 11:4	R_2CD29MhSX8Dl90I						anonymous	EN	
2023-02-18 11:4	2023-02-18 11:5	IP Address	100	413	TRUE	2023-02-18 11:5	R_2xLyYdP7IbkP57g						anonymous	EN	
2023-02-18 11:5	2023-02-18 11:5	IP Address	100	51	TRUE	2023-02-18 11:5	R_2e9xqStBueiLUkM						anonymous	EN	
2023-02-18 11:5	2023-02-18 11:5	IP Address	100	210	TRUE	2023-02-18 11:5	R_qOvDoBP6hme19gB						anonymous	EN	
2023-02-18 11:5	2023-02-18 11:5	IP Address	100	136	TRUE	2023-02-18 11:5	R_3LYfdWrEj3Pmtza						anonymous	EN	
2023-02-17 20:4	2023-02-18 15:2	IP Address	100	67384	TRUE	2023-02-18 15:2	R_3NxrtPbJr4vOKkw						anonymous	EN	
2023-02-18 15:2	2023-02-18 15:2	IP Address	100	209	TRUE	2023-02-18 15:2	R_1giJq8aaTsnMkuJ						anonymous	EN	
2023-02-19 5:26	2023-02-19 5:33	IP Address	100	425	TRUE	2023-02-19 5:33	R_30kj5Xe6ePBuAZb						anonymous	EN	
2023-02-19 5:33	2023-02-19 5:37	IP Address	100	265	TRUE	2023-02-19 5:37	R_2cdUms9KlCyE2bF						anonymous	EN	
2023-02-19 5:37	2023-02-19 5:43	IP Address	100	344	TRUE	2023-02-19 5:43	R_3DiU3ULGcd6dBzr						anonymous	EN	
2023-02-19 5:43	2023-02-19 5:44	IP Address	100	25	TRUE	2023-02-19 5:44	R_3oT0R3r0dGm77YS						anonymous	EN	
2023-02-19 5:44	2023-02-19 5:45	IP Address	100	54	TRUE	2023-02-19 5:45	R_3LXO6drvEO4cNGz						anonymous	EN	
2023-02-19 5:45	2023-02-19 5:48	IP Address	100	169	TRUE	2023-02-19 5:48	R_3nN0LFeQOHjVd4A						anonymous	EN	
2023-02-19 5:48	2023-02-19 5:54	IP Address	100	366	TRUE	2023-02-19 5:54	R_pN0nEMxVaCpXFg5						anonymous	EN	
2023-02-19 5:54	2023-02-19 5:54	IP Address	100	15	TRUE	2023-02-19 5:54	R_10DCVBKG98EN6gs						anonymous	EN	
2023-02-19 5:54	2023-02-19 5:56	IP Address	100	91	TRUE	2023-02-19 5:56	R_3mn4ppFo367NIbq						anonymous	EN	
2023-02-19 5:56	2023-02-19 5:56	IP Address	100	33	TRUE	2023-02-19 5:56	R_POQ4b50rBSf6Xsd						anonymous	EN	
2023-02-19 5:56	2023-02-19 5:57	IP Address	100	31	TRUE	2023-02-19 5:57	R_1nPnuwcC1HMK7Dc						anonymous	EN	
2023-02-19 5:57	2023-02-19 5:58	IP Address	100	76	TRUE	2023-02-19 5:58	R_WoFenwfSSWoIELv						anonymous	EN	
2023-02-19 5:58	2023-02-19 17:0	IP Address	100	39815	TRUE	2023-02-19 17:0	R_2QlH0h8zcwvHbjQ						anonymous	EN	
2023-02-19 17:0	2023-02-19 17:0	IP Address	100	322	TRUE	2023-02-19 17:0	R_3Gyd3jQjvp0GbFo						anonymous	EN	
2023-02-19 17:0	2023-02-19 17:1	IP Address	100	502	TRUE	2023-02-19 17:1	R_3I51fQANSNHnALv						anonymous	EN	
2023-02-19 17:1	2023-02-19 17:1	IP Address	100	202	TRUE	2023-02-19 17:1	R_3kNdZpI0NXq3rAa						anonymous	EN	
2023-02-19 17:1	2023-02-19 17:2	IP Address	100	383	TRUE	2023-02-19 17:2	R_2AKEoSgDOGCKUWb						anonymous	EN	
2023-02-19 17:2	2023-02-19 17:3	IP Address	100	414	TRUE	2023-02-19 17:3	R_1gotyZO7jtJGkCI						anonymous	EN	
2023-02-19 17:3	2023-02-19 17:3	IP Address	100	308	TRUE	2023-02-19 17:3	R_3h0wDNWrnxqXrBH						anonymous	EN	
2023-02-19 17:3	2023-02-19 17:4	IP Address	100	219	TRUE	2023-02-19 17:4	R_1QMGDSAHNSVfnnJ						anonymous	EN	
2023-02-18 15:3	2023-02-19 20:2	IP Address	100	103906	TRUE	2023-02-19 20:2	R_Y4aWQ9ndDO2cRRn						anonymous	EN	
2023-02-19 20:2	2023-02-19 20:2	IP Address	100	157	TRUE	2023-02-19 20:2	R_3PN8SW9saI0lI4q						anonymous	EN	
2023-02-19 20:2	2023-02-19 20:2	IP Address	100	124	TRUE	2023-02-19 20:2	R_vq6EnVPuxDTNpjX						anonymous	EN	

2023-02-19 20:2	2023-02-19 20:3	IP Address	100	169	TRUE	2023-02-19 20:3	R_3lSnQWm615ivegG						anonymous	EN	
2023-02-19 20:5	2023-02-19 20:5	IP Address	100	198	TRUE	2023-02-19 20:5	R_1mDEpmfMlmRYSKm						anonymous	EN	
2023-02-19 20:5	2023-02-19 21:2	IP Address	100	1664	TRUE	2023-02-19 21:2	R_3mfdsPZJgin2mll						anonymous	EN	
2023-02-19 21:2	2023-02-19 21:2	IP Address	100	260	TRUE	2023-02-19 21:2	R_2cthwbVvAUVRQ4b						anonymous	EN	
2023-02-19 21:4	2023-02-19 21:4	IP Address	100	253	TRUE	2023-02-19 21:4	R_23fO26kT9NCefoY						anonymous	EN	
2023-02-19 21:4	2023-02-19 21:5	IP Address	100	52	TRUE	2023-02-19 21:5	R_1ohuSQfWwS4hGCS						anonymous	EN	
2023-02-19 21:5	2023-02-19 21:5	IP Address	100	362	TRUE	2023-02-19 21:5	R_2WPF7EuJVig2j23						anonymous	EN	
2023-02-20 8:40:	2023-02-20 8:50:	IP Address	100	600	TRUE	2023-02-20 8:50:	R_25SF1LmBkuVkemF						anonymous	EN	
2023-02-20 8:50:	2023-02-20 8:55:	IP Address	100	297	TRUE	2023-02-20 8:55:	R_z1HvyjEgmUeymVb						anonymous	EN	
2023-02-20 8:55:	2023-02-20 8:56:	IP Address	100	55	TRUE	2023-02-20 8:56:	R_2s0f65V8pfYSFYI						anonymous	EN	
2023-02-20 8:56:	2023-02-20 8:57:	IP Address	100	104	TRUE	2023-02-20 8:57:	R_1H0IXubtawe4oEp						anonymous	EN	
2023-02-20 8:58:	2023-02-20 9:08:	IP Address	100	634	TRUE	2023-02-20 9:08:	R_OyhzRqc3hZ5woff						anonymous	EN	
2023-02-20 9:08:	2023-02-20 9:12:	IP Address	100	242	TRUE	2023-02-20 9:12:	R_eqT2Knwa62pe5Fv						anonymous	EN	
2023-02-20 9:12:	2023-02-20 9:17:	IP Address	100	319	TRUE	2023-02-20 9:18:	R_1CHlZMLv8e8P27j						anonymous	EN	
2023-02-20 9:18:	2023-02-20 9:19:	IP Address	100	63	TRUE	2023-02-20 9:19:	R_21hSEdYduxKRasi						anonymous	EN	
2023-02-20 9:19:	2023-02-20 9:19:	IP Address	100	46	TRUE	2023-02-20 9:19:	R_aaCdGbyk44s8kWB						anonymous	EN	
2023-02-20 9:19:	2023-02-20 16:3	IP Address	100	25883	TRUE	2023-02-20 16:3	R_1QF8qh7DwbMUbV2						anonymous	EN	
2023-02-20 16:3	2023-02-20 16:3	IP Address	100	131	TRUE	2023-02-20 16:3	R_1n1HtdBqFDTcfTB						anonymous	EN	
2023-02-20 16:3	2023-02-20 16:3	IP Address	100	222	TRUE	2023-02-20 16:3	R_1IElCuhViZpWklo						anonymous	EN	
2023-02-20 16:3	2023-02-20 16:4	IP Address	100	295	TRUE	2023-02-20 16:4	R_2VJQDx5CJbHYtFF						anonymous	EN	
2023-02-20 16:4	2023-02-20 16:4	IP Address	100	264	TRUE	2023-02-20 16:4	R_1lfRt2q7GhLyoMg						anonymous	EN	
2023-02-20 16:4	2023-02-20 16:5	IP Address	100	87	TRUE	2023-02-20 16:5	R_3hxP4Q4t4DJHMBr						anonymous	EN	
2023-02-20 16:5	2023-02-20 16:5	IP Address	100	276	TRUE	2023-02-20 16:5	R_2q89XNDSIT4f9vp						anonymous	EN	
2023-02-20 16:5	2023-02-20 16:5	IP Address	100	27	TRUE	2023-02-20 16:5	R_3RkYaCmfB5GMMCV						anonymous	EN	
2023-02-20 16:5	2023-02-20 16:5	IP Address	100	52	TRUE	2023-02-20 16:5	R_2c6ZXTtOV1WaohZ						anonymous	EN	
2023-02-20 16:5	2023-02-20 17:0	IP Address	100	255	TRUE	2023-02-20 17:0	R_2AN0QeRNjkgfFhN						anonymous	EN	
2023-02-21 13:5	2023-02-21 14:1	IP Address	100	1004	TRUE	2023-02-21 14:1	R_u2ZKeHzkEseZ4ch						anonymous	EN	
2023-02-21 14:1	2023-02-21 14:1	IP Address	100	191	TRUE	2023-02-21 14:1	R_Y9Yj04Cpz7qVwUV						anonymous	EN	
2023-02-21 14:2	2023-02-21 14:2	IP Address	100	207	TRUE	2023-02-21 14:2	R_21o1jv4BEVoWxCW						anonymous	EN	
2023-02-21 14:3	2023-02-21 14:4	IP Address	100	127	TRUE	2023-02-21 14:4	R_1P61vGNcHWfokTa						anonymous	EN	
2023-02-21 14:4	2023-02-21 14:4	IP Address	100	101	TRUE	2023-02-21 14:4	R_1rkPGXTnCxTrnqb						anonymous	EN	
2023-02-21 15:3	2023-02-21 15:3	IP Address	100	146	TRUE	2023-02-21 15:3	R_2Tt3tnClGkzGYCa						anonymous	EN	
2023-02-22 13:1	2023-02-22 13:1	IP Address	100	273	TRUE	2023-02-22 13:1	R_eIGBUQgqBZEYE2B						anonymous	EN	
2023-02-22 13:1	2023-02-22 13:1	IP Address	100	47	TRUE	2023-02-22 13:1	R_2S6xad1pYUqL4Rg						anonymous	EN	
2023-02-22 13:1	2023-02-22 13:1	IP Address	100	43	TRUE	2023-02-22 13:1	R_2zpC3Wiak8Xu8U5						anonymous	EN	
2023-02-22 13:1	2023-02-22 13:1	IP Address	100	63	TRUE	2023-02-22 13:1	R_3Reuqyb7dwSvHrz						anonymous	EN	
2023-02-22 13:1	2023-02-22 13:2	IP Address	100	195	TRUE	2023-02-22 13:2	R_2a9OjofpfKc2ZuC						anonymous	EN	
2023-02-22 13:2	2023-02-22 13:2	IP Address	100	16	TRUE	2023-02-22 13:2	R_vGhB4feVeWKUnhD						anonymous	EN	
2023-02-22 13:2	2023-02-22 13:3	IP Address	100	505	TRUE	2023-02-22 13:3	R_3Kpy1iLxmEqGwXx						anonymous	EN	
2023-02-22 13:3	2023-02-22 13:3	IP Address	100	50	TRUE	2023-02-22 13:3	R_6S8JUn3VgS5Ogfv						anonymous	EN	
2023-02-22 13:3	2023-02-22 13:3	IP Address	100	252	TRUE	2023-02-22 13:3	R_1hXCNsqB3Lusgk5						anonymous	EN	
2023-02-22 13:3	2023-02-22 13:4	IP Address	100	207	TRUE	2023-02-22 13:4	R_233OUA5351kufu5						anonymous	EN	
2023-02-22 13:4	2023-02-22 13:4	IP Address	100	188	TRUE	2023-02-22 13:4	R_2uBDBtsTHOlS3a2						anonymous	EN	
2023-02-22 13:4	2023-02-22 13:4	IP Address	100	29	TRUE	2023-02-22 13:4	R_1NfPNqvqjh9t0pM						anonymous	EN	
2023-02-22 13:4	2023-02-22 13:4	IP Address	100	77	TRUE	2023-02-22 13:4	R_pEOi8GaQyWL6aXf						anonymous	EN	
2023-02-22 13:4	2023-02-22 13:4	IP Address	100	35	TRUE	2023-02-22 13:4	R_239aZZlMHPxFfDQ						anonymous	EN	
2023-02-22 13:4	2023-02-22 13:5	IP Address	100	215	TRUE	2023-02-22 13:5	R_2SdEZuuoMMzO7Zc						anonymous	EN	
2023-02-22 13:5	2023-02-22 13:5	IP Address	100	146	TRUE	2023-02-22 13:5	R_3pgRoOCQTjnhbuw						anonymous	EN	
2023-02-22 13:5	2023-02-22 13:5	IP Address	100	35	TRUE	2023-02-22 13:5	R_0ww68H8mJOME0A9						anonymous	EN	
2023-02-22 13:5	2023-02-22 13:5	IP Address	100	362	TRUE	2023-02-22 13:5	R_3sjEUnl39uPog6U						anonymous	EN	
2023-02-22 13:5	2023-02-22 16:4	IP Address	100	10012	TRUE	2023-02-22 16:4	R_AFg9i6yOx9xh6w1						anonymous	EN	
2023-02-22 16:4	2023-02-22 16:5	IP Address	100	264	TRUE	2023-02-22 16:5	R_1NkTfbhqYbA1Bgg						anonymous	EN	
2023-02-22 16:5	2023-02-22 16:5	IP Address	100	255	TRUE	2023-02-22 16:5	R_3PNURMIyCfv3bnC						anonymous	EN	
2023-02-22 16:5	2023-02-22 17:0	IP Address	100	249	TRUE	2023-02-22 17:0	R_1jTgo4UUly7rEGW						anonymous	EN	
2023-02-22 16:5	2023-02-22 17:0	IP Address	100	690	TRUE	2023-02-22 17:0	R_3lQVsn4SXKm2vGq						anonymous	EN	
2023-02-22 17:0	2023-02-22 17:0	IP Address	100	165	TRUE	2023-02-22 17:0	R_2WvtyHA02aYwJ6s						anonymous	EN	
2023-02-22 17:0	2023-02-22 17:0	IP Address	100	180	TRUE	2023-02-22 17:0	R_1QLVHjhwzi9nPTg						anonymous	EN	
2023-02-22 17:0	2023-02-22 17:1	IP Address	100	355	TRUE	2023-02-22 17:1	R_1ln3I4V5bYVDGMl						anonymous	EN	
2023-02-22 17:1	2023-02-22 17:1	IP Address	100	137	TRUE	2023-02-22 17:1	R_e5Tn7JdKEiLzXVL						anonymous	EN	

2023-02-22 17:1	2023-02-22 17:1	IP Address	100	110	TRUE	2023-02-22 17:1	R_26lQh23GKpiGt0j							anonymous	EN	
2023-02-22 17:1	2023-02-22 17:2	IP Address	100	380	TRUE	2023-02-22 17:2	R_1KvafByi5bRYkBh							anonymous	EN	
2023-02-22 17:2	2023-02-22 17:2	IP Address	100	84	TRUE	2023-02-22 17:2	R_DvLeC9IB5Bm28sF							anonymous	EN	
2023-02-22 17:2	2023-02-22 17:2	IP Address	100	33	TRUE	2023-02-22 17:2	R_1QN0DU8oBsJLHre							anonymous	EN	
2023-02-22 17:2	2023-02-22 17:3	IP Address	100	259	TRUE	2023-02-22 17:3	R_3rZD5wfC8uHC6F2							anonymous	EN	
2023-02-22 17:3	2023-02-22 17:3	IP Address	100	377	TRUE	2023-02-22 17:3	R_1CBDLIPU4fw4Sk7							anonymous	EN	
2023-02-22 17:3	2023-02-22 17:3	IP Address	100	136	TRUE	2023-02-22 17:3	R_WiYBdYoASPGwjHb							anonymous	EN	
2023-02-22 17:3	2023-02-22 17:4	IP Address	100	175	TRUE	2023-02-22 17:4	R_vP2smMdkYv1FDB7							anonymous	EN	
2023-02-22 17:4	2023-02-22 17:4	IP Address	100	114	TRUE	2023-02-22 17:4	R_1ibxKQZQaZ83RJF							anonymous	EN	
2023-02-22 17:4	2023-02-22 17:4	IP Address	100	283	TRUE	2023-02-22 17:4	R_2wpg1Il12acFCCL							anonymous	EN	
2023-02-22 17:4	2023-02-22 17:5	IP Address	100	163	TRUE	2023-02-22 17:5	R_6VANIgdJYcpMRrj							anonymous	EN	
2023-02-22 17:5	2023-02-22 17:5	IP Address	100	249	TRUE	2023-02-22 17:5	R_2Yu2VqpEesO96WT							anonymous	EN	
2023-02-22 17:5	2023-02-22 18:0	IP Address	100	213	TRUE	2023-02-22 18:0	R_2YXOlpWCsrXcyd9							anonymous	EN	
2023-02-23 7:45	2023-02-23 7:55	IP Address	100	582	TRUE	2023-02-23 7:55	R_PU7Pw27cTcelhrX							anonymous	EN	
2023-02-23 7:55	2023-02-23 7:58	IP Address	100	156	TRUE	2023-02-23 7:58	R_1mfsXB27q94pWcA							anonymous	EN	
2023-02-23 7:58	2023-02-23 8:03	IP Address	100	326	TRUE	2023-02-23 8:03	R_RQ54zOfvigsenwl							anonymous	EN	
2023-02-23 8:03	2023-02-23 8:08	IP Address	100	280	TRUE	2023-02-23 8:08	R_1dGGRpwfSZJYaJf							anonymous	EN	
2023-02-23 8:08	2023-02-23 8:14	IP Address	100	364	TRUE	2023-02-23 8:14	R_1170IMHpsAWfnPQ							anonymous	EN	
2023-02-23 8:14	2023-02-23 8:17	IP Address	100	136	TRUE	2023-02-23 8:17	R_2Xj21iBG35AKtVG							anonymous	EN	
2023-02-23 8:17	2023-02-23 8:35	IP Address	100	1125	TRUE	2023-02-23 8:35	R_1r3zYL9joidQ6xF							anonymous	EN	
2023-02-23 8:35	2023-02-23 8:37	IP Address	100	87	TRUE	2023-02-23 8:37	R_2YWavYrxK57UKlr							anonymous	EN	
2023-02-23 8:37	2023-02-23 8:51	IP Address	100	874	TRUE	2023-02-23 8:51	R_D5yXrL3xzZKatk5							anonymous	EN	
2023-02-23 8:51	2023-02-23 9:02	IP Address	100	637	TRUE	2023-02-23 9:02	R_1mftIPMFXQ6jmx3							anonymous	EN	
2023-02-23 10:2	2023-02-23 10:3	IP Address	100	409	TRUE	2023-02-23 10:3	R_3lY4C3uockESWwi							anonymous	EN	
2023-02-23 10:3	2023-02-23 10:3	IP Address	100	309	TRUE	2023-02-23 10:3	R_3NOroCUXdxWy2W8							anonymous	EN	
2023-02-23 10:4	2023-02-23 10:4	IP Address	100	216	TRUE	2023-02-23 10:4	R_3RfQD31wx6tRxud							anonymous	EN	
2023-02-23 15:0	2023-02-23 15:2	IP Address	100	972	TRUE	2023-02-23 15:2	R_bdWgWGxWBVGcf3H							anonymous	EN	
2023-02-23 15:2	2023-02-23 15:2	IP Address	100	252	TRUE	2023-02-23 15:2	R_3qwdKiANjca8QT2							anonymous	EN	
2023-02-23 15:2	2023-02-23 15:3	IP Address	100	134	TRUE	2023-02-23 15:3	R_3PzrFgHChkODCUO							anonymous	EN	
2023-02-23 15:3	2023-02-23 15:3	IP Address	100	198	TRUE	2023-02-23 15:3	R_SPppXzn1oK1wsWR							anonymous	EN	
2023-02-23 15:3	2023-02-23 15:3	IP Address	100	263	TRUE	2023-02-23 15:3	R_3240tHbQzVmE6Fr							anonymous	EN	
2023-02-23 15:3	2023-02-23 15:4	IP Address	100	117	TRUE	2023-02-23 15:4	R_SNLk6trc5lu7jRn							anonymous	EN	
2023-02-23 15:4	2023-02-23 15:4	IP Address	100	136	TRUE	2023-02-23 15:4	R_12u4ssbvw9HuZk5							anonymous	EN	
2023-02-23 15:4	2023-02-23 15:4	IP Address	100	154	TRUE	2023-02-23 15:4	R_1WXpetIS7Qu9AJP							anonymous	EN	
2023-02-23 15:4	2023-02-23 15:4	IP Address	100	93	TRUE	2023-02-23 15:4	R_2P0VoB2FMeBUD8d							anonymous	EN	
2023-02-23 15:4	2023-02-23 15:5	IP Address	100	559	TRUE	2023-02-23 15:5	R_Y4yo0EtxjXOX7lD							anonymous	EN	
2023-02-23 15:5	2023-02-23 16:0	IP Address	100	207	TRUE	2023-02-23 16:0	R_3fIrwKDsSlLFu41							anonymous	EN	
2023-02-23 16:0	2023-02-24 11:0	IP Address	100	68408	TRUE	2023-02-24 11:0	R_3hDrrPvNg0NSBJj							anonymous	EN	
2023-02-24 11:0	2023-02-24 11:0	IP Address	100	478	TRUE	2023-02-24 11:0	R_2EGnSQSwGnJ9jkp							anonymous	EN	
2023-02-24 11:0	2023-02-24 11:1	IP Address	100	137	TRUE	2023-02-24 11:1	R_2CNzcAWciIXchhm							anonymous	EN	
2023-02-24 11:1	2023-02-24 11:1	IP Address	100	216	TRUE	2023-02-24 11:1	R_2TEOFo0otA5HDRX							anonymous	EN	
2023-02-24 11:1	2023-02-24 11:1	IP Address	100	147	TRUE	2023-02-24 11:1	R_3HwDEdbONliy3mY							anonymous	EN	
2023-02-24 11:1	2023-02-24 11:1	IP Address	100	49	TRUE	2023-02-24 11:1	R_3rHtS8nJHjbIaIz							anonymous	EN	
2023-02-24 11:1	2023-02-24 11:2	IP Address	100	216	TRUE	2023-02-24 11:2	R_3m2BTPkkkgCwJRo							anonymous	EN	
2023-02-24 11:2	2023-02-24 11:2	IP Address	100	134	TRUE	2023-02-24 11:2	R_2XmxNdrfCB0Wiw6							anonymous	EN	
2023-02-24 11:2	2023-02-24 11:3	IP Address	100	575	TRUE	2023-02-24 11:3	R_eF1yvuVktaBzG2B							anonymous	EN	
2023-02-24 11:3	2023-02-24 11:4	IP Address	100	950	TRUE	2023-02-24 11:4	R_1f0G62pdmG0BBPW							anonymous	EN	
2023-02-24 11:2	2023-02-24 11:4	IP Address	100	1387	TRUE	2023-02-24 11:4	R_2f9QZkAGQhNaiwV							anonymous	EN	
2023-02-24 11:4	2023-02-24 11:5	IP Address	100	147	TRUE	2023-02-24 11:5	R_10UjOArrxwxyICI							anonymous	EN	
2023-02-24 11:4	2023-02-24 11:5	IP Address	100	540	TRUE	2023-02-24 11:5	R_1JIrBBT6Ffsjg8o							anonymous	EN	
2023-02-24 11:5	2023-02-24 12:0	IP Address	100	502	TRUE	2023-02-24 12:0	R_2Yt4Zbzyk934s10							anonymous	EN	
2023-02-24 12:0	2023-02-24 12:0	IP Address	100	97	TRUE	2023-02-24 12:0	R_3L1llG1LZr1cd2x							anonymous	EN	
2023-02-24 12:2	2023-02-24 12:3	IP Address	100	1043	TRUE	2023-02-24 12:3	R_ZkkXptnopwYktjj							anonymous	EN	
2023-02-24 12:3	2023-02-24 12:4	IP Address	100	245	TRUE	2023-02-24 12:4	R_3nckUwz9u9txkWe							anonymous	EN	
2023-02-24 12:4	2023-02-24 12:4	IP Address	100	81	TRUE	2023-02-24 12:4	R_PvPWQ8h90y54yeB							anonymous	EN	
2023-02-24 12:5	2023-02-24 13:1	IP Address	100	1259	TRUE	2023-02-24 13:1	R_3G2JrWEiJtr8J7N							anonymous	EN	
2023-02-24 13:1	2023-02-24 13:1	IP Address	100	178	TRUE	2023-02-24 13:1	R_3hzHvqdSK42bU72							anonymous	EN	
2023-02-24 12:0	2023-02-24 13:4	IP Address	100	6062	TRUE	2023-02-24 13:4	R_12LxMXvyJMpuHCg							anonymous	EN	
2023-02-24 13:4	2023-02-24 13:5	IP Address	100	118	TRUE	2023-02-24 13:5	R_2usOagR0kySlYys							anonymous	EN	

2023-02-24 13:5	2023-02-24 13:5	IP Address	100	74	TRUE	2023-02-24 13:5	R_8841tT9TWdA5CXD							anonymous	EN	
2023-02-24 13:5	2023-02-24 13:5	IP Address	100	130	TRUE	2023-02-24 13:5	R_3Ld9LgdSiPiqZoE							anonymous	EN	
2023-02-24 13:5	2023-02-24 14:0	IP Address	100	223	TRUE	2023-02-24 14:0	R_3suVUbqUwucXSOn							anonymous	EN	
2023-02-24 14:0	2023-02-24 14:0	IP Address	100	413	TRUE	2023-02-24 14:0	R_pEP0O0AHRjWaTVD							anonymous	EN	
2023-02-24 14:0	2023-02-24 14:1	IP Address	100	245	TRUE	2023-02-24 14:1	R_1LSD7jrYu6RU6r7							anonymous	EN	
2023-02-24 14:1	2023-02-24 14:1	IP Address	100	66	TRUE	2023-02-24 14:1	R_12EZepWu6vICxXZ							anonymous	EN	
2023-02-24 14:1	2023-02-24 14:1	IP Address	100	199	TRUE	2023-02-24 14:1	R_3U8mBn9xFieSKgV							anonymous	EN	
2023-02-24 14:1	2023-02-24 14:1	IP Address	100	149	TRUE	2023-02-24 14:1	R_2ccMF6bBR0rcSST							anonymous	EN	
2023-02-24 14:2	2023-02-24 14:3	IP Address	100	604	TRUE	2023-02-24 14:3	R_3fld7ZAkwXrPssi							anonymous	EN	
2023-02-24 14:3	2023-02-24 14:4	IP Address	100	573	TRUE	2023-02-24 14:4	R_Q4VOMNXm2Zw66UF							anonymous	EN	
2023-02-24 14:4	2023-02-24 14:5	IP Address	100	626	TRUE	2023-02-24 14:5	R_3J30e94v06K1l7g							anonymous	EN	
2023-02-24 14:5	2023-02-24 14:5	IP Address	100	230	TRUE	2023-02-24 14:5	R_2S0sNK9g0mCrGIj							anonymous	EN	
2023-02-24 14:5	2023-02-24 15:0	IP Address	100	434	TRUE	2023-02-24 15:0	R_zcimeSNS6bcxoSR							anonymous	EN	
2023-02-24 14:5	2023-02-24 15:1	IP Address	100	1066	TRUE	2023-02-24 15:1	R_2QofhqqIJcHN1fO							anonymous	EN	
2023-02-24 15:1	2023-02-24 15:2	IP Address	100	737	TRUE	2023-02-24 15:2	R_1gFT3NN4VsgXkNB							anonymous	EN	
2023-02-24 15:2	2023-02-24 15:4	IP Address	100	929	TRUE	2023-02-24 15:4	R_2PiFVsnGSPx4aTB							anonymous	EN	
2023-02-24 15:4	2023-02-24 17:4	IP Address	100	7199	TRUE	2023-02-24 17:4	R_31oAeUWBFh6H9vv							anonymous	EN	
2023-02-25 3:25:	2023-02-25 3:30:	IP Address	100	259	TRUE	2023-02-25 3:30:	R_1C3Bv4kav5byKgl							anonymous	EN	
2023-02-25 3:30:	2023-02-25 3:33:	IP Address	100	160	TRUE	2023-02-25 3:33:	R_XGQunX1dsAAe0vL							anonymous	EN	
2023-02-25 3:33:	2023-02-25 3:38:	IP Address	100	334	TRUE	2023-02-25 3:38:	R_2afdpCgrbklXsiT							anonymous	EN	
2023-02-25 3:38:	2023-02-25 3:43:	IP Address	100	261	TRUE	2023-02-25 3:43:	R_3CBmvDNWoNEA6NL							anonymous	EN	
2023-02-25 3:43:	2023-02-25 3:48:	IP Address	100	350	TRUE	2023-02-25 3:48:	R_1pJRKlRVilYGxew							anonymous	EN	
2023-02-25 3:49:	2023-02-25 3:54:	IP Address	100	330	TRUE	2023-02-25 3:54:	R_1dtbxBhafeSB0qi							anonymous	EN	
2023-02-25 3:54:	2023-02-25 3:55:	IP Address	100	39	TRUE	2023-02-25 3:55:	R_3GfH0GAksyHwnWh							anonymous	EN	
2023-02-25 3:55:	2023-02-25 4:03:	IP Address	100	525	TRUE	2023-02-25 4:04:	R_2zTSFN5ir81lN25							anonymous	EN	
2023-02-25 4:04:	2023-02-25 4:04:	IP Address	100	39	TRUE	2023-02-25 4:04:	R_3Ryed8jQdhO2CRh							anonymous	EN	
2023-02-25 4:04:	2023-02-25 4:14:	IP Address	100	589	TRUE	2023-02-25 4:14:	R_1KqYVE8XJW8j0sZ							anonymous	EN	
2023-02-25 4:14:	2023-02-25 4:16:	IP Address	100	124	TRUE	2023-02-25 4:16:	R_2EmZMTl01UVnnk0							anonymous	EN	
2023-02-25 4:16:	2023-02-25 4:20:	IP Address	100	220	TRUE	2023-02-25 4:20:	R_3DiQVKB2Wt7TOE2							anonymous	EN	
2023-02-25 4:20:	2023-02-25 4:26:	IP Address	100	362	TRUE	2023-02-25 4:26:	R_pGxoV2glipVbDnX							anonymous	EN	
2023-02-25 4:26:	2023-02-25 4:29:	IP Address	100	159	TRUE	2023-02-25 4:29:	R_d5SCAtesrOTQjkd							anonymous	EN	
2023-02-25 4:29:	2023-02-25 4:31:	IP Address	100	125	TRUE	2023-02-25 4:31:	R_3ff3lEsY9RgKmj9							anonymous	EN	
2023-02-25 4:31:	2023-02-25 4:34:	IP Address	100	172	TRUE	2023-02-25 4:34:	R_WqiNRl23rcMihkR							anonymous	EN	
2023-02-25 4:34:	2023-02-25 4:36:	IP Address	100	159	TRUE	2023-02-25 4:36:	R_3ER0RsC9r59amo2							anonymous	EN	
2023-02-25 4:36:	2023-02-25 4:37:	IP Address	100	40	TRUE	2023-02-25 4:37:	R_phpZU8MZwqlPLUZ							anonymous	EN	
2023-02-25 4:37:	2023-02-25 4:42:	IP Address	100	271	TRUE	2023-02-25 4:42:	R_2EfSicEH9NgJ1um							anonymous	EN	
2023-02-25 4:42:	2023-02-25 4:44:	IP Address	100	136	TRUE	2023-02-25 4:44:	R_2724LkGNw89rzGi							anonymous	EN	
2023-02-25 4:44:	2023-02-25 8:07:	IP Address	100	12209	TRUE	2023-02-25 8:07:	R_1nPBNp8ZxhL39Dt							anonymous	EN	
2023-02-25 8:07:	2023-02-25 8:09:	IP Address	100	71	TRUE	2023-02-25 8:09:	R_2wssvmg4Tk0xIDb							anonymous	EN	
2023-02-25 8:09:	2023-02-25 8:11:	IP Address	100	114	TRUE	2023-02-25 8:11:	R_2upJRmHQULyCzsp							anonymous	EN	
2023-02-25 8:11:	2023-02-25 8:12:	IP Address	100	61	TRUE	2023-02-25 8:12:	R_1DU0D9Cn9iKG2xl							anonymous	EN	
2023-02-25 8:12:	2023-02-25 8:19:	IP Address	100	412	TRUE	2023-02-25 8:19:	R_3flQJIWnpMDgevA							anonymous	EN	
2023-02-25 8:15:	2023-02-25 8:21:	IP Address	100	312	TRUE	2023-02-25 8:21:	R_1BSmXYfub6oaacc							anonymous	EN	
2023-02-25 8:19:	2023-02-25 8:21:	IP Address	100	158	TRUE	2023-02-25 8:21:	R_1ILA9YHLB6t34kn							anonymous	EN	
2023-02-25 8:22:	2023-02-25 8:24:	IP Address	100	95	TRUE	2023-02-25 8:24:	R_33kTliPYYSaGyOg							anonymous	EN	
2023-02-25 8:21:	2023-02-25 8:24:	IP Address	100	153	TRUE	2023-02-25 8:24:	R_308cVbY76w98dgh							anonymous	EN	
2023-02-25 8:24:	2023-02-25 8:27:	IP Address	100	189	TRUE	2023-02-25 8:27:	R_3EXxET9V9gC8P3M							anonymous	EN	
2023-02-25 8:24:	2023-02-25 8:29:	IP Address	100	311	TRUE	2023-02-25 8:29:	R_29pTIOHn78KZah3							anonymous	EN	
2023-02-25 8:29:	2023-02-25 8:40:	IP Address	100	642	TRUE	2023-02-25 8:40:	R_3m7zsH04MkWuxJ4							anonymous	EN	
2023-02-25 8:40:	2023-02-25 8:46:	IP Address	100	370	TRUE	2023-02-25 8:46:	R_2a8HVYMcVqL5BgN							anonymous	EN	
2023-02-25 8:46:	2023-02-25 9:06:	IP Address	100	1223	TRUE	2023-02-25 9:06:	R_2zNIwPuTW6w2McO							anonymous	EN	
2023-02-25 9:07:	2023-02-25 9:09:	IP Address	100	109	TRUE	2023-02-25 9:09:	R_slr7YOBaEuwNx5f							anonymous	EN	
2023-02-25 9:09:	2023-02-25 9:18:	IP Address	100	510	TRUE	2023-02-25 9:18:	R_3dEuGAgneLPu05x							anonymous	EN	
2023-02-25 9:18:	2023-02-25 9:21:	IP Address	100	173	TRUE	2023-02-25 9:21:	R_3JdXzct44tClrCw							anonymous	EN	
2023-02-28 9:13:	2023-02-28 9:17:	IP Address	100	273	TRUE	2023-02-28 9:17:	R_2sSc4Xpniyvh3MK							anonymous	EN	
2023-02-28 9:19:	2023-02-28 9:22:	IP Address	100	170	TRUE	2023-02-28 9:22:	R_3iKjKttBqIlonUD							anonymous	EN	
2023-02-28 9:22:	2023-02-28 9:33:	IP Address	100	644	TRUE	2023-02-28 9:33:	R_1Kd2oQHOIVn0EaO							anonymous	EN	
2023-02-28 9:33:	2023-02-28 9:36:	IP Address	100	209	TRUE	2023-02-28 9:36:	R_WlClcUqqCPqazTP							anonymous	EN	
2023-02-28 9:36:	2023-02-28 9:40:	IP Address	100	195	TRUE	2023-02-28 9:40:	R_9vmsCXet0G6pjGN							anonymous	EN	

2023-03-01 18:2	2023-03-01 19:2	IP Address	100	3783	TRUE	2023-03-01 19:2	R_3iVT46XPTLzCQxT							anonymous	EN	
2023-03-02 7:54	2023-03-02 7:59	IP Address	100	318	TRUE	2023-03-02 8:00	R_3q9ihs3N4jW37lv							anonymous	EN	
2023-03-02 8:56	2023-03-02 8:57	IP Address	100	89	TRUE	2023-03-02 8:57	R_2z5uZyrolUSSvFz							anonymous	EN	
2023-03-02 15:3	2023-03-02 15:5	IP Address	100	1451	TRUE	2023-03-02 15:5	R_28NdM3NRQl2hhsi							anonymous	EN	
2023-03-02 15:5	2023-03-02 16:0	IP Address	100	211	TRUE	2023-03-02 16:0	R_ONdbP2w4utjq9cR							anonymous	EN	
2023-03-02 16:0	2023-03-02 16:0	IP Address	100	33	TRUE	2023-03-02 16:0	R_2v05lEuulcA4iwo							anonymous	EN	
2023-03-02 16:1	2023-03-02 16:2	IP Address	100	475	TRUE	2023-03-02 16:2	R_3LirrnrQYlTPXcD							anonymous	EN	
2023-03-02 16:2	2023-03-02 16:2	IP Address	100	49	TRUE	2023-03-02 16:2	R_1n8QWAgUKoLEOXP							anonymous	EN	
2023-03-02 16:2	2023-03-02 16:2	IP Address	100	139	TRUE	2023-03-02 16:2	R_1Oje5ioqkRPNFle							anonymous	EN	
2023-03-02 16:2	2023-03-02 16:3	IP Address	100	397	TRUE	2023-03-02 16:3	R_1FmjiOZyy5Nw1Pn							anonymous	EN	
2023-03-02 16:3	2023-03-02 16:5	IP Address	100	1539	TRUE	2023-03-02 16:5	R_3Rmf79SxLI0MdHb							anonymous	EN	
2023-03-03 9:11	2023-03-03 9:19	IP Address	100	483	TRUE	2023-03-03 9:19	R_3qByadSPC0OrXmD							anonymous	EN	
2023-03-03 9:19	2023-03-03 11:3	IP Address	100	8365	TRUE	2023-03-03 11:3	R_2y9GpQRx0UP1msp							anonymous	EN	
2023-03-03 11:3	2023-03-03 13:0	IP Address	100	5382	TRUE	2023-03-03 13:0	R_yrpzjxZOiIUGbO9							anonymous	EN	
2023-03-03 15:2	2023-03-03 15:2	IP Address	100	191	TRUE	2023-03-03 15:2	R_3CWpPm4G52JoVZD							anonymous	EN	
2023-03-03 17:2	2023-03-03 17:2	IP Address	100	153	TRUE	2023-03-03 17:2	R_1OkTIC8CZSJXVFY							anonymous	EN	
2023-03-03 17:2	2023-03-03 17:2	IP Address	100	251	TRUE	2023-03-03 17:2	R_3pbalvjCFAceigA							anonymous	EN	
2023-03-03 18:1	2023-03-03 18:1	IP Address	100	221	TRUE	2023-03-03 18:1	R_u8jruOJAYuaXxRv							anonymous	EN	
2023-03-03 18:4	2023-03-03 18:4	IP Address	100	19	TRUE	2023-03-03 18:4	R_1gcFsni1yYS3OkI							anonymous	EN	
2023-03-03 19:0	2023-03-05 18:3	IP Address	100	170888	TRUE	2023-03-05 18:3	R_33jdDEAhojKsKvY							anonymous	EN	
2023-03-06 3:43	2023-03-06 4:02	IP Address	100	1168	TRUE	2023-03-06 4:02	R_4PFihtSYdwn9w2t							anonymous	EN	
2023-03-06 4:02	2023-03-06 4:03	IP Address	100	35	TRUE	2023-03-06 4:03	R_3M3zk1teENAjgcV							anonymous	EN	
2023-03-06 4:03	2023-03-06 4:06	IP Address	100	178	TRUE	2023-03-06 4:06	R_9Rk12bKZnTBeBb3							anonymous	EN	
2023-03-06 4:06	2023-03-06 4:08	IP Address	100	135	TRUE	2023-03-06 4:08	R_9N5ZM7skfVMDxeN							anonymous	EN	
2023-03-06 4:09	2023-03-06 4:36	IP Address	100	1671	TRUE	2023-03-06 4:36	R_5unn5ns5Nf767lL							anonymous	EN	
2023-03-06 4:38	2023-03-06 4:39	IP Address	100	69	TRUE	2023-03-06 4:39	R_2f1CkHV0TkffN49							anonymous	EN	
2023-03-06 4:39	2023-03-06 4:42	IP Address	100	193	TRUE	2023-03-06 4:42	R_1gdqsBIlvmecCG4							anonymous	EN	
2023-03-06 4:42	2023-03-06 4:49	IP Address	100	384	TRUE	2023-03-06 4:49	R_DGF6McrSgmnyVZT							anonymous	EN	
2023-03-06 4:49	2023-03-06 5:05	IP Address	100	990	TRUE	2023-03-06 5:05	R_vphNb5GoNgZwp8t							anonymous	EN	
2023-03-06 5:05	2023-03-06 5:10	IP Address	100	280	TRUE	2023-03-06 5:10	R_3R49HShO4SxBwAl							anonymous	EN	
2023-03-06 5:10	2023-03-06 5:13	IP Address	100	172	TRUE	2023-03-06 5:13	R_2sYAAsDnDOlvuSl							anonymous	EN	
2023-03-06 5:13	2023-03-06 5:16	IP Address	100	184	TRUE	2023-03-06 5:16	R_3PnVJbs3DfY81OP							anonymous	EN	
2023-03-06 5:16	2023-03-06 5:19	IP Address	100	139	TRUE	2023-03-06 5:19	R_2tgr2OI4BkhUXvF							anonymous	EN	
2023-03-06 5:20	2023-03-06 5:22	IP Address	100	96	TRUE	2023-03-06 5:22	R_x45W6sAMnESoAaB							anonymous	EN	
2023-03-06 5:22	2023-03-06 5:40	IP Address	100	1047	TRUE	2023-03-06 5:40	R_00xR9pwNm5MLwS5							anonymous	EN	
2023-03-06 6:38	2023-03-06 6:39	IP Address	100	61	TRUE	2023-03-06 6:39	R_2rBEhWnFGrAog8l							anonymous	EN	
2023-03-06 6:43	2023-03-06 7:27	IP Address	100	2630	TRUE	2023-03-06 7:27	R_Rxz9CZJjkoQeFr3							anonymous	EN	
2023-03-07 6:13	2023-03-07 6:14	IP Address	100	40	TRUE	2023-03-07 6:14	R_322UqQcygjs39JQ							anonymous	EN	
2023-03-07 6:14	2023-03-07 6:24	IP Address	100	596	TRUE	2023-03-07 6:24	R_0ixq6iWdvvdrFXX							anonymous	EN	
2023-03-07 6:24	2023-03-07 6:30	IP Address	100	348	TRUE	2023-03-07 6:30	R_3r2rF6qNan3DODj							anonymous	EN	
2023-03-07 6:30	2023-03-07 6:41	IP Address	100	641	TRUE	2023-03-07 6:41	R_3NUtZRQoDT1kY2J							anonymous	EN	
2023-03-07 17:4	2023-03-07 17:4	IP Address	100	106	TRUE	2023-03-07 17:4	R_TuDDxBrckfAifuN							anonymous	EN	
2023-03-02 23:0	2023-03-09 23:1	IP Address	100	605862	TRUE	2023-03-09 23:1	R_1hK02yYLYRlt1n3							anonymous	EN	
2023-03-09 23:1	2023-03-09 23:2	IP Address	100	81	TRUE	2023-03-09 23:2	R_2VshPItuNgkV1FK							anonymous	EN	
2023-03-10 16:4	2023-03-10 17:1	IP Address	100	1947	TRUE	2023-03-10 17:1	R_1nTGYxQ2WmpUyH4							anonymous	EN	
2023-03-10 17:3	2023-03-10 17:3	IP Address	100	15	TRUE	2023-03-10 17:3	R_1lzqcDNlYQ9tIMy							anonymous	EN	
2023-03-10 17:3	2023-03-10 17:3	IP Address	100	14	TRUE	2023-03-10 17:3	R_8hON3UnsFr5huCJ							anonymous	EN	
2023-03-10 17:2	2023-03-10 18:4	IP Address	100	4588	TRUE	2023-03-10 18:4	R_5coL4wuHOqfebS1							anonymous	EN	
2023-03-10 18:4	2023-03-10 18:5	IP Address	100	572	TRUE	2023-03-10 18:5	R_2dRdLJZ4yOlSnC5							anonymous	EN	
2023-03-10 18:5	2023-03-10 18:5	IP Address	100	52	TRUE	2023-03-10 18:5	R_2Yrj2MmEJDUb8eL							anonymous	EN	
2023-03-10 18:5	2023-03-10 19:0	IP Address	100	549	TRUE	2023-03-10 19:0	R_3n7yzl0xtXMMX8k							anonymous	EN	
2023-03-10 19:0	2023-03-10 19:1	IP Address	100	444	TRUE	2023-03-10 19:1	R_1l4y1cC0x7l0lxF							anonymous	EN	
2023-03-10 19:1	2023-03-10 19:3	IP Address	100	1358	TRUE	2023-03-10 19:3	R_2TueUVpRX9lzzRL							anonymous	EN	
2023-03-10 19:3	2023-03-10 19:3	IP Address	100	209	TRUE	2023-03-10 19:3	R_b9HMmfbcD6ufNyp							anonymous	EN	
2023-03-11 12:4	2023-03-11 13:0	IP Address	100	1051	TRUE	2023-03-11 13:0	R_2ZJRqGS95zZeUUZ							anonymous	EN	
2023-03-11 13:0	2023-03-11 13:0	IP Address	100	192	TRUE	2023-03-11 13:0	R_29iaME273pJMnU0							anonymous	EN	
2023-03-11 13:0	2023-03-11 13:1	IP Address	100	349	TRUE	2023-03-11 13:1	R_2R3tyQkGhbzpzUy							anonymous	EN	
2023-03-11 13:1	2023-03-11 13:2	IP Address	100	539	TRUE	2023-03-11 13:2	R_7VXVwZKN8LxQLjr							anonymous	EN	
2023-03-11 13:2	2023-03-11 13:2	IP Address	100	467	TRUE	2023-03-11 13:2	R_33d22XaTjlNviop							anonymous	EN	

2023-03-11 13:2	2023-03-11 13:3	IP Address	100	166	TRUE	2023-03-11 13:3	R_2PvPJoLQ1oEn8TR							anonymous	EN	
2023-03-11 13:3	2023-03-11 13:3	IP Address	100	145	TRUE	2023-03-11 13:3	R_2ckKFgU3nhBgMRx							anonymous	EN	
2023-03-11 14:2	2023-03-11 14:2	IP Address	100	16	TRUE	2023-03-11 14:2	R_27BtxglbXuMvpK2							anonymous	EN	
2023-03-12 6:29:	2023-03-12 6:36:	IP Address	100	418	TRUE	2023-03-12 6:36:	R_sUsfR97Lf0M215n							anonymous	EN	
2023-03-12 6:36:	2023-03-12 6:56:	IP Address	100	1193	TRUE	2023-03-12 6:56:	R_cTOrAIeWzj4yomZ							anonymous	EN	
2023-03-12 6:56:	2023-03-12 7:00:	IP Address	100	240	TRUE	2023-03-12 7:00:	R_uyNAqxqb0oVvhxD							anonymous	EN	
2023-03-12 7:00:	2023-03-12 7:03:	IP Address	100	161	TRUE	2023-03-12 7:03:	R_1kFvbLgO80KfIgq							anonymous	EN	
2023-03-12 7:03:	2023-03-12 7:07:	IP Address	100	292	TRUE	2023-03-12 7:07:	R_1Ne59R59LfGnyVp							anonymous	EN	
2023-03-12 7:08:	2023-03-12 7:13:	IP Address	100	299	TRUE	2023-03-12 7:13:	R_3R4STz6PdLaoIuA							anonymous	EN	
2023-03-12 11:5	2023-03-12 11:5	IP Address	100	227	TRUE	2023-03-12 11:5	R_1iICAnIjm24j9Ow							anonymous	EN	
2023-03-12 11:5	2023-03-12 12:0	IP Address	100	410	TRUE	2023-03-12 12:0	R_1OJ3WUriyoQ4ZH4							anonymous	EN	
2023-03-12 12:0	2023-03-12 12:0	IP Address	100	154	TRUE	2023-03-12 12:0	R_1jy3GOzLAbrDS2d							anonymous	EN	
2023-03-12 12:0	2023-03-12 12:1	IP Address	100	265	TRUE	2023-03-12 12:1	R_3nOVBa4FIT4akXO							anonymous	EN	
2023-03-12 12:1	2023-03-12 12:1	IP Address	100	133	TRUE	2023-03-12 12:1	R_2wuduImQlGJbMgz							anonymous	EN	
2023-03-12 12:1	2023-03-12 12:1	IP Address	100	78	TRUE	2023-03-12 12:1	R_2SGpU8pt99uk7zo							anonymous	EN	
2023-03-12 12:1	2023-03-12 12:1	IP Address	100	178	TRUE	2023-03-12 12:1	R_rfjq5yPswRCh1y9							anonymous	EN	
2023-03-12 12:1	2023-03-12 12:2	IP Address	100	264	TRUE	2023-03-12 12:2	R_1r0l6WhbcDfHnXb							anonymous	EN	
2023-03-12 12:2	2023-03-12 12:2	IP Address	100	175	TRUE	2023-03-12 12:2	R_12MbuA3iLj05PgS							anonymous	EN	
2023-03-12 12:2	2023-03-12 12:2	IP Address	100	197	TRUE	2023-03-12 12:2	R_2doUiDxt9uasrXV							anonymous	EN	
2023-03-09 23:2	2023-03-12 22:2	IP Address	100	252340	TRUE	2023-03-12 22:2	R_25HGQZZofYE1yY0							anonymous	EN	
2023-03-12 22:2	2023-03-12 22:3	IP Address	100	572	TRUE	2023-03-12 22:3	R_3FUHRSr3rCKTTZz							anonymous	EN	
2023-03-12 22:3	2023-03-13 0:00:	IP Address	100	5024	TRUE	2023-03-13 0:00:	R_YYMvgy4k32AoDRf							anonymous	EN	
2023-03-13 5:22:	2023-03-13 5:36:	IP Address	100	876	TRUE	2023-03-13 5:36:	R_3suiAlDgPXS5eZh							anonymous	EN	
2023-03-13 5:37:	2023-03-13 5:39:	IP Address	100	153	TRUE	2023-03-13 5:39:	R_1FQ9h7Dw7NI5Z6n							anonymous	EN	
2023-03-13 5:39:	2023-03-13 5:43:	IP Address	100	210	TRUE	2023-03-13 5:43:	R_29mErlPw9wlfyry							anonymous	EN	
2023-03-13 5:43:	2023-03-13 5:44:	IP Address	100	56	TRUE	2023-03-13 5:44:	R_40gLt4w1YU4TTUd							anonymous	EN	
2023-03-13 5:58:	2023-03-13 5:58:	IP Address	100	34	TRUE	2023-03-13 5:58:	R_3J4qNvXRlgQnssJ							anonymous	EN	
2023-03-13 5:58:	2023-03-13 6:09:	IP Address	100	620	TRUE	2023-03-13 6:09:	R_ujIwGYHrn35zTTX							anonymous	EN	
2023-03-13 6:09:	2023-03-13 6:10:	IP Address	100	66	TRUE	2023-03-13 6:10:	R_1C7p9QTbfc3TKs8							anonymous	EN	
2023-03-13 6:10:	2023-03-13 6:13:	IP Address	100	169	TRUE	2023-03-13 6:13:	R_r72ZqcxNz2djRyV							anonymous	EN	
2023-03-13 6:13:	2023-03-13 6:36:	IP Address	100	1415	TRUE	2023-03-13 6:36:	R_2zUDxEznc5fNuOl							anonymous	EN	
2023-03-13 6:36:	2023-03-13 6:39:	IP Address	100	160	TRUE	2023-03-13 6:39:	R_3G9yTX6fvCWKBzq							anonymous	EN	
2023-03-13 6:39:	2023-03-13 6:43:	IP Address	100	238	TRUE	2023-03-13 6:43:	R_3fpEH45Pqkat01V							anonymous	EN	
2023-03-14 19:1	2023-03-14 19:4	IP Address	100	1987	TRUE	2023-03-14 19:4	R_2AWmJgxGbQ8c22K							anonymous	EN	
2023-03-14 19:5	2023-03-14 19:5	IP Address	100	39	TRUE	2023-03-14 19:5	R_1pGvPLxKPm791J0							anonymous	EN	
2023-03-14 20:0	2023-03-14 20:0	IP Address	100	43	TRUE	2023-03-14 20:0	R_ezXqyk8akT3ae5j							anonymous	EN	
2023-03-14 20:0	2023-03-14 20:2	IP Address	100	901	TRUE	2023-03-14 20:2	R_2Yxkc3MxpH5tNDc							anonymous	EN	
2023-03-14 20:3	2023-03-14 20:3	IP Address	100	57	TRUE	2023-03-14 20:3	R_25KNGqVbq6vXB9x							anonymous	EN	
2023-03-13 0:00:	2023-03-14 23:1	IP Address	100	170022	TRUE	2023-03-14 23:1	R_etaudEPtmqL4yXv							anonymous	EN	
2023-03-14 23:1	2023-03-14 23:1	IP Address	100	165	TRUE	2023-03-14 23:1	R_zTJOtLhlZJEutAB							anonymous	EN	
2023-03-14 23:1	2023-03-14 23:2	IP Address	100	402	TRUE	2023-03-14 23:2	R_31WnSJAlxTGyKqi							anonymous	EN	
2023-03-15 19:2	2023-03-15 19:3	IP Address	100	410	TRUE	2023-03-15 19:3	R_2Qt8DXh97fqvz1n							anonymous	EN	
2023-03-15 19:3	2023-03-15 19:4	IP Address	100	285	TRUE	2023-03-15 19:4	R_3Rl1Ves6iVw5a19							anonymous	EN	
2023-03-15 19:4	2023-03-15 19:4	IP Address	100	32	TRUE	2023-03-15 19:4	R_1nNhqKLXsm8U4st							anonymous	EN	
2023-03-15 19:4	2023-03-15 19:4	IP Address	100	109	TRUE	2023-03-15 19:4	R_eeXBLlm1lZ6zdwB							anonymous	EN	
2023-03-15 19:4	2023-03-15 19:4	IP Address	100	166	TRUE	2023-03-15 19:4	R_2PhsB4tyCjSQ1Te							anonymous	EN	
2023-03-11 14:3	2023-03-11 14:3	IP Address	83	17	FALSE	2023-03-18 15:3	R_3HOKVMvIG42ri8m							anonymous	EN	
2023-03-18 19:3	2023-03-18 19:4	IP Address	100	587	TRUE	2023-03-18 19:4	R_0Ai72yHneRrR3oZ							anonymous	EN	
2023-03-18 19:4	2023-03-18 19:5	IP Address	100	638	TRUE	2023-03-18 19:5	R_1BQaENZanw91dlB							anonymous	EN	
2023-03-18 19:5	2023-03-18 19:5	IP Address	100	418	TRUE	2023-03-18 19:5	R_3NDREG4ko6vI5L6							anonymous	EN	
2023-03-19 17:5	2023-03-19 18:0	IP Address	100	564	TRUE	2023-03-19 18:0	R_2ZJpUYwOgA514gN							anonymous	EN	
2023-03-19 18:2	2023-03-19 18:2	IP Address	100	179	TRUE	2023-03-19 18:2	R_3PdyxmhdBHrxddr							anonymous	EN	
2023-03-19 18:2	2023-03-19 18:2	IP Address	100	95	TRUE	2023-03-19 18:2	R_ToTxhF1MrSW1t61							anonymous	EN	
2023-03-19 18:2	2023-03-19 18:3	IP Address	100	654	TRUE	2023-03-19 18:3	R_3Gvn3Vl6BnWypmf							anonymous	EN	
2023-03-19 18:3	2023-03-19 19:1	IP Address	100	2369	TRUE	2023-03-19 19:1	R_1eVIUPQR3SMiduS							anonymous	EN	
2023-03-19 19:5	2023-03-19 20:1	IP Address	100	655	TRUE	2023-03-19 20:1	R_23Ucv8V4Z9qSQjA							anonymous	EN	
2023-03-21 5:24:	2023-03-21 5:28:	IP Address	100	227	TRUE	2023-03-21 5:28:	R_3VLn2xeu6spwNhf							anonymous	EN	
2023-03-21 5:28:	2023-03-21 5:29:	IP Address	100	109	TRUE	2023-03-21 5:29:	R_3JqL0AoxjOGtUrK							anonymous	EN	
2023-03-21 5:29:	2023-03-21 5:39:	IP Address	100	585	TRUE	2023-03-21 5:39:	R_10obEN5tozksj54							anonymous	EN	

2023-03-21 5:41:	2023-03-21 5:46:	IP Address	100	264	TRUE	2023-03-21 5:46:	R_1mme4UXmEjhLx93						anonymous	EN	
2023-03-21 15:09	2023-03-21 15:19	IP Address	100	629	TRUE	2023-03-21 15:19	R_3F8IpxK7FOONdtL						anonymous	EN	
2023-03-21 15:20	2023-03-21 15:22	IP Address	100	160	TRUE	2023-03-21 15:22	R_1pAIna7FFFpRWLQ						anonymous	EN	
2023-03-21 15:23	2023-03-21 15:24	IP Address	100	90	TRUE	2023-03-21 15:24	R_2xKJVJ3NxbTZXj3						anonymous	EN	
2023-03-21 15:25	2023-03-21 15:32	IP Address	100	432	TRUE	2023-03-21 15:32	R_29VR4t4TvZxwwdH						anonymous	EN	
2023-03-21 15:32	2023-03-21 15:36	IP Address	100	200	TRUE	2023-03-21 15:36	R_3Ic1NRyTzChy3f9						anonymous	EN	
2023-03-21 15:36	2023-03-21 15:37	IP Address	100	55	TRUE	2023-03-21 15:37	R_1QuUsq9asZ3lbjd						anonymous	EN	
2023-03-21 15:38	2023-03-21 15:47	IP Address	100	518	TRUE	2023-03-21 15:47	R_30egO5Ugp9Qoptk						anonymous	EN	
2023-03-21 15:48	2023-03-21 15:50	IP Address	100	145	TRUE	2023-03-21 15:50	R_DofCP2iQito53lD						anonymous	EN	
2023-03-21 15:50	2023-03-21 15:53	IP Address	100	186	TRUE	2023-03-21 15:53	R_3g5HEViNRRZen2h						anonymous	EN	
2023-03-21 15:53	2023-03-21 15:54	IP Address	100	37	TRUE	2023-03-21 15:54	R_USiTOX0CyACkKNH						anonymous	EN	
2023-03-21 15:54	2023-03-21 15:55	IP Address	100	58	TRUE	2023-03-21 15:55	R_A4CQg9yHrc8syn7						anonymous	EN	
2023-03-21 15:56	2023-03-21 15:56	IP Address	100	39	TRUE	2023-03-21 15:56	R_2YLh5Y7tQRtKetR						anonymous	EN	
2023-03-21 15:56	2023-03-21 15:57	IP Address	100	56	TRUE	2023-03-21 15:57	R_1gA25NSXanKfYA7						anonymous	EN	
2023-03-21 15:58	2023-03-21 16:00	IP Address	100	147	TRUE	2023-03-21 16:00	R_1NCWP7zyv616NLW						anonymous	EN	
2023-03-21 16:01	2023-03-21 16:01	IP Address	100	56	TRUE	2023-03-21 16:01	R_2E02XJkdKm3VmCk						anonymous	EN	
2023-03-21 16:02	2023-03-21 16:04	IP Address	100	175	TRUE	2023-03-21 16:04	R_3ezb0eEBxu8Ikfl						anonymous	EN	
2023-03-21 16:05	2023-03-21 16:08	IP Address	100	170	TRUE	2023-03-21 16:08	R_3ef6tCXVNaJG7SE						anonymous	EN	
2023-03-21 16:08	2023-03-21 16:09	IP Address	100	47	TRUE	2023-03-21 16:09	R_28MRqxMrQnAN7sR						anonymous	EN	
2023-03-21 16:09	2023-03-21 16:11	IP Address	100	127	TRUE	2023-03-21 16:11	R_3HGQpz82vDhxYvv						anonymous	EN	
2023-03-21 16:11	2023-03-21 16:14	IP Address	100	165	TRUE	2023-03-21 16:14	R_2cBFeureGDkBRuJ						anonymous	EN	
2023-03-21 16:14	2023-03-21 16:17	IP Address	100	194	TRUE	2023-03-21 16:17	R_3OenZyMqAbbk9ET						anonymous	EN	
2023-03-21 16:20	2023-03-21 16:21	IP Address	100	64	TRUE	2023-03-21 16:21	R_2YX3Rtz4UwSEOto						anonymous	EN	
2023-03-21 16:22	2023-03-21 16:24	IP Address	100	156	TRUE	2023-03-21 16:24	R_qPCMkJBpnEq1jqh						anonymous	EN	
2023-03-21 16:29	2023-03-21 16:34	IP Address	100	286	TRUE	2023-03-21 16:34	R_3NwX28o93xvmWf3						anonymous	EN	
2023-03-21 16:34	2023-03-21 16:37	IP Address	100	153	TRUE	2023-03-21 16:37	R_2qxxQ1xcX0Ai3Dw						anonymous	EN	
2023-03-21 16:37	2023-03-21 16:39	IP Address	100	105	TRUE	2023-03-21 16:39	R_3JyqE3qtfuDfgXR						anonymous	EN	
2023-03-21 16:40	2023-03-21 16:43	IP Address	100	196	TRUE	2023-03-21 16:43	R_1IB5h54sddQSdmR						anonymous	EN	
2023-03-21 16:20	2023-03-21 16:28	IP Address	100	160	TRUE	2023-03-21 16:44	R_1dgI8pSfxhQUoG7						anonymous	EN	
2023-03-21 16:44	2023-03-21 16:45	IP Address	100	87	TRUE	2023-03-21 16:45	R_BR10OsMq6OqZGg1						anonymous	EN	
2023-03-21 16:45	2023-03-21 16:50	IP Address	100	286	TRUE	2023-03-21 16:50	R_3RrcGxJdcX078jG						anonymous	EN	
2023-03-21 16:51	2023-03-21 16:52	IP Address	100	108	TRUE	2023-03-21 16:52	R_1r32kgadfiz23ki						anonymous	EN	
2023-03-21 16:53	2023-03-21 16:56	IP Address	100	186	TRUE	2023-03-21 16:56	R_10xzXrxGJT23uKz						anonymous	EN	
2023-03-21 16:57	2023-03-21 16:58	IP Address	100	103	TRUE	2023-03-21 16:58	R_3O8hbnMVhElyhL5						anonymous	EN	
2023-03-21 17:02	2023-03-21 17:04	IP Address	100	111	TRUE	2023-03-21 17:04	R_2tzpTnBd4pQfLvQ						anonymous	EN	
2023-03-21 17:05	2023-03-21 17:08	IP Address	100	164	TRUE	2023-03-21 17:08	R_3pgadgybL0PMjGy						anonymous	EN	
2023-03-21 17:10	2023-03-21 17:12	IP Address	100	113	TRUE	2023-03-21 17:12	R_2CZEFGKceTtB0J9						anonymous	EN	
2023-03-21 17:12	2023-03-21 17:14	IP Address	100	154	TRUE	2023-03-21 17:14	R_2ATN1MI6uXFLlLw						anonymous	EN	
2023-03-21 17:15	2023-03-21 17:17	IP Address	100	114	TRUE	2023-03-21 17:17	R_27p1CUmJMPO3ss1						anonymous	EN	
2023-03-21 17:18	2023-03-21 17:19	IP Address	100	89	TRUE	2023-03-21 17:19	R_3h3c4IBjpqJKLTV						anonymous	EN	
2023-03-22 14:02	2023-03-22 14:03	IP Address	100	82	TRUE	2023-03-22 14:03	R_2ZR0X5tzO22zk5a						anonymous	EN	
2023-03-22 14:04	2023-03-22 14:11	IP Address	100	415	TRUE	2023-03-22 14:11	R_qwmEAMt51o3wG7T						anonymous	EN	
2023-03-22 14:11	2023-03-22 14:25	IP Address	100	807	TRUE	2023-03-22 14:25	R_7a0W5XpTdNNxquJ						anonymous	EN	
2023-03-22 14:25	2023-03-22 14:25	IP Address	100	44	TRUE	2023-03-22 14:25	R_2dEDu3W5fxgvrdX						anonymous	EN	
2023-03-22 14:25	2023-03-22 14:27	IP Address	100	108	TRUE	2023-03-22 14:27	R_2vhP5S3fQyA6JSf						anonymous	EN	
2023-03-22 16:34	2023-03-22 16:51	IP Address	100	1049	TRUE	2023-03-22 16:51	R_239RuMxTz7a3Whw						anonymous	EN	
2023-03-22 21:40	2023-03-22 21:48	IP Address	100	65	TRUE	2023-03-22 21:48	R_21ahlYXW1Vq5YLQ						anonymous	EN	
2023-03-14 23:23	2023-03-16 3:21:	IP Address	83	100654	FALSE	2023-03-23 3:21:	R_3hH0kNZ5W4jlCYI						anonymous	EN	
2023-03-23 17:57	2023-03-23 17:57	IP Address	100	18	TRUE	2023-03-23 17:57	R_2rpxcKtXBlPXXWi						anonymous	EN	
2023-03-24 10:47	2023-03-24 10:56	IP Address	100	550	TRUE	2023-03-24 10:56	R_30v0dpkEmsYMTO8						anonymous	EN	
2023-03-24 10:56	2023-03-24 10:57	IP Address	100	57	TRUE	2023-03-24 10:57	R_24kjzmawLzESeDz						anonymous	EN	
2023-03-24 10:57	2023-03-24 11:00	IP Address	100	139	TRUE	2023-03-24 11:00	R_e3yJXHmwK06dnvH						anonymous	EN	
2023-03-24 11:00	2023-03-24 11:08	IP Address	100	517	TRUE	2023-03-24 11:08	R_3mkQPj5Ibbs1cC6						anonymous	EN	
2023-03-24 11:08	2023-03-24 11:11	IP Address	100	189	TRUE	2023-03-24 11:11	R_2WJ4ugNEG9SfXC1						anonymous	EN	
2023-03-24 11:11	2023-03-24 11:15	IP Address	100	200	TRUE	2023-03-24 11:15	R_3LbtJ7hUnAE44Yg						anonymous	EN	
2023-03-24 11:15	2023-03-24 11:16	IP Address	100	96	TRUE	2023-03-24 11:16	R_UY2YKwJa6BLlk4h						anonymous	EN	
2023-03-24 11:16	2023-03-24 11:17	IP Address	100	39	TRUE	2023-03-24 11:17	R_3fJMXuiYq40mV17						anonymous	EN	
2023-03-24 11:17	2023-03-24 11:22	IP Address	100	276	TRUE	2023-03-24 11:22	R_3lKqJNLXtvEfpI9						anonymous	EN	
2023-03-24 11:22	2023-03-24 11:27	IP Address	100	321	TRUE	2023-03-24 11:27	R_b4rnqTqeg1TKuhb						anonymous	EN	

2023-03-24 11:2	2023-03-24 11:3	IP Address	100	528	TRUE	2023-03-24 11:3	R_1PXmkte4nri0Msv							anonymous	EN	
2023-03-24 11:3	2023-03-24 11:4	IP Address	100	216	TRUE	2023-03-24 11:4	R_2EveJO4M4X4yN6m							anonymous	EN	
2023-03-24 11:4	2023-03-24 11:4	IP Address	100	248	TRUE	2023-03-24 11:4	R_3MhaaGvBVpzBhhJ							anonymous	EN	
2023-03-24 11:4	2023-03-24 11:4	IP Address	100	213	TRUE	2023-03-24 11:4	R_2aws0Ott7OH3o31							anonymous	EN	
2023-03-24 11:4	2023-03-24 11:5	IP Address	100	455	TRUE	2023-03-24 11:5	R_1Ibr1EVIS0S9EQc							anonymous	EN	
2023-03-24 12:1	2023-03-24 12:1	IP Address	100	14	TRUE	2023-03-24 12:1	R_1jxnPUdlvxVjieV							anonymous	EN	
2023-03-24 20:5	2023-03-24 21:1	IP Address	100	1047	TRUE	2023-03-24 21:1	R_bJbMzWJSfzzSulj							anonymous	EN	
2023-03-24 21:1	2023-03-24 21:5	IP Address	100	2090	TRUE	2023-03-24 21:5	R_294TIARz6tdDoxr							anonymous	EN	
2023-03-24 21:5	2023-03-24 21:5	IP Address	100	85	TRUE	2023-03-24 21:5	R_27fgPrZCpRSvxcm							anonymous	EN	
2023-03-23 21:5	2023-03-24 22:3	IP Address	100	89187	TRUE	2023-03-24 22:3	R_1OSIucxwmp60fdu							anonymous	EN	
2023-03-24 21:5	2023-03-24 22:3	IP Address	100	2737	TRUE	2023-03-24 22:3	R_3G1TnPXPD2NIs5I							anonymous	EN	
2023-03-24 22:3	2023-03-24 23:1	IP Address	100	2553	TRUE	2023-03-24 23:1	R_214cHUe4WpRDlCT							anonymous	EN	
2023-03-24 22:3	2023-03-25 0:00	IP Address	100	4963	TRUE	2023-03-25 0:00	R_21aBSYORWlggQPt							anonymous	EN	
2023-03-25 0:00	2023-03-25 3:02	IP Address	100	10918	TRUE	2023-03-25 3:02	R_2VyCy5m1I1LBCSx							anonymous	EN	
2023-03-25 3:02	2023-03-25 3:07	IP Address	100	298	TRUE	2023-03-25 3:07	R_3niQuGyfWkqIsdY							anonymous	EN	
2023-03-25 10:2	2023-03-25 10:3	IP Address	100	548	TRUE	2023-03-25 10:3	R_3h09FM0fidALIxU							anonymous	EN	
2023-03-25 10:3	2023-03-25 10:4	IP Address	100	335	TRUE	2023-03-25 10:4	R_WvO6PeDowOgYRfH							anonymous	EN	
2023-03-25 10:4	2023-03-25 10:5	IP Address	100	481	TRUE	2023-03-25 10:5	R_3LchzNhXaFH1ElP							anonymous	EN	
2023-03-25 10:5	2023-03-25 11:0	IP Address	100	259	TRUE	2023-03-25 11:0	R_3dEvT5FoHlB9k6y							anonymous	EN	
2023-03-25 11:0	2023-03-25 11:0	IP Address	100	190	TRUE	2023-03-25 11:0	R_8HBiWIGnq2CeO09							anonymous	EN	
2023-03-25 11:0	2023-03-25 11:1	IP Address	100	213	TRUE	2023-03-25 11:1	R_1QEC3fKbKlKkzYo							anonymous	EN	
2023-03-25 11:1	2023-03-25 11:1	IP Address	100	211	TRUE	2023-03-25 11:1	R_2ZUqJhuwdpOBlng							anonymous	EN	
2023-03-25 11:2	2023-03-25 11:2	IP Address	100	192	TRUE	2023-03-25 11:2	R_20U7QwZN8tcAbNN							anonymous	EN	
2023-03-25 11:3	2023-03-25 11:3	IP Address	100	205	TRUE	2023-03-25 11:3	R_3kAKqNewPLTpohx							anonymous	EN	
2023-03-25 11:3	2023-03-25 11:4	IP Address	100	214	TRUE	2023-03-25 11:4	R_31oBBrC1iho7PCM							anonymous	EN	
2023-03-25 11:4	2023-03-25 11:4	IP Address	100	193	TRUE	2023-03-25 11:4	R_3dRZn4f6PnjnosP							anonymous	EN	
2023-03-25 0:22	2023-03-25 12:3	IP Address	100	43899	TRUE	2023-03-25 12:3	R_tFBxWTjUXRcww8N							anonymous	EN	
2023-03-25 11:5	2023-03-25 12:3	IP Address	100	2580	TRUE	2023-03-25 12:3	R_1eRFYESzauItQmk							anonymous	EN	
2023-03-25 12:3	2023-03-25 12:3	IP Address	100	276	TRUE	2023-03-25 12:3	R_31bxT5Z14cikxT2							anonymous	EN	
2023-03-26 17:4	2023-03-26 18:0	IP Address	100	1021	TRUE	2023-03-26 18:0	R_31pwFaWq48szCo6							anonymous	EN	
2023-03-26 18:0	2023-03-26 18:2	IP Address	100	1124	TRUE	2023-03-26 18:2	R_dgyirbvxASusmCR							anonymous	EN	
2023-03-26 18:2	2023-03-26 18:3	IP Address	100	570	TRUE	2023-03-26 18:3	R_2qxyLkNyxerIYRd							anonymous	EN	
2023-03-26 19:4	2023-03-26 19:5	IP Address	100	484	TRUE	2023-03-26 19:5	R_2sTuMIWuCfPQOvV							anonymous	EN	
2023-03-26 19:5	2023-03-26 19:5	IP Address	100	152	TRUE	2023-03-26 19:5	R_UXdoRL6HZgIEHiV							anonymous	EN	
2023-03-27 8:29	2023-03-27 8:36	IP Address	100	397	TRUE	2023-03-27 8:36	R_3MPyMy9uPvkhLbV							anonymous	EN	
2023-03-27 8:36	2023-03-27 8:47	IP Address	100	655	TRUE	2023-03-27 8:47	R_eQGrlOVfYNK0gVP							anonymous	EN	
2023-03-27 8:47	2023-03-27 8:48	IP Address	100	68	TRUE	2023-03-27 8:48	R_22tpTaED7Hv7E4o							anonymous	EN	
2023-03-27 8:48	2023-03-27 8:48	IP Address	100	44	TRUE	2023-03-27 8:48	R_2zi7kRwryYa1fKP							anonymous	EN	
2023-03-27 8:49	2023-03-27 8:52	IP Address	100	182	TRUE	2023-03-27 8:52	R_3qiGkj4pz7UFaYV							anonymous	EN	
2023-03-27 8:52	2023-03-27 8:59	IP Address	100	420	TRUE	2023-03-27 8:59	R_2uUtnclbwPlEm7e							anonymous	EN	
2023-03-27 8:59	2023-03-27 9:06	IP Address	100	443	TRUE	2023-03-27 9:06	R_T1MdOEQKd0e64z7							anonymous	EN	
2023-03-27 9:06	2023-03-27 9:15	IP Address	100	551	TRUE	2023-03-27 9:15	R_2tzNN0l2VNUFs9E							anonymous	EN	
2023-03-27 9:15	2023-03-27 9:20	IP Address	100	284	TRUE	2023-03-27 9:20	R_3qJyPXR06TS7BiR							anonymous	EN	
2023-03-27 9:20	2023-03-27 9:22	IP Address	100	104	TRUE	2023-03-27 9:22	R_28ZkZOM6Tsae6iW							anonymous	EN	
2023-03-27 9:22	2023-03-27 9:24	IP Address	100	119	TRUE	2023-03-27 9:24	R_3jTDKz1nXJivqiU							anonymous	EN	
2023-03-27 9:24	2023-03-27 9:26	IP Address	100	111	TRUE	2023-03-27 9:26	R_XyORl71QvrM6eJP							anonymous	EN	
2023-03-27 9:26	2023-03-27 9:30	IP Address	100	280	TRUE	2023-03-27 9:30	R_1JLa0hIPQiEwW3w							anonymous	EN	
2023-03-27 9:30	2023-03-27 9:33	IP Address	100	126	TRUE	2023-03-27 9:33	R_2dQgJYfTZgSyp6C							anonymous	EN	
2023-03-28 12:4	2023-03-28 12:4	IP Address	100	326	TRUE	2023-03-28 12:4	R_1IABs7I0vrHaRKk							anonymous	EN	
2023-03-28 13:1	2023-03-28 13:3	IP Address	100	1067	TRUE	2023-03-28 13:3	R_2By85w7MXmS9DId							anonymous	EN	
2023-03-28 13:3	2023-03-28 13:3	IP Address	100	34	TRUE	2023-03-28 13:3	R_1gBnw6wHJVSEhzB							anonymous	EN	
2023-03-28 14:0	2023-03-28 14:0	IP Address	100	44	TRUE	2023-03-28 14:0	R_2rGcSYuCLruwiyg							anonymous	EN	
2023-03-28 22:0	2023-03-28 22:0	IP Address	100	193	TRUE	2023-03-28 22:0	R_bNrZqN5fivG3lzb							anonymous	EN	
2023-03-28 22:0	2023-03-28 22:1	IP Address	100	469	TRUE	2023-03-28 22:1	R_1gRzv8hRMORbQb9							anonymous	EN	
2023-03-28 22:1	2023-03-28 23:2	IP Address	100	4422	TRUE	2023-03-28 23:2	R_2f3gYYhz5qBMQJ8							anonymous	EN	
2023-03-29 14:0	2023-03-29 14:1	IP Address	100	199	TRUE	2023-03-29 14:1	R_tXNs4lGHdOZbxND							anonymous	EN	
2023-03-29 14:1	2023-03-29 14:1	IP Address	100	12	TRUE	2023-03-29 14:1	R_3qgnL2QZjmajwiC							anonymous	EN	
2023-03-30 8:38	2023-03-30 8:52	IP Address	100	825	TRUE	2023-03-30 8:52	R_1gI8LaKT88r41Qo							anonymous	EN	
2023-03-30 8:52	2023-03-30 8:55	IP Address	100	201	TRUE	2023-03-30 8:55	R_3XlMz7csJdXTChP							anonymous	EN	

2023-03-30 8:55	2023-03-30 8:57	IP Address	100	107	TRUE	2023-03-30 8:57	R_6XpiWsQhRrTsOAh							anonymous	EN	
2023-03-30 8:57	2023-03-30 9:04	IP Address	100	448	TRUE	2023-03-30 9:04	R_12SicpjKYIv1eDK							anonymous	EN	
2023-03-30 9:04	2023-03-30 9:06	IP Address	100	88	TRUE	2023-03-30 9:06	R_3Myw3Oo4xxL2MiA							anonymous	EN	
2023-03-30 9:06	2023-03-30 9:12	IP Address	100	388	TRUE	2023-03-30 9:12	R_2QsWWQ1G1bmRt4H							anonymous	EN	
2023-03-30 9:12	2023-03-30 9:14	IP Address	100	88	TRUE	2023-03-30 9:14	R_1pL4HVM5buKD2HO							anonymous	EN	
2023-03-30 9:14	2023-03-30 9:15	IP Address	100	60	TRUE	2023-03-30 9:15	R_2DTAlAdC8sMAYM5							anonymous	EN	
2023-03-30 9:15	2023-03-30 9:18	IP Address	100	180	TRUE	2023-03-30 9:18	R_2pMHwQKXTyEKFK4							anonymous	EN	
2023-03-30 9:18	2023-03-30 9:19	IP Address	100	51	TRUE	2023-03-30 9:19	R_3HTSplmwhqQxjmd							anonymous	EN	
2023-03-30 9:19	2023-03-30 9:25	IP Address	100	364	TRUE	2023-03-30 9:25	R_3L2ea3cGIEcq2ot							anonymous	EN	
2023-03-30 9:25	2023-03-30 9:28	IP Address	100	200	TRUE	2023-03-30 9:28	R_1llPn6pINJ3GvbK							anonymous	EN	
2023-03-30 9:29	2023-03-30 9:34	IP Address	100	336	TRUE	2023-03-30 9:34	R_wZu2wryMtl1UIwx							anonymous	EN	
2023-03-30 9:34	2023-03-30 9:38	IP Address	100	236	TRUE	2023-03-30 9:38	R_yrkZYaZeyQroWf7							anonymous	EN	
2023-03-30 9:38	2023-03-30 9:40	IP Address	100	124	TRUE	2023-03-30 9:40	R_2QzdYSSHWsO5ysc							anonymous	EN	
2023-03-30 9:40	2023-03-30 9:43	IP Address	100	162	TRUE	2023-03-30 9:43	R_2bZw3TV86xbyQRl							anonymous	EN	
2023-03-30 9:43	2023-03-30 9:46	IP Address	100	148	TRUE	2023-03-30 9:46	R_3kh8LTw1LWpDkZ3							anonymous	EN	
2023-03-30 9:46	2023-03-30 9:47	IP Address	100	65	TRUE	2023-03-30 9:47	R_roPmwVNCn2Q6erL							anonymous	EN	
2023-03-30 9:47	2023-03-30 9:51	IP Address	100	255	TRUE	2023-03-30 9:51	R_ZghuYiXLbyoIOYh							anonymous	EN	
2023-03-30 9:51	2023-03-30 9:55	IP Address	100	194	TRUE	2023-03-30 9:55	R_d13O6EpF3mc0xjz							anonymous	EN	
2023-03-30 9:57	2023-03-30 10:0	IP Address	100	470	TRUE	2023-03-30 10:0	R_2SpYTs0svXmVRZ8							anonymous	EN	
2023-03-30 10:0	2023-03-30 10:0	IP Address	100	79	TRUE	2023-03-30 10:0	R_2rJx0EK3Iliatcl							anonymous	EN	
2023-03-28 23:2	2023-03-30 22:2	IP Address	100	169347	TRUE	2023-03-30 22:2	R_31z9p7OdPGITggf							anonymous	EN	
2023-03-30 22:4	2023-03-30 22:4	IP Address	100	261	TRUE	2023-03-30 22:4	R_3Rf5Me95UA8oAJ1							anonymous	EN	
2023-03-30 10:0	2023-03-31 5:29	IP Address	100	69703	TRUE	2023-03-31 5:29	R_2xLhhKBg0C0LpbU							anonymous	EN	
2023-03-31 5:29	2023-03-31 5:39	IP Address	100	591	TRUE	2023-03-31 5:39	R_1BSeTVcfmWiOSBO							anonymous	EN	
2023-03-31 5:40	2023-03-31 5:44	IP Address	100	239	TRUE	2023-03-31 5:44	R_xx5eceR6txvyXn3							anonymous	EN	
2023-03-31 5:44	2023-03-31 5:45	IP Address	100	45	TRUE	2023-03-31 5:45	R_3oBFdRr5zk0ZXj3							anonymous	EN	
2023-03-31 5:45	2023-03-31 5:55	IP Address	100	596	TRUE	2023-03-31 5:55	R_3iOv67ndPi1uTgx							anonymous	EN	
2023-03-31 5:55	2023-03-31 6:00	IP Address	100	320	TRUE	2023-03-31 6:00	R_1ozoGVwo8xTXvRY							anonymous	EN	
2023-03-31 6:00	2023-03-31 6:03	IP Address	100	150	TRUE	2023-03-31 6:03	R_1JEgab3ryPLZUqO							anonymous	EN	
2023-03-31 6:03	2023-03-31 6:09	IP Address	100	366	TRUE	2023-03-31 6:09	R_QaBa0rKHH0eJMcx							anonymous	EN	
2023-03-31 6:09	2023-03-31 6:10	IP Address	100	35	TRUE	2023-03-31 6:10	R_1n77dX4VjEp9fB1							anonymous	EN	
2023-03-31 6:10	2023-03-31 6:12	IP Address	100	122	TRUE	2023-03-31 6:12	R_VUVkg726OJnxXGx							anonymous	EN	
2023-03-31 6:12	2023-03-31 6:19	IP Address	100	448	TRUE	2023-03-31 6:19	R_UF72mD8bC5P8hNf							anonymous	EN	
2023-03-31 6:19	2023-03-31 6:24	IP Address	100	273	TRUE	2023-03-31 6:24	R_cTtO0PAsODXvyUh							anonymous	EN	
2023-03-31 6:24	2023-03-31 6:24	IP Address	100	45	TRUE	2023-03-31 6:24	R_ZknTxBiSTAZFoul							anonymous	EN	
2023-03-31 6:24	2023-03-31 6:25	IP Address	100	39	TRUE	2023-03-31 6:25	R_CaDVhQfOyQnlL8d							anonymous	EN	
2023-03-31 6:25	2023-03-31 6:32	IP Address	100	402	TRUE	2023-03-31 6:32	R_1pGQuGHV6Xq9pV5							anonymous	EN	
2023-03-31 6:32	2023-03-31 6:44	IP Address	100	732	TRUE	2023-03-31 6:44	R_3plEryPjnG9yKni							anonymous	EN	
2023-03-31 6:44	2023-03-31 6:48	IP Address	100	219	TRUE	2023-03-31 6:48	R_W6EuE055IelPNdv							anonymous	EN	
2023-03-31 6:48	2023-03-31 6:52	IP Address	100	277	TRUE	2023-03-31 6:52	R_2tsBeg3Y39rGAhK							anonymous	EN	
2023-03-31 6:52	2023-03-31 7:00	IP Address	100	470	TRUE	2023-03-31 7:00	R_veoLi445a43egFj							anonymous	EN	
2023-03-31 15:1	2023-03-31 22:5	IP Address	100	27669	TRUE	2023-03-31 22:5	R_6SCfFaCggSFWysN							anonymous	EN	
2023-03-31 22:5	2023-03-31 23:0	IP Address	100	157	TRUE	2023-03-31 23:0	R_2uQi2ho2Qh0ZuNa							anonymous	EN	
2023-03-25 3:07	2023-03-25 3:10	IP Address	83	224	FALSE	2023-04-01 3:10	R_3pmPhc2xLQFQUaX							anonymous	EN	
2023-04-01 4:56	2023-04-01 4:59	IP Address	100	140	TRUE	2023-04-01 4:59	R_2TuxcT5Ewj7YUYY							anonymous	EN	
2023-04-01 4:59	2023-04-01 5:02	IP Address	100	220	TRUE	2023-04-01 5:02	R_33fYOQXGVfJKzhf							anonymous	EN	
2023-04-01 5:02	2023-04-01 5:09	IP Address	100	395	TRUE	2023-04-01 5:09	R_2Evs0eEwv34RJYA							anonymous	EN	
2023-04-01 5:09	2023-04-01 5:12	IP Address	100	189	TRUE	2023-04-01 5:12	R_2tmKgsqNlXBwSyo							anonymous	EN	
2023-04-01 5:12	2023-04-01 5:14	IP Address	100	98	TRUE	2023-04-01 5:14	R_21p35THf3SmpdLu							anonymous	EN	
2023-04-01 5:14	2023-04-01 5:17	IP Address	100	178	TRUE	2023-04-01 5:17	R_2B2UbkqJlFSHg3W							anonymous	EN	
2023-04-01 5:17	2023-04-01 5:17	IP Address	100	38	TRUE	2023-04-01 5:17	R_2iejsh7k0BCDeQF							anonymous	EN	
2023-04-01 5:17	2023-04-01 5:22	IP Address	100	255	TRUE	2023-04-01 5:22	R_10OyaZ7oAdhEJ7l							anonymous	EN	
2023-04-01 5:22	2023-04-01 5:23	IP Address	100	95	TRUE	2023-04-01 5:23	R_27kGrHc5K6ynBsP							anonymous	EN	
2023-04-01 5:23	2023-04-01 5:24	IP Address	100	34	TRUE	2023-04-01 5:24	R_3Mmq5yGL23jQWPo							anonymous	EN	
2023-04-01 5:24	2023-04-01 5:27	IP Address	100	191	TRUE	2023-04-01 5:27	R_2QQKc6Eh7ckXr2v							anonymous	EN	
2023-04-01 5:27	2023-04-01 5:28	IP Address	100	40	TRUE	2023-04-01 5:28	R_eJ2DJ6kmWo2DuQ9							anonymous	EN	
2023-04-01 5:28	2023-04-01 5:30	IP Address	100	102	TRUE	2023-04-01 5:30	R_9Rl9ZvD6FDUkgkF							anonymous	EN	
2023-04-01 5:30	2023-04-01 5:35	IP Address	100	310	TRUE	2023-04-01 5:35	R_2duO36qLLCJmzaQ							anonymous	EN	
2023-04-01 5:35	2023-04-01 5:37	IP Address	100	116	TRUE	2023-04-01 5:37	R_1K3GNd9mEz7AIsX							anonymous	EN	

2023-04-01 5:37	2023-04-01 5:38	IP Address	100	87	TRUE	2023-04-01 5:38	R_bQUnWP1oY2VmLcZ							anonymous	EN	
2023-04-01 5:38	2023-04-01 5:45	IP Address	100	405	TRUE	2023-04-01 5:45	R_37R9cNOnQmpIUff							anonymous	EN	
2023-04-01 5:46	2023-04-01 5:49	IP Address	100	136	TRUE	2023-04-01 5:49	R_3saGHONQdmPX4iJ							anonymous	EN	
2023-04-01 5:49	2023-04-01 5:50	IP Address	100	89	TRUE	2023-04-01 5:50	R_Dc4g4HNTf8XG6bv							anonymous	EN	
2023-04-01 5:50	2023-04-01 5:51	IP Address	100	48	TRUE	2023-04-01 5:51	R_8oc5Rpzo5nG25z3							anonymous	EN	
2023-04-01 11:1	2023-04-01 11:1	IP Address	100	19	TRUE	2023-04-01 11:1	R_2DTa4jo5egX1xHl							anonymous	EN	
2023-04-01 5:51	2023-04-02 14:5	IP Address	100	118960	TRUE	2023-04-02 14:5	R_3RwPNouR3DombtM							anonymous	EN	
2023-04-02 14:5	2023-04-02 14:5	IP Address	100	174	TRUE	2023-04-02 14:5	R_r3lug6EutxZ5beF							anonymous	EN	
2023-04-02 14:5	2023-04-02 14:5	IP Address	100	110	TRUE	2023-04-02 14:5	R_2v2nmjnjGiAm00F							anonymous	EN	
2023-04-02 14:5	2023-04-02 15:0	IP Address	100	497	TRUE	2023-04-02 15:0	R_1Q476n5fCXWBkTH							anonymous	EN	
2023-04-02 15:0	2023-04-02 15:1	IP Address	100	447	TRUE	2023-04-02 15:1	R_1Os4KLchmIGZls4							anonymous	EN	
2023-04-02 15:1	2023-04-02 15:1	IP Address	100	76	TRUE	2023-04-02 15:1	R_1GVRuFf7FWdrQwD							anonymous	EN	
2023-04-02 15:1	2023-04-02 15:1	IP Address	100	76	TRUE	2023-04-02 15:1	R_1i21lc4BNqx364r							anonymous	EN	
2023-04-02 10:3	2023-04-02 23:2	IP Address	100	46227	TRUE	2023-04-02 23:2	R_1FJ09fuU5K6j43l							anonymous	EN	
2023-04-03 1:41	2023-04-03 1:47	IP Address	100	395	TRUE	2023-04-03 1:47	R_2EbH96mqLEsc5tu							anonymous	EN	
2023-04-03 1:58	2023-04-03 1:59	IP Address	100	41	TRUE	2023-04-03 1:59	R_29jgcvg0DyGwbpE							anonymous	EN	
2023-03-27 4:02	2023-03-27 4:12	IP Address	83	640	FALSE	2023-04-03 4:12	R_31iLRrwtbbufUmL							anonymous	EN	
2023-04-06 3:34	2023-04-06 3:38	IP Address	100	220	TRUE	2023-04-06 3:38	R_0AnJbKpFGY0cipb							anonymous	EN	
2023-04-06 15:0	2023-04-06 15:0	IP Address	100	187	TRUE	2023-04-06 15:0	R_1jHrwuam9ObjAq1							anonymous	EN	
2023-04-06 15:0	2023-04-06 15:0	IP Address	100	86	TRUE	2023-04-06 15:0	R_1labBKB93Mc1Rn3							anonymous	EN	
2023-04-06 15:0	2023-04-06 15:1	IP Address	100	117	TRUE	2023-04-06 15:1	R_QoVMIcp1ZaC6A13							anonymous	EN	
2023-04-06 15:1	2023-04-06 15:1	IP Address	100	342	TRUE	2023-04-06 15:1	R_3nUeWNYATa96kTx							anonymous	EN	
2023-04-06 15:1	2023-04-06 15:2	IP Address	100	239	TRUE	2023-04-06 15:2	R_3KBKQta7QJleUfP							anonymous	EN	
2023-04-06 15:2	2023-04-06 15:2	IP Address	100	262	TRUE	2023-04-06 15:2	R_1dLIjrjvBkOlXpq							anonymous	EN	
2023-04-06 15:2	2023-04-06 15:2	IP Address	100	282	TRUE	2023-04-06 15:2	R_3JlcKzk76y8DYzV							anonymous	EN	
2023-04-06 15:2	2023-04-06 15:3	IP Address	100	194	TRUE	2023-04-06 15:3	R_vIHRWKcwcA4FGyB							anonymous	EN	
2023-04-09 13:0	2023-04-09 13:0	IP Address	100	43	TRUE	2023-04-09 13:0	R_Zw5P3SQWuKHlDO1							anonymous	EN	
2023-04-09 13:0	2023-04-09 13:1	IP Address	100	212	TRUE	2023-04-09 13:1	R_umiadGKYXYgHIJ3							anonymous	EN	
2023-04-09 13:1	2023-04-09 13:1	IP Address	100	381	TRUE	2023-04-09 13:1	R_2AStWqWjxka6s9H							anonymous	EN	
2023-04-09 13:1	2023-04-09 13:2	IP Address	100	243	TRUE	2023-04-09 13:2	R_2BaMs9Ncg8pbPk9							anonymous	EN	
2023-04-09 13:2	2023-04-09 13:2	IP Address	100	55	TRUE	2023-04-09 13:2	R_1FnShxwAMcKtb7r							anonymous	EN	
2023-04-09 13:2	2023-04-09 13:3	IP Address	100	118	TRUE	2023-04-09 13:3	R_2dQugHMDhVEKA7R							anonymous	EN	
2023-04-10 15:3	2023-04-10 15:4	IP Address	100	465	TRUE	2023-04-10 15:4	R_2UVFfw994GXGS2G							anonymous	EN	
2023-04-10 15:4	2023-04-10 15:4	IP Address	100	183	TRUE	2023-04-10 15:4	R_25KLe3mXaNkiMhA							anonymous	EN	
2023-04-10 15:4	2023-04-10 15:4	IP Address	100	31	TRUE	2023-04-10 15:4	R_3QYoPfZnQwh0L8c							anonymous	EN	
2023-04-10 15:4	2023-04-10 15:5	IP Address	100	209	TRUE	2023-04-10 15:5	R_3EMDpWZOwPt72Rx							anonymous	EN	
2023-04-10 15:5	2023-04-10 15:5	IP Address	100	123	TRUE	2023-04-10 15:5	R_1gTub0faVDcOvwx							anonymous	EN	
2023-04-10 15:5	2023-04-11 14:5	IP Address	100	82810	TRUE	2023-04-11 14:5	R_21u3bD7zVd0kzqx							anonymous	EN	
2023-04-11 14:5	2023-04-11 14:5	IP Address	100	230	TRUE	2023-04-11 14:5	R_UEGYAjKzhJcKwiR							anonymous	EN	
2023-04-11 14:5	2023-04-11 15:0	IP Address	100	313	TRUE	2023-04-11 15:0	R_3ef3xnsibHeANPP							anonymous	EN	
2023-04-11 15:0	2023-04-11 15:1	IP Address	100	613	TRUE	2023-04-11 15:1	R_soimmHKDNtnEcFj							anonymous	EN	
2023-04-11 15:1	2023-04-11 15:1	IP Address	100	36	TRUE	2023-04-11 15:1	R_3elc6nF6ESeOIDy							anonymous	EN	
2023-04-11 15:1	2023-04-11 15:1	IP Address	100	169	TRUE	2023-04-11 15:1	R_3I9N0VBE2CsJ9C1							anonymous	EN	
2023-04-11 15:1	2023-04-11 15:1	IP Address	100	196	TRUE	2023-04-11 15:1	R_1M5Oaht7yLh80n0							anonymous	EN	
2023-04-11 15:1	2023-04-11 15:2	IP Address	100	466	TRUE	2023-04-11 15:2	R_1n2YGupgC4G8a4q							anonymous	EN	
2023-04-11 15:2	2023-04-11 15:3	IP Address	100	730	TRUE	2023-04-11 15:3	R_3JF5nU6gJjZSd7U							anonymous	EN	
2023-04-12 8:18	2023-04-12 8:25	IP Address	100	424	TRUE	2023-04-12 8:25	R_22WVpETMR2X3yOs							anonymous	EN	
2023-04-12 8:25	2023-04-12 8:37	IP Address	100	721	TRUE	2023-04-12 8:37	R_2AKk8hKgyMXclDt							anonymous	EN	
2023-04-12 8:37	2023-04-12 8:39	IP Address	100	113	TRUE	2023-04-12 8:39	R_56Yafs15gLRiV9f							anonymous	EN	
2023-04-12 8:39	2023-04-12 8:47	IP Address	100	466	TRUE	2023-04-12 8:47	R_9RFVqVjwUsAx3Il							anonymous	EN	
2023-04-12 12:1	2023-04-12 12:1	IP Address	100	119	TRUE	2023-04-12 12:1	R_25QLP40Ov4zlmLx							anonymous	EN	
2023-04-12 12:1	2023-04-12 12:1	IP Address	100	80	TRUE	2023-04-12 12:1	R_1rB6o7kFsyMen5n							anonymous	EN	
2023-04-12 12:1	2023-04-12 12:2	IP Address	100	206	TRUE	2023-04-12 12:2	R_Za5weAqLTBviyFb							anonymous	EN	
2023-04-12 12:2	2023-04-12 12:2	IP Address	100	64	TRUE	2023-04-12 12:2	R_XFnVY1iYeQuCj29							anonymous	EN	
2023-04-12 12:2	2023-04-12 12:2	IP Address	100	88	TRUE	2023-04-12 12:2	R_1JVhuOBApCgnMoG							anonymous	EN	
2023-04-12 12:2	2023-04-12 12:3	IP Address	100	559	TRUE	2023-04-12 12:3	R_2fwglxnjte3wXVQ							anonymous	EN	
2023-04-12 12:3	2023-04-12 12:3	IP Address	100	150	TRUE	2023-04-12 12:3	R_3mgAgqOqXOMfJ5s							anonymous	EN	
2023-04-12 12:3	2023-04-12 12:4	IP Address	100	657	TRUE	2023-04-12 12:4	R_3MfgC9B6SUcUDuO							anonymous	EN	
2023-04-12 12:4	2023-04-12 13:0	IP Address	100	794	TRUE	2023-04-12 13:0	R_3nB2W1BeQOYLSiJ							anonymous	EN	

2023-04-12 13:02	2023-04-12 13:04	IP Address	100	115	TRUE	2023-04-12 13:04	R_3GcSV3svKshpveb							anonymous	EN	
2023-04-12 13:04	2023-04-12 13:06	IP Address	100	98	TRUE	2023-04-12 13:06	R_8IABhf7z959Vplv							anonymous	EN	
2023-04-12 13:06	2023-04-12 13:12	IP Address	100	357	TRUE	2023-04-12 13:12	R_2V9kQGAfAwHO0Ls							anonymous	EN	
2023-04-12 13:12	2023-04-12 13:13	IP Address	100	83	TRUE	2023-04-12 13:13	R_1OVxFbxMXKIA7Z8							anonymous	EN	
2023-04-12 13:19	2023-04-12 13:20	IP Address	100	73	TRUE	2023-04-12 13:20	R_RWVGSLxc7FuQ9XP							anonymous	EN	
2023-04-12 13:20	2023-04-12 13:23	IP Address	100	164	TRUE	2023-04-12 13:23	R_2Yf9tXoZjBKW0YN							anonymous	EN	
2023-04-12 13:25	2023-04-12 13:37	IP Address	100	720	TRUE	2023-04-12 13:37	R_5irocMc73ipeAAp							anonymous	EN	
2023-04-12 13:37	2023-04-12 13:42	IP Address	100	293	TRUE	2023-04-12 13:42	R_1fdrUHnvO3S8vs2							anonymous	EN	
2023-04-12 13:42	2023-04-12 13:43	IP Address	100	50	TRUE	2023-04-12 13:43	R_wRkgwMfkX3BDPOx							anonymous	EN	
2023-04-12 13:43	2023-04-12 13:46	IP Address	100	161	TRUE	2023-04-12 13:46	R_1KqJ618u6k7v4GN							anonymous	EN	
2023-04-12 13:46	2023-04-12 13:50	IP Address	100	242	TRUE	2023-04-12 13:50	R_3GBbnpmgJPsdSrp							anonymous	EN	
2023-04-12 13:50	2023-04-12 13:53	IP Address	100	180	TRUE	2023-04-12 13:53	R_3LdJDGaWM5ekuW3							anonymous	EN	
2023-04-12 13:53	2023-04-12 14:01	IP Address	100	493	TRUE	2023-04-12 14:01	R_1pWoZVa8rVIP56x							anonymous	EN	
2023-04-12 14:01	2023-04-12 14:09	IP Address	100	472	TRUE	2023-04-12 14:09	R_Xplr93lVsNT9Gud							anonymous	EN	
2023-04-12 14:09	2023-04-12 14:16	IP Address	100	435	TRUE	2023-04-12 14:17	R_31mUuIVJ4LThqX6							anonymous	EN	
2023-04-12 14:22	2023-04-12 14:26	IP Address	100	280	TRUE	2023-04-12 14:26	R_1F3YhxLtds5rxne							anonymous	EN	
2023-04-12 14:29	2023-04-12 14:37	IP Address	100	465	TRUE	2023-04-12 14:37	R_1eyHlmyuAEIytOg							anonymous	EN	
2023-04-12 14:37	2023-04-12 14:42	IP Address	100	312	TRUE	2023-04-12 14:42	R_1GU2UD42oCYueXF							anonymous	EN	
2023-04-12 14:42	2023-04-12 14:44	IP Address	100	138	TRUE	2023-04-12 14:44	R_1E5UyHEzrOiC47P							anonymous	EN	
2023-04-12 14:44	2023-04-12 14:51	IP Address	100	389	TRUE	2023-04-12 14:51	R_1QG8Ct6PnoFmYYI							anonymous	EN	
2023-04-12 14:51	2023-04-12 15:01	IP Address	100	597	TRUE	2023-04-12 15:01	R_1K0CFTzJDXxExHt							anonymous	EN	
2023-04-13 10:32	2023-04-13 10:49	IP Address	100	1025	TRUE	2023-04-13 10:49	R_21ui0DDMdQaqsel							anonymous	EN	
2023-04-13 10:49	2023-04-13 11:00	IP Address	100	619	TRUE	2023-04-13 11:00	R_2xGo95CgAx9OmT0							anonymous	EN	
2023-04-13 11:00	2023-04-13 11:04	IP Address	100	247	TRUE	2023-04-13 11:04	R_3FJoD4nP17NDNuR							anonymous	EN	
2023-04-13 11:04	2023-04-13 11:06	IP Address	100	96	TRUE	2023-04-13 11:06	R_dj1xNje8W9kepgd							anonymous	EN	
2023-04-13 11:06	2023-04-13 11:15	IP Address	100	534	TRUE	2023-04-13 11:15	R_277LUZYkWgNvpDk							anonymous	EN	
2023-04-13 11:15	2023-04-13 11:16	IP Address	100	54	TRUE	2023-04-13 11:16	R_8BTkQjbKRDJTY8F							anonymous	EN	
2023-04-13 11:16	2023-04-13 11:23	IP Address	100	473	TRUE	2023-04-13 11:23	R_2EFyDdpLyLgIOGw							anonymous	EN	
2023-04-13 11:23	2023-04-13 11:30	IP Address	100	366	TRUE	2023-04-13 11:30	R_3HGLV7MDk0D8NKI							anonymous	EN	
2023-04-13 11:30	2023-04-13 11:34	IP Address	100	236	TRUE	2023-04-13 11:34	R_3sBK72lCibCuz9U							anonymous	EN	
2023-04-13 11:34	2023-04-13 11:37	IP Address	100	199	TRUE	2023-04-13 11:37	R_3nvkZbO7bSBuzGz							anonymous	EN	
2023-04-13 11:37	2023-04-13 11:38	IP Address	100	82	TRUE	2023-04-13 11:38	R_VKdYMNPcFpwROdr							anonymous	EN	
2023-04-13 11:38	2023-04-13 11:39	IP Address	100	43	TRUE	2023-04-13 11:39	R_332uQKWffTOkWfV							anonymous	EN	
2023-04-13 11:39	2023-04-13 11:40	IP Address	100	81	TRUE	2023-04-13 11:40	R_1gchO44MoLjf3aW							anonymous	EN	
2023-04-13 11:41	2023-04-13 11:56	IP Address	100	932	TRUE	2023-04-13 11:56	R_3NDQPxPNb8Ol5sn							anonymous	EN	
2023-04-13 11:56	2023-04-13 12:01	IP Address	100	276	TRUE	2023-04-13 12:01	R_1Q6KFXKSmXXduGz							anonymous	EN	
2023-04-13 12:01	2023-04-13 12:05	IP Address	100	256	TRUE	2023-04-13 12:05	R_3fPffz0DyaCAdVs							anonymous	EN	
2023-04-13 16:32	2023-04-13 17:22	IP Address	100	2976	TRUE	2023-04-13 17:22	R_3poqHCwPpTtcSPg							anonymous	EN	
2023-04-13 17:24	2023-04-13 17:25	IP Address	100	83	TRUE	2023-04-13 17:25	R_26mbkCuTkXRvuRe							anonymous	EN	
2023-04-13 17:25	2023-04-13 17:28	IP Address	100	167	TRUE	2023-04-13 17:28	R_prPokoZPOexlgzv							anonymous	EN	
2023-04-13 17:28	2023-04-13 17:31	IP Address	100	201	TRUE	2023-04-13 17:31	R_2yj4Np2SqwxXP8e							anonymous	EN	
2023-04-13 17:32	2023-04-13 18:41	IP Address	100	4131	TRUE	2023-04-13 18:41	R_vMgYJySSvmCUY81							anonymous	EN	
2023-04-14 3:58:	2023-04-14 4:04:	IP Address	100	309	TRUE	2023-04-14 4:04:	R_WePiTuGaob1r72h							anonymous	EN	
2023-04-14 4:04:	2023-04-14 4:06:	IP Address	100	144	TRUE	2023-04-14 4:06:	R_3psafWBcYdlhD08							anonymous	EN	
2023-04-14 4:06:	2023-04-14 4:08:	IP Address	100	106	TRUE	2023-04-14 4:08:	R_1Pd9OQkstMDknE2							anonymous	EN	
2023-04-14 4:08:	2023-04-14 4:11:	IP Address	100	163	TRUE	2023-04-14 4:11:	R_2uD1ij424XyRMCQ							anonymous	EN	
2023-04-14 4:11:	2023-04-14 4:12:	IP Address	100	107	TRUE	2023-04-14 4:12:	R_x4xueXiRE59CIaB							anonymous	EN	
2023-04-14 4:13:	2023-04-14 4:31:	IP Address	100	1091	TRUE	2023-04-14 4:31:	R_2Y5iEnjGcT4J7Ts							anonymous	EN	
2023-04-14 4:32:	2023-04-14 4:37:	IP Address	100	260	TRUE	2023-04-14 4:37:	R_tEEnJjyPFMZzdV7							anonymous	EN	
2023-04-14 4:37:	2023-04-14 4:53:	IP Address	100	962	TRUE	2023-04-14 4:53:	R_1rBJPo2gfvOw7Zr							anonymous	EN	
2023-04-14 4:53:	2023-04-14 4:55:	IP Address	100	119	TRUE	2023-04-14 4:55:	R_3iDuQobpXV6C3JT							anonymous	EN	
2023-04-14 4:55:	2023-04-14 5:07:	IP Address	100	718	TRUE	2023-04-14 5:07:	R_DMFA0kWM9xVnPUJ							anonymous	EN	
2023-04-14 5:07:	2023-04-14 5:09:	IP Address	100	102	TRUE	2023-04-14 5:09:	R_reRGJt5iGbuX3X3							anonymous	EN	
2023-04-14 5:09:	2023-04-14 5:19:	IP Address	100	600	TRUE	2023-04-14 5:19:	R_2AG2M3f26ijq39I							anonymous	EN	
2023-04-14 5:19:	2023-04-14 5:26:	IP Address	100	416	TRUE	2023-04-14 5:26:	R_1dsNo9xrWECb4BF							anonymous	EN	
2023-04-15 13:29	2023-04-15 13:43	IP Address	100	868	TRUE	2023-04-15 13:43	R_3O0MSTiaT1YzyGa							anonymous	EN	
2023-04-15 13:43	2023-04-15 14:03	IP Address	100	1199	TRUE	2023-04-15 14:03	R_3ExKrZzAwj2Hpfl							anonymous	EN	
2023-04-15 14:05	2023-04-15 14:06	IP Address	100	82	TRUE	2023-04-15 14:06	R_2OT18AvJCIm3Xva							anonymous	EN	
2023-04-15 14:06	2023-04-15 14:46	IP Address	100	2352	TRUE	2023-04-15 14:46	R_3hi10B1UPuy3dOW							anonymous	EN	

2023-04-16 6:14:	2023-04-16 6:19:	IP Address	100	271	TRUE	2023-04-16 6:19:	R_3JwnbR8LPCgJOFv						anonymous	EN	
2023-04-16 6:19:	2023-04-16 6:26:	IP Address	100	436	TRUE	2023-04-16 6:26:	R_24qGDtZH70QQHRn						anonymous	EN	
2023-04-16 6:26:	2023-04-16 6:31:	IP Address	100	296	TRUE	2023-04-16 6:31:	R_12F29j1mdBXPxwH						anonymous	EN	
2023-04-16 6:31:	2023-04-16 6:33:	IP Address	100	132	TRUE	2023-04-16 6:33:	R_2CNa1Lz3QhCt3nR						anonymous	EN	
2023-04-16 6:34:	2023-04-16 6:35:	IP Address	100	73	TRUE	2023-04-16 6:35:	R_31MbyQcrZhqxMN6						anonymous	EN	
2023-04-16 6:35:	2023-04-16 6:38:	IP Address	100	174	TRUE	2023-04-16 6:38:	R_301dzcGAlZ897vR						anonymous	EN	
2023-04-16 6:38:	2023-04-16 6:41:	IP Address	100	182	TRUE	2023-04-16 6:41:	R_3ET1dlVjReYliuX						anonymous	EN	
2023-04-16 6:41:	2023-04-16 6:42:	IP Address	100	70	TRUE	2023-04-16 6:42:	R_2fp2c8oGfRoR5GF						anonymous	EN	
2023-04-16 6:42:	2023-04-16 6:45:	IP Address	100	186	TRUE	2023-04-16 6:45:	R_beIrZJ27aKYU3Kh						anonymous	EN	
2023-04-16 6:48:	2023-04-16 6:50:	IP Address	100	87	TRUE	2023-04-16 6:50:	R_2ve2Do7iTYpO9ac						anonymous	EN	
2023-04-16 6:50:	2023-04-16 6:54:	IP Address	100	232	TRUE	2023-04-16 6:54:	R_BEXkxHqWbF4oaSB						anonymous	EN	
2023-04-16 18:0	2023-04-16 18:1	IP Address	100	558	TRUE	2023-04-16 18:1	R_3DitIuIgAk4lKGP						anonymous	EN	
2023-04-16 18:1	2023-04-16 19:2	IP Address	100	4357	TRUE	2023-04-16 19:2	R_3Ol7faVbEPO1qnm						anonymous	EN	
2023-04-17 8:29:	2023-04-17 8:46:	IP Address	100	1016	TRUE	2023-04-17 8:46:	R_Zy1yIaeRnxakZa1						anonymous	EN	
2023-04-17 8:46:	2023-04-17 9:00:	IP Address	100	804	TRUE	2023-04-17 9:00:	R_2wNw4EJauGwISGp						anonymous	EN	
2023-04-17 9:00:	2023-04-17 9:05:	IP Address	100	332	TRUE	2023-04-17 9:05:	R_2TLXwWQTfNr1r1N						anonymous	EN	
2023-04-17 9:06:	2023-04-17 9:10:	IP Address	100	261	TRUE	2023-04-17 9:10:	R_123WExIYIS0aCRI						anonymous	EN	
2023-04-17 9:10:	2023-04-17 9:11:	IP Address	100	67	TRUE	2023-04-17 9:11:	R_2s6cm7kK4atrys6						anonymous	EN	
2023-04-17 9:11:	2023-04-17 9:12:	IP Address	100	66	TRUE	2023-04-17 9:12:	R_1BRSZ5e1QqnI9XT						anonymous	EN	
2023-04-17 9:12:	2023-04-17 9:13:	IP Address	100	39	TRUE	2023-04-17 9:13:	R_s522v2Oz6sZUnn3						anonymous	EN	
2023-04-17 9:13:	2023-04-17 9:17:	IP Address	100	217	TRUE	2023-04-17 9:17:	R_cSmbWkA4Yt5UHER						anonymous	EN	
2023-04-17 9:17:	2023-04-17 9:19:	IP Address	100	136	TRUE	2023-04-17 9:19:	R_OuFVTmrTKXtYgff						anonymous	EN	
2023-04-17 9:19:	2023-04-17 9:20:	IP Address	100	54	TRUE	2023-04-17 9:20:	R_CdjgdMqGEGOuVUt						anonymous	EN	
2023-04-17 9:20:	2023-04-17 9:26:	IP Address	100	345	TRUE	2023-04-17 9:26:	R_2f6y6Npl6lNjkqm						anonymous	EN	
2023-04-17 9:26:	2023-04-17 9:28:	IP Address	100	113	TRUE	2023-04-17 9:28:	R_3hiMylFleRpGioA						anonymous	EN	
2023-04-17 9:28:	2023-04-17 9:30:	IP Address	100	164	TRUE	2023-04-17 9:30:	R_3NPPyd4NXb4uzna						anonymous	EN	
2023-04-17 9:30:	2023-04-17 9:33:	IP Address	100	178	TRUE	2023-04-17 9:33:	R_DV2vJJEcba5vgml						anonymous	EN	
2023-04-17 9:33:	2023-04-17 9:44:	IP Address	100	646	TRUE	2023-04-17 9:44:	R_TjDwvq27kZlfGcV						anonymous	EN	
2023-04-17 9:44:	2023-04-17 9:57:	IP Address	100	785	TRUE	2023-04-17 9:57:	R_2UfSb3CMJZOB7Px						anonymous	EN	
2023-04-17 9:57:	2023-04-17 10:0	IP Address	100	182	TRUE	2023-04-17 10:0	R_2hG5yoJTaZhDYm5						anonymous	EN	
2023-04-17 17:2	2023-04-17 18:5	IP Address	100	5554	TRUE	2023-04-17 18:5	R_3jcGqaHKCpvqYa9						anonymous	EN	
2023-04-18 8:02:	2023-04-18 8:10:	IP Address	100	476	TRUE	2023-04-18 8:10:	R_2V1LNDUDg51LbqT						anonymous	EN	
2023-04-18 8:10:	2023-04-18 8:11:	IP Address	100	51	TRUE	2023-04-18 8:11:	R_2saOKwyHC3Mqz10						anonymous	EN	
2023-04-18 8:11:	2023-04-18 8:19:	IP Address	100	492	TRUE	2023-04-18 8:19:	R_2UWvzMV4WYgXg4p						anonymous	EN	
2023-04-18 8:19:	2023-04-18 8:27:	IP Address	100	459	TRUE	2023-04-18 8:27:	R_31ZYbggAR7vDMcZ						anonymous	EN	
2023-04-18 8:27:	2023-04-18 8:30:	IP Address	100	169	TRUE	2023-04-18 8:30:	R_3JeHjTxuoCAvwoS						anonymous	EN	
2023-04-18 8:30:	2023-04-18 8:55:	IP Address	100	1553	TRUE	2023-04-18 8:55:	R_1LST3vsJYvq3mH3						anonymous	EN	
2023-04-18 8:56:	2023-04-18 9:15:	IP Address	100	1194	TRUE	2023-04-18 9:15:	R_10DdzGGKIGSeMZi						anonymous	EN	
2023-04-18 9:16:	2023-04-18 9:18:	IP Address	100	153	TRUE	2023-04-18 9:18:	R_3CNoUjxH954EGKh						anonymous	EN	
2023-04-18 9:18:	2023-04-18 9:25:	IP Address	100	415	TRUE	2023-04-18 9:25:	R_2Cs1ZBRWsuJZsTI						anonymous	EN	
2023-04-18 9:25:	2023-04-18 9:31:	IP Address	100	369	TRUE	2023-04-18 9:31:	R_0eziFURmzvIwvmh						anonymous	EN	
2023-04-18 16:2	2023-04-18 16:4	IP Address	100	800	TRUE	2023-04-18 16:4	R_2Eod8LdkXDHJF1J						anonymous	EN	
2023-04-18 16:4	2023-04-18 16:5	IP Address	100	527	TRUE	2023-04-18 16:5	R_eJwQYc16l7mZ1M5						anonymous	EN	
2023-04-18 16:5	2023-04-18 16:5	IP Address	100	577	TRUE	2023-04-18 16:5	R_3kEo7tzcC0JZTlD						anonymous	EN	
2023-04-19 12:0	2023-04-19 12:1	IP Address	100	629	TRUE	2023-04-19 12:1	R_3oRbCdptwqiKKXI						anonymous	EN	
2023-04-19 12:1	2023-04-19 12:3	IP Address	100	1202	TRUE	2023-04-19 12:3	R_3JxBBMCCOJXTs28						anonymous	EN	
2023-04-19 12:3	2023-04-19 12:5	IP Address	100	1151	TRUE	2023-04-19 12:5	R_2OHce1Sn34NJE0D						anonymous	EN	
2023-04-19 12:5	2023-04-19 12:5	IP Address	100	22	TRUE	2023-04-19 12:5	R_2dFSwzymCLiUBBW						anonymous	EN	
2023-04-19 12:5	2023-04-19 12:5	IP Address	100	22	TRUE	2023-04-19 12:5	R_1hRzNP12RUlJWX7						anonymous	EN	
2023-04-19 16:3	2023-04-19 16:4	IP Address	100	984	TRUE	2023-04-19 16:4	R_3qJxyxAwscd0JTj						anonymous	EN	
2023-04-19 16:5	2023-04-19 16:5	IP Address	100	296	TRUE	2023-04-19 16:5	R_2ZNWGS5vaQJmkuk						anonymous	EN	
2023-04-19 16:5	2023-04-19 17:0	IP Address	100	488	TRUE	2023-04-19 17:0	R_22VcKPJSv6E7DnR						anonymous	EN	
2023-04-19 17:0	2023-04-19 17:1	IP Address	100	651	TRUE	2023-04-19 17:1	R_1Nn1Yx7pwEUm494						anonymous	EN	
2023-04-19 17:1	2023-04-19 17:2	IP Address	100	388	TRUE	2023-04-19 17:2	R_1FvKkXQ2tLupQhr						anonymous	EN	
2023-04-19 17:2	2023-04-19 17:2	IP Address	100	415	TRUE	2023-04-19 17:2	R_25CfWrhuHbvzqVj						anonymous	EN	
2023-04-20 4:06:	2023-04-20 4:12:	IP Address	100	332	TRUE	2023-04-20 4:12:	R_3JpXfEu6JwbCQFk						anonymous	EN	
2023-04-20 4:12:	2023-04-20 4:15:	IP Address	100	201	TRUE	2023-04-20 4:15:	R_2SiWGVNBf3dQw6z						anonymous	EN	
2023-04-20 4:15:	2023-04-20 4:17:	IP Address	100	91	TRUE	2023-04-20 4:17:	R_26g2ijaiu3IumKK						anonymous	EN	
2023-04-20 4:17:	2023-04-20 4:18:	IP Address	100	86	TRUE	2023-04-20 4:18:	R_8dL6ZzdimtbuKK5						anonymous	EN	

2023-04-20 4:18	2023-04-20 4:24	IP Address	100	375	TRUE	2023-04-20 4:24	R_3dGO0Gl1ZqZvZ6J							anonymous	EN	
2023-04-20 4:24	2023-04-20 4:26	IP Address	100	83	TRUE	2023-04-20 4:26	R_4ITtsH3U1sHGo8h							anonymous	EN	
2023-04-20 4:26	2023-04-20 4:27	IP Address	100	99	TRUE	2023-04-20 4:27	R_2bJ0swkLGCY1qwU							anonymous	EN	
2023-04-20 4:27	2023-04-20 4:33	IP Address	100	336	TRUE	2023-04-20 4:33	R_3RrWYgHoZ7xTPIl							anonymous	EN	
2023-04-20 4:33	2023-04-20 4:39	IP Address	100	336	TRUE	2023-04-20 4:39	R_8A4t5vFlc73vF4d							anonymous	EN	
2023-04-20 14:31	2023-04-20 14:34	IP Address	100	137	TRUE	2023-04-20 14:34	R_2QmsFEVcZXMwOTJ							anonymous	EN	
2023-04-20 14:34	2023-04-20 14:55	IP Address	100	1282	TRUE	2023-04-20 14:55	R_3FPRCdAx18uW6tM							anonymous	EN	
2023-04-20 14:55	2023-04-20 15:15	IP Address	100	1176	TRUE	2023-04-20 15:15	R_2CV4W5K7f7pKSGe							anonymous	EN	
2023-04-20 15:15	2023-04-20 15:47	IP Address	100	1940	TRUE	2023-04-20 15:47	R_1NfYBjcLftH7HJG							anonymous	EN	
2023-04-20 15:47	2023-04-20 15:57	IP Address	100	614	TRUE	2023-04-20 15:57	R_2BaLvmseK6RM7XN							anonymous	EN	
2023-04-20 15:57	2023-04-20 16:15	IP Address	100	1063	TRUE	2023-04-20 16:15	R_1hMbBatgk4Sst6u							anonymous	EN	
2023-04-21 6:00	2023-04-21 6:36	IP Address	100	2160	TRUE	2023-04-21 6:36	R_Tua3JZHeOKM8H9D							anonymous	EN	
2023-04-21 6:36	2023-04-21 6:38	IP Address	100	133	TRUE	2023-04-21 6:38	R_1ezdhoOlE05zJDn							anonymous	EN	
2023-04-21 6:38	2023-04-21 6:41	IP Address	100	174	TRUE	2023-04-21 6:41	R_3erUpy3haklK0AM							anonymous	EN	
2023-04-21 6:41	2023-04-21 6:43	IP Address	100	131	TRUE	2023-04-21 6:43	R_R3tb4EPJ1aK574R							anonymous	EN	
2023-04-21 6:43	2023-04-21 6:48	IP Address	100	293	TRUE	2023-04-21 6:48	R_24wkAU84KtnN6Gr							anonymous	EN	
2023-04-21 6:48	2023-04-21 6:53	IP Address	100	302	TRUE	2023-04-21 6:53	R_3EbR3UBrKS3q5uC							anonymous	EN	
2023-04-21 6:53	2023-04-21 6:59	IP Address	100	362	TRUE	2023-04-21 6:59	R_3GDoMkNxPB62T8o							anonymous	EN	
2023-04-21 7:00	2023-04-21 7:06	IP Address	100	392	TRUE	2023-04-21 7:06	R_3lPkVEz0DbJEF7a							anonymous	EN	
2023-04-21 7:06	2023-04-21 7:07	IP Address	100	31	TRUE	2023-04-21 7:07	R_yxbiCc2YPhGzKvv							anonymous	EN	
2023-04-21 7:07	2023-04-21 7:17	IP Address	100	632	TRUE	2023-04-21 7:17	R_3fjD9bR9VHZSAuB							anonymous	EN	
2023-04-21 7:17	2023-04-21 7:26	IP Address	100	511	TRUE	2023-04-21 7:26	R_ukQfXO8Cki4C8Y9							anonymous	EN	
2023-04-21 7:26	2023-04-21 7:28	IP Address	100	108	TRUE	2023-04-21 7:28	R_22ApiVJQh99rnO0							anonymous	EN	
2023-04-21 7:28	2023-04-21 7:44	IP Address	100	1004	TRUE	2023-04-21 7:44	R_2ZELDAlb2fHeSJ0							anonymous	EN	
2023-04-21 7:45	2023-04-21 7:48	IP Address	100	230	TRUE	2023-04-21 7:48	R_1n2AfUFsX9LcLcF							anonymous	EN	
2023-04-21 7:48	2023-04-21 7:54	IP Address	100	366	TRUE	2023-04-21 7:54	R_1hGZZdrAkXzNSr1							anonymous	EN	
2023-04-21 7:55	2023-04-21 8:07	IP Address	100	742	TRUE	2023-04-21 8:07	R_2TKJeTnVc3zV1MZ							anonymous	EN	
2023-04-21 8:07	2023-04-21 8:12	IP Address	100	267	TRUE	2023-04-21 8:12	R_2pK6ldU6qAIJsJT							anonymous	EN	
2023-04-21 8:12	2023-04-21 8:13	IP Address	100	103	TRUE	2023-04-21 8:13	R_7X7yV8pghGa03V7							anonymous	EN	
2023-04-21 8:13	2023-04-21 8:22	IP Address	100	519	TRUE	2023-04-21 8:22	R_An8KtNsWKw4Mowx							anonymous	EN	
2023-04-21 8:22	2023-04-21 8:24	IP Address	100	113	TRUE	2023-04-21 8:24	R_1Dp8i4tgkAVjLBm							anonymous	EN	
2023-04-21 8:24	2023-04-21 8:38	IP Address	100	849	TRUE	2023-04-21 8:38	R_Ow1xkhL8NQVb6H7							anonymous	EN	
2023-04-21 10:00	2023-04-21 11:10	IP Address	100	3822	TRUE	2023-04-21 11:10	R_1OwrEk5gq6MBoQj							anonymous	EN	
2023-04-21 11:10	2023-04-21 11:26	IP Address	100	963	TRUE	2023-04-21 11:26	R_3343ccAWLPGcfYK							anonymous	EN	
2023-04-21 11:26	2023-04-21 11:52	IP Address	100	1563	TRUE	2023-04-21 11:52	R_wY0bXRH5LUzctZT							anonymous	EN	
2023-04-21 11:52	2023-04-21 11:54	IP Address	100	110	TRUE	2023-04-21 11:54	R_wSDLUm3dUmfgikN							anonymous	EN	
2023-04-22 17:24	2023-04-22 17:28	IP Address	100	262	TRUE	2023-04-22 17:28	R_3eraHDFwiSxfwE2							anonymous	EN	
2023-04-22 17:28	2023-04-22 17:32	IP Address	100	203	TRUE	2023-04-22 17:32	R_1Pepj0k1QBFloTV							anonymous	EN	
2023-04-22 17:32	2023-04-22 17:33	IP Address	100	91	TRUE	2023-04-22 17:33	R_3OlxsdpF8p2l29O							anonymous	EN	
2023-04-22 17:33	2023-04-22 17:42	IP Address	100	495	TRUE	2023-04-22 17:42	R_3MyW40YRcd8I181							anonymous	EN	
2023-04-22 17:42	2023-04-22 17:47	IP Address	100	301	TRUE	2023-04-22 17:47	R_2YfSjS3uo313CGt							anonymous	EN	
2023-04-22 17:47	2023-04-22 17:48	IP Address	100	67	TRUE	2023-04-22 17:48	R_1goNYiDke1XQqtv							anonymous	EN	
2023-04-22 17:48	2023-04-22 17:51	IP Address	100	189	TRUE	2023-04-22 17:51	R_XpkOP3OTLjf7G5b							anonymous	EN	
2023-04-22 17:51	2023-04-22 17:53	IP Address	100	134	TRUE	2023-04-22 17:53	R_7Um1ND1ve5LpR0B							anonymous	EN	
2023-04-22 17:53	2023-04-22 18:03	IP Address	100	588	TRUE	2023-04-22 18:03	R_338cTLJmhvtgg1y							anonymous	EN	
2023-04-22 18:03	2023-04-22 18:10	IP Address	100	401	TRUE	2023-04-22 18:10	R_2SvOCB5Qxu0CTGp							anonymous	EN	
2023-04-22 18:10	2023-04-22 18:15	IP Address	100	304	TRUE	2023-04-22 18:15	R_1Krom9zNwI5d8QQ							anonymous	EN	
2023-04-22 18:15	2023-04-22 18:17	IP Address	100	136	TRUE	2023-04-22 18:17	R_R38BZVmyO6KUodr							anonymous	EN	
2023-04-23 15:54	2023-04-23 18:00	IP Address	100	7571	TRUE	2023-04-23 18:00	R_4OUX5h7xaeuPNlv							anonymous	EN	
2023-04-23 18:00	2023-04-23 18:02	IP Address	100	103	TRUE	2023-04-23 18:02	R_OPV4wqJri8F3hnP							anonymous	EN	
2023-04-23 19:19	2023-04-23 19:20	IP Address	100	52	TRUE	2023-04-23 19:20	R_OlJr4UQxGtNpVgl							anonymous	EN	
2023-04-23 18:02	2023-04-23 20:23	IP Address	100	8460	TRUE	2023-04-23 20:23	R_1dpq2ZCvdXstv1s							anonymous	EN	
2023-04-23 20:25	2023-04-23 20:26	IP Address	100	57	TRUE	2023-04-23 20:26	R_YWVjNtCoAzE8KVr							anonymous	EN	
2023-04-24 11:21	2023-04-24 11:57	IP Address	100	2177	TRUE	2023-04-24 11:57	R_3RmX3ct1JzoDSDk							anonymous	EN	
2023-04-24 11:49	2023-04-24 11:58	IP Address	100	536	TRUE	2023-04-24 11:58	R_7R1RExjT8IZLQCB							anonymous	EN	
2023-04-24 11:58	2023-04-24 12:00	IP Address	100	118	TRUE	2023-04-24 12:00	R_2TsLrorgeR7tq8k							anonymous	EN	
2023-04-24 11:57	2023-04-24 12:11	IP Address	100	804	TRUE	2023-04-24 12:11	R_1oHgHKywbFPrJoD							anonymous	EN	
2023-04-24 12:00	2023-04-24 12:39	IP Address	100	2336	TRUE	2023-04-24 12:39	R_1C3YWLkcXgMmLew							anonymous	EN	
2023-04-24 12:39	2023-04-24 12:42	IP Address	100	143	TRUE	2023-04-24 12:42	R_3s6SUKlxE04fzEe							anonymous	EN	

2023-04-24 13:5	2023-04-24 14:1	IP Address	100	701	TRUE	2023-04-24 14:1	R_1jGNgtpSuEDzPNN						anonymous	EN	
2023-04-24 14:1	2023-04-24 14:1	IP Address	100	338	TRUE	2023-04-24 14:1	R_Z3RrWCUtNTWRDW1						anonymous	EN	
2023-04-24 14:1	2023-04-24 15:1	IP Address	100	3323	TRUE	2023-04-24 15:1	R_30w4FBjpxEpGXeF						anonymous	EN	
2023-04-24 15:1	2023-04-24 15:5	IP Address	100	1981	TRUE	2023-04-24 15:5	R_1eK0Ctw6JKwLtoc						anonymous	EN	
2023-04-24 22:5	2023-04-24 22:5	IP Address	100	161	TRUE	2023-04-24 22:5	R_3HZz8tILhLtzzCO						anonymous	EN	
2023-04-25 12:0	2023-04-25 12:4	IP Address	100	1846	TRUE	2023-04-25 12:4	R_3lJHa9g10hV9FZK						anonymous	EN	
2023-04-25 12:4	2023-04-25 12:4	IP Address	100	53	TRUE	2023-04-25 12:4	R_2s10kcDiKjSNTQC						anonymous	EN	
2023-04-25 12:5	2023-04-25 13:1	IP Address	100	1285	TRUE	2023-04-25 13:1	R_8wimWgbz2osDu25						anonymous	EN	
2023-04-25 14:0	2023-04-25 14:2	IP Address	100	1147	TRUE	2023-04-25 14:2	R_3qlgtyIJgULEVbm						anonymous	EN	
2023-04-25 14:2	2023-04-25 14:4	IP Address	100	1174	TRUE	2023-04-25 14:4	R_3EEbSxAskMYYWnM						anonymous	EN	
2023-04-25 14:4	2023-04-25 15:0	IP Address	100	1233	TRUE	2023-04-25 15:0	R_2Tu8lZxmLWr8CRI						anonymous	EN	
2023-04-25 15:0	2023-04-25 15:3	IP Address	100	1851	TRUE	2023-04-25 15:3	R_25YL0NYAY3qfvC1						anonymous	EN	
2023-04-25 15:3	2023-04-25 15:4	IP Address	100	780	TRUE	2023-04-25 15:4	R_3dXiSCtfoITIwc1						anonymous	EN	
2023-04-25 15:4	2023-04-25 15:5	IP Address	100	551	TRUE	2023-04-25 15:5	R_9FbyYzrbkvCn8NX						anonymous	EN	
2023-04-25 15:5	2023-04-25 15:5	IP Address	100	54	TRUE	2023-04-25 15:5	R_DMEiGQpoTrCjGb7						anonymous	EN	
2023-04-26 9:29	2023-04-26 9:45	IP Address	100	969	TRUE	2023-04-26 9:45	R_262rt2AkO7t3PCx						anonymous	EN	
2023-04-26 9:59	2023-04-26 10:0	IP Address	100	86	TRUE	2023-04-26 10:0	R_2XghsLQ2nSTZ8q1						anonymous	EN	
2023-04-26 9:45	2023-04-26 10:0	IP Address	100	1223	TRUE	2023-04-26 10:0	R_1NCGuYvT0qOux4q						anonymous	EN	
2023-04-26 10:1	2023-04-26 10:1	IP Address	100	291	TRUE	2023-04-26 10:1	R_RaCTGdGisNwkfRL						anonymous	EN	
2023-04-26 10:4	2023-04-26 10:4	IP Address	100	98	TRUE	2023-04-26 10:4	R_3MJBV4B3rRMLsdL						anonymous	EN	
2023-04-26 10:1	2023-04-26 11:2	IP Address	100	4493	TRUE	2023-04-26 11:2	R_AG4iOu3vYhbojER						anonymous	EN	
2023-04-26 11:2	2023-04-26 11:3	IP Address	100	713	TRUE	2023-04-26 11:3	R_AjHXWpZKcb7Eb6h						anonymous	EN	
2023-04-26 11:3	2023-04-26 11:4	IP Address	100	356	TRUE	2023-04-26 11:4	R_1f2wdP65fautgQl						anonymous	EN	
2023-04-26 11:4	2023-04-26 11:4	IP Address	100	94	TRUE	2023-04-26 11:4	R_1QyJh7beFD8sjfM						anonymous	EN	
2023-04-26 11:4	2023-04-26 11:5	IP Address	100	388	TRUE	2023-04-26 11:5	R_7OIocF73nWoGqAN						anonymous	EN	
2023-04-26 11:5	2023-04-26 11:5	IP Address	100	279	TRUE	2023-04-26 11:5	R_1gFJzgRIheThd0U						anonymous	EN	
2023-04-26 11:5	2023-04-26 12:5	IP Address	100	3374	TRUE	2023-04-26 12:5	R_1f2eqtoQ0jKsEHG						anonymous	EN	
2023-04-26 12:5	2023-04-26 12:5	IP Address	100	117	TRUE	2023-04-26 12:5	R_1owfhYdH7oPfjwi						anonymous	EN	
2023-04-26 16:1	2023-04-26 16:4	IP Address	100	1587	TRUE	2023-04-26 16:4	R_3DDnxvr22tj5jCp						anonymous	EN	
2023-04-26 16:4	2023-04-26 16:5	IP Address	100	775	TRUE	2023-04-26 16:5	R_3exyczeJwfyR0mT						anonymous	EN	
2023-04-26 16:5	2023-04-26 17:0	IP Address	100	257	TRUE	2023-04-26 17:0	R_3HCt9iO0o2PWLRq						anonymous	EN	
2023-04-26 17:0	2023-04-26 17:5	IP Address	100	3333	TRUE	2023-04-26 17:5	R_2uy4kfnfdzvTHlE						anonymous	EN	
2023-04-27 9:52	2023-04-27 10:0	IP Address	100	866	TRUE	2023-04-27 10:0	R_3nJysJZW02Ueu9A						anonymous	EN	
2023-04-27 10:0	2023-04-27 10:1	IP Address	100	333	TRUE	2023-04-27 10:1	R_10UeV9CS3fhuyiy						anonymous	EN	
2023-04-27 10:1	2023-04-27 10:3	IP Address	100	1490	TRUE	2023-04-27 10:3	R_31yq9ikGOSm4b63						anonymous	EN	
2023-04-27 13:0	2023-04-27 13:0	IP Address	100	142	TRUE	2023-04-27 13:0	R_rliELSGJI1v2pVf						anonymous	EN	
2023-04-27 13:0	2023-04-27 13:1	IP Address	100	36	TRUE	2023-04-27 13:1	R_50DHiD6LlSAOBWN						anonymous	EN	
2023-04-27 13:1	2023-04-27 13:1	IP Address	100	28	TRUE	2023-04-27 13:1	R_vqUOF3NbXoYIOS5						anonymous	EN	
2023-04-27 13:1	2023-04-27 13:1	IP Address	100	59	TRUE	2023-04-27 13:1	R_3O24cfB6vnxfgg2						anonymous	EN	
2023-04-27 13:1	2023-04-27 13:1	IP Address	100	103	TRUE	2023-04-27 13:1	R_1LXWA2HHQnTyW50						anonymous	EN	
2023-04-27 13:1	2023-04-27 13:1	IP Address	100	62	TRUE	2023-04-27 13:1	R_urZCOsHriBKjn69						anonymous	EN	
2023-04-27 13:1	2023-04-27 13:3	IP Address	100	972	TRUE	2023-04-27 13:3	R_2aWfF6NgPsWumlQ						anonymous	EN	
2023-04-27 13:2	2023-04-27 13:4	IP Address	100	1299	TRUE	2023-04-27 13:4	R_u8miRkjm16UWbtv						anonymous	EN	
2023-04-27 13:4	2023-04-27 13:5	IP Address	100	728	TRUE	2023-04-27 13:5	R_cNmQIndZ1NWuVhL						anonymous	EN	
2023-04-27 13:5	2023-04-27 13:5	IP Address	100	64	TRUE	2023-04-27 13:5	R_3hmrsMwWccqQAwS						anonymous	EN	
2023-04-27 13:3	2023-04-27 14:0	IP Address	100	2236	TRUE	2023-04-27 14:0	R_2dZ6gwDbL6s0C9N						anonymous	EN	
2023-04-27 13:5	2023-04-27 14:3	IP Address	100	1983	TRUE	2023-04-27 14:3	R_1gNrzjgV1BOS6MF						anonymous	EN	
2023-04-28 10:5	2023-04-28 11:0	IP Address	100	631	TRUE	2023-04-28 11:0	R_28J6YS0EDWMwIP0						anonymous	EN	
2023-04-28 11:0	2023-04-28 11:0	IP Address	100	32	TRUE	2023-04-28 11:0	R_31WWWzfNwpd832y						anonymous	EN	
2023-04-28 11:0	2023-04-28 11:5	IP Address	100	3192	TRUE	2023-04-28 11:5	R_2VD6oQnVaNe2mfO						anonymous	EN	
2023-04-28 11:5	2023-04-28 12:4	IP Address	100	2899	TRUE	2023-04-28 12:4	R_9YK9NhaZSmqQhgt						anonymous	EN	
2023-04-28 15:5	2023-04-28 16:1	IP Address	100	1083	TRUE	2023-04-28 16:1	R_2eRWAVIjANFK8VR						anonymous	EN	
2023-04-28 16:2	2023-04-28 16:2	IP Address	100	54	TRUE	2023-04-28 16:2	R_3JFbN3R4zbbLKco						anonymous	EN	
2023-04-28 16:5	2023-04-28 16:5	IP Address	100	48	TRUE	2023-04-28 16:5	R_25WNvPhdftvtM0y						anonymous	EN	
2023-04-28 17:1	2023-04-28 17:2	IP Address	100	78	TRUE	2023-04-28 17:2	R_2mFvpaPPhXJKWbL						anonymous	EN	
2023-04-28 17:2	2023-04-28 17:2	IP Address	100	70	TRUE	2023-04-28 17:2	R_12QDQ0f3q4rXfwS						anonymous	EN	
2023-04-28 22:4	2023-04-28 22:4	IP Address	100	66	TRUE	2023-04-28 22:4	R_31Wprx68JzlXzww						anonymous	EN	
2023-04-28 22:5	2023-04-28 22:5	IP Address	100	55	TRUE	2023-04-28 22:5	R_23TXRa0HxoLOwSg						anonymous	EN	
2023-04-29 11:0	2023-04-29 11:0	IP Address	100	123	TRUE	2023-04-29 11:0	R_28SvyQnR2fUdwB5						anonymous	EN	

2023-04-29 11:26	2023-04-29 11:36	IP Address	100	635	TRUE	2023-04-29 11:36	R_1HiYhvVKblhGdfL							anonymous	EN	
2023-04-29 11:42	2023-04-29 11:44	IP Address	100	78	TRUE	2023-04-29 11:44	R_3iIOTYyACgzHMqu							anonymous	EN	
2023-04-29 11:36	2023-04-29 12:02	IP Address	100	1574	TRUE	2023-04-29 12:02	R_bDx1ypwQeB5ZQqt							anonymous	EN	
2023-04-29 12:04	2023-04-29 12:05	IP Address	100	65	TRUE	2023-04-29 12:05	R_Bu2PY98LEa6fFMl							anonymous	EN	
2023-04-29 12:02	2023-04-29 12:11	IP Address	100	506	TRUE	2023-04-29 12:11	R_21bLyaVClamM1Lr							anonymous	EN	
2023-04-29 12:11	2023-04-29 12:18	IP Address	100	428	TRUE	2023-04-29 12:18	R_1GK29odAJ4UXYOm							anonymous	EN	
2023-04-29 12:18	2023-04-29 12:21	IP Address	100	144	TRUE	2023-04-29 12:21	R_3oFQiOOKthJGvlu							anonymous	EN	
2023-04-29 12:21	2023-04-29 12:25	IP Address	100	258	TRUE	2023-04-29 12:25	R_3Jla2mvvn52zty9							anonymous	EN	
2023-04-29 12:25	2023-04-29 12:32	IP Address	100	433	TRUE	2023-04-29 12:32	R_3PX3yFcCGQp2huv							anonymous	EN	
2023-04-29 12:32	2023-04-29 12:42	IP Address	100	610	TRUE	2023-04-29 12:42	R_YbKpK3YZfbVNMfT							anonymous	EN	
2023-04-29 12:42	2023-04-29 12:50	IP Address	100	477	TRUE	2023-04-29 12:50	R_3m90H5p6QgVIift							anonymous	EN	
2023-04-29 12:50	2023-04-29 12:53	IP Address	100	163	TRUE	2023-04-29 12:53	R_1M5QwOoG3i5AV6D							anonymous	EN	
2023-04-29 12:53	2023-04-29 12:56	IP Address	100	191	TRUE	2023-04-29 12:56	R_3MhQqWhH771BhLo							anonymous	EN	
2023-04-29 13:52	2023-04-29 13:53	IP Address	100	55	TRUE	2023-04-29 13:53	R_28MXQY46fr6Xc8L							anonymous	EN	
2023-04-29 14:05	2023-04-29 14:06	IP Address	100	40	TRUE	2023-04-29 14:06	R_33sBMNXmIHkyBw3							anonymous	EN	
2023-04-29 14:15	2023-04-29 14:15	IP Address	100	30	TRUE	2023-04-29 14:15	R_1AYCZcHszCULrW1							anonymous	EN	
2023-04-29 14:22	2023-04-29 14:24	IP Address	100	105	TRUE	2023-04-29 14:24	R_OcYjBOrol9fstAB							anonymous	EN	
2023-04-29 20:50	2023-04-29 20:51	IP Address	100	49	TRUE	2023-04-29 20:51	R_2B9AmZaUfJdzNtG							anonymous	EN	
2023-04-30 10:59	2023-04-30 11:08	IP Address	100	541	TRUE	2023-04-30 11:08	R_0lHZjTQEQltKINP							anonymous	EN	
2023-04-30 11:08	2023-04-30 11:14	IP Address	100	351	TRUE	2023-04-30 11:14	R_31LsQQ1OZxfC5Pj							anonymous	EN	
2023-04-30 11:14	2023-04-30 11:21	IP Address	100	420	TRUE	2023-04-30 11:21	R_sBeGCzGQQ8CAVi1							anonymous	EN	
2023-04-30 11:21	2023-04-30 11:25	IP Address	100	226	TRUE	2023-04-30 11:25	R_3lPopyNMgrBfKPN							anonymous	EN	
2023-04-30 11:25	2023-04-30 11:28	IP Address	100	166	TRUE	2023-04-30 11:28	R_3PsEpAyjLzEFRXq							anonymous	EN	
2023-04-30 11:28	2023-04-30 11:38	IP Address	100	586	TRUE	2023-04-30 11:38	R_56ca8ZmQkprQ521							anonymous	EN	
2023-04-30 11:38	2023-04-30 11:40	IP Address	100	104	TRUE	2023-04-30 11:40	R_1fZ1qzzcOG1LGiG							anonymous	EN	
2023-04-30 11:40	2023-04-30 11:54	IP Address	100	879	TRUE	2023-04-30 11:54	R_2cwjb9cHp9osX0k							anonymous	EN	
2023-04-30 11:54	2023-04-30 11:55	IP Address	100	25	TRUE	2023-04-30 11:55	R_Rh6QP25yVigdDq1							anonymous	EN	
2023-04-30 19:38	2023-04-30 19:39	IP Address	100	36	TRUE	2023-04-30 19:39	R_BrhX4pMoft4Qqyt							anonymous	EN	
2023-04-30 20:02	2023-04-30 20:04	IP Address	100	70	TRUE	2023-04-30 20:04	R_1jWiWTVSvHSyZ3r							anonymous	EN	
2023-04-30 23:54	2023-04-30 23:55	IP Address	100	54	TRUE	2023-04-30 23:55	R_3fCQZOLMuocDykh							anonymous	EN	
2023-05-01 0:10:	2023-05-01 0:10:	IP Address	100	47	TRUE	2023-05-01 0:10:	R_1C8ujLLFxBf8tor							anonymous	EN	
2023-05-01 0:34:	2023-05-01 0:37:	IP Address	100	129	TRUE	2023-05-01 0:37:	R_3kEXe27JadfT99l							anonymous	EN	
2023-05-05 6:42:	2023-05-05 7:07:	IP Address	100	1475	TRUE	2023-05-05 7:07:	R_31nsYXj1Twaj62h							anonymous	EN	
2023-05-05 7:07:	2023-05-05 7:17:	IP Address	100	634	TRUE	2023-05-05 7:17:	R_tPZ9zIaSwQNsMNP							anonymous	EN	
2023-05-05 7:42:	2023-05-05 7:47:	IP Address	100	310	TRUE	2023-05-05 7:47:	R_2zkiV8P2bP6Wkjz							anonymous	EN	
2023-05-05 7:47:	2023-05-05 10:20	IP Address	100	9533	TRUE	2023-05-05 10:20	R_26bYx1V9m3wFb7X							anonymous	EN	
2023-05-05 10:30	2023-05-05 10:31	IP Address	100	32	TRUE	2023-05-05 10:31	R_25u3SQGXSgpouvH							anonymous	EN	
2023-05-10 5:00:	2023-05-10 5:13:	IP Address	100	792	TRUE	2023-05-10 5:13:	R_1jBBN2RhMTxGVGC							anonymous	EN	
2023-05-10 5:13:	2023-05-10 5:15:	IP Address	100	140	TRUE	2023-05-10 5:15:	R_1FwB8BDxs9higZs							anonymous	EN	
2023-05-10 5:15:	2023-05-10 5:28:	IP Address	100	760	TRUE	2023-05-10 5:28:	R_3OqV8TMFTlj6CuP							anonymous	EN	
2023-05-10 5:28:	2023-05-10 5:37:	IP Address	100	553	TRUE	2023-05-10 5:37:	R_3iz3W6jfeiBTSXA							anonymous	EN	
2023-05-11 6:17:	2023-05-11 6:25:	IP Address	100	430	TRUE	2023-05-11 6:25:	R_1OWdWEDhJctlNXw							anonymous	EN	
2023-05-11 6:25:	2023-05-11 6:28:	IP Address	100	201	TRUE	2023-05-11 6:28:	R_3Mhz0N3DR72qheW							anonymous	EN	
2023-05-11 6:28:	2023-05-11 6:33:	IP Address	100	275	TRUE	2023-05-11 6:33:	R_2VEIhG2aAZywa1b							anonymous	EN	
2023-05-11 6:33:	2023-05-11 6:40:	IP Address	100	414	TRUE	2023-05-11 6:40:	R_2qxeOyc3wn9ybag							anonymous	EN	
2023-05-11 6:40:	2023-05-11 7:08:	IP Address	100	1684	TRUE	2023-05-11 7:08:	R_Qlxjt5l83Hxhf1v							anonymous	EN	
2023-05-11 7:08:	2023-05-11 7:23:	IP Address	100	891	TRUE	2023-05-11 7:23:	R_1dNvvINTmmNT7Rt							anonymous	EN	
2023-05-12 11:39	2023-05-12 12:25	IP Address	100	2736	TRUE	2023-05-12 12:25	R_1HiX19ByOLSTiKR							anonymous	EN	
2023-05-12 12:25	2023-05-12 12:28	IP Address	100	174	TRUE	2023-05-12 12:28	R_1pF4TgsBcrl4TTl							anonymous	EN	
2023-05-12 12:28	2023-05-12 12:30	IP Address	100	148	TRUE	2023-05-12 12:30	R_UmU09AR9JvxUBQB							anonymous	EN	
2023-05-12 12:30	2023-05-12 12:34	IP Address	100	238	TRUE	2023-05-12 12:34	R_2D66otQsWYjwZNV							anonymous	EN	
2023-05-13 12:31	2023-05-13 12:33	IP Address	100	116	TRUE	2023-05-13 12:33	R_1obi1GRcprKLtF5							anonymous	EN	
2023-05-13 12:33	2023-05-13 12:35	IP Address	100	144	TRUE	2023-05-13 12:35	R_T7PJLjyy6mdoSwp							anonymous	EN	
2023-05-13 12:35	2023-05-13 12:36	IP Address	100	82	TRUE	2023-05-13 12:36	R_2ykL7FsYNLyA0aB							anonymous	EN	
2023-05-13 12:37	2023-05-13 12:39	IP Address	100	141	TRUE	2023-05-13 12:39	R_2uzSG72cifltRzH							anonymous	EN	
2023-05-13 12:39	2023-05-13 13:20	IP Address	100	2491	TRUE	2023-05-13 13:20	R_1cRMPrmIhJgmoPT							anonymous	EN	
2023-05-13 13:20	2023-05-13 13:22	IP Address	100	120	TRUE	2023-05-13 13:22	R_2aWDn9sbwWl3005							anonymous	EN	
2023-05-13 13:23	2023-05-13 13:27	IP Address	100	261	TRUE	2023-05-13 13:27	R_1jy4qMYrJCjOJ3c							anonymous	EN	
2023-05-13 13:27	2023-05-13 13:30	IP Address	100	183	TRUE	2023-05-13 13:30	R_3244SXWPD5SQnI9							anonymous	EN	

2023-05-13 20:1	2023-05-13 20:1	IP Address	100	348	TRUE	2023-05-13 20:1	R_6fI1Ymakx9O7lWp							anonymous	EN	
2023-05-14 16:4	2023-05-14 17:0	IP Address	100	923	TRUE	2023-05-14 17:0	R_DTfQt6NsSsDKO77							anonymous	EN	
2023-05-14 17:0	2023-05-14 17:0	IP Address	100	86	TRUE	2023-05-14 17:0	R_3PZf4wlgQxKKfe1							anonymous	EN	
2023-05-14 17:0	2023-05-14 17:0	IP Address	100	121	TRUE	2023-05-14 17:0	R_z7oaQ6CpMo1lmRz							anonymous	EN	
2023-05-14 17:0	2023-05-14 17:1	IP Address	100	235	TRUE	2023-05-14 17:1	R_1pWi0YMjlKiTugF							anonymous	EN	
2023-05-14 12:2	2023-05-14 17:2	IP Address	100	18260	TRUE	2023-05-14 17:2	R_1TvkbsNjumopc1r							anonymous	EN	
2023-05-14 17:2	2023-05-14 17:2	IP Address	100	144	TRUE	2023-05-14 17:2	R_2CvRR04t5QhexVi							anonymous	EN	
2023-05-16 8:00	2023-05-16 8:10	IP Address	100	621	TRUE	2023-05-16 8:10	R_1Cv7LIAry1unJjU							anonymous	EN	
2023-05-16 8:10	2023-05-16 8:12	IP Address	100	110	TRUE	2023-05-16 8:12	R_1I5kmsZ8UPPp2s0							anonymous	EN	
2023-05-16 8:12	2023-05-16 8:14	IP Address	100	114	TRUE	2023-05-16 8:14	R_2eVHfqLWM0GCP1A							anonymous	EN	
2023-05-16 8:15	2023-05-16 8:16	IP Address	100	91	TRUE	2023-05-16 8:16	R_24tLztivf488d9l							anonymous	EN	
2023-05-16 8:16	2023-05-16 8:20	IP Address	100	248	TRUE	2023-05-16 8:20	R_XYtQyD3OkXQP4hb							anonymous	EN	
2023-05-16 8:20	2023-05-16 9:21	IP Address	100	3645	TRUE	2023-05-16 9:21	R_1eXzV1QRLg37McO							anonymous	EN	
2023-05-16 9:21	2023-05-16 9:23	IP Address	100	87	TRUE	2023-05-16 9:23	R_2ah6WgqHGLiARgl							anonymous	EN	
2023-05-16 9:23	2023-05-16 9:43	IP Address	100	1197	TRUE	2023-05-16 9:43	R_2BwK9MgBtnWCDQ2							anonymous	EN	
2023-05-16 9:43	2023-05-16 9:45	IP Address	100	106	TRUE	2023-05-16 9:45	R_41IGFYIDHpoDXSp							anonymous	EN	
2023-05-17 4:22	2023-05-17 4:34	IP Address	100	713	TRUE	2023-05-17 4:34	R_10CABQhDm6fljbg							anonymous	EN	
2023-05-17 4:34	2023-05-17 4:51	IP Address	100	995	TRUE	2023-05-17 4:51	R_qwO1yKMWUEF8FY5							anonymous	EN	
2023-05-17 4:51	2023-05-17 5:05	IP Address	100	876	TRUE	2023-05-17 5:05	R_3J4aoLKvuTXojUd							anonymous	EN	
2023-05-17 5:05	2023-05-17 5:29	IP Address	100	1396	TRUE	2023-05-17 5:29	R_2fkA59LeHG4PnG7							anonymous	EN	
2023-05-18 17:5	2023-05-18 18:0	IP Address	100	285	TRUE	2023-05-18 18:0	R_DOUrrH4nwPjiMkF							anonymous	EN	
2023-05-18 18:0	2023-05-18 18:0	IP Address	100	393	TRUE	2023-05-18 18:0	R_2QDtpb5hWTPt7a9							anonymous	EN	
2023-05-18 18:0	2023-05-18 18:0	IP Address	100	114	TRUE	2023-05-18 18:0	R_XZoHjrD7v63Sotr							anonymous	EN	
2023-05-18 18:0	2023-05-18 18:1	IP Address	100	91	TRUE	2023-05-18 18:1	R_9mZp9Fhl1Gryywp							anonymous	EN	
2023-05-18 18:1	2023-05-18 18:1	IP Address	100	116	TRUE	2023-05-18 18:1	R_3n9pnGLZWgw6ksq							anonymous	EN	
2023-05-18 18:1	2023-05-18 18:1	IP Address	100	135	TRUE	2023-05-18 18:1	R_3kAkPnxDnjjOX1K							anonymous	EN	
2023-05-18 18:1	2023-05-18 18:2	IP Address	100	336	TRUE	2023-05-18 18:2	R_qPoraIA6sNe1iz7							anonymous	EN	
2023-05-19 8:03	2023-05-19 8:15	IP Address	100	731	TRUE	2023-05-19 8:15	R_3Phs2VOwjASZgcy							anonymous	EN	
2023-05-19 8:16	2023-05-19 8:18	IP Address	100	91	TRUE	2023-05-19 8:18	R_3kuCFHPgpAd34AJ							anonymous	EN	
2023-05-19 8:18	2023-05-19 8:20	IP Address	100	151	TRUE	2023-05-19 8:20	R_3O1QWom70XTuPns							anonymous	EN	
2023-05-19 8:20	2023-05-19 8:23	IP Address	100	131	TRUE	2023-05-19 8:23	R_2BmULZrQTQo6cWl							anonymous	EN	
2023-05-19 8:23	2023-05-19 8:24	IP Address	100	82	TRUE	2023-05-19 8:24	R_0D07vZZB585ame5							anonymous	EN	
2023-05-19 8:24	2023-05-19 9:24	IP Address	100	3605	TRUE	2023-05-19 9:24	R_1nPmiY8jLqyXcPn							anonymous	EN	
2023-05-19 9:24	2023-05-19 9:26	IP Address	100	85	TRUE	2023-05-19 9:26	R_1hKDc4Q9CJZq8CZ							anonymous	EN	
2023-05-19 9:26	2023-05-19 9:27	IP Address	100	72	TRUE	2023-05-19 9:27	R_1Q0nE3J5ShHGnqh							anonymous	EN	
2023-05-19 9:27	2023-05-19 9:28	IP Address	100	83	TRUE	2023-05-19 9:28	R_29v2phqymsr0YRN							anonymous	EN	
2023-05-19 9:28	2023-05-19 9:30	IP Address	100	101	TRUE	2023-05-19 9:30	R_1Iv1IJt89KV86uV							anonymous	EN	
2023-05-19 9:30	2023-05-19 9:33	IP Address	100	163	TRUE	2023-05-19 9:33	R_RkVa84tl9Q5ODN7							anonymous	EN	
2023-05-20 18:4	2023-05-20 18:4	IP Address	100	94	TRUE	2023-05-20 18:4	R_2dRttwiVCuz6eSH							anonymous	EN	
2023-05-20 18:4	2023-05-20 18:4	IP Address	100	28	TRUE	2023-05-20 18:4	R_2fg0iowlmpxJY0L							anonymous	EN	
2023-05-20 18:4	2023-05-20 18:5	IP Address	100	287	TRUE	2023-05-20 18:5	R_RCB2x8aJqAigdK9							anonymous	EN	
2023-05-20 18:5	2023-05-20 18:5	IP Address	100	89	TRUE	2023-05-20 18:5	R_2ve34A4SyAEwm3t							anonymous	EN	
2023-05-20 18:5	2023-05-20 18:5	IP Address	100	109	TRUE	2023-05-20 18:5	R_29jqIqky5GEy20L							anonymous	EN	
2023-05-20 18:5	2023-05-20 18:5	IP Address	100	68	TRUE	2023-05-20 18:5	R_1igStCBadrfPo2U							anonymous	EN	
2023-05-20 18:5	2023-05-20 18:5	IP Address	100	65	TRUE	2023-05-20 18:5	R_2aRDa09c3wyLZRy							anonymous	EN	
2023-05-21 15:4	2023-05-21 16:0	IP Address	100	1078	TRUE	2023-05-21 16:0	R_3JyOkZouFLbmduG							anonymous	EN	
2023-05-21 16:0	2023-05-21 16:0	IP Address	100	170	TRUE	2023-05-21 16:0	R_RKVoo0SBKQHmZ0d							anonymous	EN	
2023-05-22 15:2	2023-05-22 15:2	IP Address	100	215	TRUE	2023-05-22 15:2	R_1rv5tslYFNMTWI1							anonymous	EN	
2023-05-22 15:2	2023-05-22 15:3	IP Address	100	335	TRUE	2023-05-22 15:3	R_1mK6TsuacwsXMgD							anonymous	EN	
2023-05-22 15:3	2023-05-22 15:3	IP Address	100	219	TRUE	2023-05-22 15:3	R_21dJ3yqw8QqyZ6T							anonymous	EN	
2023-05-22 15:3	2023-05-22 16:0	IP Address	100	1750	TRUE	2023-05-22 16:0	R_wTSh258OofGJT5n							anonymous	EN	
2023-05-22 16:0	2023-05-22 16:0	IP Address	100	78	TRUE	2023-05-22 16:0	R_cSbOLRFrtCpxRBL							anonymous	EN	
2023-05-22 16:0	2023-05-22 16:0	IP Address	100	73	TRUE	2023-05-22 16:0	R_3hsk0CSaTDdccA3							anonymous	EN	
2023-05-22 16:0	2023-05-22 16:0	IP Address	100	91	TRUE	2023-05-22 16:0	R_2CBuXPvJsOMhvZg							anonymous	EN	
2023-05-22 16:0	2023-05-22 16:2	IP Address	100	1165	TRUE	2023-05-22 16:2	R_D0FMinGq9bEqJ57							anonymous	EN	
2023-05-22 16:2	2023-05-22 16:3	IP Address	100	125	TRUE	2023-05-22 16:3	R_237Q8svE4PqdPXA							anonymous	EN	
2023-05-22 16:3	2023-05-22 16:3	IP Address	100	365	TRUE	2023-05-22 16:3	R_238KAWVjhsBiPPK							anonymous	EN	
2023-05-23 18:0	2023-05-23 18:0	IP Address	100	186	TRUE	2023-05-23 18:0	R_1rAG7Y5NXQz25Oj							anonymous	EN	
2023-05-23 18:0	2023-05-23 18:0	IP Address	100	90	TRUE	2023-05-23 18:0	R_3hxsi2gII5OT0Gm							anonymous	EN	

2023-05-23 18:0	2023-05-23 18:0	IP Address	100	203	TRUE	2023-05-23 18:0	R_33fdRl9XUcGwBhH							anonymous	EN	
2023-05-23 18:0	2023-05-23 18:1	IP Address	100	123	TRUE	2023-05-23 18:1	R_OILXO9y7rig3lGp							anonymous	EN	
2023-05-23 18:1	2023-05-23 18:1	IP Address	100	157	TRUE	2023-05-23 18:1	R_1DIu3I7vQpWDIoR							anonymous	EN	
2023-05-23 18:1	2023-05-23 18:2	IP Address	100	308	TRUE	2023-05-23 18:2	R_33sagccf8oknKaE							anonymous	EN	
2023-05-23 18:2	2023-05-23 18:2	IP Address	100	48	TRUE	2023-05-23 18:2	R_2BhS6K11OcdAaBD							anonymous	EN	
2023-05-24 13:2	2023-05-24 13:3	IP Address	100	240	TRUE	2023-05-24 13:3	R_3QVFYI4h0CCSWEH							anonymous	EN	
2023-05-24 13:3	2023-05-24 13:3	IP Address	100	59	TRUE	2023-05-24 13:3	R_2wNtLIFwCPQVZsF							anonymous	EN	
2023-05-24 13:3	2023-05-24 13:3	IP Address	100	166	TRUE	2023-05-24 13:3	R_1XGUjizA4xwbC5H							anonymous	EN	
2023-05-24 13:3	2023-05-24 13:3	IP Address	100	89	TRUE	2023-05-24 13:3	R_3lGDnCMG9MyLcEN							anonymous	EN	
2023-05-24 13:3	2023-05-24 13:4	IP Address	100	374	TRUE	2023-05-24 13:4	R_30iQHRarycz1P9l							anonymous	EN	
2023-05-24 13:4	2023-05-24 13:4	IP Address	100	145	TRUE	2023-05-24 13:4	R_2Y3dVFJ5oPr2GEb							anonymous	EN	
2023-05-24 13:4	2023-05-24 13:4	IP Address	100	108	TRUE	2023-05-24 13:4	R_2WYl2g4iOkLYsit							anonymous	EN	
2023-05-24 13:4	2023-05-24 13:4	IP Address	100	73	TRUE	2023-05-24 13:4	R_3mf6ofk2Pb3uqop							anonymous	EN	
2023-05-24 13:4	2023-05-24 13:5	IP Address	100	168	TRUE	2023-05-24 13:5	R_3g7k92yvULyjkJz							anonymous	EN	
2023-05-24 13:5	2023-05-24 14:0	IP Address	100	913	TRUE	2023-05-24 14:0	R_0udQYrw3VWsbwWd							anonymous	EN	
2023-05-24 14:0	2023-05-24 14:1	IP Address	100	511	TRUE	2023-05-24 14:1	R_3iC3yeJ9BZXnZv0							anonymous	EN	
2023-05-24 14:1	2023-05-24 14:2	IP Address	100	702	TRUE	2023-05-24 14:2	R_R36QK7uAtQEHsK5							anonymous	EN	
2023-05-25 22:3	2023-05-25 22:4	IP Address	100	570	TRUE	2023-05-25 22:4	R_8dbQQbMpi5dEd1v							anonymous	EN	
2023-05-26 16:3	2023-05-26 16:3	IP Address	100	142	TRUE	2023-05-26 16:3	R_Dc1F3KqiRlz6BCp							anonymous	EN	
2023-05-26 16:3	2023-05-26 16:3	IP Address	100	81	TRUE	2023-05-26 16:3	R_1ItnOe5espuryOa							anonymous	EN	
2023-05-26 16:3	2023-05-26 16:4	IP Address	100	168	TRUE	2023-05-26 16:4	R_1NsnX7gtYzuZpOf							anonymous	EN	
2023-05-26 16:4	2023-05-26 16:4	IP Address	100	58	TRUE	2023-05-26 16:4	R_1Kmap3YWAcpdf78							anonymous	EN	
2023-05-26 16:4	2023-05-26 16:4	IP Address	100	140	TRUE	2023-05-26 16:4	R_VOVvsYLNbUmmIZr							anonymous	EN	
2023-05-26 16:4	2023-05-26 16:5	IP Address	100	614	TRUE	2023-05-26 16:5	R_1CgxaY39uk3QDR4							anonymous	EN	
2023-05-26 16:5	2023-05-26 17:0	IP Address	100	375	TRUE	2023-05-26 17:0	R_6FEigEPudnNRcLD							anonymous	EN	
2023-05-26 17:0	2023-05-26 17:0	IP Address	100	58	TRUE	2023-05-26 17:0	R_9TXTXNZ9dW9jbfX							anonymous	EN	
2023-05-26 17:0	2023-05-26 17:0	IP Address	100	71	TRUE	2023-05-26 17:0	R_3oLXjn1BRksn9vk							anonymous	EN	
2023-05-26 17:0	2023-05-26 17:0	IP Address	100	76	TRUE	2023-05-26 17:0	R_3OqQSrOgob2Vj0V							anonymous	EN	
2023-05-26 17:0	2023-05-26 17:0	IP Address	100	108	TRUE	2023-05-26 17:0	R_stAANDvyLyzdAvD							anonymous	EN	
2023-05-26 17:0	2023-05-26 17:2	IP Address	100	970	TRUE	2023-05-26 17:2	R_2z93dMm1teBSmZX							anonymous	EN	
2023-05-26 23:4	2023-05-26 23:5	IP Address	100	481	TRUE	2023-05-26 23:5	R_2diFTzKexwaSeUX							anonymous	EN	
2023-05-27 17:0	2023-05-27 17:0	IP Address	100	27	TRUE	2023-05-27 17:0	R_R53z29b0C38Os49							anonymous	EN	
2023-05-27 20:1	2023-05-27 20:1	IP Address	100	16	TRUE	2023-05-27 20:1	R_2S8O0JS2oTQcq8S							anonymous	EN	
2023-05-27 20:3	2023-05-27 20:4	IP Address	100	174	TRUE	2023-05-27 20:4	R_29hRz0zbjYJ8pl6							anonymous	EN	
2023-05-29 12:1	2023-05-29 12:2	IP Address	100	172	TRUE	2023-05-29 12:2	R_6FQmTU4OazxMVsB							anonymous	EN	
2023-05-29 12:2	2023-05-29 12:2	IP Address	100	239	TRUE	2023-05-29 12:2	R_1eLasIc0gVAlgdb							anonymous	EN	
2023-05-29 12:2	2023-05-29 12:4	IP Address	100	1107	TRUE	2023-05-29 12:4	R_3G0p3UHEpZPbapy							anonymous	EN	
2023-05-29 12:4	2023-05-29 12:4	IP Address	100	245	TRUE	2023-05-29 12:4	R_25RaC55Ny4Nzd5f							anonymous	EN	
2023-05-29 12:4	2023-05-29 12:5	IP Address	100	371	TRUE	2023-05-29 12:5	R_1IvmjqqOrOPKVMB							anonymous	EN	
2023-05-29 12:5	2023-05-29 13:0	IP Address	100	642	TRUE	2023-05-29 13:0	R_3HGHwp4LaW3Plj6							anonymous	EN	
2023-05-30 16:2	2023-05-30 16:3	IP Address	100	581	TRUE	2023-05-30 16:3	R_1EbqH2lpKY8t0ci							anonymous	EN	
2023-05-30 16:3	2023-05-30 16:3	IP Address	100	291	TRUE	2023-05-30 16:3	R_1H8A4OZDD35x8x6							anonymous	EN	
2023-05-30 16:3	2023-05-30 16:3	IP Address	100	73	TRUE	2023-05-30 16:3	R_1NaCjScU1rUi62y							anonymous	EN	
2023-05-30 16:3	2023-05-30 16:4	IP Address	100	447	TRUE	2023-05-30 16:4	R_12tBRXmvOSnkWkF							anonymous	EN	
2023-05-30 16:4	2023-05-30 16:4	IP Address	100	231	TRUE	2023-05-30 16:4	R_2aglwMsWYjsZj6k							anonymous	EN	
2023-05-30 16:4	2023-05-30 16:5	IP Address	100	436	TRUE	2023-05-30 16:5	R_XsHgprOQg4mN68x							anonymous	EN	
2023-05-30 16:5	2023-05-30 16:5	IP Address	100	79	TRUE	2023-05-30 16:5	R_1nUtj61gwGBmL5z							anonymous	EN	
2023-05-30 16:5	2023-05-30 17:0	IP Address	100	167	TRUE	2023-05-30 17:0	R_XnXf6FIyoglegZH							anonymous	EN	
2023-05-30 17:0	2023-05-30 17:0	IP Address	100	361	TRUE	2023-05-30 17:0	R_1Lu0QJ0z4AZSwl1							anonymous	EN	
2023-05-30 17:0	2023-05-30 17:0	IP Address	100	120	TRUE	2023-05-30 17:0	R_3FeoJElsS1rL6QV							anonymous	EN	
2023-05-30 17:0	2023-05-30 17:1	IP Address	100	559	TRUE	2023-05-30 17:1	R_27QzYJBeX8rNQcE							anonymous	EN	
2023-05-30 17:1	2023-05-30 17:2	IP Address	100	211	TRUE	2023-05-30 17:2	R_1T7DTUWu98nvrTb							anonymous	EN	
2023-05-31 4:45:	2023-05-31 5:03:	IP Address	100	1101	TRUE	2023-05-31 5:03:	R_xfuQT5Yg6EzATtf							anonymous	EN	
2023-05-31 5:03:	2023-05-31 5:07:	IP Address	100	215	TRUE	2023-05-31 5:07:	R_3feKGx2aFziJPzj							anonymous	EN	
2023-05-31 5:07:	2023-05-31 5:08:	IP Address	100	26	TRUE	2023-05-31 5:08:	R_3Ea2vapiIryIF4L							anonymous	EN	
2023-05-31 5:08:	2023-05-31 5:41:	IP Address	100	1976	TRUE	2023-05-31 5:41:	R_1eqQrvLxLEqzpOt							anonymous	EN	
2023-05-31 5:41:	2023-05-31 6:13:	IP Address	100	1940	TRUE	2023-05-31 6:13:	R_2EdaZohkjugMd1E							anonymous	EN	
2023-05-31 6:13:	2023-05-31 6:24:	IP Address	100	653	TRUE	2023-05-31 6:24:	R_1kFc1NVavLwQzN5							anonymous	EN	
2023-05-31 16:0	2023-05-31 16:1	IP Address	100	204	TRUE	2023-05-31 16:1	R_2Ym6X7qZKIM77KU							anonymous	EN	

2023-05-31 16:1	2023-05-31 16:5	IP Address	100	2655	TRUE	2023-05-31 16:5	R_C9RhVFrTiPwgrVD							anonymous	EN	
2023-06-04 10:5	2023-06-04 10:5	IP Address	100	144	TRUE	2023-06-04 10:5	R_1DYlBfKSDdtFjfp							anonymous	EN	
2023-06-04 17:5	2023-06-04 17:5	IP Address	100	108	TRUE	2023-06-04 17:5	R_2EAi9Lekd2dUGJO							anonymous	EN	
2023-06-04 19:5	2023-06-04 20:0	IP Address	100	155	TRUE	2023-06-04 20:0	R_2bOGagwBDFWdzEa							anonymous	EN	
2023-06-07 5:04	2023-06-07 5:19	IP Address	100	888	TRUE	2023-06-07 5:19	R_1r8MhYaHwtUVSEj							anonymous	EN	
2023-06-07 5:19	2023-06-07 5:22	IP Address	100	213	TRUE	2023-06-07 5:22	R_pDBO6t98gltH7ih							anonymous	EN	
2023-06-07 5:53	2023-06-07 5:58	IP Address	100	282	TRUE	2023-06-07 5:58	R_2X5MWHvx1D037gc							anonymous	EN	
2023-06-07 22:5	2023-06-07 23:0	IP Address	100	975	TRUE	2023-06-07 23:0	R_ag6SWnRlt6O2uFb							anonymous	EN	
2023-06-07 23:0	2023-06-07 23:1	IP Address	100	215	TRUE	2023-06-07 23:1	R_3HHxWNDpFjz5GPZ							anonymous	EN	
2023-06-08 15:3	2023-06-08 16:1	IP Address	100	2744	TRUE	2023-06-08 16:1	R_3CJVygbOh3uXqSO							anonymous	EN	
2023-06-08 16:1	2023-06-08 16:1	IP Address	100	78	TRUE	2023-06-08 16:1	R_3JgBSljHa8Lgc7Y							anonymous	EN	
2023-06-09 14:1	2023-06-09 14:1	IP Address	100	27	TRUE	2023-06-09 14:1	R_2aOZgIxx8rZ6WEl							anonymous	EN	
2023-06-19 9:54	2023-06-19 14:1	IP Address	100	15825	TRUE	2023-06-19 14:1	R_2fIEPbavdXNc1Ck							anonymous	EN	
2023-06-19 14:1	2023-06-19 14:1	IP Address	100	22	TRUE	2023-06-19 14:1	R_1lAUR1ujEiJxRaB							anonymous	EN	
2023-06-19 14:1	2023-06-19 14:1	Spam	100	10	TRUE	2023-06-19 14:1	R_1GVzJuzjsCel84d							anonymous	EN	
2023-06-19 15:4	2023-06-19 15:5	IP Address	100	915	TRUE	2023-06-19 15:5	R_2VPeoAovrP05cRa							anonymous	EN	
2023-06-19 15:5	2023-06-19 16:0	IP Address	100	549	TRUE	2023-06-19 16:0	R_3elj0gwXc8DqSDn							anonymous	EN	
2023-06-19 16:0	2023-06-19 16:0	IP Address	100	115	TRUE	2023-06-19 16:0	R_12ok2JqCmxplRHx							anonymous	EN	
2023-06-20 18:2	2023-06-20 18:2	IP Address	100	104	TRUE	2023-06-20 18:2	R_1JQHVD3g90uYfn4							anonymous	EN	
2023-06-20 18:2	2023-06-20 18:2	IP Address	100	80	TRUE	2023-06-20 18:2	R_BQc0ttts8UaUEkF							anonymous	EN	
2023-06-20 18:2	2023-06-21 16:4	IP Address	100	80591	TRUE	2023-06-21 16:4	R_eJWsJfy5LChUd69							anonymous	EN	
2023-06-21 16:4	2023-06-21 16:5	IP Address	100	218	TRUE	2023-06-21 16:5	R_R7YAHIE2ebvQZtn							anonymous	EN	
2023-06-21 16:5	2023-06-21 16:5	IP Address	100	183	TRUE	2023-06-21 16:5	R_6my56JyCJXfFRlf							anonymous	EN	
2023-06-21 16:5	2023-06-21 17:0	IP Address	100	433	TRUE	2023-06-21 17:0	R_3MJsIdIk0tbgeLG							anonymous	EN	
2023-06-21 17:0	2023-06-21 17:0	IP Address	100	136	TRUE	2023-06-21 17:0	R_42ZIQP8jp1D8VMJ							anonymous	EN	
2023-06-21 17:0	2023-06-21 17:0	IP Address	100	218	TRUE	2023-06-21 17:0	R_pmFpZ91IDzcicw1							anonymous	EN	
2023-06-21 17:0	2023-06-21 17:1	IP Address	100	457	TRUE	2023-06-21 17:1	R_3IRBASFa7RGxUWj							anonymous	EN	
2023-06-22 10:5	2023-06-22 11:1	IP Address	100	1409	TRUE	2023-06-22 11:1	R_9nPC2HHlvE1Rlap							anonymous	EN	
2023-06-22 11:1	2023-06-22 11:1	Spam	100	3	TRUE	2023-06-22 11:1	R_2YWVsoluyFhug2F							anonymous	EN	
2023-06-22 11:1	2023-06-22 11:1	IP Address	100	8	TRUE	2023-06-22 11:1	R_2coUOJ17Zk6EEG7							anonymous	EN	
2023-06-23 9:19	2023-06-23 9:22	IP Address	100	184	TRUE	2023-06-23 9:22	R_UDUcZlQ5b5DgWoV							anonymous	EN	
2023-06-23 9:22	2023-06-23 9:25	IP Address	100	174	TRUE	2023-06-23 9:25	R_2yejMywsufHU0SI							anonymous	EN	
2023-06-23 9:25	2023-06-23 9:26	IP Address	100	83	TRUE	2023-06-23 9:26	R_1hKKOAiqBuciaDr							anonymous	EN	
2023-06-23 9:26	2023-06-23 9:27	IP Address	100	57	TRUE	2023-06-23 9:27	R_1gHJS7ueD61Wlca							anonymous	EN	
2023-06-23 9:27	2023-06-23 9:30	IP Address	100	154	TRUE	2023-06-23 9:30	R_PGp1dwXKugkJxQd							anonymous	EN	
2023-06-23 9:30	2023-06-24 9:58	IP Address	100	88095	TRUE	2023-06-24 9:58	R_1lB7F4veIdwHxrl							anonymous	EN	
2023-06-24 9:58	2023-06-24 10:0	IP Address	100	209	TRUE	2023-06-24 10:0	R_VWjpRJ6UAqfG4RX							anonymous	EN	
2023-06-24 10:0	2023-06-24 10:0	IP Address	100	192	TRUE	2023-06-24 10:0	R_1pGWgKPOy5rvkdc							anonymous	EN	
2023-06-24 10:0	2023-06-24 10:0	IP Address	100	95	TRUE	2023-06-24 10:0	R_231XjN2s8w9X4VW							anonymous	EN	
2023-06-24 10:0	2023-06-24 10:0	IP Address	100	98	TRUE	2023-06-24 10:0	R_6igffHNFa1dVMk1							anonymous	EN	
2023-06-24 15:5	2023-06-24 16:1	IP Address	100	1052	TRUE	2023-06-24 16:1	R_30l7ARwBPjBExJQ							anonymous	EN	
2023-06-24 16:1	2023-06-24 16:2	IP Address	100	322	TRUE	2023-06-24 16:2	R_pzaK1vHKahe1FYt							anonymous	EN	
2023-06-24 16:2	2023-06-24 16:3	IP Address	100	493	TRUE	2023-06-24 16:3	R_2ahpwF6L4kU9650							anonymous	EN	
2023-06-24 16:3	2023-06-24 16:4	IP Address	100	576	TRUE	2023-06-24 16:4	R_3LXScIU4n9KsPCw							anonymous	EN	
2023-06-24 16:4	2023-06-24 16:4	IP Address	100	143	TRUE	2023-06-24 16:4	R_0DHo0neqit56ehH							anonymous	EN	
2023-06-24 16:4	2023-06-24 16:5	IP Address	100	628	TRUE	2023-06-24 16:5	R_1j9YYB9jsudlbIB							anonymous	EN	
2023-06-24 16:5	2023-06-24 17:1	IP Address	100	985	TRUE	2023-06-24 17:1	R_sHGIc4zpCk2lzsR							anonymous	EN	
2023-06-24 17:1	2023-06-24 17:1	IP Address	100	21	TRUE	2023-06-24 17:1	R_1QnBOr3oGsfSzol							anonymous	EN	
2023-06-24 17:1	2023-06-24 17:2	IP Address	100	814	TRUE	2023-06-24 17:2	R_su6pHSaW6UbDdCx							anonymous	EN	
2023-06-22 11:1	2023-06-26 13:4	IP Address	100	354273	TRUE	2023-06-26 13:4	R_umL3rlf6JZxk0ed							anonymous	EN	
2023-06-26 13:4	2023-06-26 14:1	IP Address	100	1631	TRUE	2023-06-26 14:1	R_cMgpj29qoRTJq6Z							anonymous	EN	
2023-06-26 14:1	2023-06-26 14:1	IP Address	100	45	TRUE	2023-06-26 14:1	R_1goKB4Os3C2lRtl							anonymous	EN	
2023-06-29 14:2	2023-06-29 14:2	IP Address	100	348	TRUE	2023-06-29 14:2	R_1GhyG8UBKQqhIWJ							anonymous	EN	
2023-06-29 14:2	2023-06-29 14:3	IP Address	100	176	TRUE	2023-06-29 14:3	R_ZluqVerkpCK364F							anonymous	EN	
2023-06-29 14:3	2023-06-29 14:3	IP Address	100	415	TRUE	2023-06-29 14:3	R_1BXyxwu8uGURkah							anonymous	EN	
2023-06-29 14:3	2023-06-29 15:0	IP Address	100	1670	TRUE	2023-06-29 15:0	R_1JF256GBMkMZ9dw							anonymous	EN	
2023-06-29 15:0	2023-06-29 15:0	IP Address	100	92	TRUE	2023-06-29 15:0	R_3D6YQhNS0ZJuzrj							anonymous	EN	
2023-06-29 15:0	2023-06-29 15:0	IP Address	100	90	TRUE	2023-06-29 15:0	R_UgBreUKrrbsq5Ud							anonymous	EN	
2023-07-01 2:36	2023-07-01 2:37	IP Address	100	74	TRUE	2023-07-01 2:37	R_A73X5B10cFnt6Jr							anonymous	EN	

2023-07-01 2:37	2023-07-01 2:44	IP Address	100	379	TRUE	2023-07-01 2:44	R_1GZgn6VekAWWCad							anonymous	EN	
2023-07-01 2:44	2023-07-01 2:53	IP Address	100	573	TRUE	2023-07-01 2:53	R_SPIOq4DEtxoj5GV							anonymous	EN	
2023-07-01 2:53	2023-07-01 3:02	IP Address	100	554	TRUE	2023-07-01 3:02	R_27vWe0mksuNtIIB							anonymous	EN	
2023-07-01 3:02	2023-07-01 3:05	IP Address	100	147	TRUE	2023-07-01 3:05	R_DS4dJWb26gzSbyp							anonymous	EN	
2023-07-01 3:05	2023-07-01 3:07	IP Address	100	143	TRUE	2023-07-01 3:07	R_1NmGiw2sMs6PykZ							anonymous	EN	
2023-07-01 3:07	2023-07-01 3:09	IP Address	100	117	TRUE	2023-07-01 3:09	R_xsavMBn6yiZY797							anonymous	EN	
2023-07-04 18:1	2023-07-04 18:1	IP Address	100	78	TRUE	2023-07-04 18:1	R_Cl8DjnsnhekXROF							anonymous	EN	
2023-07-05 17:5	2023-07-05 17:5	IP Address	100	192	TRUE	2023-07-05 17:5	R_BWZEyEBRJEajsdz							anonymous	EN	
2023-07-05 17:5	2023-07-05 18:0	IP Address	100	519	TRUE	2023-07-05 18:0	R_3mh1RRp15PpSugr							anonymous	EN	
2023-07-07 14:1	2023-07-07 14:2	IP Address	100	250	TRUE	2023-07-07 14:2	R_2CfhWQ3AinEzII9							anonymous	EN	
2023-07-07 14:2	2023-07-07 14:2	IP Address	100	221	TRUE	2023-07-07 14:2	R_3PFE9ldeWKRLyeL							anonymous	EN	
2023-07-07 14:2	2023-07-07 14:2	IP Address	100	159	TRUE	2023-07-07 14:2	R_1kFSemhE8CduQcd							anonymous	EN	
2023-07-07 14:2	2023-07-07 14:2	IP Address	100	67	TRUE	2023-07-07 14:2	R_ClVjgJpiU9zaluF							anonymous	EN	
2023-07-07 14:2	2023-07-07 14:3	IP Address	100	87	TRUE	2023-07-07 14:3	R_Z2XsIrH73NE0xup							anonymous	EN	
2023-07-07 14:3	2023-07-07 14:3	IP Address	100	407	TRUE	2023-07-07 14:3	R_2aE4Hko8UBu7Mh7							anonymous	EN	
2023-07-07 14:3	2023-07-07 14:4	IP Address	100	173	TRUE	2023-07-07 14:4	R_6mnwSPUonvXfedb							anonymous	EN	
2023-07-07 14:4	2023-07-07 15:0	IP Address	100	1279	TRUE	2023-07-07 15:0	R_5zFdwB2ojsdVlst							anonymous	EN	
2023-07-07 15:0	2023-07-07 15:0	IP Address	100	203	TRUE	2023-07-07 15:0	R_31iTasTDNXaaoam							anonymous	EN	
2023-07-07 15:0	2023-07-07 15:0	IP Address	100	53	TRUE	2023-07-07 15:0	R_2uQVKc5pf69YhYa							anonymous	EN	
2023-07-07 15:0	2023-07-07 15:0	IP Address	100	91	TRUE	2023-07-07 15:0	R_9SvnCWnqOADFa4V							anonymous	EN	
2023-07-07 15:0	2023-07-07 15:1	IP Address	100	350	TRUE	2023-07-07 15:1	R_3peS0zPDHbHH0IC							anonymous	EN	
2023-07-07 15:1	2023-07-07 15:1	IP Address	100	261	TRUE	2023-07-07 15:1	R_db8OKXm27GOWRbP							anonymous	EN	
2023-07-07 15:1	2023-07-07 15:2	IP Address	100	254	TRUE	2023-07-07 15:2	R_p4yICHfoZsZdpZL							anonymous	EN	
2023-07-07 15:2	2023-07-07 15:2	IP Address	100	174	TRUE	2023-07-07 15:2	R_2X0NUREFd1vjvOr							anonymous	EN	
2023-07-07 15:2	2023-07-07 16:0	IP Address	100	2406	TRUE	2023-07-07 16:0	R_0qxSbbDjhdzpJu1							anonymous	EN	
2023-07-07 16:0	2023-07-07 16:1	IP Address	100	619	TRUE	2023-07-07 16:1	R_25RgRm8d6s2C9v8							anonymous	EN	
2023-07-07 16:1	2023-07-07 16:2	IP Address	100	176	TRUE	2023-07-07 16:2	R_RC6JkeceaSfkdSF							anonymous	EN	
2023-07-07 16:2	2023-07-07 16:2	IP Address	100	273	TRUE	2023-07-07 16:2	R_AmSu00idtjBDqzD							anonymous	EN	
2023-07-04 13:1	2023-07-04 13:1	IP Address	83	60	FALSE	2023-07-11 13:1	R_2BbvTpj81dbnhHP							anonymous	EN	
2023-07-04 18:1	2023-07-04 18:2	IP Address	83	733	FALSE	2023-07-11 18:2	R_1mn51aI89l0WqRO							anonymous	EN	
2023-07-12 14:1	2023-07-12 14:1	IP Address	100	65	TRUE	2023-07-12 14:1	R_3j3Cbyxqbk1kNhO							anonymous	EN	
2023-07-12 14:1	2023-07-12 14:1	IP Address	100	58	TRUE	2023-07-12 14:1	R_9RD0VB4oxlOTgtP							anonymous	EN	
2023-07-12 14:1	2023-07-12 14:2	IP Address	100	103	TRUE	2023-07-12 14:2	R_2vkxGnCGTVsfcnG							anonymous	EN	
2023-07-12 14:2	2023-07-12 14:2	IP Address	100	145	TRUE	2023-07-12 14:2	R_29UxJCAD3Umnslj							anonymous	EN	
2023-07-12 14:2	2023-07-12 14:4	IP Address	100	1061	TRUE	2023-07-12 14:4	R_1HjEPwhfnTcvXyO							anonymous	EN	
2023-07-12 14:4	2023-07-12 15:0	IP Address	100	1205	TRUE	2023-07-12 15:0	R_1jOJrzg6rpDWUwl							anonymous	EN	
2023-07-12 15:0	2023-07-12 15:0	IP Address	100	66	TRUE	2023-07-12 15:0	R_2ypDHXHfTiHfsaI							anonymous	EN	
2023-07-12 15:0	2023-07-12 15:0	IP Address	100	66	TRUE	2023-07-12 15:0	R_1n7Qn01GvMvgeIn							anonymous	EN	
2023-07-12 15:0	2023-07-12 15:0	IP Address	100	84	TRUE	2023-07-12 15:0	R_3oZaBF6bGSbvb3f							anonymous	EN	
2023-07-15 12:2	2023-07-15 12:3	IP Address	100	167	TRUE	2023-07-15 12:3	R_6MaA1BlmNutHYrf							anonymous	EN	
2023-07-15 12:3	2023-07-15 12:3	IP Address	100	96	TRUE	2023-07-15 12:3	R_2WJ8X1WeFU0js9r							anonymous	EN	
2023-07-15 12:3	2023-07-15 12:3	IP Address	100	91	TRUE	2023-07-15 12:3	R_57kqvFw6rM7bo1b							anonymous	EN	
2023-07-15 12:3	2023-07-15 12:3	IP Address	100	127	TRUE	2023-07-15 12:3	R_1P7uvD1kg6KgPKQ							anonymous	EN	
2023-07-15 12:3	2023-07-15 12:4	IP Address	100	117	TRUE	2023-07-15 12:4	R_1giv2Upj0BtNSpO							anonymous	EN	
2023-07-15 12:4	2023-07-15 13:0	IP Address	100	1359	TRUE	2023-07-15 13:0	R_3kbRXihidzoTthD							anonymous	EN	
2023-07-15 13:0	2023-07-15 13:1	IP Address	100	481	TRUE	2023-07-15 13:1	R_3dYSSZ8sjJZwhgr							anonymous	EN	
2023-07-15 13:1	2023-07-15 13:1	IP Address	100	487	TRUE	2023-07-15 13:1	R_ssXkEChxv0Ngjmh							anonymous	EN	
2023-07-15 18:1	2023-07-15 18:2	IP Address	100	348	TRUE	2023-07-15 18:2	R_1o1KvfIF0HakVZi							anonymous	EN	
2023-07-20 16:3	2023-07-20 16:3	IP Address	100	91	TRUE	2023-07-20 16:3	R_u1yOFOm8YGCqufn							anonymous	EN	
2023-07-20 16:3	2023-07-20 16:3	IP Address	100	104	TRUE	2023-07-20 16:3	R_3sbmSbbX8Ge5b8I							anonymous	EN	
2023-07-20 16:3	2023-07-20 16:4	IP Address	100	140	TRUE	2023-07-20 16:4	R_Wfe6xEsF24kMRa1							anonymous	EN	
2023-07-20 16:4	2023-07-20 16:4	Spam	100	83	TRUE	2023-07-20 16:4	R_2ea7OoO9OB3rhTa							anonymous	EN	
2023-07-20 16:4	2023-07-20 16:4	IP Address	100	82	TRUE	2023-07-20 16:4	R_3P7WBSvj3lPFngN							anonymous	EN	
2023-07-20 16:4	2023-07-20 16:4	IP Address	100	124	TRUE	2023-07-20 16:4	R_2YmPe0wJbcXtE5Y							anonymous	EN	
2023-07-20 16:4	2023-07-20 16:5	IP Address	100	180	TRUE	2023-07-20 16:5	R_1M05vUGXbZbrRbE							anonymous	EN	
2023-07-20 16:5	2023-07-20 16:5	IP Address	100	186	TRUE	2023-07-20 16:5	R_ZKU2iM6CscdmTkJ							anonymous	EN	
2023-07-20 16:5	2023-07-20 16:5	IP Address	100	134	TRUE	2023-07-20 16:5	R_1ezbbmqGeOzbejs							anonymous	EN	
2023-07-20 16:5	2023-07-20 16:5	IP Address	100	140	TRUE	2023-07-20 16:5	R_1JD7YEvHcQ9iXpJ							anonymous	EN	
2023-07-20 16:5	2023-07-20 16:5	IP Address	100	63	TRUE	2023-07-20 16:5	R_V3IyJcD7dK3MxX3							anonymous	EN	

2023-07-20 16:5	2023-07-20 17:0	IP Address	100	142	TRUE	2023-07-20 17:0	R_1PTbvNuktTEt3CV						anonymous	EN	
2023-07-20 17:0	2023-07-20 17:0	IP Address	100	85	TRUE	2023-07-20 17:0	R_3nPhWVxDnEbmdmk						anonymous	EN	
2023-07-20 17:0	2023-07-20 17:0	IP Address	100	306	TRUE	2023-07-20 17:0	R_UaQJ48WT2Xyl31v						anonymous	EN	
2023-07-20 17:0	2023-07-20 17:0	IP Address	100	57	TRUE	2023-07-20 17:0	R_1JPjtmXXsuWiEOe						anonymous	EN	
2023-07-20 17:0	2023-07-20 17:0	IP Address	100	48	TRUE	2023-07-20 17:0	R_3iPFn0GjORn5MSe						anonymous	EN	
2023-07-21 14:2	2023-07-21 14:2	IP Address	100	101	TRUE	2023-07-21 14:2	R_301ySJRwPirp371						anonymous	EN	
2023-07-21 14:2	2023-07-21 14:2	IP Address	100	81	TRUE	2023-07-21 14:2	R_3Jfl6aYkDVt7CSp						anonymous	EN	
2023-07-21 14:2	2023-07-21 14:2	IP Address	100	56	TRUE	2023-07-21 14:2	R_2YzzJCRzeJNmIso						anonymous	EN	
2023-07-21 14:2	2023-07-21 14:2	IP Address	100	113	TRUE	2023-07-21 14:2	R_bsA2bHvGnXrM8Zb						anonymous	EN	
2023-07-21 15:3	2023-07-21 15:3	IP Address	100	175	TRUE	2023-07-21 15:3	R_2v78VjKAi7AJa8z						anonymous	EN	
2023-07-21 15:4	2023-07-21 15:4	IP Address	100	29	TRUE	2023-07-21 15:4	R_2pLglJh25gzeIjL						anonymous	EN	
2023-07-21 15:4	2023-07-21 15:4	IP Address	100	172	TRUE	2023-07-21 15:4	R_CfYoFb09unLAp8Z						anonymous	EN	
2023-07-21 15:4	2023-07-21 15:4	IP Address	100	81	TRUE	2023-07-21 15:4	R_3NE55DwaccrVPw9						anonymous	EN	
2023-07-21 15:4	2023-07-21 15:5	IP Address	100	393	TRUE	2023-07-21 15:5	R_3J2eEVDfqRGvgH9						anonymous	EN	
2023-07-21 15:5	2023-07-21 16:0	IP Address	100	641	TRUE	2023-07-21 16:0	R_2f8xnZdmvgg9KfQ						anonymous	EN	
2023-07-21 16:0	2023-07-21 16:0	IP Address	100	116	TRUE	2023-07-21 16:0	R_3e1T9NRQSDRMbd2						anonymous	EN	
2023-07-21 16:0	2023-07-21 16:0	IP Address	100	212	TRUE	2023-07-21 16:0	R_1BRgVWSLYt9sUcW						anonymous	EN	
2023-07-21 16:0	2023-07-21 16:1	IP Address	100	180	TRUE	2023-07-21 16:1	R_2QVsIOKl8OcWi81						anonymous	EN	
2023-07-25 3:35	2023-07-25 3:37	IP Address	100	111	TRUE	2023-07-25 3:37	R_24NavbI87XIIasI						anonymous	EN	
2023-07-25 3:37	2023-07-25 3:39	IP Address	100	110	TRUE	2023-07-25 3:39	R_2t3CfL1Zdi5Ct9D						anonymous	EN	
2023-07-25 3:39	2023-07-25 3:41	IP Address	100	116	TRUE	2023-07-25 3:41	R_1NeKq5QEC7vn03x						anonymous	EN	
2023-07-25 3:41	2023-07-25 3:43	IP Address	100	139	TRUE	2023-07-25 3:43	R_24LW6VctWJJ5Aoy						anonymous	EN	
2023-07-25 3:44	2023-07-25 3:48	IP Address	100	246	TRUE	2023-07-25 3:48	R_1n9CbJqicYJ6zbI						anonymous	EN	
2023-07-27 3:55	2023-07-27 4:05	IP Address	100	594	TRUE	2023-07-27 4:05	R_2fuHAgDQpaWcPXY						anonymous	EN	
2023-07-27 4:05	2023-07-27 4:06	IP Address	100	49	TRUE	2023-07-27 4:06	R_sFt7hOVba8CGWqZ						anonymous	EN	
2023-07-27 4:06	2023-07-27 4:07	IP Address	100	72	TRUE	2023-07-27 4:07	R_30cNCfFqGtj06CS						anonymous	EN	
2023-07-27 4:08	2023-07-27 4:09	IP Address	100	76	TRUE	2023-07-27 4:09	R_3GDtyDCqLL9kX7Z						anonymous	EN	
2023-07-27 4:09	2023-07-27 4:10	IP Address	100	42	TRUE	2023-07-27 4:10	R_3iEwox203yjMYku						anonymous	EN	
2023-07-27 4:10	2023-07-27 4:11	IP Address	100	93	TRUE	2023-07-27 4:11	R_1hEjd9RpJXYn6Rr						anonymous	EN	
2023-07-27 4:11	2023-07-27 4:13	IP Address	100	81	TRUE	2023-07-27 4:13	R_30680TJACXvybqq						anonymous	EN	
2023-07-28 2:58	2023-07-28 3:02	IP Address	100	262	TRUE	2023-07-28 3:03	R_1JOBIEKl2hHSNcU						anonymous	EN	
2023-07-28 3:03	2023-07-28 3:03	IP Address	100	52	TRUE	2023-07-28 3:03	R_27PsGcbCwvaGXZl						anonymous	EN	
2023-07-28 3:03	2023-07-28 3:05	IP Address	100	75	TRUE	2023-07-28 3:05	R_V2va129FldcSGnn						anonymous	EN	
2023-07-28 3:05	2023-07-28 3:09	IP Address	100	234	TRUE	2023-07-28 3:09	R_2ygfvpsxHPR2mqx						anonymous	EN	
2023-07-28 17:4	2023-07-28 17:5	IP Address	100	299	TRUE	2023-07-28 17:5	R_3lsOxX9W8MXBHzC						anonymous	EN	
2023-07-28 17:5	2023-07-28 17:5	IP Address	100	60	TRUE	2023-07-28 17:5	R_5oNhrQsP0XI51nP						anonymous	EN	
2023-07-28 17:5	2023-07-28 17:5	IP Address	100	132	TRUE	2023-07-28 17:5	R_1CpiuYYLAJPwsuN						anonymous	EN	
2023-07-28 17:5	2023-07-28 17:5	IP Address	100	75	TRUE	2023-07-28 17:5	R_2zT0oTs9eH5n1NC						anonymous	EN	
2023-07-28 17:5	2023-07-28 17:5	IP Address	100	116	TRUE	2023-07-28 17:5	R_bfKWMc6Dx4nGDv3						anonymous	EN	
2023-07-28 17:5	2023-07-28 18:0	IP Address	100	123	TRUE	2023-07-28 18:0	R_1DZ6wXJlaHaKqoU						anonymous	EN	
2023-07-28 18:0	2023-07-28 18:0	IP Address	100	340	TRUE	2023-07-28 18:0	R_27qErdM2RvuZQCC						anonymous	EN	
2023-07-28 18:0	2023-07-28 18:0	IP Address	100	59	TRUE	2023-07-28 18:0	R_2qvXw8wQZJ80umq						anonymous	EN	
2023-07-28 18:0	2023-07-28 18:2	IP Address	100	988	TRUE	2023-07-28 18:2	R_cLN8Xm21Il3tbxL						anonymous	EN	
2023-08-02 10:2	2023-08-02 10:3	IP Address	100	556	TRUE	2023-08-02 10:3	R_dbZ8KdgQQvOLaLL						anonymous	EN	
2023-08-02 10:3	2023-08-02 10:5	IP Address	100	865	TRUE	2023-08-02 10:5	R_27QdwbyhI2zggxX						anonymous	EN	
2023-08-02 10:5	2023-08-02 11:2	IP Address	100	2032	TRUE	2023-08-02 11:2	R_2yq1vYDRk57DJq6						anonymous	EN	
2023-08-02 11:2	2023-08-02 11:2	IP Address	100	75	TRUE	2023-08-02 11:2	R_1hMeU9py1TBfIWq						anonymous	EN	
2023-08-02 11:2	2023-08-02 12:0	IP Address	100	2313	TRUE	2023-08-02 12:0	R_vwANw3DJU8YOjiF						anonymous	EN	
2023-08-02 12:0	2023-08-02 12:1	IP Address	100	235	TRUE	2023-08-02 12:1	R_3OavQ3fpLzXx7IR						anonymous	EN	
2023-08-02 12:1	2023-08-02 12:1	IP Address	100	94	TRUE	2023-08-02 12:1	R_32LqL9d3rG0fLTB						anonymous	EN	
2023-08-04 8:36	2023-08-04 8:38	IP Address	100	116	TRUE	2023-08-04 8:38	R_2zk0b2tOAXdyfWQ						anonymous	EN	
2023-08-04 8:38	2023-08-04 8:40	IP Address	100	138	TRUE	2023-08-04 8:40	R_9srqWTdbSLBBrs5						anonymous	EN	
2023-08-04 8:40	2023-08-04 8:43	IP Address	100	157	TRUE	2023-08-04 8:43	R_D00IUtkDi4MWQcp						anonymous	EN	
2023-08-04 10:0	2023-08-04 10:0	IP Address	100	49	TRUE	2023-08-04 10:0	R_3I3Rtif4GUOLmYv						anonymous	EN	
2023-08-06 19:0	2023-08-06 19:0	IP Address	100	142	TRUE	2023-08-06 19:0	R_2rk2964bsQVYRdq						anonymous	EN	
2023-08-06 19:0	2023-08-06 19:0	IP Address	100	143	TRUE	2023-08-06 19:0	R_2timJ1OLOwfEXSR						anonymous	EN	
2023-08-10 21:3	2023-08-10 21:4	IP Address	100	221	TRUE	2023-08-10 21:4	R_1IB0xkR7ScR9gQG						anonymous	EN	
2023-08-10 21:4	2023-08-10 21:4	IP Address	100	27	TRUE	2023-08-10 21:4	R_2ANl2qd3ri3dOJk						anonymous	EN	
2023-08-10 21:4	2023-08-10 21:5	IP Address	100	671	TRUE	2023-08-10 21:5	R_31mVEhrKNnJFiEC						anonymous	EN	

2023-08-10 22:0	2023-08-10 22:1	IP Address	100	445	TRUE	2023-08-10 22:1	R_1Ka9PwzyZUBzXA2						anonymous	EN	
2023-08-10 22:2	2023-08-10 22:2	IP Address	100	62	TRUE	2023-08-10 22:2	R_1pEbij37NDGspaq						anonymous	EN	
2023-08-11 0:31:	2023-08-11 0:45:	IP Address	100	816	TRUE	2023-08-11 0:45:	R_0CZ4SPnQZPw8Edr						anonymous	EN	
2023-08-11 1:20:	2023-08-11 1:38:	IP Address	100	1043	TRUE	2023-08-11 1:38:	R_2ONOTYHHteEzouz						anonymous	EN	
2023-08-11 1:43:	2023-08-11 1:52:	IP Address	100	537	TRUE	2023-08-11 1:52:	R_2DRNoWDNZlbejL8						anonymous	EN	
2023-08-11 18:3	2023-08-11 18:3	IP Address	100	118	TRUE	2023-08-11 18:3	R_2sZ6SohvZ1W3APB						anonymous	EN	
2023-08-11 18:3	2023-08-11 18:4	IP Address	100	173	TRUE	2023-08-11 18:4	R_31GhzyeU9wcNYTi						anonymous	EN	
2023-08-11 18:4	2023-08-11 18:4	IP Address	100	73	TRUE	2023-08-11 18:4	R_1l9TN6DeGIMy48d						anonymous	EN	
2023-08-11 18:4	2023-08-11 18:4	IP Address	100	109	TRUE	2023-08-11 18:4	R_3PiXNbLkxhop0UO						anonymous	EN	
2023-08-11 18:4	2023-08-11 19:0	IP Address	100	1203	TRUE	2023-08-11 19:0	R_vSk5w11rubMbqfL						anonymous	EN	
2023-08-11 19:0	2023-08-11 19:0	IP Address	100	268	TRUE	2023-08-11 19:0	R_1CKeWOIzy2RmvWl						anonymous	EN	
2023-08-12 11:4	2023-08-12 11:5	IP Address	100	833	TRUE	2023-08-12 11:5	R_8oJFsAC4LU8WQtX						anonymous	EN	
2023-08-14 19:4	2023-08-14 19:4	IP Address	100	116	TRUE	2023-08-14 19:4	R_2XgrYkZ6F5CGPCA						anonymous	EN	
2023-08-14 19:4	2023-08-14 19:4	IP Address	100	71	TRUE	2023-08-14 19:4	R_2tJa4x1rJm2HSuy						anonymous	EN	
2023-08-14 19:4	2023-08-14 20:0	IP Address	100	1002	TRUE	2023-08-14 20:0	R_3J3ihgGx4uxBLXd						anonymous	EN	
2023-08-14 20:0	2023-08-14 20:0	IP Address	100	63	TRUE	2023-08-14 20:0	R_e40pYRKdQh9nbcR						anonymous	EN	
2023-08-15 16:1	2023-08-15 16:1	IP Address	100	410	TRUE	2023-08-15 16:1	R_1E6St6hUcagrcFg						anonymous	EN	
2023-08-17 15:5	2023-08-17 15:5	IP Address	100	75	TRUE	2023-08-17 15:5	R_3qL7CvKExvGO9ey						anonymous	EN	
2023-08-17 15:5	2023-08-17 15:5	IP Address	100	123	TRUE	2023-08-17 15:5	R_2c75BnNq0kxCoXF						anonymous	EN	
2023-08-17 15:5	2023-08-17 16:0	IP Address	100	103	TRUE	2023-08-17 16:0	R_2f98n2YuKe9gVyP						anonymous	EN	
2023-08-18 1:24:	2023-08-18 1:29:	IP Address	100	315	TRUE	2023-08-18 1:29:	R_3lAnj2KL2biXVFe						anonymous	EN	
2023-08-18 1:29:	2023-08-18 1:30:	IP Address	100	49	TRUE	2023-08-18 1:30:	R_2rqqTV1Hwi5n56K						anonymous	EN	
2023-08-18 1:31:	2023-08-18 1:32:	IP Address	100	24	TRUE	2023-08-18 1:32:	R_38bapacFMfBjHix						anonymous	EN	
2023-08-18 1:32:	2023-08-18 1:33:	IP Address	100	59	TRUE	2023-08-18 1:33:	R_2cw5XbWi948wRKJ						anonymous	EN	
2023-08-18 1:33:	2023-08-18 1:42:	IP Address	100	553	TRUE	2023-08-18 1:42:	R_bvVw8CIz8V432ox						anonymous	EN	
2023-08-18 1:42:	2023-08-18 1:45:	IP Address	100	188	TRUE	2023-08-18 1:45:	R_3078Ak9TWofzFeO						anonymous	EN	
2023-08-18 1:45:	2023-08-18 1:59:	IP Address	100	835	TRUE	2023-08-18 1:59:	R_3l4UixSjEd6zGM5						anonymous	EN	
2023-08-18 1:59:	2023-08-18 2:15:	IP Address	100	941	TRUE	2023-08-18 2:15:	R_1CHlg2kvkmS8vmw						anonymous	EN	
2023-08-18 13:4	2023-08-18 13:4	IP Address	100	31	TRUE	2023-08-18 13:4	R_sMuLJo6vlCJ86bL						anonymous	EN	
2023-08-18 13:5	2023-08-18 13:5	IP Address	100	30	TRUE	2023-08-18 13:5	R_1Fg52gpal2rXh5H						anonymous	EN	
2023-08-18 13:5	2023-08-18 13:5	IP Address	100	282	TRUE	2023-08-18 13:5	R_2woQKSffx7bFzCz						anonymous	EN	
2023-08-18 13:5	2023-08-18 14:0	IP Address	100	184	TRUE	2023-08-18 14:0	R_3NCVBNed6iQcT8q						anonymous	EN	
2023-08-18 14:0	2023-08-18 14:0	IP Address	100	119	TRUE	2023-08-18 14:0	R_23gJZkAVJ7HlbJ8						anonymous	EN	
2023-08-18 14:0	2023-08-18 14:0	IP Address	100	321	TRUE	2023-08-18 14:0	R_27HyEznTewXafXQ						anonymous	EN	
2023-08-18 14:0	2023-08-18 14:1	IP Address	100	381	TRUE	2023-08-18 14:1	R_1f7ntme5AYKkPp9						anonymous	EN	
2023-08-18 14:1	2023-08-18 14:3	IP Address	100	848	TRUE	2023-08-18 14:3	R_3foVygZiA4k2cRq						anonymous	EN	
2023-08-18 14:3	2023-08-18 14:3	IP Address	100	145	TRUE	2023-08-18 14:3	R_2xQxxZqJpNrR1XA						anonymous	EN	
2023-08-15 16:1	2023-08-18 14:4	IP Address	100	253333	TRUE	2023-08-18 14:4	R_UzNcvGvdODZhzDr						anonymous	EN	
2023-08-18 15:0	2023-08-18 15:1	IP Address	100	870	TRUE	2023-08-18 15:1	R_3nDIOfRnpvtfHHv						anonymous	EN	
2023-08-18 15:1	2023-08-18 15:3	IP Address	100	1086	TRUE	2023-08-18 15:3	R_PTX8Hn3jPw1k0s9						anonymous	EN	
2023-08-18 15:3	2023-08-18 15:5	IP Address	100	1236	TRUE	2023-08-18 15:5	R_Od5HYFNTpoBJORX						anonymous	EN	
2023-08-18 15:5	2023-08-18 17:2	IP Address	100	5091	TRUE	2023-08-18 17:2	R_Z4PuQgGbft1sJcl						anonymous	EN	
2023-08-18 17:2	2023-08-18 17:2	IP Address	100	298	TRUE	2023-08-18 17:2	R_2yeEn4gU6VFxPCh						anonymous	EN	
2023-08-18 17:3	2023-08-18 17:3	IP Address	100	21	TRUE	2023-08-18 17:3	R_2v5Dq9IVCTc3q1w						anonymous	EN	
2023-08-18 20:2	2023-08-18 23:4	IP Address	100	12295	TRUE	2023-08-18 23:4	R_8hNBtYwuLlqOBrP						anonymous	EN	
2023-08-18 23:4	2023-08-19 0:50:	IP Address	100	3701	TRUE	2023-08-19 0:50:	R_1QoUzQdVmL044Kg						anonymous	EN	
2023-08-19 0:53:	2023-08-19 1:35:	IP Address	100	2502	TRUE	2023-08-19 1:35:	R_0xfqiDiXsxx9h4t						anonymous	EN	
2023-08-19 1:37:	2023-08-19 1:39:	IP Address	100	156	TRUE	2023-08-19 1:39:	R_12JUY30HYKkIx1j						anonymous	EN	
2023-08-19 1:43:	2023-08-19 1:43:	IP Address	100	15	TRUE	2023-08-19 1:43:	R_3gZedKL9QfGZkzW						anonymous	EN	
2023-08-19 1:46:	2023-08-19 1:47:	IP Address	100	13	TRUE	2023-08-19 1:47:	R_1Ks9nUdsOSZb8MX						anonymous	EN	
2023-08-17 16:0	2023-08-20 10:3	IP Address	100	239519	TRUE	2023-08-20 10:3	R_3dGPAdhGrlv9WIl						anonymous	EN	
2023-08-20 10:3	2023-08-20 10:3	IP Address	100	39	TRUE	2023-08-20 10:3	R_2P5zwF8NYGcPs0O						anonymous	EN	
2023-08-20 10:3	2023-08-20 10:3	IP Address	100	79	TRUE	2023-08-20 10:3	R_WoL7lLvlB3Apuhj						anonymous	EN	
2023-08-20 10:3	2023-08-20 10:3	IP Address	100	102	TRUE	2023-08-20 10:3	R_Oxxeh159ylOaHvz						anonymous	EN	
2023-08-20 10:3	2023-08-20 10:3	IP Address	100	101	TRUE	2023-08-20 10:3	R_2qkjP0HkUrlIxlo						anonymous	EN	
2023-08-20 10:3	2023-08-20 11:2	IP Address	100	2621	TRUE	2023-08-20 11:2	R_1N4ccZnpxoQNbg2						anonymous	EN	
2023-08-20 11:2	2023-08-20 11:2	IP Address	100	61	TRUE	2023-08-20 11:2	R_2wNOnzKW4oVfwdP						anonymous	EN	
2023-08-20 11:2	2023-08-20 11:2	IP Address	100	67	TRUE	2023-08-20 11:2	R_bmBrTCI2w3tHD3j						anonymous	EN	
2023-08-20 11:2	2023-08-20 11:2	IP Address	100	70	TRUE	2023-08-20 11:2	R_21j6eONunK58fET						anonymous	EN	

2023-08-20 20:2	2023-08-20 21:0	IP Address	100	2232	TRUE	2023-08-20 21:0	R_2ZJPBrStuG4rcOX							anonymous	EN	
2023-08-20 21:0	2023-08-20 21:0	IP Address	100	517	TRUE	2023-08-20 21:0	R_29i6zwSRHFtsg6B							anonymous	EN	
2023-08-20 21:1	2023-08-20 23:0	IP Address	100	6445	TRUE	2023-08-20 23:0	R_1LLl3gixMbxNGOl							anonymous	EN	
2023-08-20 23:0	2023-08-20 23:2	IP Address	100	765	TRUE	2023-08-20 23:2	R_xhjOwxF5BOyPRnj							anonymous	EN	
2023-08-20 23:2	2023-08-20 23:2	IP Address	100	119	TRUE	2023-08-20 23:2	R_30oTw3QS7IEIkP8							anonymous	EN	
2023-08-20 23:3	2023-08-21 1:59:	IP Address	100	8852	TRUE	2023-08-21 1:59:	R_2YJqV4Wo4gQ8D5f							anonymous	EN	
2023-08-21 2:26:	2023-08-21 2:50:	IP Address	100	1441	TRUE	2023-08-21 2:50:	R_3plRn7CFn2GRD5v							anonymous	EN	
2023-08-21 2:50:	2023-08-21 14:4	IP Address	100	42671	TRUE	2023-08-21 14:4	R_1dBu7gaw9ldTb06							anonymous	EN	
2023-08-18 2:15:	2023-08-21 23:1	IP Address	100	334843	TRUE	2023-08-21 23:1	R_2lyIMk4nAkYK90d							anonymous	EN	
2023-08-21 23:1	2023-08-21 23:1	IP Address	100	101	TRUE	2023-08-21 23:1	R_3Mbzy3ZqQ8Xl9Mf							anonymous	EN	
2023-08-21 23:1	2023-08-21 23:1	IP Address	100	55	TRUE	2023-08-21 23:1	R_3KqJyiqVOSZexLx							anonymous	EN	
2023-08-21 23:1	2023-08-21 23:2	IP Address	100	218	TRUE	2023-08-21 23:2	R_72jRiN7lb8KZufL							anonymous	EN	
2023-08-21 23:2	2023-08-21 23:2	IP Address	100	375	TRUE	2023-08-21 23:2	R_1o6kQ6TPsJ87O9S							anonymous	EN	
2023-08-21 23:2	2023-08-21 23:3	IP Address	100	134	TRUE	2023-08-21 23:3	R_2CxefRqk61f8fkU							anonymous	EN	
2023-08-21 23:3	2023-08-21 23:3	IP Address	100	411	TRUE	2023-08-21 23:3	R_3NPLm2qhcStJup9							anonymous	EN	
2023-08-21 23:3	2023-08-21 23:4	IP Address	100	204	TRUE	2023-08-21 23:4	R_XMK9bewniTZGuCR							anonymous	EN	
2023-08-21 14:4	2023-08-21 23:4	IP Address	100	32813	TRUE	2023-08-21 23:4	R_NVDyzUjkemNbaIV							anonymous	EN	
2023-08-21 23:4	2023-08-21 23:5	IP Address	100	592	TRUE	2023-08-21 23:5	R_2QLC9YiUZtkMAkW							anonymous	EN	
2023-08-21 23:5	2023-08-21 23:5	IP Address	100	310	TRUE	2023-08-21 23:5	R_2YV4ZiOBFLhGaN1							anonymous	EN	
2023-08-21 23:5	2023-08-21 23:5	IP Address	100	171	TRUE	2023-08-21 23:5	R_3PZfBH52cWH0pAz							anonymous	EN	
2023-08-21 23:5	2023-08-22 0:06:	IP Address	100	416	TRUE	2023-08-22 0:06:	R_OJ4A4i5ed0k88O5							anonymous	EN	
2023-08-22 0:06:	2023-08-22 0:21:	IP Address	100	887	TRUE	2023-08-22 0:21:	R_3ncMj0t5ap3fZiX							anonymous	EN	
2023-08-21 23:4	2023-08-22 1:08:	IP Address	100	4756	TRUE	2023-08-22 1:08:	R_Y0sLQtzR9G1Qrtv							anonymous	EN	
2023-08-22 1:11:	2023-08-22 1:14:	IP Address	100	193	TRUE	2023-08-22 1:14:	R_DdYTtEzysmIgqvn							anonymous	EN	
2023-08-22 1:14:	2023-08-22 1:15:	IP Address	100	41	TRUE	2023-08-22 1:15:	R_1rceJmi4MkX6pJH							anonymous	EN	
2023-08-22 0:21:	2023-08-22 1:16:	IP Address	100	3288	TRUE	2023-08-22 1:16:	R_3R3hajtz5Vzr1xo							anonymous	EN	
2023-08-22 1:15:	2023-08-22 1:21:	IP Address	100	358	TRUE	2023-08-22 1:21:	R_1JEggtv1QIRJsW1							anonymous	EN	
2023-08-22 1:21:	2023-08-22 1:21:	IP Address	100	37	TRUE	2023-08-22 1:21:	R_2YhWkmMNfJ7lJzQ							anonymous	EN	
2023-08-22 1:21:	2023-08-22 1:31:	IP Address	100	557	TRUE	2023-08-22 1:31:	R_57RIyIjdraYZAJP							anonymous	EN	
2023-08-22 1:31:	2023-08-22 1:35:	IP Address	100	234	TRUE	2023-08-22 1:35:	R_3CCtaf8nvkdSG4e							anonymous	EN	
2023-08-22 1:35:	2023-08-22 1:40:	IP Address	100	343	TRUE	2023-08-22 1:40:	R_2Cqs1smqFHRFsTC							anonymous	EN	
2023-08-22 1:42:	2023-08-22 2:24:	IP Address	100	2531	TRUE	2023-08-22 2:24:	R_1NEob7vpX0lNelv							anonymous	EN	
2023-08-22 2:24:	2023-08-22 2:32:	IP Address	100	457	TRUE	2023-08-22 2:32:	R_2CPzNPS0l3l8r4D							anonymous	EN	
2023-08-22 2:32:	2023-08-22 2:42:	IP Address	100	641	TRUE	2023-08-22 2:42:	R_12o87gZrLL9YdeV							anonymous	EN	
2023-08-22 2:44:	2023-08-22 2:47:	IP Address	100	139	TRUE	2023-08-22 2:47:	R_1FEumfDCjOMq5lU							anonymous	EN	
2023-08-22 2:47:	2023-08-22 14:4	IP Address	100	43005	TRUE	2023-08-22 14:4	R_2y79Nkx2R2NvA2M							anonymous	EN	
2023-08-22 14:4	2023-08-22 14:5	IP Address	100	802	TRUE	2023-08-22 14:5	R_3suyBLAH22Plvtn							anonymous	EN	
2023-08-22 15:0	2023-08-22 15:0	IP Address	100	27	TRUE	2023-08-22 15:0	R_SITr0MvwAZtem1H							anonymous	EN	
2023-08-22 15:0	2023-08-22 15:1	IP Address	100	211	TRUE	2023-08-22 15:1	R_6RkMIKXdvJhmolb							anonymous	EN	
2023-08-22 15:1	2023-08-22 15:1	IP Address	100	262	TRUE	2023-08-22 15:1	R_6Ry8c6vjJ5mwrPX							anonymous	EN	
2023-08-22 15:1	2023-08-22 17:1	IP Address	100	7038	TRUE	2023-08-22 17:1	R_1FL1ZDNvhMT5lPP							anonymous	EN	
2023-08-22 17:1	2023-08-22 17:2	IP Address	100	551	TRUE	2023-08-22 17:2	R_prNQcPhaBoAdKb7							anonymous	EN	
2023-08-22 17:2	2023-08-22 17:2	IP Address	100	172	TRUE	2023-08-22 17:2	R_2rjk1lWTD3LOmc1							anonymous	EN	
2023-08-22 17:2	2023-08-22 17:3	IP Address	100	417	TRUE	2023-08-22 17:3	R_2WD21l4HdNoNhct							anonymous	EN	
2023-08-22 17:3	2023-08-22 17:4	IP Address	100	817	TRUE	2023-08-22 17:4	R_21bJvxH3fQAEMGA							anonymous	EN	
2023-08-22 17:4	2023-08-22 20:1	IP Address	100	8879	TRUE	2023-08-22 20:1	R_5jcWkl6xOrNVNfP							anonymous	EN	
2023-08-22 20:1	2023-08-22 20:1	IP Address	100	225	TRUE	2023-08-22 20:1	R_PLQexwYPRUJuPNT							anonymous	EN	
2023-08-22 20:1	2023-08-22 20:2	IP Address	100	395	TRUE	2023-08-22 20:2	R_3qDtdxqyUtFiVcw							anonymous	EN	
2023-08-22 20:2	2023-08-22 20:4	IP Address	100	1159	TRUE	2023-08-22 20:4	R_2PoGliFWTiRIYEY							anonymous	EN	
2023-08-22 20:5	2023-08-22 20:5	IP Address	100	88	TRUE	2023-08-22 20:5	R_2w77FweLVEGMg7f							anonymous	EN	
2023-08-22 20:5	2023-08-22 20:5	IP Address	100	145	TRUE	2023-08-22 20:5	R_2CfvviNBCSwlXMw							anonymous	EN	
2023-08-22 20:5	2023-08-22 20:5	IP Address	100	130	TRUE	2023-08-22 20:5	R_2Uhc4o0yDNuHv0j							anonymous	EN	
2023-08-22 20:5	2023-08-22 21:0	IP Address	100	200	TRUE	2023-08-22 21:0	R_2YtYDFC1hyyHyeg							anonymous	EN	
2023-08-22 1:16:	2023-08-22 21:0	IP Address	100	71141	TRUE	2023-08-22 21:0	R_21hSyH9ZWwRhHe0							anonymous	EN	
2023-08-22 21:0	2023-08-22 21:1	IP Address	100	503	TRUE	2023-08-22 21:1	R_Rk00y9PmqeG5KLL							anonymous	EN	
2023-08-22 21:0	2023-08-22 21:1	IP Address	100	950	TRUE	2023-08-22 21:1	R_sj2FdDX5Z9I5FzH							anonymous	EN	
2023-08-22 21:1	2023-08-22 21:2	IP Address	100	460	TRUE	2023-08-22 21:2	R_2ts5TC6f98Esiq5							anonymous	EN	
2023-08-22 21:2	2023-08-22 21:2	IP Address	100	35	TRUE	2023-08-22 21:2	R_2RPlgkFDmiRzUsE							anonymous	EN	
2023-08-22 21:3	2023-08-22 21:3	IP Address	100	15	TRUE	2023-08-22 21:3	R_3g0Fuc76kf4Tdek							anonymous	EN	

2023-08-22 21:3	2023-08-22 21:3	IP Address	100	115	TRUE	2023-08-22 21:3	R_3q86QbEbbH1GYLg							anonymous	EN	
2023-08-22 21:2	2023-08-23 1:47:	IP Address	100	15687	TRUE	2023-08-23 1:47:	R_25Tq0kWLBt935HK							anonymous	EN	
2023-08-23 1:47:	2023-08-23 1:56:	IP Address	100	545	TRUE	2023-08-23 1:56:	R_OIqmBSXhrjNb8MF							anonymous	EN	
2023-08-23 2:13:	2023-08-23 4:25:	IP Address	100	7918	TRUE	2023-08-23 4:25:	R_2aJLN39Wb9i1l1r							anonymous	EN	
2023-08-23 4:25:	2023-08-23 4:36:	IP Address	100	665	TRUE	2023-08-23 4:36:	R_3etpzqjIXe25htz							anonymous	EN	
2023-08-23 4:36:	2023-08-23 4:41:	IP Address	100	287	TRUE	2023-08-23 4:41:	R_1jIkBtT6KB07DCT							anonymous	EN	
2023-08-23 20:1	2023-08-23 21:0	IP Address	100	3146	TRUE	2023-08-23 21:0	R_8pksmYr73RzFuXT							anonymous	EN	
