# Supplementary material for: Leveraging Canadian Health Care Worker Volunteers to Address COVID-19 Vaccine Misinformation on Facebook: Qualitative Program Evaluation Study
Source: J Med Internet Res. 2025 Jul 24;27:e65361. doi: 10.2196/65361 (PMC12288766; doi:10.2196/65361)
Supplement: Multimedia Appendix 3 [file jmir-v27-e65361-s003.docx]

"lambda,delta,myocarditis,ivermectin,natural immunity,aborted,fetuses,contamination,heart inflammation,children,infants,study,research"
