# Supplementary material for: Leveraging Canadian Health Care Worker Volunteers to Address COVID-19 Vaccine Misinformation on Facebook: Qualitative Program Evaluation Study
Source: J Med Internet Res. 2025 Jul 24;27:e65361. doi: 10.2196/65361 (PMC12288766; doi:10.2196/65361)
Supplement: Multimedia Appendix 4 [file jmir-v27-e65361-s004.docx]

**Initial dialogue**:

*Thank you for taking the time to participate in this interview today.* *My name is __________and I’m going to lead our discussion today. This will be a 30 minute interview during which I will ask you questions about your experience participating in the Informed Choice Project. This discussion will be used to evaluate the success of this project. All answers will be summarized into themes to ensure that no individual will be identified. This discussion will be recorded in order to allow us to go back and identify themes and analyze answers, but only those involved with the analysis will have access to these recordings. There are no right or wrong answers. You are encouraged to be honest and share your true thoughts and feelings. If at any time you feel uncomfortable or want to leave the study, you are free to do so.*

*Before we get started, does anyone have any questions?*

1. Why did you decide to get involved with this project?
2. Tell me a bit about your role on the project.
3. What has your experience been participating in the Informed Choice Project?
4. What were the strengths and weaknesses of this project?
5. What would you have done differently in order to improve the project?
   1. What were your experiences with the training?
   2. How could it have been improved?
   3. How were your experiences interacting with misinformation on social media?
   4. Did you find it easy/ hard to meet the required hours?
6. What have you learned about misinformation and social media?
7. Did you see any trends/patterns with the type of misinformation topics you were engaging with (i.e. were people scared, worried, angry etc.)?
8. What other techniques do you think would have been useful in combating misinformation online?

**Closing Comments:**

*Thank you for taking part in this interview, your discussion has been very helpful and appreciated. We are grateful to you for taking the time and sharing your experiences with me. If after we are done, there is something you still wish to share, or did not have the opportunity to discuss today, please email us at Hinna.Hasan@19tozero.ca.*
