# Supplementary material for: Leveraging Canadian Health Care Worker Volunteers to Address COVID-19 Vaccine Misinformation on Facebook: Qualitative Program Evaluation Study
Source: J Med Internet Res. 2025 Jul 24;27:e65361. doi: 10.2196/65361 (PMC12288766; doi:10.2196/65361)
Supplement: Multimedia Appendix 5 [file jmir-v27-e65361-s005.docx]

**Codes:**

- **Personal interest** — describes why participants decided to volunteer with ICP. (E.gThey were a health care provider, interest in health, aspiring PH professionals etc.)
- **Self-paced model**— comments surrounding the self-paced completion aspect of the ICP program model. This could be positive or negative feelings towards it (e.g., convenience, low time commitment, excel breakdowns, meeting requirements etc.)
- **Barriers to completion** — anything that the participants experienced that prevented them from completing their hours for ICP or made it difficult (e.g., procrastination, issues with technology, busy lives)
- **Training strengths** — describes any positive aspects of the ICP training that the participants experienced (e.g., virtual training convenience, enjoyed interactive components, easy to access coordinators, resources, etc.)
- **Training weaknesses** — describes any negative aspect of ICP **training** that the participants experienced
- **Suggested program improvements** — comments on how to correct any weaknesses that the participants identified in the training or the broader ICP program (e.g., access to program leads, refresher courses, collaboration with peers)
- **Public Emotion** — when participants interacted with the public they were faced by emotional responses from some. (e.g., sentiments could include anger, fear, distrust, anxiety)
- **Real-world benefits** — Positive influences of the program that the participants saw in their daily lives (e.g., geared towards HCPs who participated in the training and then implemented their learnings in clinical settings)
- **Personal growth** — Positive growth that the participants experienced after taking part in ICP (e.g., confidence, ability to identify misinformation)
- **Anonymity** – Sentiment around the privacy of those involved (e.g., not using personal accounts, not sharing personal data, etc.)
- **Misinformation** – Prevalence of misinformation on social media and the participants experiences combatting this online. Including topics that they saw discussed online, their strategies in responding to the misinformation, false equivalence, etc.
- **Trust** – Trust networks, lack of trust, anti-establishment etc.
